# BrdU immuno-tagged G-quadruplex ligands: a new ligand-guided immunofluorescence approach for tracking G-quadruplexes in cells

**DOI:** 10.1093/nar/gkab1166

**Published:** 2021-12-07

**Authors:** Thibaut Masson, Corinne Landras Guetta, Eugénie Laigre, Anne Cucchiarini, Patricia Duchambon, Marie-Paule Teulade-Fichou, Daniela Verga

**Affiliations:** CNRS UMR9187, INSERM U1196, Institut Curie, PSL Research University, F-91405 Orsay, France; CNRS UMR9187, INSERM U1196, Université Paris-Saclay, F-91405 Orsay, France; CNRS UMR9187, INSERM U1196, Institut Curie, PSL Research University, F-91405 Orsay, France; CNRS UMR9187, INSERM U1196, Université Paris-Saclay, F-91405 Orsay, France; CNRS UMR9187, INSERM U1196, Institut Curie, PSL Research University, F-91405 Orsay, France; CNRS UMR9187, INSERM U1196, Université Paris-Saclay, F-91405 Orsay, France; CNRS UMR9187, INSERM U1196, Institut Curie, PSL Research University, F-91405 Orsay, France; CNRS UMR9187, INSERM U1196, Université Paris-Saclay, F-91405 Orsay, France; CNRS UMR9187, INSERM U1196, Institut Curie, PSL Research University, F-91405 Orsay, France; CNRS UMR9187, INSERM U1196, Université Paris-Saclay, F-91405 Orsay, France; CNRS UMR9187, INSERM U1196, Institut Curie, PSL Research University, F-91405 Orsay, France; CNRS UMR9187, INSERM U1196, Université Paris-Saclay, F-91405 Orsay, France; CNRS UMR9187, INSERM U1196, Institut Curie, PSL Research University, F-91405 Orsay, France; CNRS UMR9187, INSERM U1196, Université Paris-Saclay, F-91405 Orsay, France

## Abstract

G-quadruplexes (G4s) are secondary structures forming in G-rich nucleic acids. G4s are assumed to play critical roles in biology, nonetheless their detection in cells is still challenging. For tracking G4s, synthetic molecules (G4 ligands) can be used as reporters and have found wide application for this purpose through chemical functionalization with a fluorescent tag. However, this approach is limited by a low-labeling degree impeding precise visualization in specific subcellular regions. Herein, we present a new visualization strategy based on the immuno-recognition of 5-bromo-2′-deoxyuridine (5-BrdU) modified G4 ligands, functionalized prior- or post-G4-target binding by CuAAC. Remarkably, recognition of the tag by antibodies leads to the detection of the modified ligands exclusively when bound to a G4 target both *in vitro*, as shown by ELISA, and in cells, thereby providing a highly efficient G4-ligand Guided Immunofluorescence Staining (G4-GIS) approach. The obtained signal amplification revealed well-defined fluorescent foci located in the perinuclear space and RNase treatment revealed the preferential binding to G4-RNA. Furthermore, ligand treatment affected significantly BG4 foci formation in cells. Our work headed to the development of a new imaging approach combining the advantages of immunostaining and G4-recognition by G4 ligands leading to visualization of G4/ligands species in cells with unrivaled precision and sensitivity.

## Graphical Abstract

**Graphical Abstract:**
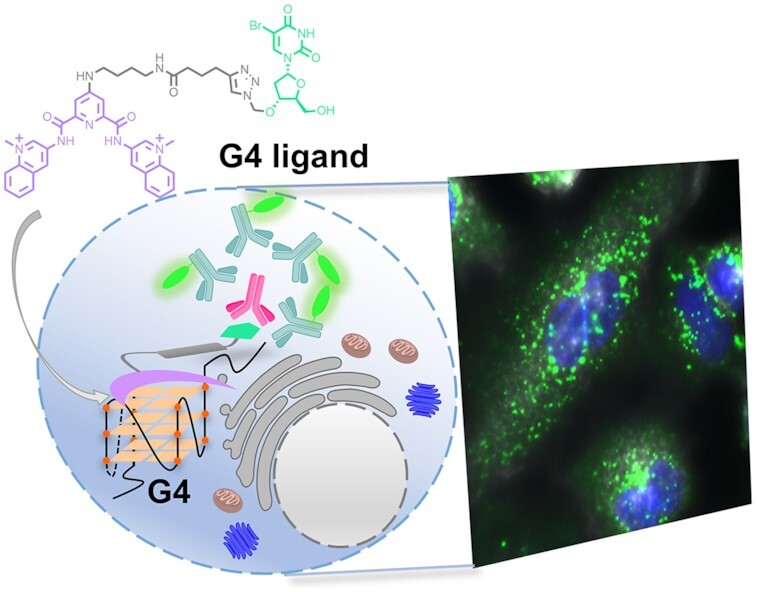
G4-GIS methodology: a new visualization strategy of G4 targets in cells based on the immuno-recognition of 5-bromo-2′-deoxyuridine (BrdU)-modified G4 ligands.

## INTRODUCTION

G-quadruplexes (G4s) are four-stranded nucleic acid secondary structures adopted by repetitive G-rich DNA and RNA sequences, which fold over upon coordination with monovalent cations (1,2). These structures are believed to play relevant biological functions during DNA transactions, such as replication, transcription, repair and recombination, and are considered to serve as regulatory elements able to modulate splicing and translation in mRNAs (3–8). However, despite accumulation of evidence for the *in vitro* existence of G-quadruplexes, their *in vivo* occurrence as well as their consensus sequence is still a matter of debate. In this regard, bioinformatics studies predict the existence of a large number of G4 structures within both human genome and transcriptome. Genome-wide, G4s were found in promoters, coding regions, introns and untranslated (UTRs) regions of genes and intergenic regions (9–11). Importantly, these findings permitted to highlight as well the presence of these structures in the protein coding and non-coding regions of the transcriptome (12). More recently by means of G4-specific antibodies, alone or in combination with G4 selective small molecules (G4 ligands), next-generation sequencing (NGS) experiments have helped to gain understanding into G4 structure frequency and functional relevance in both human genome and transcriptome, localizing them mainly in gene promoter regions, as well as in telomeres, and 5′-untranslated regions (UTRs) of mRNAs (13–19). High-throughput *in vitro* polymerase stop assay allowed the identification of 716 310 potential DNA quadruplex-forming sequences in the human genome (G4-Seq) (16). A significant reduced number of G4 structures was detected by G4 ChiP-Seq, where only 10 000 G4 sites specifically enriched in nucleosome-depleted regions were identified (17). In addition, several studies aimed at characterizing G4-RNA landscape in the human transcriptome have reported numbers of quadruplex-forming sequences comprised between 3 000 and >10 000 (18,20).

This large body of data undeniably suggest that small synthetic molecules selective for G4 structures represent highly valuable chemical biology tools necessary to unveil G4 biological functions. This is why strong effort has been focused on the design and synthesis of selective G4 ligands that can be used as G4 probes when combined with genomic and transcriptomic approaches (21,22). To date, only a few studies have investigated the changes produced by G4 targeting at transcriptional genome-wide level showing either down- or up-regulation of a large number of genes containing G4 putative forming sequences (23,24). In spite of these results, the large number of G4s identified by NGS analyses makes highly challenging the exact identification of the G4 structures targeted by G4 ligands in cells. Therefore, within this context, development of imaging techniques combined with G4 selective fluorescent tools are absolutely required to address this issue. Several imaging approaches have been devised for tracking G4 ligand distribution in cells. Numerous studies employ ON/OFF fluorescent probes, which lights up upon binding to the G4 target, as reported for QUMA-1, N-TASQ and for coumarin-quinazoline derivative (25–27). Another detection method recently proposed makes use of fluorescence lifetime imaging (FLIM), which allows visualizing the variation of the fluorescence lifetime of DAOTA-M2 when bound to different DNA topologies in cells (28). Two of the most selective G4 ligands developed so far, PDS and PhenDC3, are not fluorescent or are characterized by a very low intrinsic fluorescence strongly quenched upon binding to G4 structures, which makes them very difficult or impossible to visualize by classical fluorescence microscopy (29,30). Therefore, several labeling methodologies based on the synthesis of fluorescently tagged G4 ligands have been proposed. Once tagged, compound cellular distribution can be followed by either confocal or by the most recent HiLo microscopy (31). However, introducing a fluorophore in a small synthetic compound can produce unwanted effects, such as modifying physicochemical properties and, even more worrisome, dramatically affect biological activity. A strategy used to overcome these problems is *in situ* functionalization (32–34). This synthetic approach allows tethering the fluorophore to the G4 ligand of interest by means of a bioorthogonal reaction conducted directly in cells after target recognition by the ligand. Copper-catalyzed 1,3-dipolar azide-alkyne cycloaddition (CuAAC), or copper-catalyzed click reaction, offers an elegant strategy for *in situ* functionalization. Although the necessity of using a large excess of copper(I) salts limits the application of CuAAC in fixed cells, pioneering studies reported its use to functionalize G4 ligands with fluorescent tags allowing identification of G4 targets in both DNA and RNA (35,36). Despite these important developments, *in situ* functionalization did not allow to overcome the detection limit of the method. As a matter of fact, the spatial resolution of fluorescence microscopies (confocal or wide field) is insufficient to detect single complexes formed by tagged-G4 ligands bound to G4 structures unless the latter are present in multiple copies (37). Therefore, signal amplification is an absolute requirement to allow G4 imaging convincingly and with higher precision.

**Figure 1.**
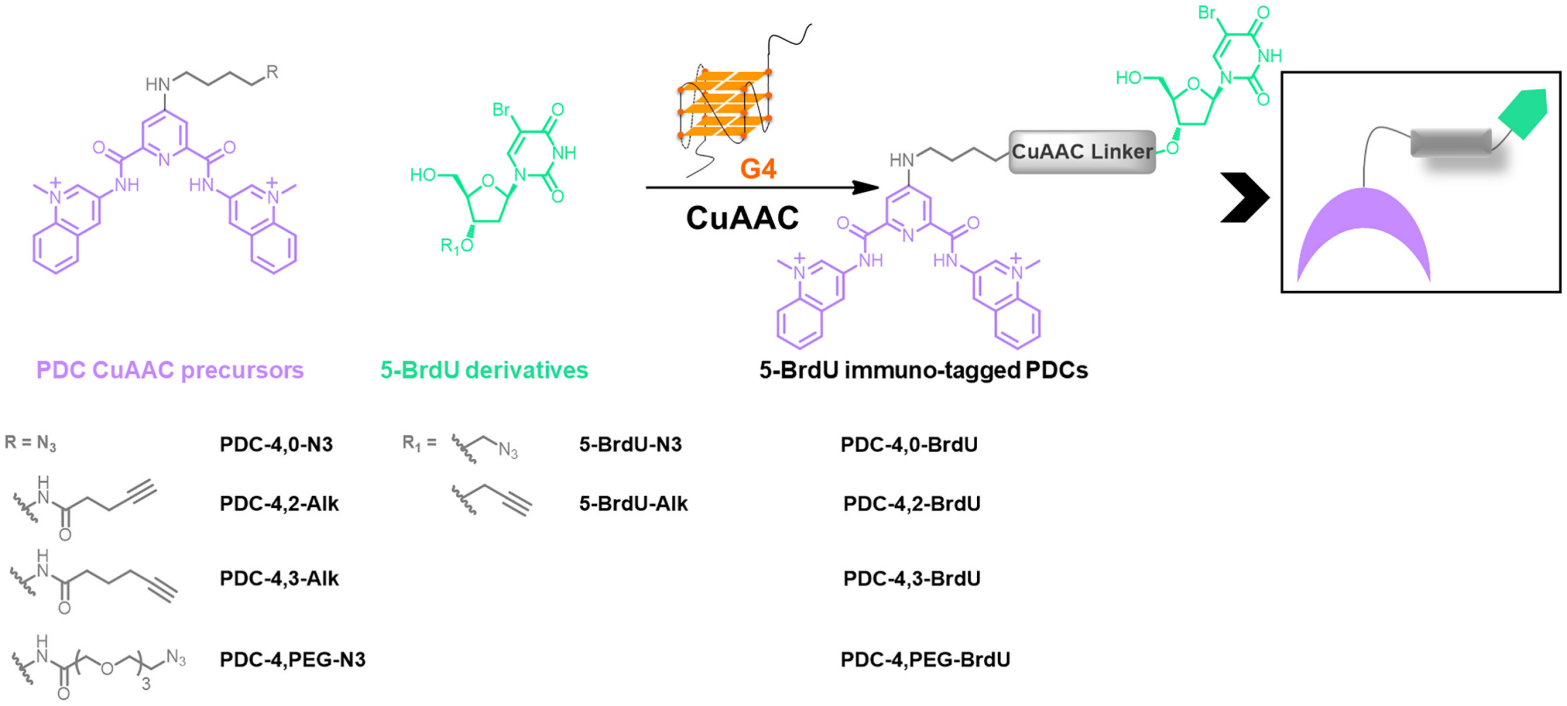
(Left) Structures of PDC CuAAC precursors and 5-BrdU partners (4 stands for the chain length between the PDC core and the amide function and 0, 2, 3 CH_2_ or PEG for the chain length between the amide and the terminal function). (Right) Schematic representation of 5-BrdU PDC conjugates obtained via *in situ* functionalization in the presence of the G4 target or prepared in solution (*ex situ*).

In this study, we proposed the development of a new visualization strategy called G4 ligand Guided Immunofluorescence Staining (G4-GIS) that capitalizes both on specific recognition by small molecule and antibody signal amplification properties. This unprecedented integrated approach is expected to be more efficient in systematically identifying G4 ligand targets in cells. By using the Pyrido Dicarboxamide (PDC) core as selective G4 ligand, we synthesized four hapten modified PDC conjugates and four PDC CuAAC precursors for *in situ* functionalization, which selectively bind G-quadruplex structures (38–41). As hapten the synthetic molecule 5-bromo-2′-deoxyuridine (5-BrdU) was chosen and called herein immuno-tag, which guarantees ligand recognition by a commercially available antibody and, as a consequence, the development of the immunofluorescence methodology. The four PDC conjugates were prepared *ex situ* following a one-step synthetic protocol from their clickable precursors (Figure 1, right) or *in situ* via a two-step synthesis after G4 target recognition (Figure 1, left), in both cases the 5-BrdU moiety was introduced by employing copper-catalyzed click reaction. Antibody recognition was validated *in vitro*. We hypothesized that PDC derivatives, after efficient internalization and diffusion within cells, reach RNA and DNA G4 targets. Subsequently, after submitting PDC precursors to CuAAC in the presence of the opportune 5-BrdU partner, the G4 ligands are recognized by anti 5-BrdU antibody permitting secondary antibody recognition and fluorescent detection. Importantly, this approach allows not only the comparison of G4 ligand distribution after and prior 5-BrdU functionalization but also to point out regions of weak ligand accumulation, hardly visible by direct fluorophore functionalization, providing a new useful approach of high sensitivity for G4 ligand target detection.

## MATERIALS AND METHODS

### Förster Resonance Energy Transfer (FRET)-melting assay

The experiments were carried out on pre-folded G-quadruplex structures: the sequences were heated at 95°C for 10 min in opportune buffer and left to fold in ice for 30 min. Duplex DNA and transfer RNA (tRNA) sequences were heated at 95°C for 10 min and left to cool down to room temperature over a period of 3 h. Buffer conditions are specified below for each G4 sequence.

As previously reported, stabilization of ligands on G4-structures was monitored via FRET-melting assay performed in 96-well plates on a real-time PCR apparatus, 7900HT Fast Real-Time PCR system. This assay was carried out with oligonucleotides mimicking biological relevant G4-forming sequences, labeled with FRET partners at each end (5′-FAM (donor) and 3′-TAMRA (acceptor)) in the presence of G4 ligands. To determine ligand G4 versus duplex selectivity, an unlabeled DNA competitor ds26 was used. In order to assess RNA-G4 versus RNA single strand selectivity, an unlabeled tRNA sequence was used. This oligonucleotide consists of an average of 85 nucleobases and was used at ≃3 μM (≃15 mol equiv, ≃60 nucleobases equiv). Each well was filled with: 0.20 μM (1 equiv) labeled G4 DNA or RNA, 1 μM (5 equiv) of ligand, and *ds*DNA competitor at 0, 3 and 10 μM (0, 15 and 50 mol equiv) respectively, or RNA competitor at 0 and 3 μM, in a total volume of 25 μl. Solutions were prepared in buffer containing 10 mM Li.Caco pH 7.2, and 10 mM KCl, completed by 90 mM LiCl for F21T, F21CTAT and 1 mM KCl, completed by 99 mM LiCl for all the others G-quadruplex sequences. The FAM channel was used to collect the fluorescence signal. Stabilization of compounds with G4-structures was monitored as followed: 5 min at 25°C and then increase of 0.5°C every minute until reaching 95°C. The stabilization was evaluated by determining the temperature at half denaturation of the G4 (obtained at the half height of the sigmoidal curve, Δ*T*_1/2_) in the absence and in the presence of ligand, duplex DNA, and tRNA competitor, by using Origin 2021.

A series of oligonucleotides covering a range of possible G4 conformations was used.

### Fluorescent intercalator displacement assay (G4-FID)

The experiments were carried out on pre-folded G-quadruplex structures: the sequences were heated at 95°C for 10 min (10 mM Li.Caco pH = 7.3 and 100 mM KCl) and left to fold in ice for 30 min. Duplex DNA was heated at 95°C for 10 min (10 mM Li.Caco pH = 7.3, and 100 mM KCl) and left to cool down to room temperature over a period of 3 h.

FID-assay was performed on a Cary Eclipse spectrofluorometer, using 1 ml quartz cell as previously reported. About 0.25 μM of folded DNA prepared in a solution of 10 mM Li.Caco pH = 7.3 and 100 mM KCl was mixed with 0.50 μM Thiazole Orange (TO) for G4-DNA and 0.75 μM for duplex DNA to obtain a total volume of 1 ml. The solution was left to stand in the dark for 30 min and then the titration was started. Each addition of ligand (from 0.5 to 10 equiv, stock solution 0.125 μM in DMSO) was followed by a 3 min equilibration time and fluorescence emission spectra were recorded from 510 to 750 nm (λ_exc_ = 495 nm, 1.0 nm increment, 0.1 s integration time and slit 5 nm). The percentage of TO displacement was calculated as follows: TOD (%) = 100 – (*F* / *F*_0_) × 100, where *F*_0_ is the maximum fluorescence emission area of bound TO before addition of ligand, and *F* is the fluorescence emission area after each ligand addition. The percentage of TO displacement was then plotted as a function of the concentration of added ligand.

A series of oligonucleotides covering a range of possible G4 conformations and a duplex DNA control were used.

### Click chemistry protocols

#### General experimental Cu-catalyzed click chemistry

Tris(3-hydroxypropyltriazolylmethyl)amine (THPTA) and CuSO_4_.5H_2_O were bought from Sigma-Aldrich. Stock solutions of PDC-click precursors (PDC-4,2-Alk, PDC-4,3-Alk, PDC-4,0-N3 and PDC-4,PEG-N3) and 5-BrdU-click partners (5-BrdU-Alk and 5-BrdU-N3) were prepared in DMSO at 1 mM concentration, and stored in the dark at −20°C. For all the following experiments, copper-catalyst (CuSO_4_.5H_2_O), THPTA and sodium ascorbate solutions were freshly prepared in ultrapure H_2_O at respectively 10 mM for the CuSO_4_.5H_2_O solution, 50 mM for the THPTA solution and 40 mM for the sodium ascorbate solution. Oligonucleotide sequences were purchased from Eurogentec as dried samples purified by HPLC-RP. Oligonucleotides were dissolved in MilliQ water at 200 μM concentration and stored at −20°C. Oligonucleotide sequences used for click reaction *in situ* are the following: c-Myc22 (G14T-G23T): TGAGGGTGGGTAGGGTGGGTAA, 22AG: AGGGTTAGGGTTAGGGTTAGGG and TERRA: AGGGUUAGGGUUAGGGUUAGGG.

Oligonucleotides were folded and CuAAC reactions were carried out in K10 buffer (10 mM Li.Caco pH 7.2, 10 mM KCl, 90 mM LiCl).

Solid-phase extraction (SPE) was performed with Clarity® OTX (Phenomenex) columns. To facilitate elution of the fractions, a manifold device as vacuum source was employed (*P* = 0.4 Bar).

#### General protocol for Cu-catalyzed click reaction (CuAAC) in the absence of G-quadruplex

In a 1.5 ml Eppendorf tube, CuSO_4_.5H_2_O (1.5 μl, 10 mM in H_2_O) and THPTA (1.5 μl, 50 mM in H_2_O) were added. The mixture was stirred and centrifuged to obtain a blue solution. Afterward, sodium ascorbate (1.5 μl, 40 mM in H_2_O) was added, and the mixture was stirred and centrifuged again to give a colorless solution. The latter was diluted with K10 buffer (11.6 μl). In a second 1.5 ml Eppendorf tube, the PDC CuAAC derivative (0.75 μl, 1 mM in DMSO) was added to K10 buffer (11.6 μl). The previously prepared copper solution was subsequently added. After stirring and centrifuging, the 5-BrdU partner was added (1.5 μl, 1 mM in K10). The reaction mixture was left to stand for 1 h at room temperature without agitation to enable the click reaction to occur. After 1 h, the reaction mixture was treated by SPE.

#### General protocol for Cu-catalyzed click reaction (CuAAC) in the presence of G-quadruplex target

Oligonucleotide solutions (100 μM) were prepared in K10 buffer. The sequences were folded in their G4 structure by heating the oligonucleotide solutions at 95°C for 10 min. The samples were then cooled down in ice for 30 min. In a 1.5 ml Eppendorf tube, the folded G4 (7.5 μl, 100 μM in K10 buffer), the PDC CuAAC derivative (0.75 μl, 1 mM in DMSO) and K10 buffer (7.9 μl) required for the click reaction were successively added. The mixture was left to stand for 30 min at room temperature without agitation. In another 1.5 ml Eppendorf tube, CuSO_4_.5H_2_O (1.5 μl, 10 mM in H_2_O) and THPTA (1.5 μl, 50 mM in H_2_O) were added. The mixture was stirred and centrifuged to obtain a blue solution. Sodium ascorbate (1.5 μl, 40 mM in H_2_O) was added and the mixture was stirred and centrifuged again to give a colorless solution. The latter was diluted with K10 buffer (7.85 μl). The copper-containing solution was then added to the G4-containing tube, and the mixture was stirred and centrifuged. Then, 5-BrdU partner was added (1.5 μl, 1 mM in K10). The reaction mixture was stirred, centrifuged, and left to stand for 1 h at room temperature without agitation to enable the click reaction to occur. After 1 h, the reaction mixture was treated by SPE.

#### Solid-phase extraction

SPE column was first conditioned as reported by the supplier (1 ml of MeOH then 1 ml of ultrapure H_2_O). The click reaction sample was diluted with 270 μl of H_2_O to reach a final volume of 300 μl and loaded on the SPE column. Then, small and medium size molecules were eluted by successively washing the SPE column with: 3 × 500 μl of [H_2_O/MeCN 1:4 (v/v)], 1 × 500 μl of MeCN and finally 1 × 1 ml of MeOH. The loading and elution fractions were collected together in a single 15 ml plastic tube. The solvent was removed under reduced pressure at 30°C for 3 h. The residue was dissolved in 30 μl of ultrapure H_2_O and analyzed by UHPLC.

#### UHPLC method and chromatograms

About 3 μl of the reaction solution were injected in an Ultra-High-Performance Liquid Chromatography (UHPLC) apparatus: Thermo UltiMate 3000, equipped with a Thermo UV/visible detector (Chromeleon^©^ software, Version 7.2.6.10049). The system was equipped with a Phenomenex Luna^®^ OMEGA Polar C18, 1.6 μm, 100 × 2.1 mm column. Elution conditions: column temperature = 40°C, flow rate = 0.4 ml/min, use of a linear A–B gradient (eluant A: 0.1% formic acid in water; eluant B: 0.1% formic acid in acetonitrile; UHPLC method: (a) 20% B isocratic for 1 min, (b) 20–80% B gradient for 4 min, (c) 80% B isocratic for 1.5 min and (d) 80–20% B gradient for 0.5 min). The chromatograms were recorded at two different wavelengths: 254 and 350 nm.

### Oligonucleotides, antibodies and cell markers

Oligonucleotides were folded in 10 mM Tris.HCl pH 7.4, 100 mM KCl at 5 μM concentration by heating up at 95°C during 10 min and by letting slowly cool down to 25°C. pSANG10–3F-BG4 plasmid encoding the BG4 single chain antibody was a gift from Shankar Balasubramanian (Addgene plasmid#55756; http://n2t.net/addgene:55756;RRID:Addgene_55756). Enzyme-linked immunosorbent assay was performed with Pierce Streptavidin Coated High Capacity Plates, Clear, 96-Well (15500, Thermo Fisher Scientific). For validation of BG4 and binding in competition with G4 ligands, BG4 was used at increasing concentrations (10, 20, 50, 75, 100, 150 and 200 nM) and detected using HRP-conjugated anti-FLAG antibody (1:10 000 dilution, ab1238, Abcam). For immunofluorescence experiments, BG4 was used at 20 nM and detected using rabbit anti-FLAG (1:800 dilution, 2368S, Cell Signaling) and Alexa Fluor 555-conjugated goat anti-rabbit (1:1 000 dilution, A27039, Invitrogen) antibodies. 5-BrdU was detected using mouse anti-5BrdU (1:250 dilution for immunofluorescence, 1:150 dilution for ELISA assay, 555627, BD Pharmingen) and Alexa Fluor 488-conjugated goat anti-mouse antibodies (1:500 dilution, A28175, Invitrogen) or HRP-conjugated goat anti-mouse antibody (1:5 000 dilution, 115–036-003, Jackson ImmunoResearch). Cell membrane was detected using wheat germ agglutinin CF®640R WGA (5 μg/ml incubation concentration, 29026, Biotium). TMB solutions as chromogenic reagents for peroxidase designed for ELISA techniques were used as suggested by the supplier (34021, Thermo Fisher Scientific). Cells were seeded in 8-well chamber slides (80841, ibidi-cells in focus), and slides were mounted with coverslips using vectashield antifade mounting agent with DAPI (H-1200, Eurobio Scientific).

### BG4 purification

The BG4-encoding plasmid (Psang10-3f-BG4) was transformed into *Escherichia coli* strain Rosetta 2 pLys (Novagen), followed by culturing in 2xYT and kanamycin (50 μg/ml) and chloramphenicol (34 μg/ml) at 37°C and 250 RPM. When OD600 reached 0.6–0.8 the temperature was lowered to 20°C, and induction was initiated with 0.5 mM IPTG. After 15 h of induction at 20°C, bacterial culture was harvested by spinning at 4 000 *g* for 30 min. Pellets were resuspended in 8 ml TES buffer 1 (50 mM Tris-HCl pH 8.0, 1 mM EDTA pH 8.0, 20% sucrose) per 100 ml volume expression culture. The mixture was put in ice for 10 min. About 12 ml of TES buffer 2 (10 mM Tris-HCl pH 8.0, 0.2 mM EDTA pH 8.0, 4% sucrose, with benzonase nuclease and 2 mM MgSO_4_) per 100 ml volume expression culture were added and the mixture was put in ice for 15 min. Cells were centrifuged at 4°C for 10 min at 8000 *g*. Supernatant was collected and filtered (0.22 μm). The sample is deposited on a column FF Crude His trap (nickel affinity, Cytiva) was washed and preequilibrated with PBS. Resin was then washed with 20 CV of washing buffer high salt (PBS, 350 mM NaCl, 10 mM imidazole pH 8.0) and 20 CV buffer low salt (PBS, 10 mM imidazole pH 8.0). BG4 protein was eluted with elution buffer (PBS, 250 mM imidazole pH 8.0), buffer-exchanged to PBS and concentrated using 50 ml centrifugal devices with 10 kDa cut-off (PALL Corporation). Protein quality was assessed by running SDS-PAGE (4–20% TGX gel Biorad), and gels were visualized with Coomassie staining. The concentrated protein was purified on HiLoad superdex 75 pg 26/600 Cytiva. Peak protein fractions were confirmed using SDS-PAGE, pooled and concentrated as described above. Final protein concentration was calculated based on absorbance measurements at 280 nm, with an absorbance ratio at 260/280 nm <0.7 indicating no nucleic acid contamination.

ELISAs for validation of BG4 binding affinity were performed using a standard method reported in literature, by employing HPLC purified biotinylated sequences purchased from Eurogentec (13).

### Enzyme-linked immunosorbent assay (ELISA)

#### Immunodetection of PDC-4,3-BrdU

About 5 μM biotinylated hTelo, c-Myc, TERRA, 22Agmut, TERRAmut, ihTelo and hp were annealed in 10 mM Tris HCl, pH 7.4, 100 mM KCl by slow cooling from 95 to 25°C. Biotinylated oligonucleotides solutions were diluted to 0.1, 1, 5, 10, 20, 50, 75 and 100 nM in 10 mM Tris HCl, pH 7.4, 100 mM KCl and bound to streptavidin-coated plates during 1 h. Immobilized hTelo, c-Myc, TERRA, 22Agmut, TERRAmut, ihTelo and hp were incubated with 250 nM PDC-4,3-BrdU solution in 10 mM Tris HCl, pH 7.4, 100 mM KCl during 1 h. After washing steps with ELISA buffer (100 mM KCl + 50 mM KH_2_PO_4_), plates were blocked with blocking buffer (3% BSA in ELISA buffer) during 1 h. After blocking, plates were incubated with mouse anti-5BrdU antibody (1/150 dilution) for 1 h. After washing steps with ELISA buffer supplemented with 0.1% Tween 20, goat anti-mouse horseradish peroxidase (HRP)-conjugated antibody (1/5 000) was incubated for 1 h. After washing (ELISA buffer + 0.1% Tween 20), binding was visualized with TMB (3,3′,5,5′-tetramethylbenzidine, HRP substrate, Thermo Fisher Scientific). Reaction was stopped with 2 N HCl and signal intensity was measured at 450 nm on a FLUOstar microplate reader (BMG Labtech).

Oligonucleotide sequences employed: Biot-hTelo – GGTTAGGGTTAGGGTTAGGGTTAGGGTTAGG, Biot-cMyc – TGAGGGTGGGTAGGGTGGGTAA, Biot-TERRA – UUAGGGUUAGGGUUAGGGUUAGG G; Biot-22Agmut – ATGGTTAGTGTTAGGTTTAGTG, Biot-TERRAmut – UUACCGUUACCGUUACCGUUACCG, Biot-ihTelo – CCCTAACCCTAACCCTAACCCT; Biot-hp – CAGUACAGAUCUGUACUG.

#### Detection of G4 in the presence of G4 ligands

ELISAs for binding affinity and specificity were performed using a modified method already reported in literature. About 5 μM biotinylated hTelo and c-Myc were annealed in 10 mM Tris HCl, pH 7.4, 100 mM KCl by slow cooling from 95 to 25°C. Biotinylated oligonucleotides solutions were diluted to 50 nM in 10 mM Tris HCl, pH 7.4, 100 mM KCl and bound to streptavidin-coated plates during 1 h. For competition experiments performed in the presence of G4 ligands (PDC-4,3-Alk, PhenDC3, and PDS), bound biotinylated hTelo and c-Myc were incubated with 250 nM ligand solution in 10 mM Tris HCl, pH 7.4, 100 mM KCl during 1 h. After washing steps with ELISA buffer (100 mM KCl, 50 mM KH_2_PO_4_), plates were blocked with blocking buffer (3% BSA in ELISA buffer) during 1 h. After blocking, plates were incubated with 0, 10, 20, 50, 75 and 100 nM BG4 antibody during 1 h. Washing steps (ELISA buffer + 0.1% Tween 20) were followed by incubation with HRP-conjugated anti-FLAG antibody (1/10 000 dilution) for 1 h. After washing (ELISA buffer + 0.1% Tween 20), plates were incubated with TMB (3,3′,5,5′-tetramethylbenzidine, HRP substrate) and reaction blocked as previously described. Signal intensity was measured at 450 nm on a FLUOstar microplate reader (BMG Labtech).

Oligonucleotide sequences employed: Biot-hTelo – GGTTAGGGTTAGGGTTAGGGTTAGGGTTAGG, Biot-cMyc – TGAGGGTGGGTAGGGTGGGTAA.

### Cell culture

A549 (ATCC® CCL-185™) cell lines were purchased from the American Type Culture Collection (ATCC, Manassas, VA, USA,) and cultured in Dulbecco’s modified Eagle’s medium GlutaMAX™ (DMEM, Life Technologies, Saint-Aubin, France) supplemented with 10% fetal bovine serum (FBS, South America origin, BioWhittaker® Lonza, Verviers, Belgium) and 1% Penicillin/Streptomycin (Life Technologies) in humidified atmosphere under 5% CO_2_ in air at 37°C. Cells were split twice a week. Cell splitting: cells were washed with Phosphate Buffer Solution (PBS, Life Technologies) and 2 ml of TrypLE Express Enzyme (Life technologies) was added for 10 min at 37°C. Cells were collected in warm medium (addition of 8 ml) by gently pipetting. Add appropriate aliquots of the cell suspension to new culture vessels. Cells were re-suspended in medium and seeded at a density of 0.5–1 million cells in a T75 culturing flask. Cells were counted using a MoxiZ™ mini automated cell counter (Orflo®, Ketchum, ID, USA).

A2780 cell lines were purchased from the American Type Culture Collection (ATCC, Manassas, VA, USA) and cultured in Roswell Park Memorial Institute (RPMI, Life Technologies, Saint-Aubin, France) supplemented with 10% fetal bovine serum (FBS, South America origin, BioWhittaker® Lonza, Verviers, Belgium) and 3% of MIX (glutamine [2 mM], streptomycin [100 UI/ml], sodium bicarbonate [750 mg/ml]) and 1% Penicillin/Streptomycin (Life Technologies) in humidified atmosphere under 5% CO_2_ in air at 37°C. Cells were split twice a week. Cell splitting: cells were washed with Phosphate Buffer Solution (PBS, Life Technologies), and 1 ml of TrypLE Express Enzyme (Life technologies) was added for 4 min at 37°C. Cells were collected in warm medium (addition of 9 ml) by gently pipetting. Add appropriate aliquots of the cell suspension to new culture vessels. Cells were re-suspended in medium and seeded at a concentration of 0.2 × 10^5^ cells/ml in a T25 culturing flask. Cells were counted using a MoxiZ™ mini automated cell counter (Orflo®, Ketchum, ID, USA).

### Cell survival experiments

A total of 20 000 A549 cells per well in 1 ml of culture medium were seeded in 24-well cell culture plates. After incubation at 37°C in a humidified atmosphere with 5% CO_2_ for 24 h, solutions of PDC-derivatives (stock solution at 10 mM in DMSO and solutions at 500 and 2 000 μM prepared in water by subsequent dilutions) were added to the cells to reach final concentrations of 1, 5, 10, 15, 20 and 30 μM. After incubation for 72 h at 37°C, culture medium was removed and cells were washed with PBS. Afterward, 100 μl of TrypLE Express Enzyme was added to each well, followed by 5 min incubation at 37°C until the cells were thoroughly dissociated from the bottom of the plate. About 900 μl of fresh culture medium was added to stop the reaction, and living cells were counted by using a MoxiZ™ mini automated cell counter (Orflo®, Ketchum, ID, USA).

### Immunofluorescence detection of 5BrdU immuno-tag modified PDC

For immunostaining, 15 000 A549 or A2780 cells were seeded and grown in 8-well chamber slides over 24 h. Cells were treated with 5, 10 and 15 μM of PDC-derivatives. After 4, 8, 16, 24 or 48 h of incubation, cells were washed two times with PBS, fixed with 4% PFA in PBS for 15 min and permeabilized with 0.25% Triton X-100 in PBS for 15 min. For samples containing PDC 5-BrdU functionalized compounds (PDC-4,2-BrdU, PDC-4,3-BrdU, PDC-4,0-BrdU and PDC-4,PEG-BrdU): cells were washed three times with PBS and blocked with blocking buffer (5% BSA in PBS) for 2 h at room temperature, then incubated with mouse anti 5-BrdU (1/250) for 1 h at room temperature in blocking buffer. Slides were then washed three times with PBS and incubated with Alexa Fluor 488 goat anti-mouse (1/500) at room temperature for 40 min in blocking buffer. Slides were washed five times with PBS and mounted. For samples containing PDC CuAAC precursors, cells were incubated with the click reaction mixture in the dark for 1 h. For 500 μl final volume per well in PBS: 0.1 mM of CuSO_4_.5H_2_O (2.5 μl of a 20 mM solution in water) was mixed with 0.5 mM of THPTA (5 μl of a 50 mM solution in water) and left at room temperature for 1 h. To the previous solution, 5 mM of sodium ascorbate (25 μl of a 100 mM solution in water) was added and left to react for 30 min at room temperature. Subsequently, 5-BrdU derivative was added to a final concentration of 50 μM and the solution added to the cells. Negative controls of CuAAC reaction were obtained preparing the click reaction mixture omitting the addition of CuSO_4_.5H_2_O. Slides were washed with PBS and the same immunostaining protocol described above was followed.

### Comparison of Cy5-functionalized and 5BrdU-functionalized PDC-derivatives by Cu-catalyzed click reaction: distribution in cellular compartments

#### Cy5-functionalized samples

About 15 000 A549 cells were seeded and grown in 8-well chamber slides (80841, ibidi-cells in focus) over 24 h. Cells were treated with 5 μM of PDC-4,3-Alk. After 16 h of incubation, cells were washed two times with PBS, fixed with 4% PFA in PBS for 15 min and permeabilized with 0.25% Triton X-100 in PBS for 15 min. Cells were incubated with the click reaction mixture in the dark for 1 h. For 500 μl final volume per well in PBS: 0.1 mM of CuSO_4_.5H_2_O (2.5 μl of a 20 mM solution in water) was mixed with 0.5 mM of THPTA (5 μl of a 50 mM solution in water) and left at room temperature for 1 h. To the previous solution 5 mM of sodium ascorbate (25 μl of a 100 mM solution in water) was added and left to react for 30 min at room temperature. Subsequently, Cy5-N3 (Cy5-N3 A3030, Luminoprobe) was added to a final concentration of 10 μM. Slides were washed five times with PBS and mounted with coverslips using vectashield antifade mounting agent with DAPI.

#### BrdU-functionalized samples

Samples were prepared and staining performed as described above. To compare the staining results with the Cy5-functionalized compounds, Cu-catalyzed click reactions were performed in the presence of 10 μM of 5BrdU-derivatives.

### RNase A-treated cells

About 15 000 A549 cells were seeded and grown in 8-well chamber slides over 24 h. Cells were treated with 5 μM of PDC-derivatives. After 16 h of incubation, cells were washed two times with PBS, fixed with 4% PFA in PBS for 15 min and permeabilized with 0.25% Triton X-100 in PBS for 15 min. CuAAC reaction samples were treated as described above to obtained 5BrdU-functionalized compounds. To eliminate RNA-structures, cells were treated with 0.1 mg/ml RNase A in PBS at 37°C for 1 h. For staining of 5-BrdU functionalized compounds, cells were treated as previously described: cells were blocked with blocking buffer (5% BSA in PBS) for 2 h at room temperature, then incubated with mouse anti-BrdU (1/250) for 1 h at room temperature in blocking buffer. Slides were then washed three times with PBS and incubated with Alexa Fluor 488 goat anti-mouse (1/500) at room temperature for 40 min in blocking buffer. Slides were washed five times with PBS and mounted.

### *In situ* visualization of G4 structures

For immunostaining, 15 000 A549 cells were seeded and grown in 8-well chamber slides over 24 h. Cells were treated with 5 and 15 μM of PDC-4,3-Alk and PDC-4,3-BrdU. After 16 h of incubation, cells were washed two times with PBS and fixed with 4% PFA in PBS for 15 min. Membrane staining was performed by incubation with wheat germ agglutinin CF®640R WGA 5 μg/ml for 10 min at room temperature. Cells were washed two times with PBS and post-fixation with 4% PFA was carried out during 10 min at room temperature. Cells were washed three times with PBS and permeabilized with 0.25% Triton X-100 in PBS for 15 min. Cells were washed three times with PBS and blocked with 10% goat serum in PBS for 2 h at room temperature. Then, they were incubated with BG4 antibody (20 nM) for 2 h at room temperature in 10% goat serum in PBS. Slides were then washed four times with PBS and incubated with rabbit anti-FLAG (1/800) for 1 h in 10% goat serum in PBS. After washing four times with PBS, slides were incubated with Alexa Fluor 555 goat anti-rabbit (1/1 000) in 10% goat serum in PBS at room temperature for 40 min. Slides were washed five times with PBS and mounted.

#### Statistical information

Results are expressed as mean ± SD (unless stated otherwise). Two-tailed non-paired *t*-tests were performed using GraphPad Prism 7.0. *P*-values are stated in the figure captions. Significance: ns, *P* > 0.05, *P < 0.05, **P < 0.01, ***P < 0.001, ****P < 0.0001. For column scatter plots, error bars are the mean ± SD, central horizontal line is the mean.

### Colocalization experiments

For immunostaining, 15 000 A549 cells were seeded and grown in 8-well chamber slides over 24 h. Cells were treated with 5 and 15 μM of PDC-4,3-Alk and after 16 h of incubation, they were washed two times with PBS and fixed with 4% PFA in PBS for 15 min. Membrane staining was performed as previously described, and after the post-fixation step and washing with PBS, cells were permeabilized with 0.25% Triton X-100 in PBS for 15 min. Cells were washed three times with PBS. Samples containing PDC-4,3-Alk were submitted to CuAAC reaction for 1 h in the dark as previously described. Samples were blocked with 10% goat serum in PBS for 2 h at room temperature. Then, cells were incubated with BG4 antibody (20 nM) for 2 h at room temperature in 10% goat serum in PBS. After 1 h incubation, mouse anti-5BrdU antibody (1/250) was added for the remaining 1 h. Slides were then washed four times with PBS and incubated with rabbit anti-FLAG (1/800) for 1 h in 10% goat serum in PBS. After washing four times with PBS, slides were co-incubated with Alexa Fluor 555 goat anti-rabbit (1/1 000) and Alexa Fluor 488 goat anti-mouse (1/500) in 10% goat serum in PBS at room temperature for 40 min. Slides were washed five times with PBS and mounted.

### Fluorescence microscopy

Images were captured using a Leica 3D SIM (Structured Illumination Microscopy) DM6000 upright wide-field microscope at room temperature with a 63×/1.4–0.6 oil (DF, DIC, POL) objective using a MetaMorph software. DAPI was excited at 405/60 nm, and fluorescence was measured at 470/40 nm. Alexa Fluor 488 was excited at 470/40 nm, and emission was measured at 525/50 nm. Alexa Fluor 555 (Cy3) was excited at 545/30 nm, and emission was measured at 610/75 nm. CF® 640R (Cy5) was excited at 620/60 nm, and emission was measured at 700/75 nm. Images were acquired with a large field, highly sensitive sCMOS camera Hamamatsu Flash 4.0 v2. Leica 3D SIM DM6000 microscope is equipped with a motorized stage in XYZ and galvanometric stage for fast and precise z-stack acquisitions.

## RESULTS AND DISCUSSION

### Synthesis of PDC CuAAC precursors, 5-BrdU click reaction partners and 5-BrdU immuno-tag modified PDCs

**Scheme 1.**
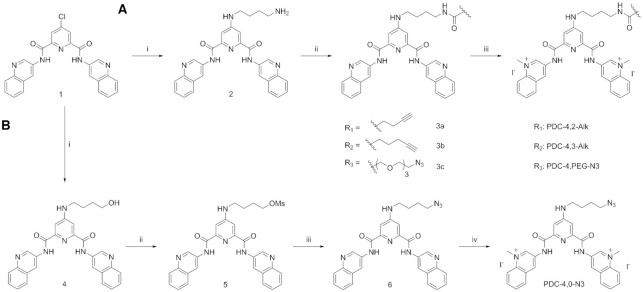
Synthesis of copper-catalyzed alkyne-azide cycloaddition (CuAAC) PDC precursors: PDC-4,2-Alk, PDC-4,3-Alk and PDC-4,PEG-N3. Route A reagents and conditions: (i) 1,4-diaminobutane (100 equiv), NEt_3_ (5 equiv), 95°C, 16 h, 90%; (ii) Carboxylic acid (1.5 equiv), EDCI (1.5 equiv), HOBt (0.15 equiv), DMF, rt, 16 h; R_1_= 4-pentynoic acid, 77%; R_2_ = 5-hexynoic acid, 70%; R_3_ = 11-azido-3,6,9-trioxaundecanoic acid, 61%; (iii) CH_3_I (235 equiv), DMF, 40°C, 16 h, R_1_ = 95%; R_2_ = 93%; R_3_ = 76%. DMF = *N,N*-dimethylformamide; EDCI = 1-Ethyl-3-(3-dimethylaminopropyl)carbodiimide; HOBt = Hydroxybenzotriazole. Route B reagents and conditions: (i) 4-amino-1-butanol (18.7 equiv), NEt_3_ (2.7 equiv), 90°C, 16 h, 66%; (ii) MsCl (4.5 equiv), NEt_3_ (9.0 equiv), DCM, 0°C to rt, 1 h 30 min, 67%; (iii) NaN_3_ (10 equiv), DMF, 110°C, 2 h, 77%; (iv) CH_3_I (250 equiv), DMF, 40°C, 16 h, 87%. MsCl = Methanesulfonyl chloride.

Four different PDC CuAAC precursors have been designed and successfully synthesized. These derivatives bear either an alkyne or an azide functional group on the linker connected to the central pyridine of the PDC core. Four linkers, differing by nature and size, were synthesized to enlarge the chemical diversity of the compounds. All derivatives were prepared starting from a chloro PDC precursor (Scheme 1, compound **1**). PDC-4,2-Alk, PDC-4,3-Alk and PDC-4,PEG-N3 (Figure 1) were prepared by following the same straightforward three-step synthetic pathway (Scheme 1, Route A), developed by slightly modifying the synthesis of PDC-4,2-Alk previously reported (33,42). The aforementioned derivative synthesis was achieved by submitting compound **2** to a peptide coupling reaction in the presence of three commercially available carboxylic acids—4-butynoic acid, 5-hexynoic acid and 11-azido-3,6,9-trioxaundecanoic acid—followed by a methylation reaction. Route A afforded final compounds PDC-4,2-Alk, PDC-4,3-Alk and PDC-4,PEG-N3 with very high yield (Scheme 1). Differently, PDC-4,0-N3 was obtained by following a four-step synthesis (Scheme 1, Route B). At first, compound **1** was submitted to nucleophilic aromatic substitution (S_N_Ar) reaction in the presence of 4-amino-1-butanol yielding compound **4**. Afterward, synthesis of mesylate **5** was achieved by reaction of methanesulfonyl chloride with alcohol **4** at 0°C. Reaction temperature and base molar equivalents play an important role and have to be carefully controlled to avoid elimination reaction. Compound **5** was directly submitted to nucleophilic substitution reaction (S_N_2) in the presence of sodium azide and final methylation reaction afforded PDC-4,0-N3 with high yield. All PDC CuAAC precursors were successfully obtained by mere precipitation and filtration.

**Scheme 2.**
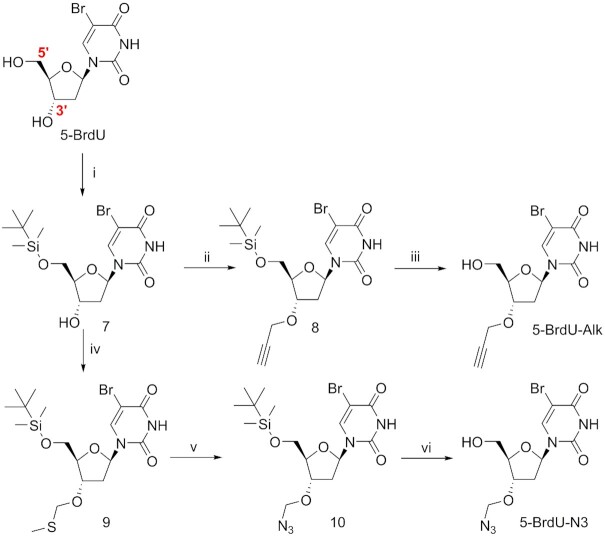
Synthesis of 3′-modified nucleoside 5-BrdU-Alk and 5-BrdU-N3. Positions 5′ and 3′ of the 5-BrdU are shown. (i) TBDMSCl, DMAP, Imidazole, DMF, 0°C – rt, 79%; (ii) NaH, Propargyl bromide, THF, rt, 83%; (iii) TBAF, THF, rt, 75%; (iv) AcOH, Ac_2_O, DMSO, rt, 94%; (v) (a) Br_2_, DCM, 0°C, (b) NaN_3_, DMF, -10°C – rt, 55% over two steps; (vi) TBAF, THF, rt, 62%.

The second synthetic effort was focused on the preparation of the 5-bromo-2′-deoxyuridine derivatives functionalized on the 3′-*O*-position of the deoxyribose ring with an azide (5-BrdU-N3) or an alkyne (5-BrdU-Alk) group by following respectively a four-step and a three-step synthesis (Figure 1 and Scheme 2). Both compounds were prepared starting from 5′-TBDMS-*O*-protected 5-BrdU (**7**). In order to achieve the synthesis of 5-BrdU-Alk, the hydroxyl group in position 3′ was deprotonated by using NaH, and the obtained alcoholate was then reacted with propargyl bromide, obtaining 3′-O-propargyl derivative **8**. A final deprotection step, carried out in the presence of TBAF, yielded final compound 5-BrdU-Alk. To functionalize the 3′-*O*-position of the sugar with an azide and obtain 5-BrdU-N3, we used a modified Pummerer rearrangement (43). Nucleoside **7** was treated with a mixture of dimethyl sulfoxide and acetic anhydride in the presence of acetic acid to obtain the 3′-*O*-methylthiomethyl derivative **9**. The latter was reacted with molecular bromine followed by sodium azide in dry DMF to produce the 3′-*O*-azidomethyl nucleoside **10**. Finally, deprotection of primary alcohol in position 5′ using TBAF led to 5-BrdU-N3. Modified nucleoside 5-BrdU-Alk and 5-BrdU-N3 were isolated by classical column chromatography and obtained with high purity.

5-BrdU PDC conjugates (Figure 1 and Supplementary Scheme S1) were obtained by CuAAC reaction between PDC precursors and the appropriate modified 5-BrdU partner. Several trials were carried out to identify the optimal conditions: we combined different molar equivalents of Cu(I) and sodium ascorbate, performed the reaction with and without copper ligands (TBTA) and employed different mixtures of cosolvents. Finally, we identified the best conditions as follows: 1 mol equiv PDC derivatives, 1.2 mol equiv 5-BrdU partner, 30 mol % CuSO_4_, 1.2 mol equiv of sodium ascorbate and DMF/H_2_O (v/v = 3/1) as mixture of solvent. Catalytic amounts of copper(I) salts, large excess of reducing agent, and inert atmosphere ensure the click reaction to proceed smoothly. Lastly, we excluded copper ligands from the reaction mixture to facilitate final product purification. Residues of copper ions were disposed by gently shaking the reaction mixture with Chelex resin buffered at pH 7. After elimination of the latter, 5-BrdU immuno-tag PDC ligands PDC-4,2-BrdU, PDC-4,3-BrdU, PDC-4,PEG-BrdU and PDC-4,0-BrdU were precipitated and obtained as pure compounds.

PDC CuAAC precursors, 3′-*O*-modified 5-BrdU derivatives and 5-BrdU immuno-tag PDC ligands were isolated with high purity, as evidenced by analytical HPLC, NMR spectra and high-resolution mass spectrometry.

### Evaluation of G4-binding properties of PDC ligands by FRET melting and G4-FID assays

**Figure 2.**
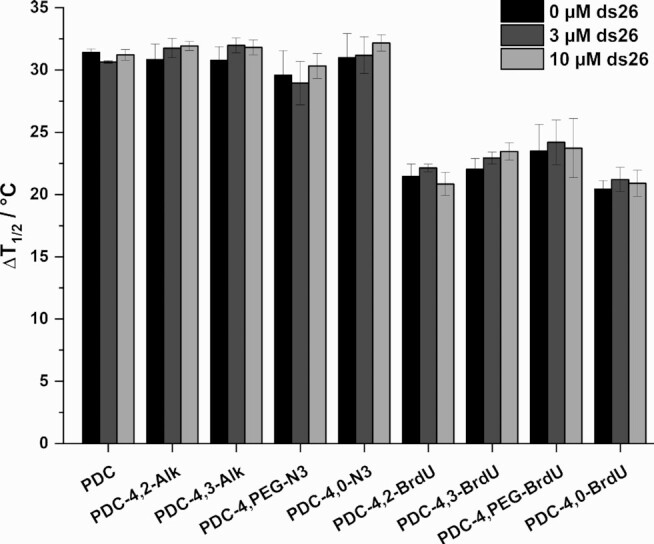
FRET-melting competition experiments with human telomeric sequence F21T in 10 mM Li.Caco buffer (pH 7.2), 90 mM LiCl and 10 mM KCl. Error bars corresponds to SD of three independent experiments.

After successfully synthesizing the G4 ligands, we first examined whether they bind and stabilize G4 structures. PDC derivatives were evaluated for their ability to thermally stabilize sequences prone to form G-quadruplex structures using the Förster resonance energy transfer (FRET) melting assay (44,45). For this aim, we chose as prototype six DNA sequences whose G4-structures are fully characterized: human telomeric sequence (F21T), the proto-oncogene sequences of c-Myc (FcMyc22T, 22G14T-G23T) and c-kit2 (Fkit2T), the human minisatellite repeat native sequence CEB25wt (FCEB25wtT) and modified sequence CEB25L111T (FCEB25L111TT), and a human telomeric sequence variant 21CTA (F21CTAT) (Supplementary Table S1) (46–51). All sequences are doubly labeled with fluorophores to keep track of structure unfolding via FRET. As expected from our previous results, the stabilization effects (Δ*T*_1/2_) observed in the presence of the telomeric sequence are strong in K-rich buffer (Figure 2). Clearly, PDC derivatives can be divided in two distinct groups: PDC CuAAC precursors, which induce stabilization comparable to PDC (360A, progenitor of this family of compounds), give rise to high Δ*T*_1/2_ values ranging from 29 to 31°C and 5-BrdU PDC conjugates that induce stabilization lying in the range slightly below (Δ*T*_1/2_ from 20 to 23°C). As anticipated, the presence of the immuno-tag somewhat affects G4 ligand binding properties by decreasing Δ*T*_1/2_ values of ∼10–15°C, but this effect is minor and the high values observed still denote the strong G4 binding ability of the conjugates in particular considering the condition of the assay (low concentration in G4 DNA). Importantly, the functionalization borne by the ligands does not affect selectivity toward G4 DNA as compared to duplex DNA (Figure 2 and Supplementary Figure S37). A similar trend was observed with the five other G4 structures used in this study (Supplementary Figure S1 and Supplementary Figures S38–S42): the presence of the 5-BrdU tag decreases Δ*T*_1/2_ of about 10°C as compared to the PDC CuAAC precursors.

To further confirm G4 structure selective binding, similar experiments were repeated in the presence of two G4 RNA forming sequences: the telomeric repeat-containing RNA sequence (F21RT) and the human NRAS 5′-UTR sequence (F-NRAS-T) (12,52). The synthesized ligands showed the ability to stabilize selectively the two G4 RNA structures even in the presence of a large excess of human liver tRNA used as competitor (Supplementary Figure S2 and Supplementary Figures S43–S44) thereby indicating a strong preference for the G4 RNA structure. Altogether, these results highlight the remarkable binding selectivity of the synthesized compounds toward both DNA and RNA G4 structures.

To gain additional understanding on the binding affinities of the synthesized G4 ligands, G4-FID assay was performed for all compounds in the presence of the aforementioned unlabeled G4 sequences (Supplementary Table S2) (53). The ability of the ligands to displace Thiazole Orange (TO) from the G4 structures was determined by calculating DC_50_ values (concentration required to displace 50% of TO, Supplementary Table S3, Supplementary Figures S3–S11). PDC CuAAC precursors show DC_50_ values very similar to those obtained with the reference compound PDC (DC_50_ values ranging from 0.20 to 0.45 μM). The values obtained for 5-BrdU immuno-tag modified PDCs are slightly increased, mainly while interacting with 22AG, 21CTA and CEB25wt (DC_50_ range = 0.5–1.4 μM). This behavior can be explained by the interaction of 5-BrdU with the loops. The presence of lateral loops in the 22AG and 21CTA structures as well as the 9 nucleotide long loop of CEB25wt may affect ligand positioning on the external quartets.

In the whole the two biophysical assays revealed fully consistent and highlighted the strong affinity and the remarkably high selectivity of all PDC derivatives toward G4 structures of various topologies.

### Click reactions in the presence of the G4 target

**Figure 3.**
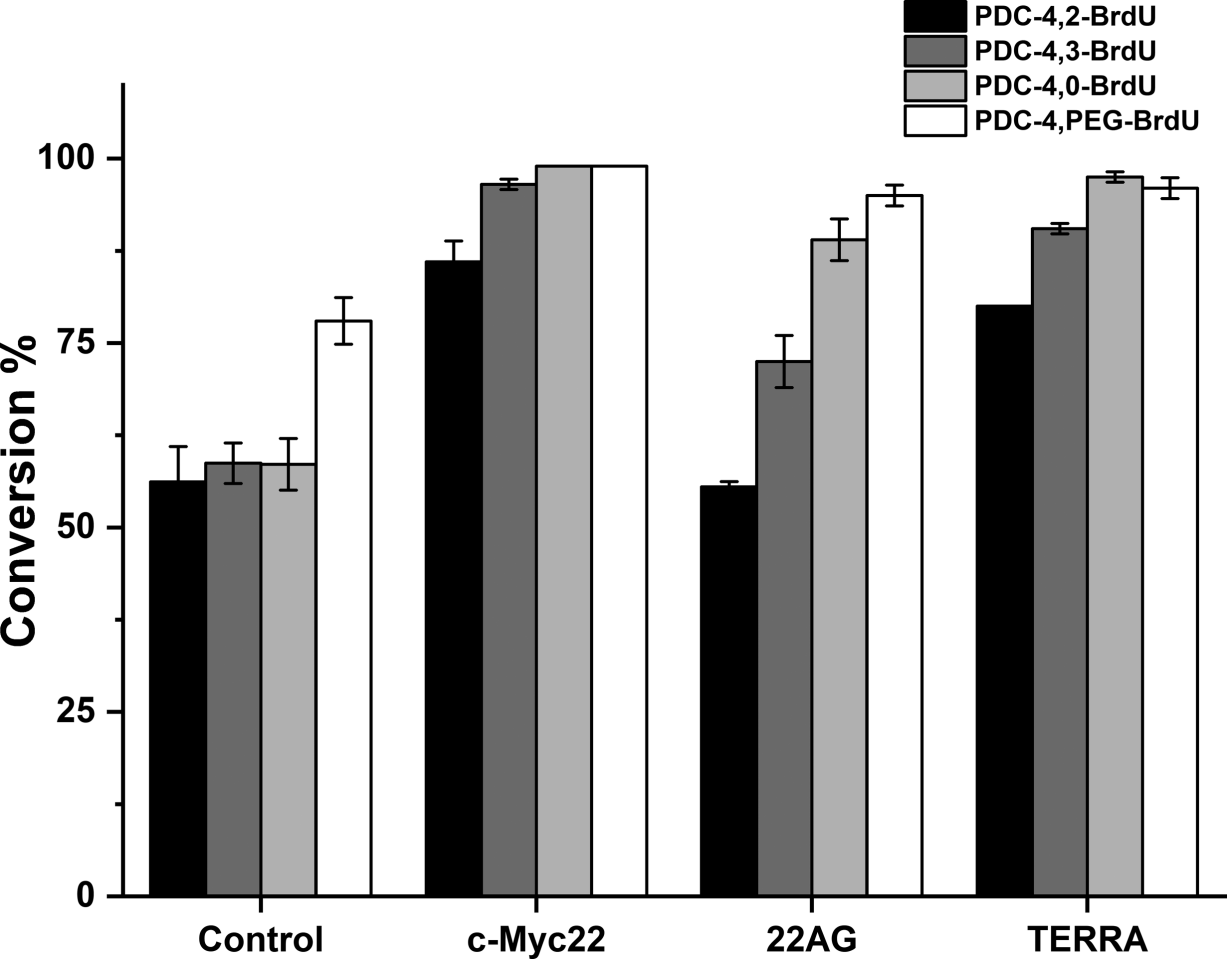
The bar graph represents the relative abundance of 5-BrdU immuno-tag PDCs obtained by CuAAC reaction in the absence (control) or in the presence of G4 structures: c-Myc22, 22AG and TERRA. PDC-4,2-BrdU (black bars), PDC-4,3-BrdU (dark gray bars), PDC-4,0-BrdU (light gray bars) and PDC-4,PEG-BrdU (white bars). Error bars represent the SD calculated from three replicates.

As previously mentioned, the presence of the immuno-tag may affect cellular uptake and cellular subcompartment distribution. Therefore, we explored the reactivity of the four alkyne or azide terminated PDCs in the presence of suitable 5-BrdU click partner post-G4 target-binding. G4 target topology may affect reaction conversions, consequently we investigated CuAAC reactions in the presence of three different G4 conformations: 22AG as example of a hybrid structure, and c-Myc22 and TERRA as examples of parallel DNA and RNA G4 structures (12,46,47). Click reaction conditions were modified compared to those described above to suit the presence of G4. In a typical experiment, PDC CuAAC precursors (25 μM) and G4 (25 μM) were mixed together in cacodylate buffer. Separately, a second solution containing CuSO_4_ (500 μM), THPTA (2.5 mM) and sodium ascorbate (2 mM) was prepared. The two solutions were then combined and 5-BrdU click partner (50 μM) was added. A control reaction in the absence of G4 target was carried out in the same conditions to analyze reaction conversions at a defined reaction time. We compared the reactivity after 1 h of incubation, time at which, in the absence of G4 target, the conversion is about 50% for most PDC derivatives (reaction kinetics followed for PDC-4,2-Alk, Supplementary Figure S12). Afterward, DNA was eliminated by solid phase extraction and the mixture was monitored by analytical HPLC (Figure 3 and Supplementary Figures S13–S15). Remarkably the presence of the G4 target significantly increased reaction conversion in all cases (up to 80–100%), thereby confirming the strong G4-templating effect as previously reported (54). Although c-Myc22 and TERRA induced similar enrichment effects, the telomeric sequence 22AG is a bit less efficient with PDC-4,2-Alk and PDC-4,3-Alk (0–10% increase, respectively). Again the presence of lateral loops may either hinder spatial proximity of the two reactive moieties or have a negative impact on alkyne-copper(I) complex generation (33). Alternatively the existence of several G4 conformations in equilibrium can also be responsible for this result (55). Globally the clear beneficial effect produced by the G4 structures on the click reaction prompted us to pursue immuno-tag functionalization to achieve our new immunofluorescence approach.

### G4-ligand guided immunofluorescence labeling using an adapted enzyme-linked immunosorbent assay (ELISA)

**Figure 4.**
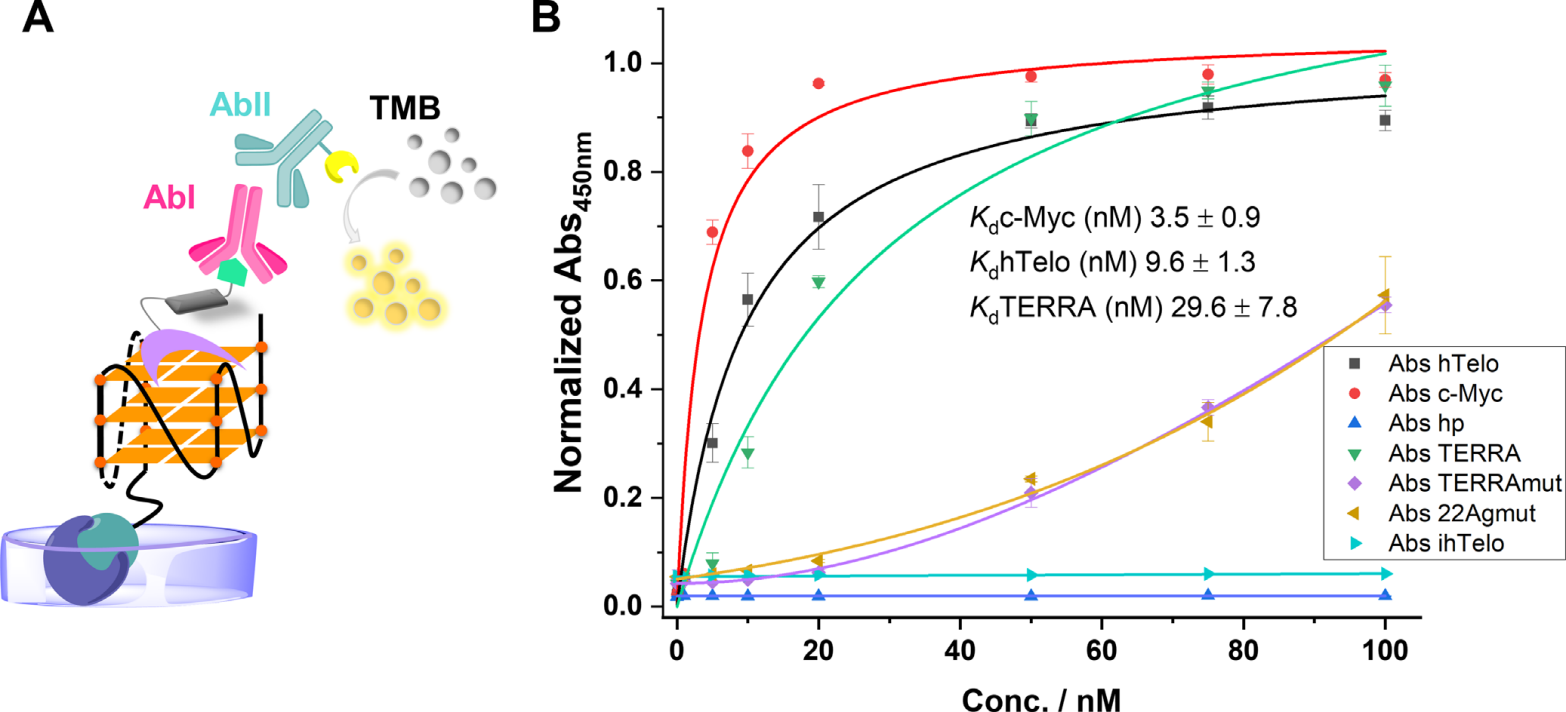
(**A**) Schematic representation of 5-BrdU PDC conjugate recognition by antibodies and chromogenic detection *in vitro* (ELISA, TMB = 3,3′,5,5′-Tetramethylbenzidine). (**B**) Binding curves determined by an adapted ELISA assay for hTelo, c-Myc, and TERRA as examples of G4s, a hairpin (hp) as example of a duplex, 22Agmut and TERRAmut as examples of G-rich sequences not able to fold in a G4 structure, and ihTelo as example of a C-rich sequence (normalized Abs_450nm_ of TMB versus oligonucleotide concentration) and dissociation constants (*K*_d_) obtained from curve fitting (1:1) in the presence of G4 sequences. Error bars represent the SD calculated from three replicates.

The next step was to verify whether specific antibodies recognized 5-BrdU modified PDC bound to G4 structures. Hence in the aim of validating our strategy we devised a solid-phase type enzyme immunoassay, to assess the detection of the complexes between 5-BrdU immuno-tag PDC ligands and G4 targets. Briefly, a biotinylated G4 sequence was immobilized on a surface, and after incubation with the immuno-tag modified ligands, anti 5-BrdU antibody (AbI) was added. AbI was then detected by HRP-conjugated secondary antibodies (AbII). Finally, an enzymatic substrate was added to allow chromogenic detection of AbII thus revealing the 5-BrdU-functionalized ligand associated to G4 (Figure 4A).

Since all 5-BrdU immuno-tag PDCs showed similar biophysical properties toward the different prototype G4 structures, we selected PDC-4,3-BrdU as a reference compound and as biotinylated sequences to perform the assay: hTelo, c-Myc and TERRA as examples of G4s, a hairpin (hp) as example of a duplex, 22Agmut and TERRAmut as examples of G-rich sequences not able to fold in a G4 structure and ihTelo as example of a C-rich sequence. Remarkably PDC-4,3-BrdU was successfully detected when bound to the three G4 structures in a clear G4-concentration dependent manner (Figure 4B). The ligand was not detected in the presence of the hairpin (hp) and the C-rich (ihTelo) sequence. Differently, a much lower chromogenic signal was generated at high DNA concentration in the presence of the mutated G-rich sequences, suggesting a low binding affinity produced by unspecific binding. Importantly, this result provides evidence that the antibodies can efficiently detect only G4-bound ligand species.

Using this adapted ELISA assay, we were able to determine the binding constants (*K*_d_) of PDC-4,3-BrdU for hTelo, c-Myc and TERRA: *K*_d_[hTelo] = 9.6 ± 1.3 nM, *K*_d_[c-Myc] = 3.5 ± 0.9 nM, and *K*_d_[TERRA] = 29.6 ± 7.8 nM respectively. All *K*_d_ values are lying in the low nanomolar range confirming the exceptionally high affinity of this ligand toward G4 structures. To be highlighted, this affinity is close to that observed for the BG4 antibody. Importantly this biochemical assay provided the undisputable proof of concept that 5-BrdU immuno-tag PDCs can be recognized and visualized by antibodies, when bound to G4 targets, thereby fully validating our G4-ligand guided immunostaining strategy.

### G4-ligand guided immunofluorescence staining (G4-GIS) in cells

**Figure 5.**
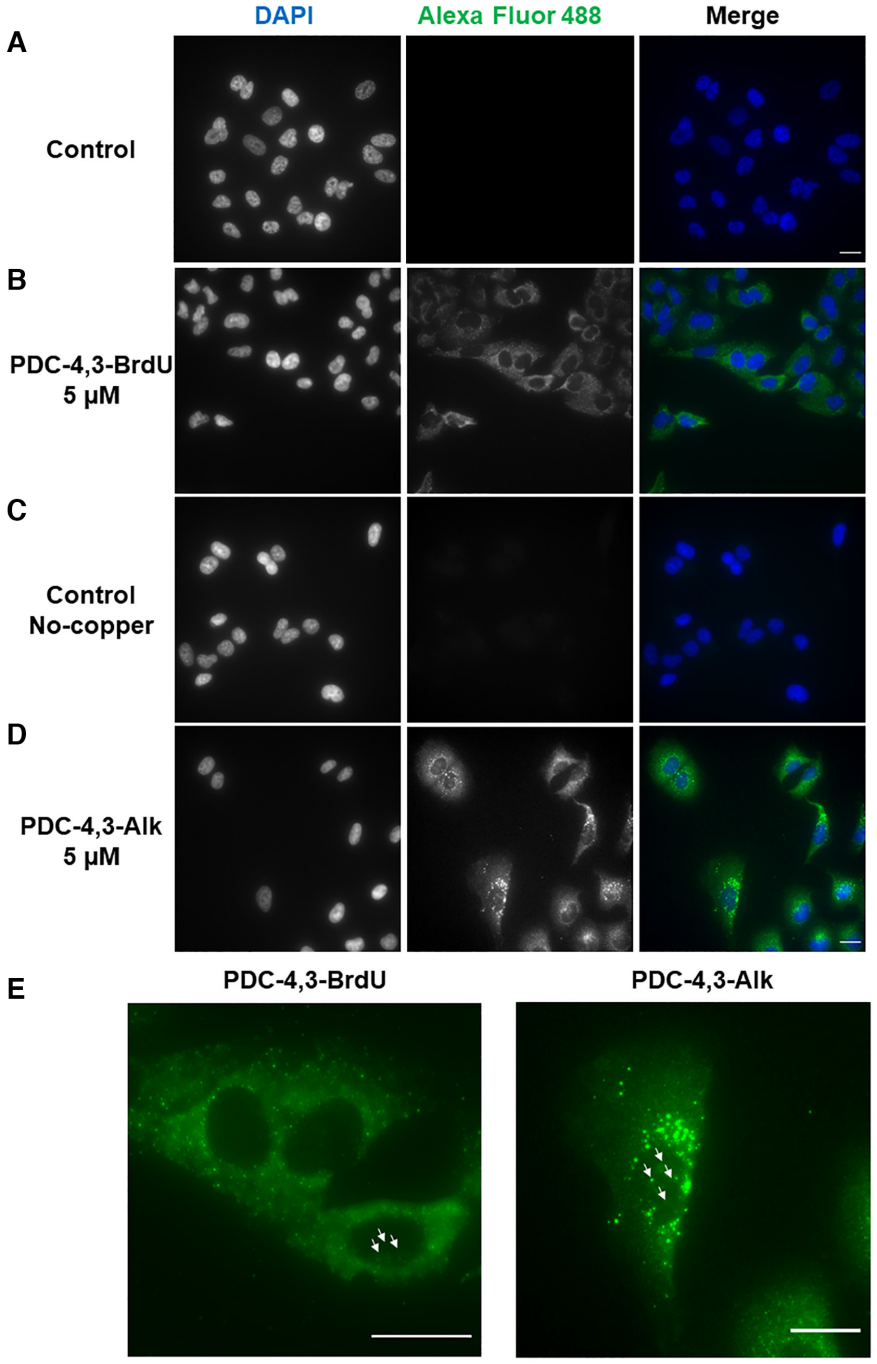
G4 ligand immunofluorescence staining in A549 cells and nucleus visualized with DAPI. Fluorescence wide-field microscopy images of A549 cells (**A**) untreated, and after 16 h incubation with (**B**) 5 μM PDC-4,3-BrdU, (**C**) 5 μM PDC-4,3-Alk control, obtained by treatment with 5-BrdU-N3 in the absence of copper, (**D**) 5 μM PDC-4,3-Alk copper-based click reaction in the presence of 50 μM 5-BrdU-N3, 100 μM CuSO_4_·5H_2_O, 500 μM THPTA and 5 mM NaAsc, and (**E**) magnification Alexa Fluor 488 images. Arrows indicate nuclear foci. Images are represented as a Z-projection; scale bar: 20 μm.

Strongly encouraged by these results, we next examined the feasibility to detect G4 ligand distribution in cells by immunofluorescence. A549 cell line was chosen for this study. Before proceeding, ligand cytotoxicity was assessed at 72 h to identify the best concentration to perform the imaging experiments. All ligands exhibited moderate toxicity with IC_50_ values ranging from 16 μM to >100 μM (Supplementary Table S4), with the two PEG-containing derivatives showing very low toxicity (IC_50_ > 100 μM). All PDC derivatives were incubated with A549 cells at three subtoxic concentrations (5, 10 and 15 μM) over a period of 16 h to verify the effect produced by ligand accumulation on the detection method. The fluorescence signal of the cells was monitored by using an upright wide-field microscope. Concisely, after ligand treatment, cells were fixed with paraformaldehyde and permeabilized to allow antibody penetration, then immuno-tag PDC treated cells were directly incubated with anti 5-BrdU primary antibody and Alexa Fluor 488 labeled secondary antibody. Differently, PDC CuAAC precursor treated cells were first submitted to click reaction for 1 h in the dark, optimal reaction conditions were identified as followed: 50 μM of 5-BrdU-N3 or 5-BrdU-Alk partner, 100 μM CuSO_4_·5H_2_O, 500 μM THPTA and 5 mM sodium ascorbate. Afterward, cells were treated with antibodies as described above. Interestingly cells incubated with 5-BrdU tagged PDC conjugates or PDC CuAAC precursors assembled *in situ* with BrdU partners displayed a more or less diffuse fluorescent signal in the cytoplasm together with fluorescent spots (foci) of variable intensity within the nucleus (Figure 5 and Supplementary Figures S17–S24). These results first confirm the feasibility of the G4-GIS method in cells and suggest that at the chosen incubation time, although the compounds mainly accumulated in the cytoplasm they show specific nuclear localisation as pointed out by the generation of foci. To verify the specificity of the fluorescent signal, negative controls were performed using untreated cells and cells incubated with the nucleoside derivatives 5-BrdU-N3 or 5-BrdU-Alk alone. As expected, A549 cells submitted to these conditions did not show any fluorescent signal (Figure 5A and Supplementary Figure S16), thereby confirming the specificity of the ligand guided immunostaining i.e. the antibodies recognize exclusively the 5-BrdU-tag when linked to the G4-ligand PDC moiety. The first control guaranteed the specificity of the antibodies (Figure 5A); the second confirmed that 5-BrdU immuno-tag compound distribution was not driven by the functionalization (Supplementary Figure S16). However, the staining pattern appeared strongly dependent on the concentration and on the chain length linking the two functional moieties. In most cases the blurry background likely due to non-specific binding/distribution was enhanced by increasing the concentration (Supplementary Figures S17–S24). Most strikingly the PDC-4,3-Alk derivative clearly distinguishes itself (Figure 5D,E and Supplementary Figure S22) giving a very specific staining pattern characterized by the generation of bright and well-defined fluorescent foci preferentially located in the perinuclear space. Remarkably the staining is displaying a very low background that furthermore is not amplified at higher concentration (Figure 5D,E and Supplementary Figure S22). Experiments performed in A2780 cells showed a very similar distribution, supporting that the specific localisation of PDC-4,3-Alk is not cell line dependent (Supplementary Figure S25). Interestingly cells treated with PDC-4,3-Alk and 5-BrdU-N3 but without copper addition did not display fluorescent signal (Figure 5C) thus revealing the efficacy of the click reaction and indicating again the exclusive detection of the 5-BrdU attached to the PDC scaffold. If we analyze more in depth the chain length effect, it appears that the short (4,0) and long chain (4,PEG) compounds have lower performances indicating that the 4,3 chain represents the ideal tradeoff between cellular uptake, intracellular distribution, G4-target binding and efficacy of the click reaction. Indeed, it is well known that subtle structural variations may impact significantly the cellular distribution of compounds (56). Finally, when comparing the images provided by the PDC-4,3-BrdU conjugate with that of its clickable counterpart it can be concluded that globally the *in situ* functionalization gives better results than the *ex situ* functionalization (Figure 5E). Lastly in the aim of comparing the immunostaining 5-BrdU driven method with classical labeling using a fluorescent tag, we performed in cell click labeling of PDC-4,3-Alk with the cyanine dye Cy5 (32,33). This treatment provided a less defined labeling of the cytoplasm with very poor resolution (Supplementary Figure S26), although the perinuclear accumulation is still visible. This result evidences the clear advantage of the immunostaining guided method as compared to direct labeling with a fluorophore and underscores its efficiency to detect G4 ligand distribution within cellular compartments with higher spatial precision.

**Figure 6.**
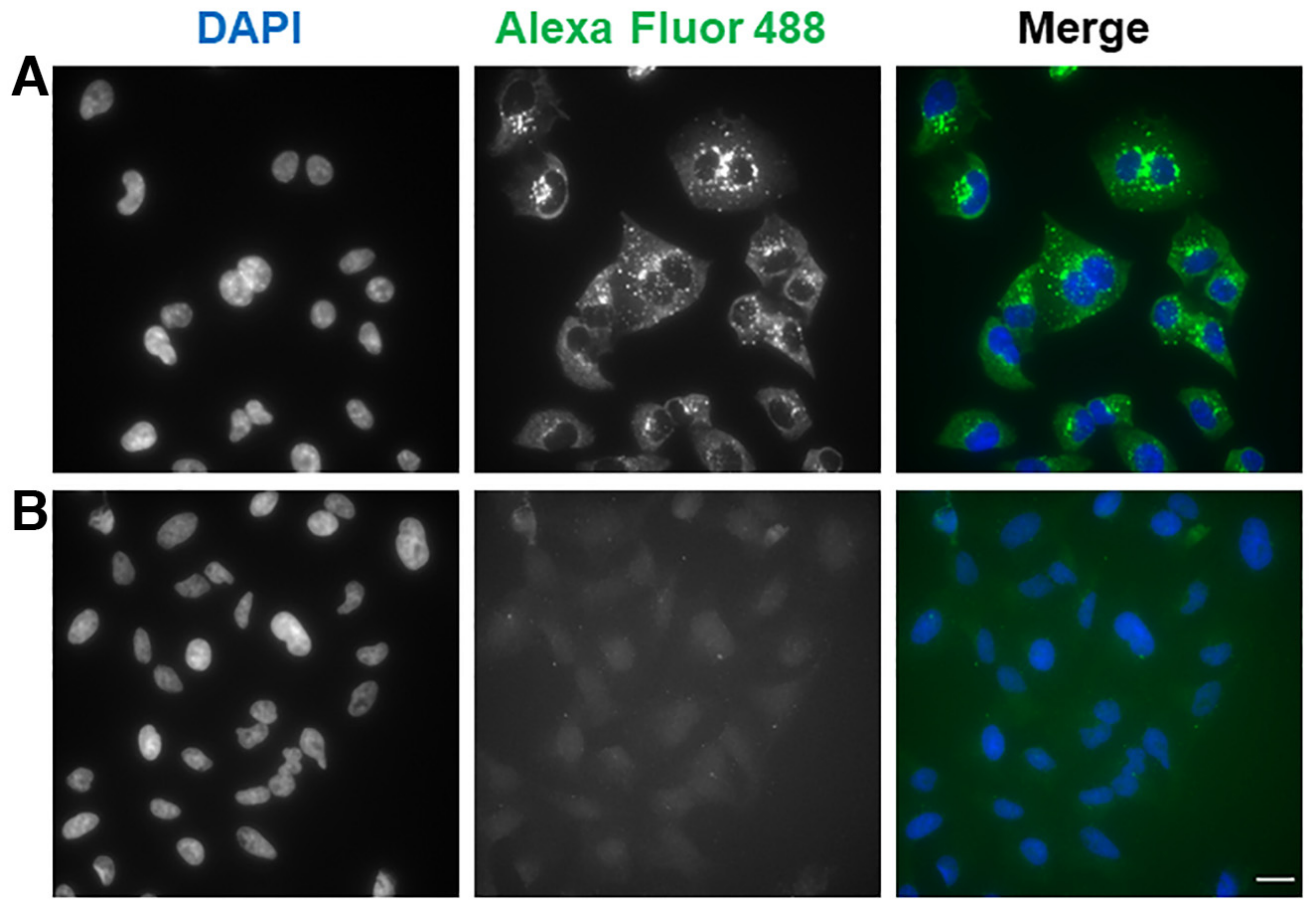
Fluorescence wide-field microscopy images of A549 cells after 16 h incubation with 5 μM PDC-4,3-Alk and (**A**) without RNase A treatment and (**B**) 0.1 mg/ml RNase A treatment at 37°C for 1 h. DAPI and Alexa Fluor 488 channels are shown separately and then merged in false colours. Images are represented as a Z-projection; scale bar: 20 μm.

The cytoplasmic accumulation of the G4 ligands and in particular that of PDC-4,3-Alk prompted us to investigate if the fluorescent signal was produced by RNA binding. In addition, biophysical assays have shown the ability of all synthesized ligands to bind and stabilize G4 RNA with high affinity and selectivity with regard to other structured RNAs (tRNA) (Supplementary Figure S2). To confirm preferential G4 RNA binding in cells, the labeling experiment using PDC-4,3-Alk was reproduced at different incubation times and followed by RNase A treatment. At short incubation times (4 and 8 h) PDC-4,3-Alk is located in the cytoplasm and clearly accumulates in the nucleoli, and only after 16 h of incubation it is possible to observe the generation of well-defined perinuclear foci (Supplementary Figure S27). Furthermore, RNase treatment induced substantial reduction of the cytoplasmic fluorescence signal (Figure 6) as well the same was observed with all ligands (Supplementary Figures S28–S35). The accumulation of the molecule in the nucleoli and in the cytoplasm, together with the substantial reduction of the cytoplasmic staining after RNase treatment strongly indicate that all ligands are binding RNA in the cytoplasm whereas only one (PDC-4,3-Alk) is able to provide a foci-based labeling pattern attributable to specific G4 RNA binding. This result, together with the perinuclear localization of the fluorescent foci, strongly suggests preferential labeling of RNA bound to the endoplasmic reticulum. This is consistent with the high content of mRNA and rRNA in this organelle and with the recent evidence of G4 formation in ribosomal RNA (57). Of note, removing the strong labeling of the cytoplasmic RNA permitted to highlight the presence of fluorescent foci within the nucleus, suggesting G4 DNA targeting by the ligand, and evidencing the sensitivity of the method that allows detection of dim signals (Figure 6 and Supplementary Figures S28–S35).

Taken together, these experiments demonstrate that 5-BrdU immuno-tag modified G4 ligands can be efficiently detected in cells by the G4-GIS methodology and led to the selection of PDC-4,3-Alk as the best candidate for the new labeling approach. Of crucial importance, this method allowed identifying RNA and most likely G4 RNA as the main target of PDC-4,3-Alk.

### Effects produced by G4 ligand treatment on BG4 foci distribution

**Figure 7.**
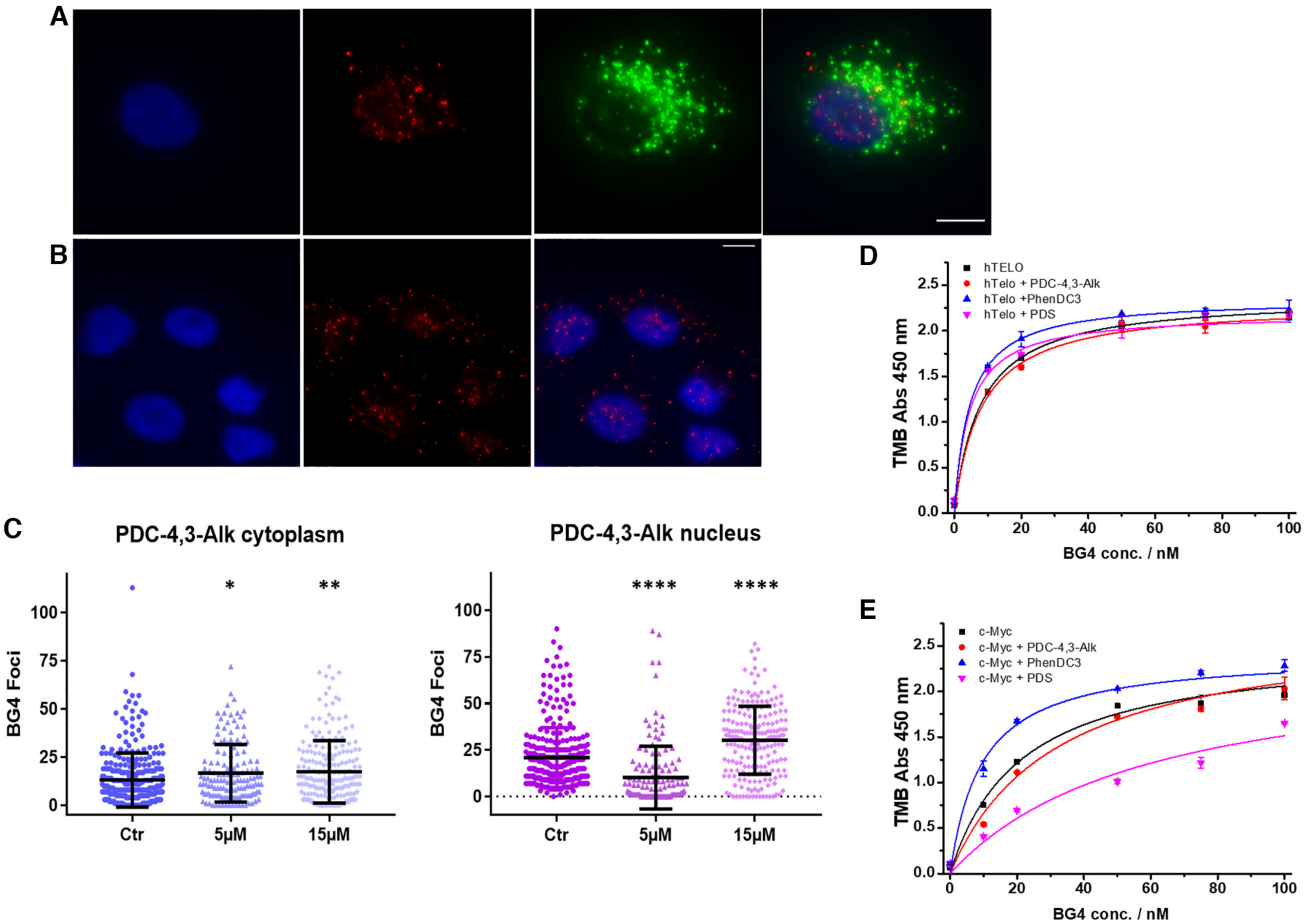
Colocalization experiments between anti 5-BrdU antibody and BG4 antibody: fluorescence wide-field microscopy images of A549 cells (**A**) after 16 h incubation with 15 μM PDC-4,3-Alk and submission to a CuAAC reaction and (**B**) untreated stained with BG4 and anti 5-BrdU antibodies; scale bar: 10 μm. Nucleus are stained with DAPI (blue); Functionalized PDC-4,3-Alk is visualized with Alexa Fluor 488 conjugated secondary antibody (green); BG4 foci are visualized with Alexa Fluor 555 conjugated antibody (red). (**C**) Column scatter plots representing the number of cytoplasmic and nuclear BG4 foci detected in control A549 cells (Ctr) or treated with PDC-4,3-Alk. Mean values are indicated for each group. *P* values were calculated toward the correspondent control: *P < 0.05, **P < 0.01, ****P < 0.0001, *t-*Student test. Binding curves determined by ELISA assay (**D**) on hTelo and (**E**) c-Myc without and with ligand. Error bars represent the SD calculated from three replicates.

To investigate further the ligand distribution specificity with regard to G4 structures, immunofluorescence experiments in the presence of the G4 specific antibody BG4 were performed (13,58). At first, by taking advantage of the G4-GIS approach described above we carried out colocalization experiments between BG4 and anti 5-BrdU antibodies. A549 cells were treated with PDC-4,3-Alk as described above (Figure 5) and co-immunostained with BG4 and anti 5-BrdU antibodies, respectively. To identify distinctly nucleus and cytoplasm, and quantify separately BG4 foci within the two compartments, we stained the first one with DAPI and the cell membrane with wheat germ agglutinin (WGA). BG4 foci were found both in the cytoplasm and in the nucleus (Figure 7A,B), but no significant colocalization was observed with anti 5-BrdU antibodies in these conditions (Figure 7A). Considering that the two antibodies are directed toward the same G4 target (G4 structure for BG4 and G4/ BrdU-ligand complexes for anti 5-BrdU Ab), they can exclude each other either by preventing accessibility of the primary antibodies or by hampering the generation of the large-sized supramolecular assemblies resulting from secondary antibodies recognition or even tertiary antibodies as required for BG4 staining. In addition, the presence of the G4 ligand may affect the initial G4 epitope recognition by BG4 as already reported (59,60). To explore further the interplay between ligand and BG4, we studied BG4 distribution after PDC-4,3-Alk treatment. Compound treatment produced a change in number of BG4 foci which was statistically significant on the analyzed population of cells (up to 400 cells) (Figure 7C and Supplementary Figure S36 [PDC-4,3-BrdU]). Indeed, a 1.3-fold increase of the number of BG4 foci in the cytoplasm was observed both at 5 and 15 μM of ligand. An even more relevant variation in BG4 foci was observed in the nucleus with a 1.4-fold increase of the total number of foci at 15 μM ligand concentration. This trend is consistent with literature data and is commonly attributed to promotion and stabilization of G4 structures by G4 ligands. Surprisingly, at the lowest concentration (5 μM) the number of BG4 foci in nucleus was significantly decreased of about 0.5-fold as compared to the untreated cells. This unexpected but highly reproducible result indicates that the interplay between BG4 and G4 ligand for binding can be difficult to predict as it might be strongly correlated to cellular status as well as G4 dynamics and accessibility.

Furthermore, these observations raised questions about the affinity of BG4 antibody toward a G4 structure already bound to a G4 ligand. To address this point, we assessed BG4 binding properties toward hTelo and c-Myc, already in complex with G4 ligands by ELISA. To this end, PDC-4,3-Alk as well as two other benchmark G4 ligands, namely PhenDC3 and PDS, were used. This assay showed that our expressed BG4 antibody displayed slightly lower affinities as compared to those reported in literature toward the two selected G4 structures: *K*_d_[hTelo] = 7.8 ± 1.5 nM and *K*_d_[c-Myc] = 21.1 ± 4.6 nM (*K*_d_[hTelo] = 1.6 ± 0.1 nM and *K*_d_[c-Myc] = 1.5 ± 0.2 nM) (13). BG4 binding affinity on hTelo was negligibly affected by PDC-4,3-Alk (*K*_d_[hTelo] = 8.0 ± 1.9 nM), and on the contrary, was slightly improved by the presence of the two benchmark G4 ligands (*K*_d_[hTelo] = 4.6 ± 0.8 nM and 4.4 ± 1.2 nM for PhenDC3 and PDS, respectively) (Figure 7D). Differently, PDC-4,3-Alk and PDS negatively affected BG4 binding on c-Myc (*K*_d_[c-Myc] = 31.7 ± 7.8 nM and 59.8 ± 28.1 nM, respectively), and PhenDC3 remarkably improved the binding (*K*_d_[c-Myc] = 9.5 ± 3.2 nM) (Figure 7E).

These *in vitro* data suggest that the nature of the G4 ligand used and the topology of the G4 structure may influence BG4 recognition in cells. We hypothesize that at lower concentration PDC-4,3-Alk targets G4 structures for which it has the strongest affinity thus competing with BG4 binding, as suggested by the *K*_d_ values determined for its conjugate PDC-4,3-BrdU. Differently, at higher concentration PDC derivatives can bind, a larger panel of G4 structures of high and low affinity and also promote their formation, altogether increasing BG4 foci.

## CONCLUSION

In this work, we have developed a new approach for guiding detection of G4 ligands in complex with G4 structures in cells. The new G4-ligand guided immunofluorescence staining (G4-GIS) approach is based on the specific recognition of 5-BrdU immuno-tag modified-G4 ligands by a commercially available primary antibody. After validation of G4 ligand binding properties toward G4 structures by biophysical assays and confirmation of *in situ* functionalization by CuAAC reaction, *in vitro* experiments have demonstrated that 5-BrdU functionalized ligands can be recognized and visualized by specific antibodies, once bound to the G4 target. These recognition properties were further verified in A549 cells, as evidenced by the generation of a fluorescent signal detected by microscopy, validating the immunofluorescence methodology. Notably, in our experimental conditions, immunofluorescent experiments showed preferential ligand cytoplasmic accumulation in defined regions (foci) with low ligand nuclear localisation and RNase A treatment highlighted RNA as the main target of the synthesized ligands. The fluorescent signal amplification guaranteed by secondary antibodies allowed to improve spatial precision and sensitivity and clearly detect perinuclear accumulation of PDC-4,3-Alk with formation of fluorescent foci. Additional immunofluorescence experiments, aiming to the detection of BG4 foci, put in evidence that the presence of a G4 ligand bound to a specific G4 topology may affect BG4 recognition, and as a consequence, BG4 foci detection. Furthermore, G4-GIS methodology showed the impossibility to perform colocalization experiments between BG4 and anti 5-BrdU antibody emphasizing on the strong dependency between secondary antibody binding and spatial proximity of the two primary antibody targets. In a broader perspective this study provides the research basis for the application of immunofluorescence methodology and associated signal amplification to visualize immuno-tag modified-compounds bound to their cellular target and improve the spatial detection of small molecules functionalized with fluorescent probes.

## Supplementary Material

gkab1166_Supplemental_Files

## Notes

Present address: Eugénie Laigre, Département de Chimie Moléculaire, UMR CNRS 5250, Université Grenoble Alpes, 38058 Grenoble, France.

Present address: Anne Cucchiarini, Laboratoire d’Optique et Biosciences, Ecole Polytechnique, CNRS, Inserm, Institut Polytechnique de Paris, 91128 Palaiseau, France.
