# Supplementary material for: BrdU immuno-tagged G-quadruplex ligands: a new ligand-guided immunofluorescence approach for tracking G-quadruplexes in cells
Source: Nucleic Acids Res. 2021 Dec 7;49(22):12644–60. doi: 10.1093/nar/gkab1166 (PMC8682774; doi:10.1093/nar/gkab1166)
Supplement: gkab1166_Supplemental_Files [file gkab1166_supplemental_files.zip › Masson et al. NAR_2021_Supplementary Information_2_Revised.pdf]

Supplementary Information

BrdU immuno-tagged G-quadruplex ligands: a new ligand-guided immunofluorescence approach for tracking G-quadruplexes in cells

Thibaut Masson[1,2], Corinne Landras Guetta[1,2], Eugénie Laigre[1,2], Anne Cucchiarini[1,2], Patricia Duchambon[1,2], Marie-Paule Teulade-Fichou[1,2,*], and Daniela Verga[1,2,*]

[1] CNRS UMR9187, INSERM U1196, Institut Curie, PSL Research University, F-91405 Orsay, France
[2] CNRS UMR9187, INSERM U1196, Université Paris-Saclay, F-91405 Orsay, France

Present Address: Eugénie Laigre, CNRS, Department of Molecular Chemistry, University Grenoble Alpes, UMR 5250, 38000 Grenoble, France; Anne Cucchiarini, Laboratoire d'Optique et Biosciences, Ecole Polytechnique, CNRS, Inserm, Institut Polytechnique de Paris, 91128 Palaiseau, France.

Table of Contents

**^{1}H NMR and APT ^{13}C NMR spectra PDC CuAAC precursors and 5-BrdU partners**	**3-18**
**^{1}H NMR spectra 5-BrdU immuno-tag modified PDCs**	**19-20**
**Analytical HPLC chromatograms: PDC-4,2-Alk, PDC-4,3-Alk, PDC-4,PEG-N3, PDC-4,0-N3**	**21-22**
**Analytical HPLC chromatograms: PDC-4,2-BrdU, PDC-4,3- BrdU, PDC-4,PEG-BrdU, PDC-4,0- BrdU**	**23-24**
**FRET-melting curves of DNA G-quadruplex sequences**	**25-48**
**Figure S37**	**25-28**
**Figure S38**	**29-32**
**Figure S39**	**33-36**
**Figure S40**	**37-40**
**Figure S41**	**41-44**
**Figure S42**	**45-48**
**FRET-melting curves of RNA G-quadruplex sequences**	**49-56**
**Figure S43**	**49-52**
**Figure S44**	**53-56**

Compound 3a – ^{1}H NMR

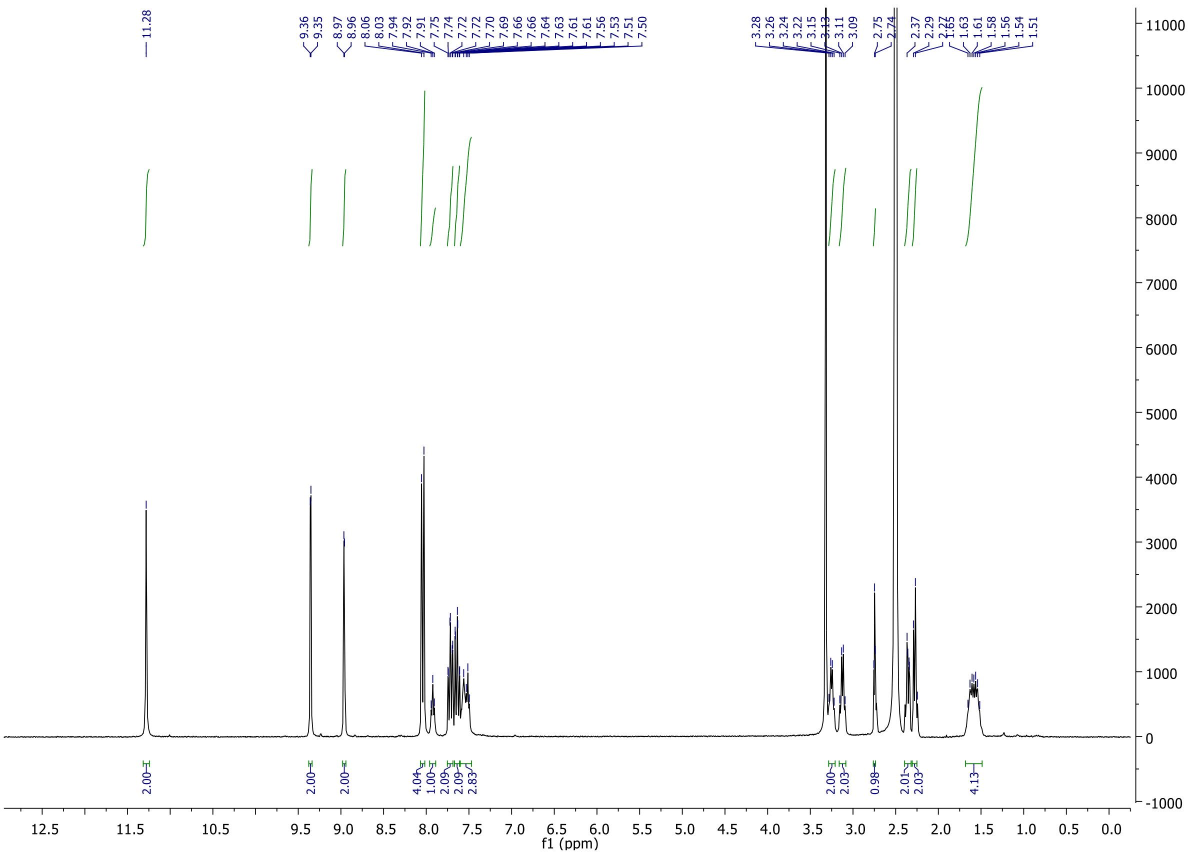

Compound 3a – APT ^{13}C NMR

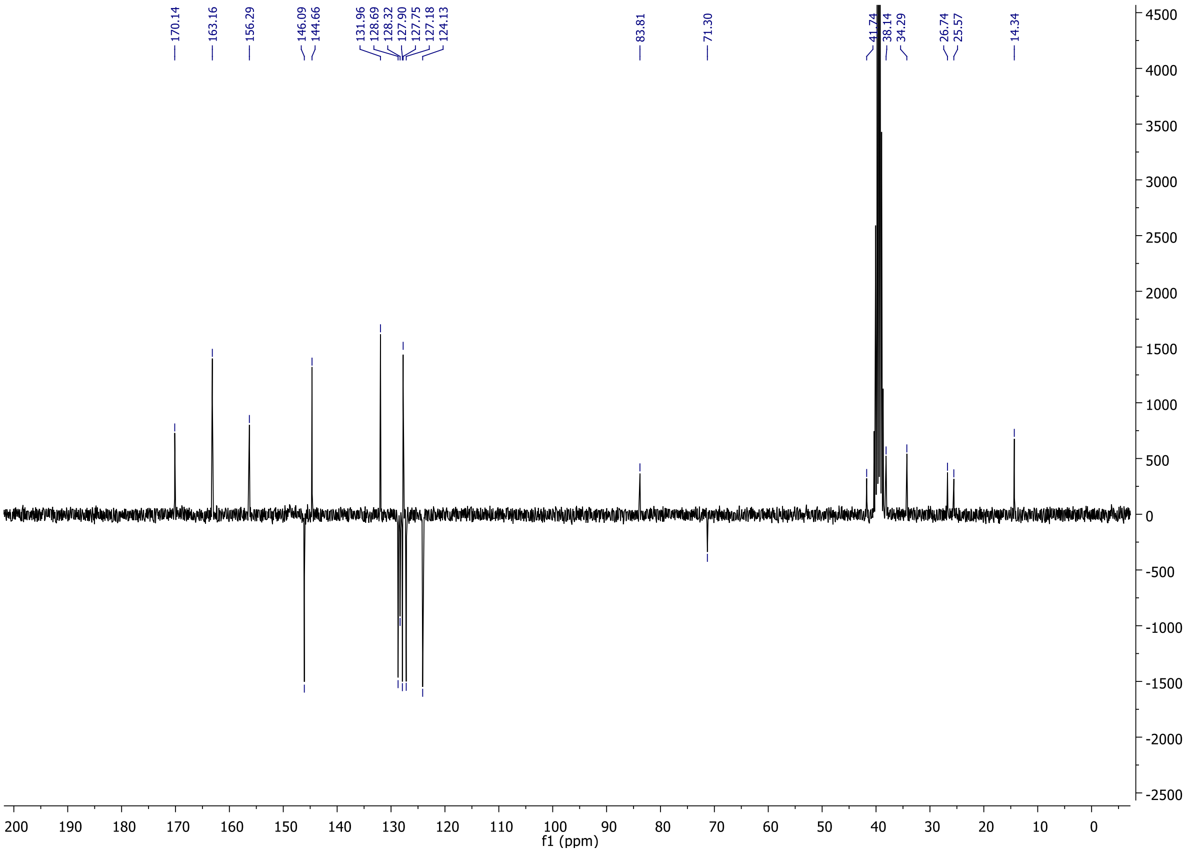

Compound 3b - ^{1}H NMR

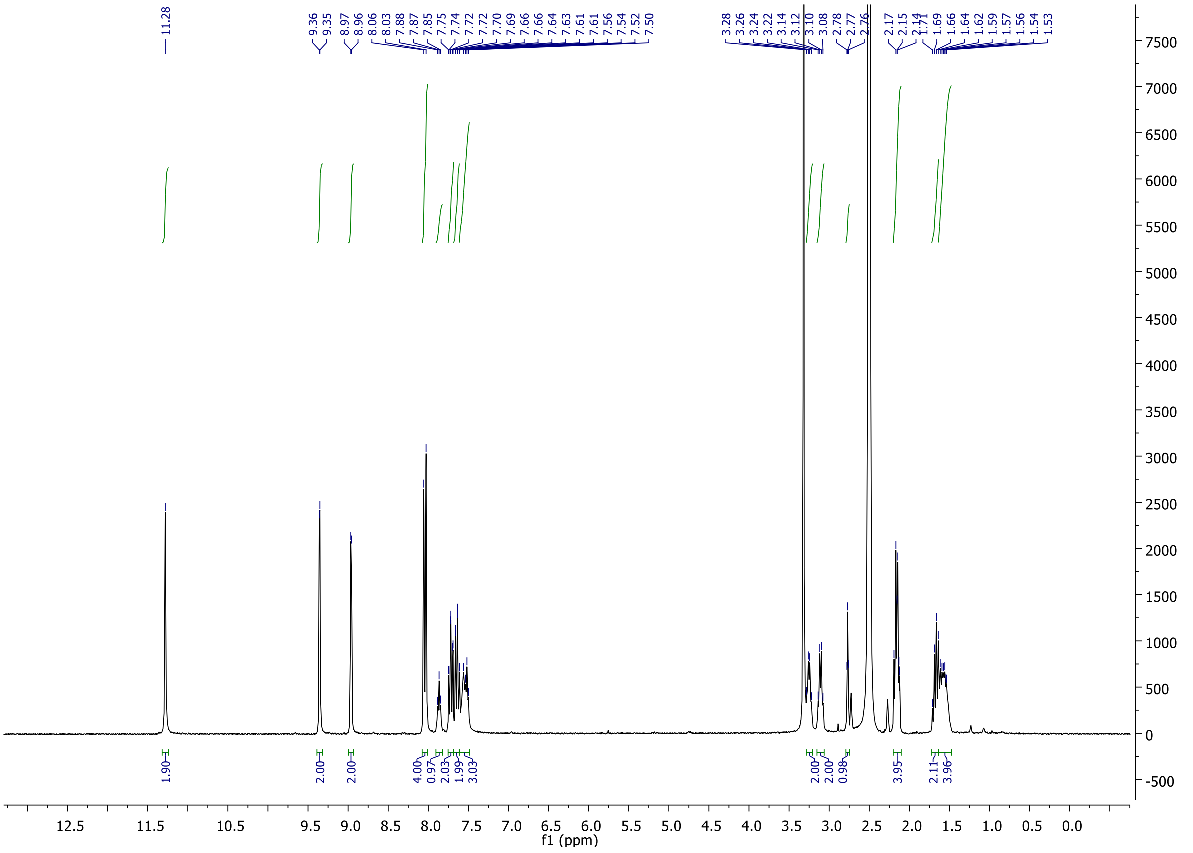

Compound 3b – APT ^{13}C NMR

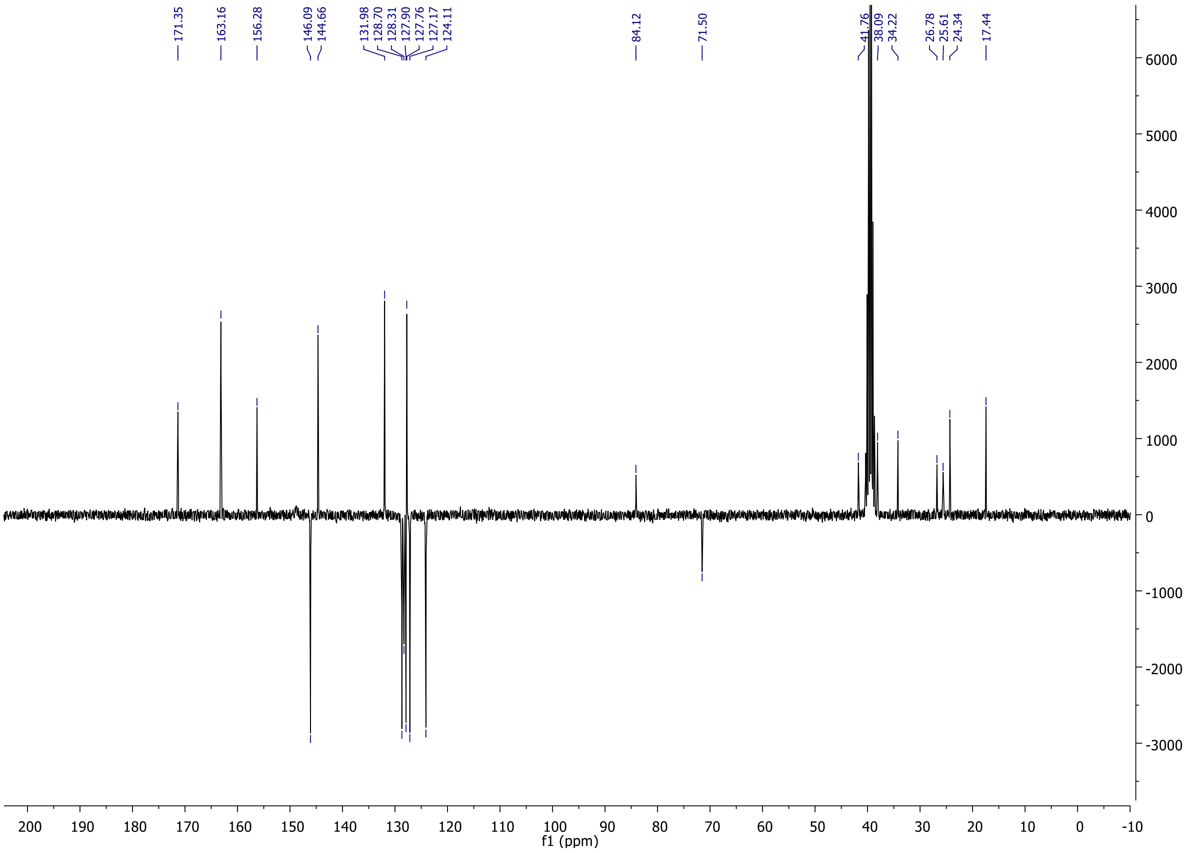

Compound 3c - ^{1}H NMR

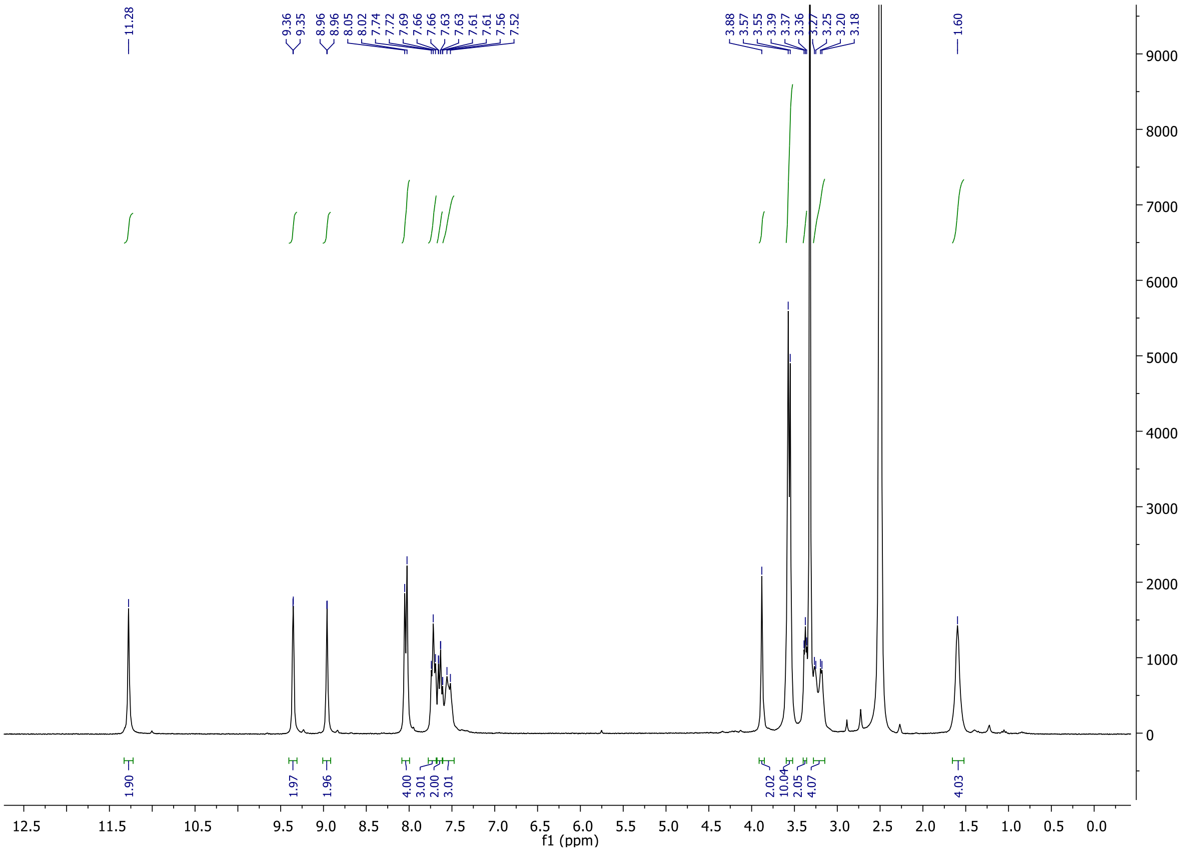

Compound 3c – APT ^{13}C NMR

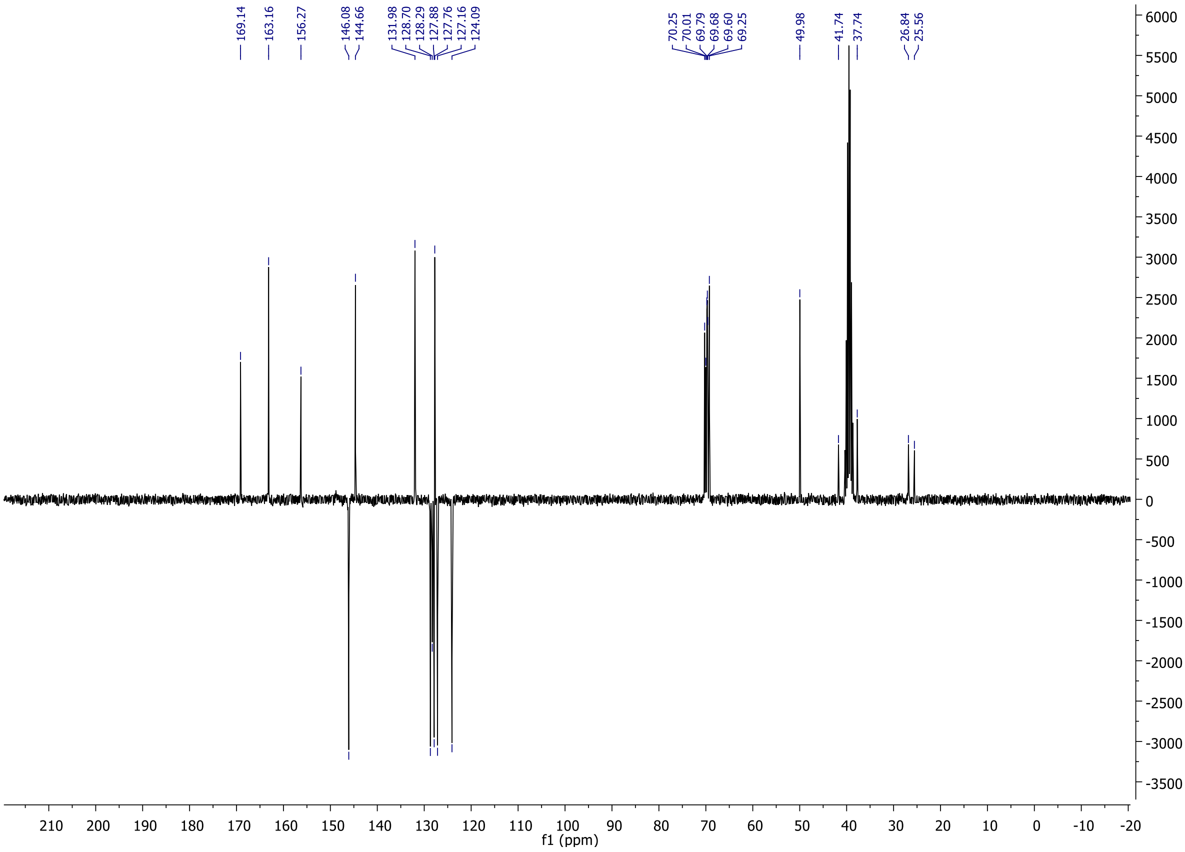

PDC-4,2-Alk - ¹H NMR

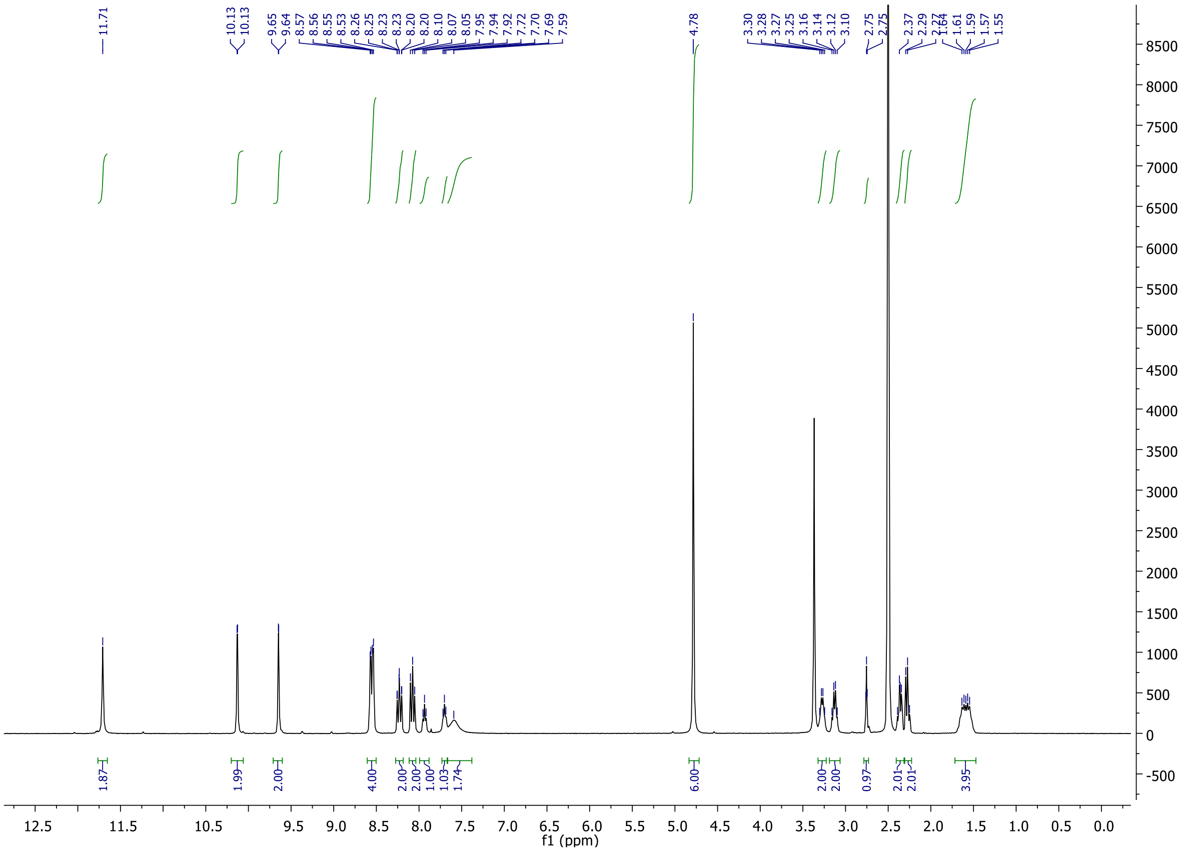

PDC-4,2-Alk - APT ¹³C NMR

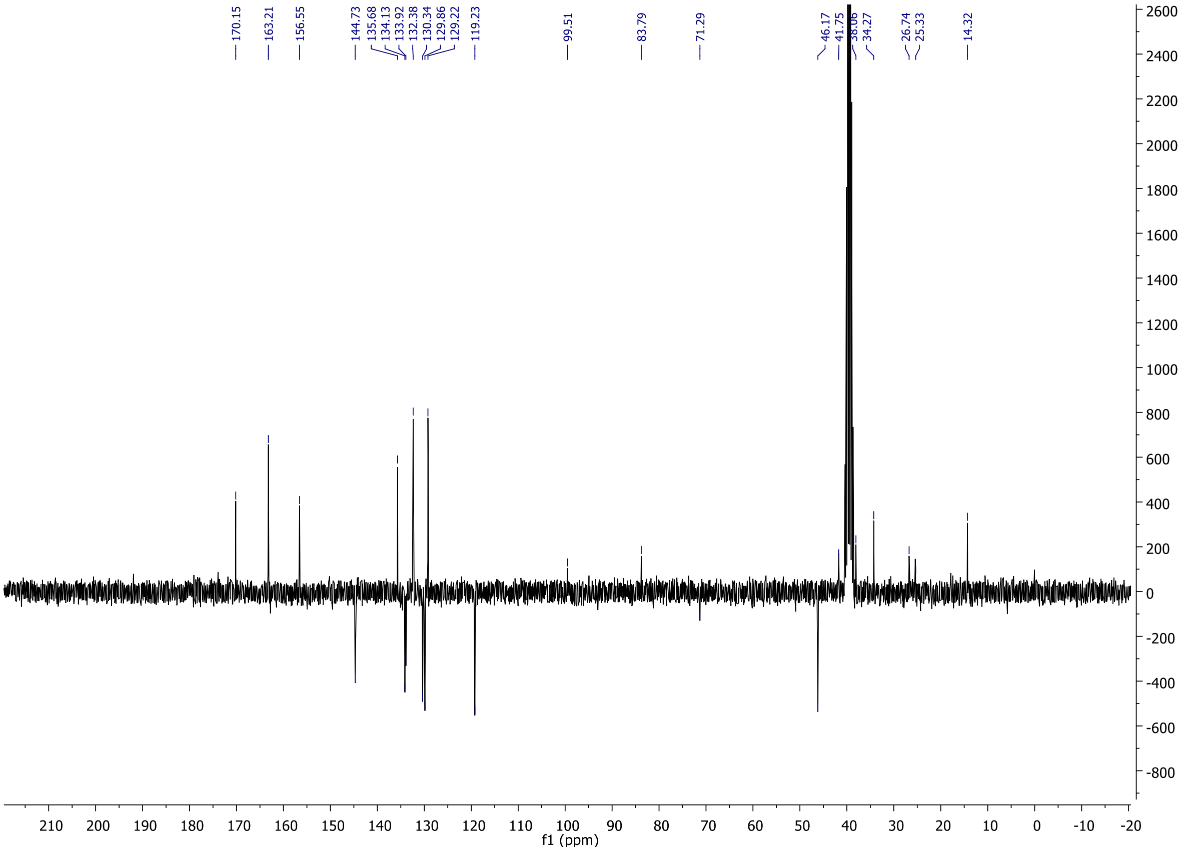

PDC-4,3-Alk - ^{1}H NMR

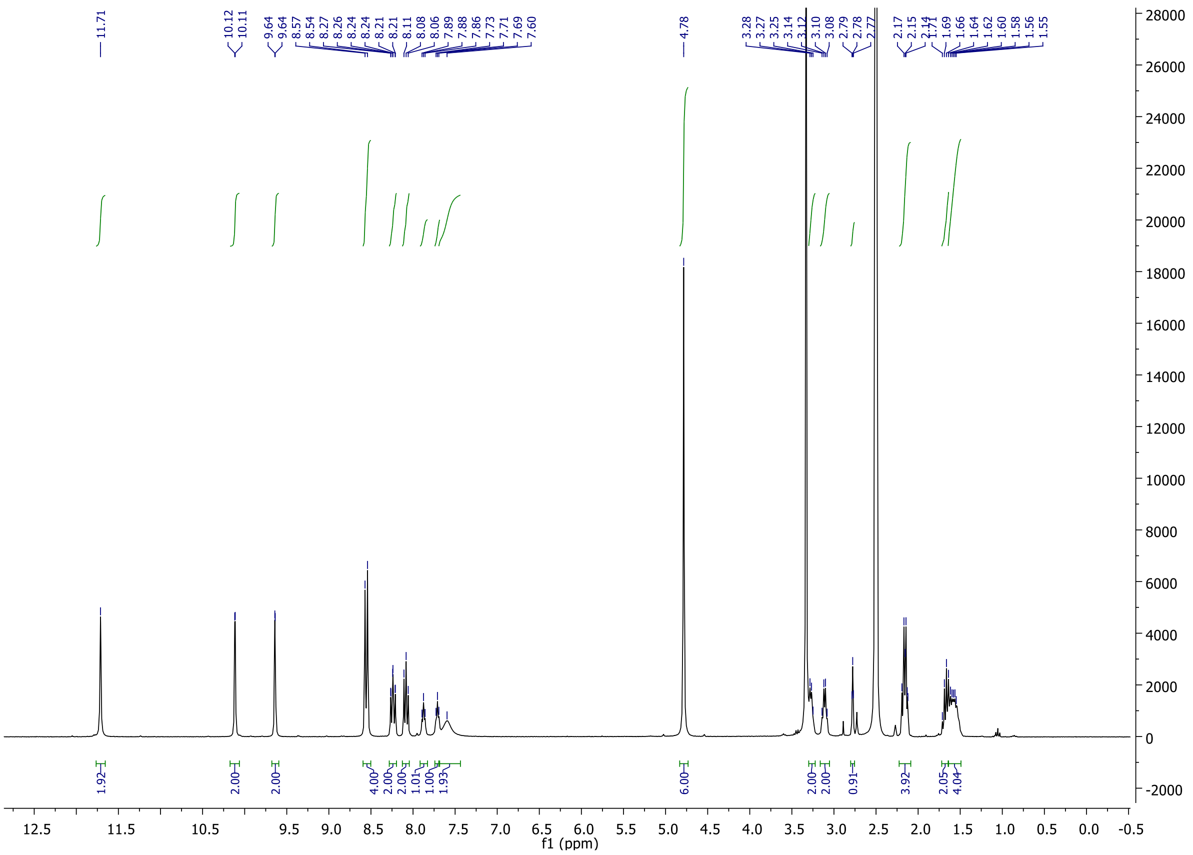

PDC-4,3-Alk – APT ^{13}C NMR

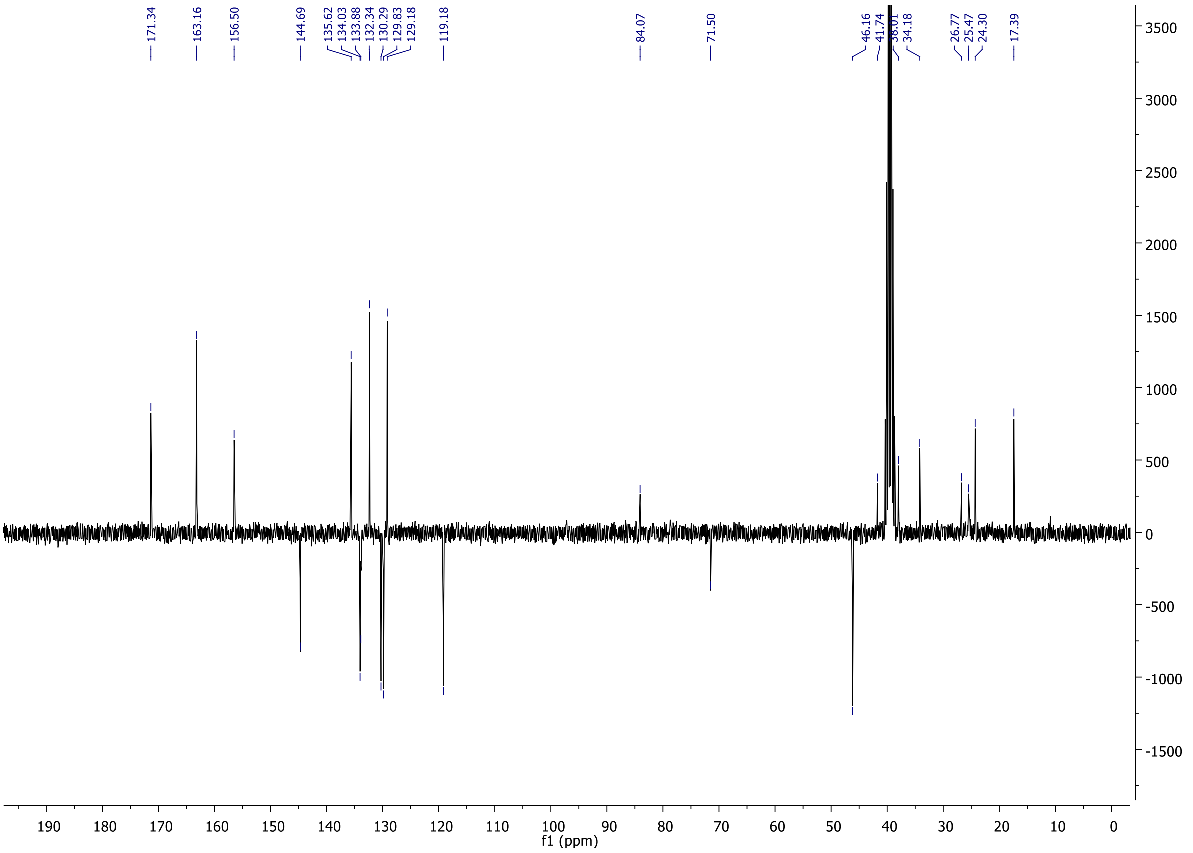

PDC-4,PEG-N3 - ^{1}H NMR

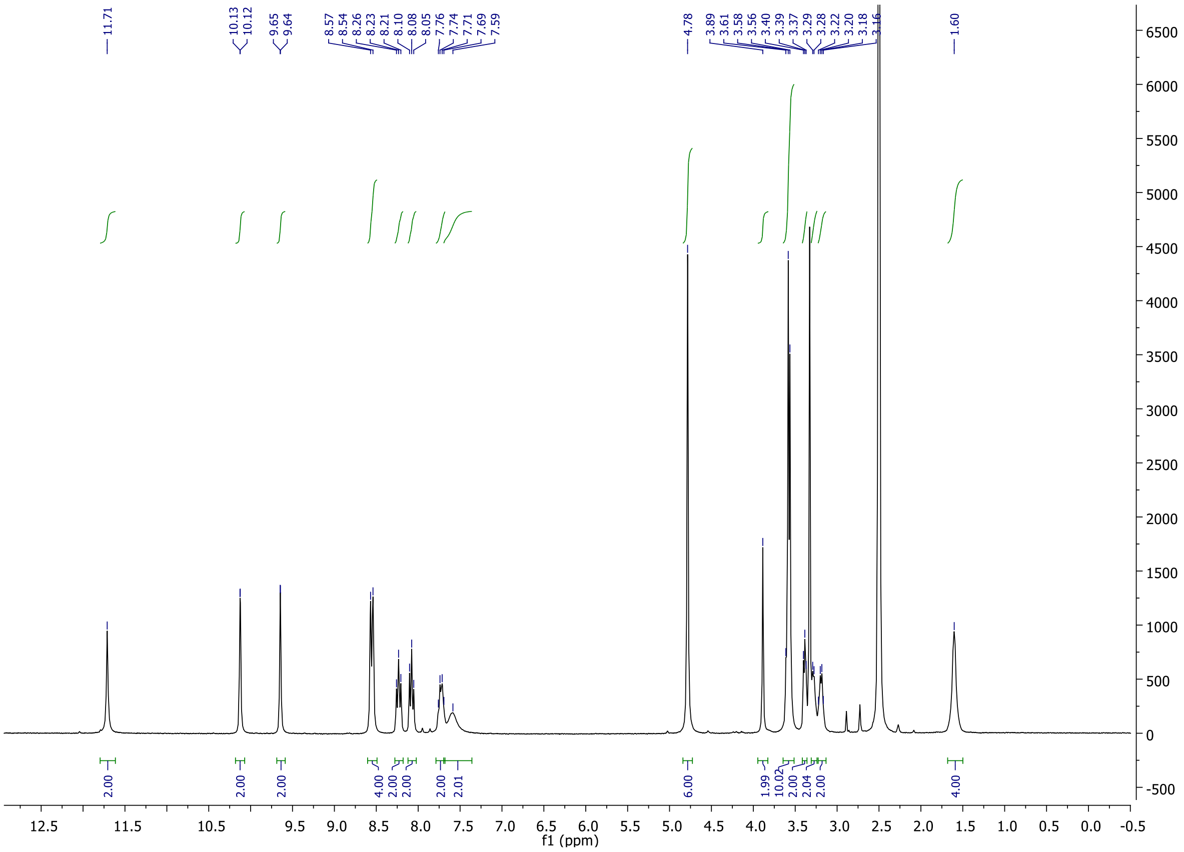

PDC-4,PEG-N3 – APT ^{13}C NMR

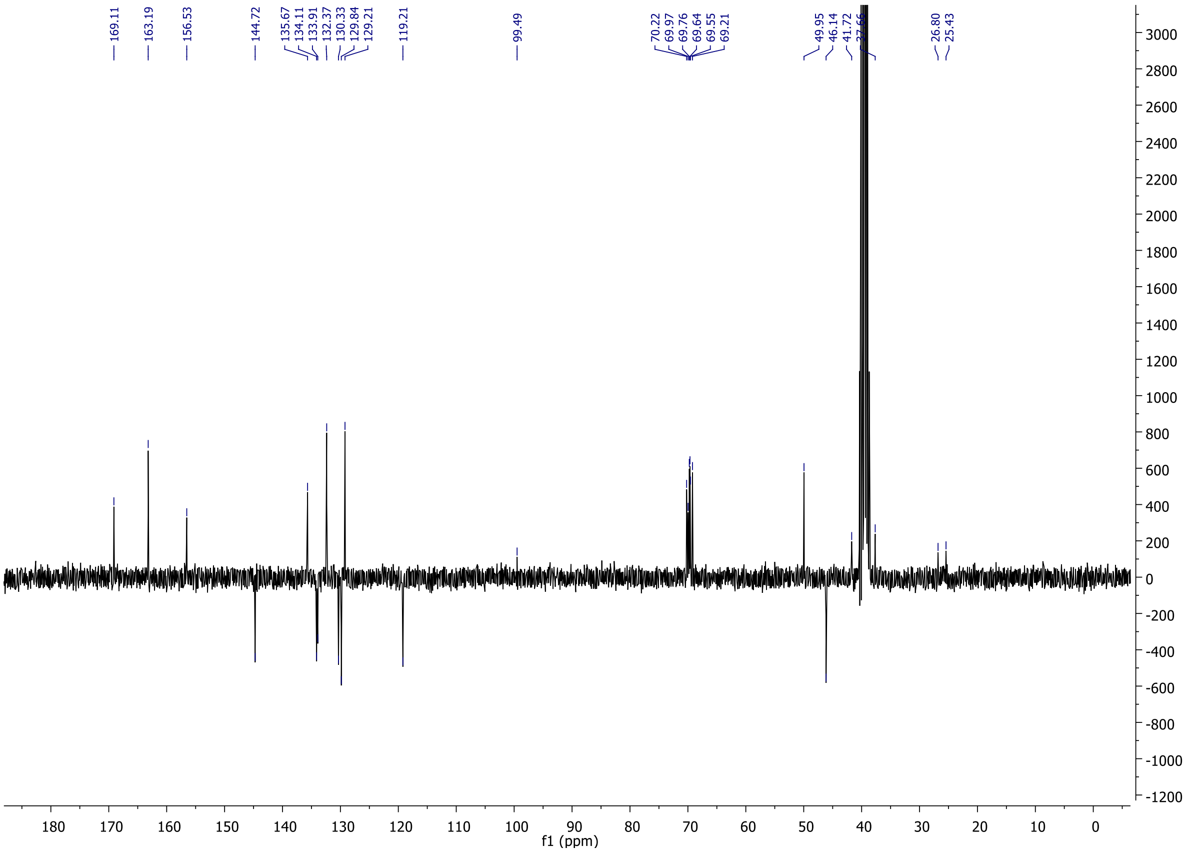

Compound 4 – ^{1}H NMR

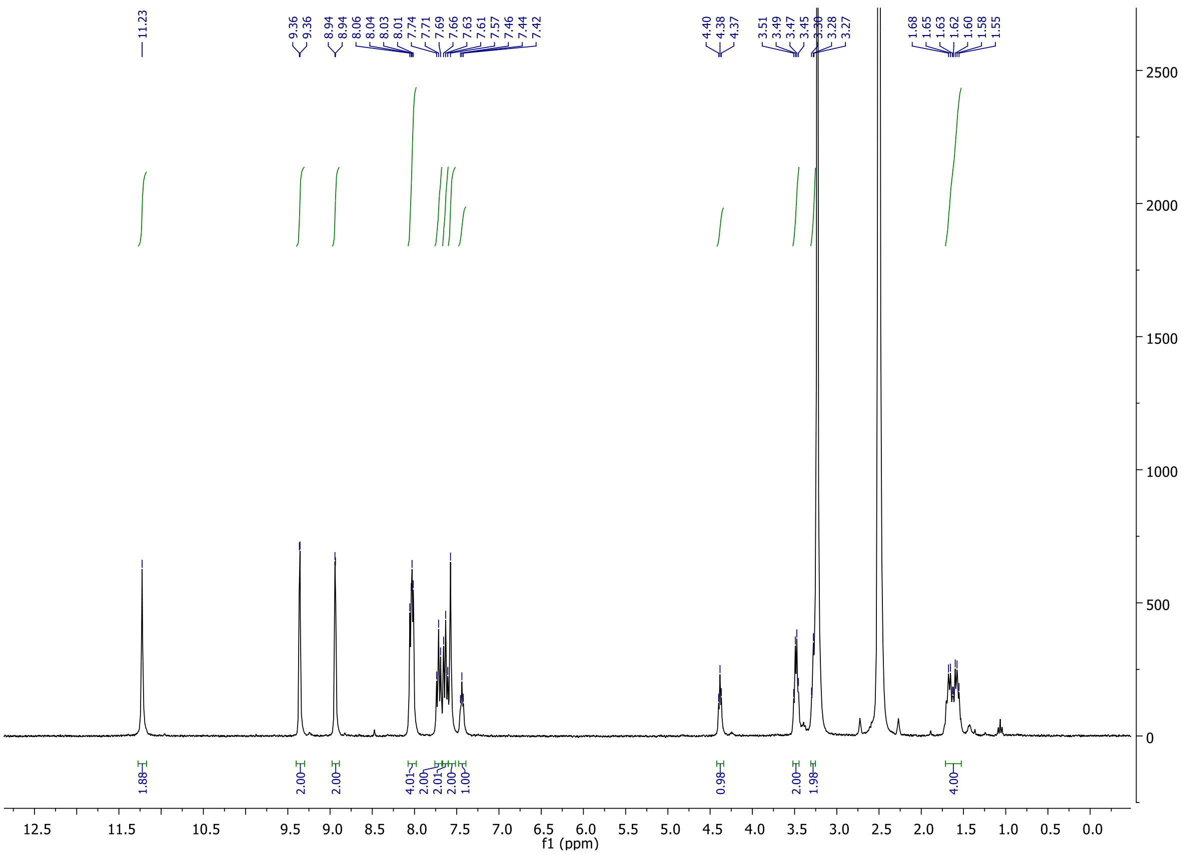

Compound 4 – APT ^{13}C NMR

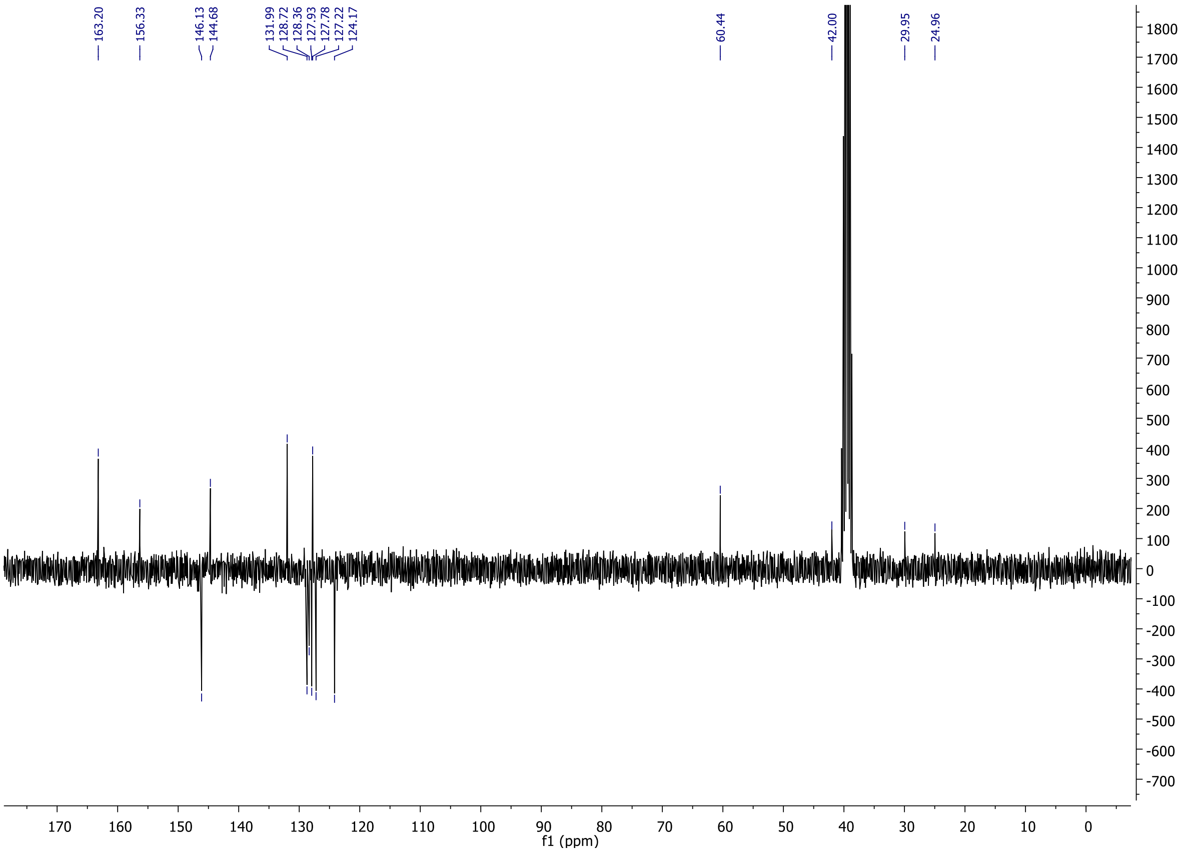

Compound 5 – ^{1}H NMR

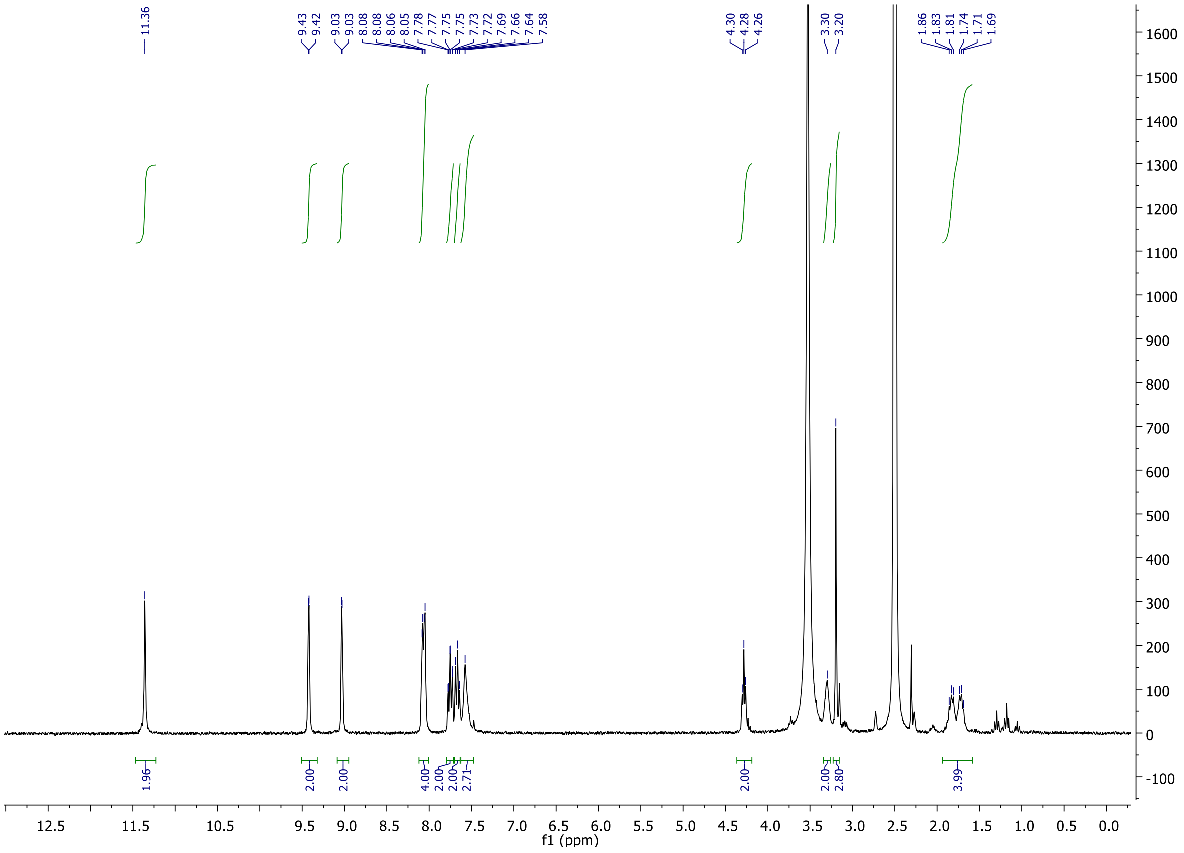

Compound 6 – ^{1}H NMR

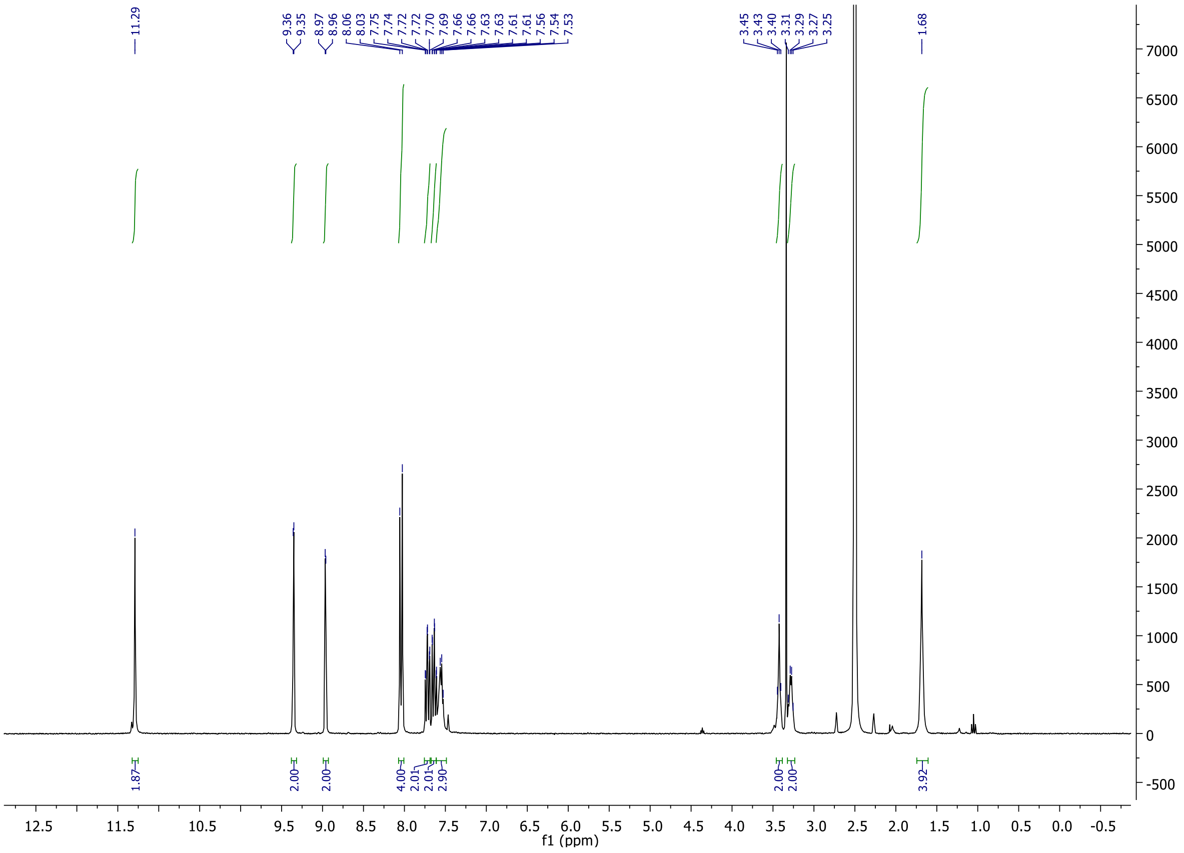

Compound 6 – APT ^{13}C NMR

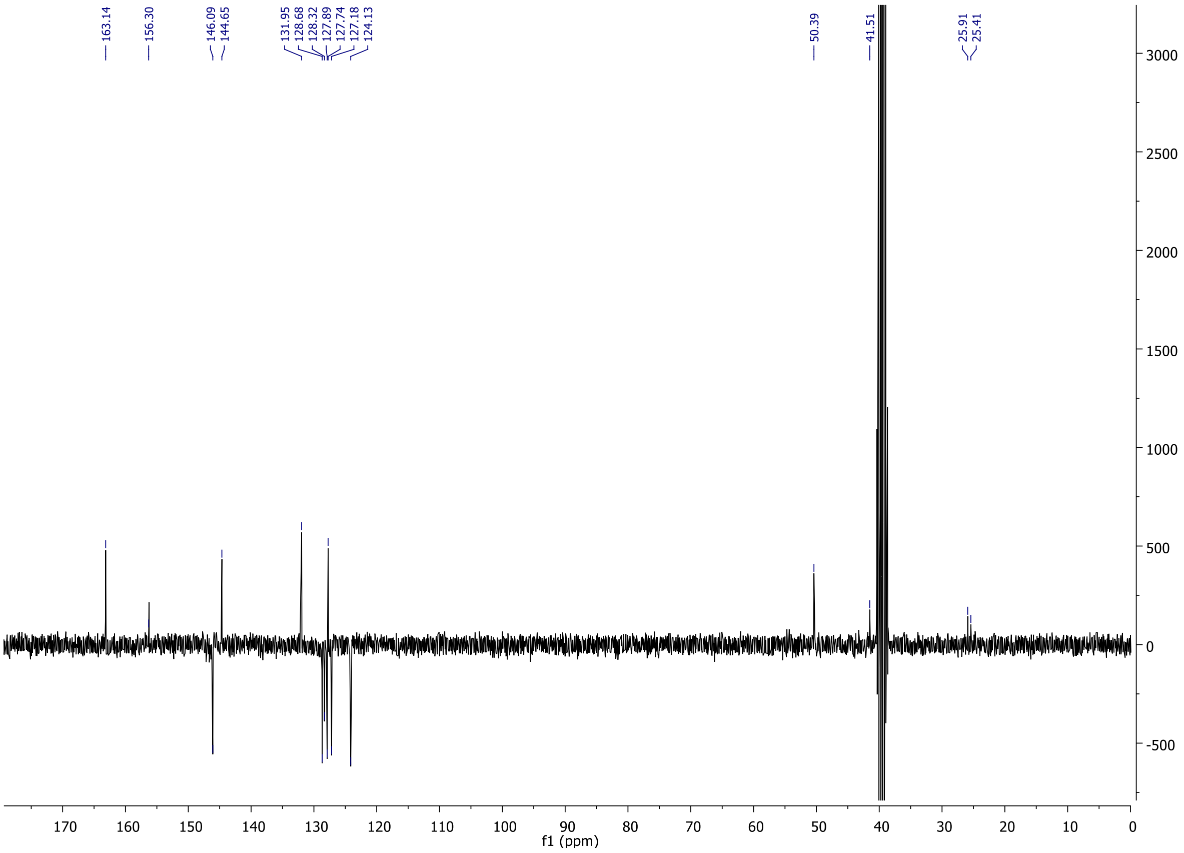

PDC-4,0-N3 – ^{1}H NMR

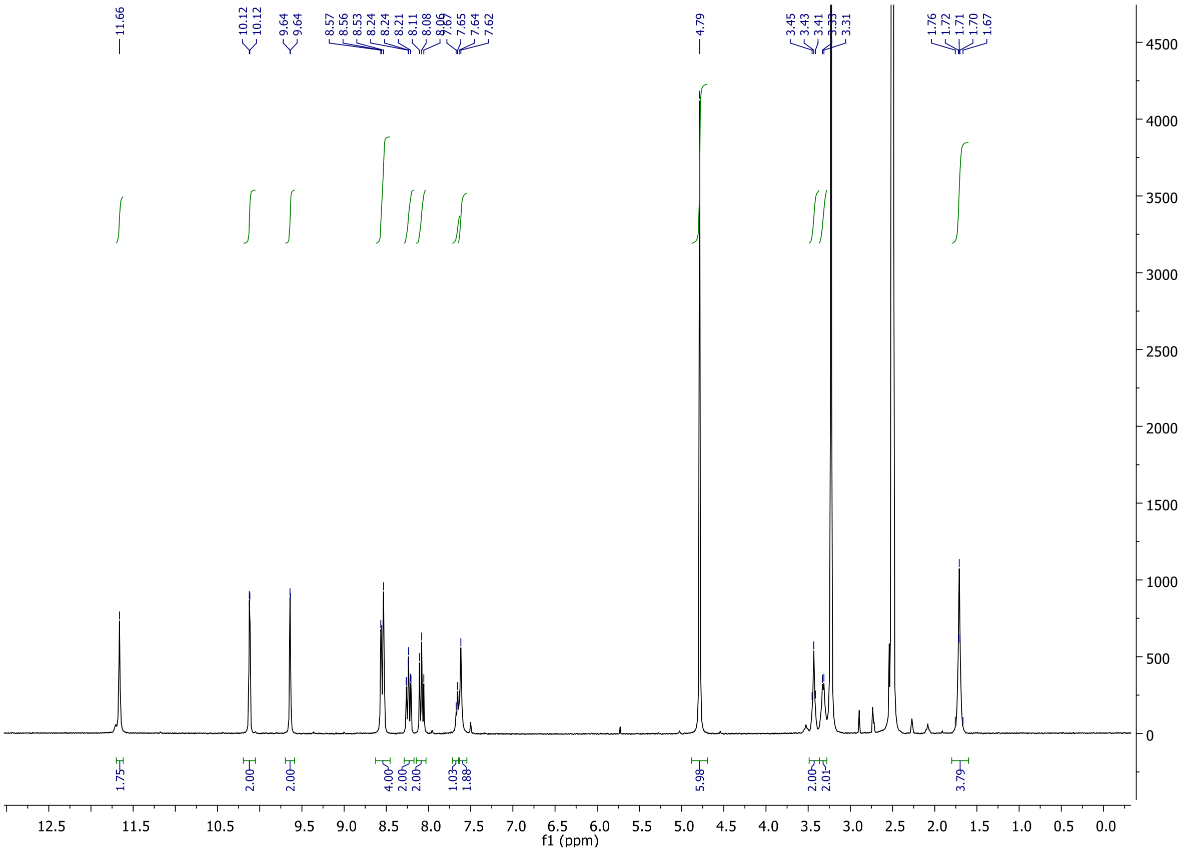

PDC-4,0-N3 – APT ^{13}C NMR

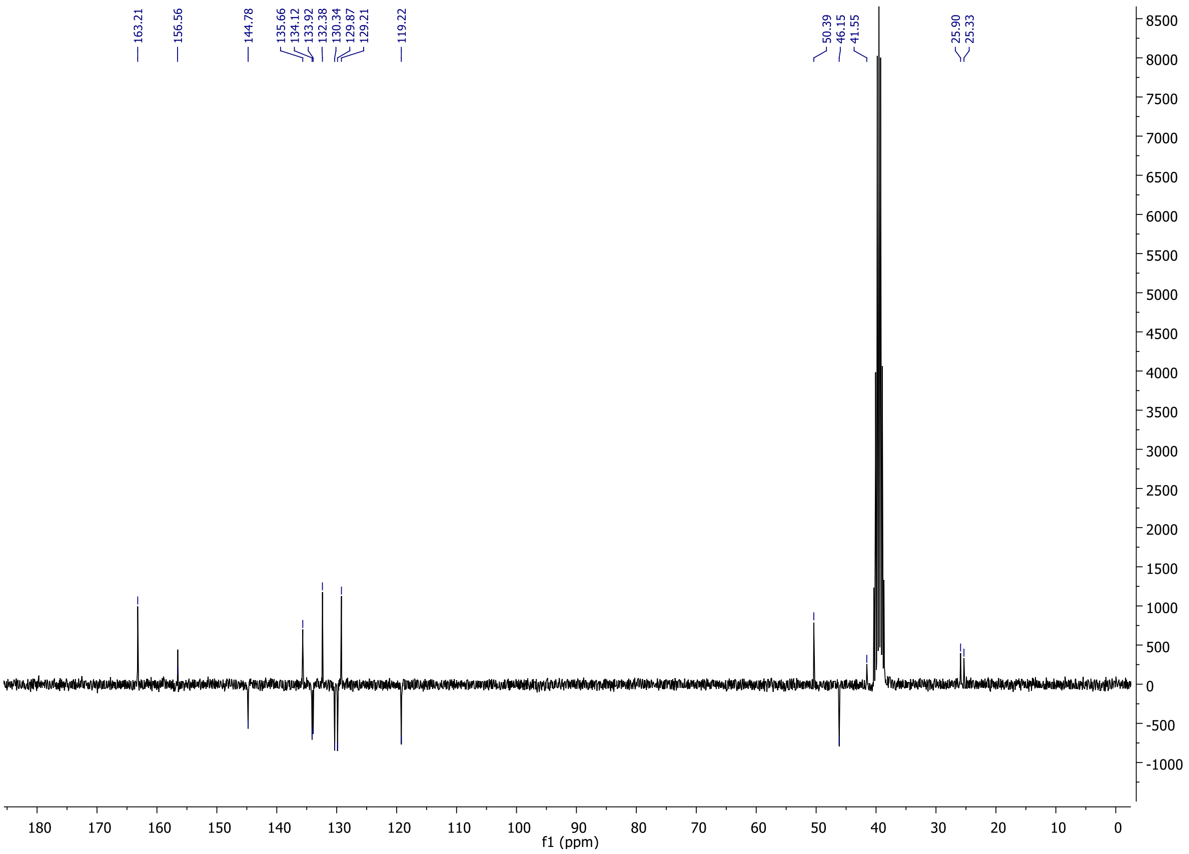

Compound 7 – ^{1}H NMR

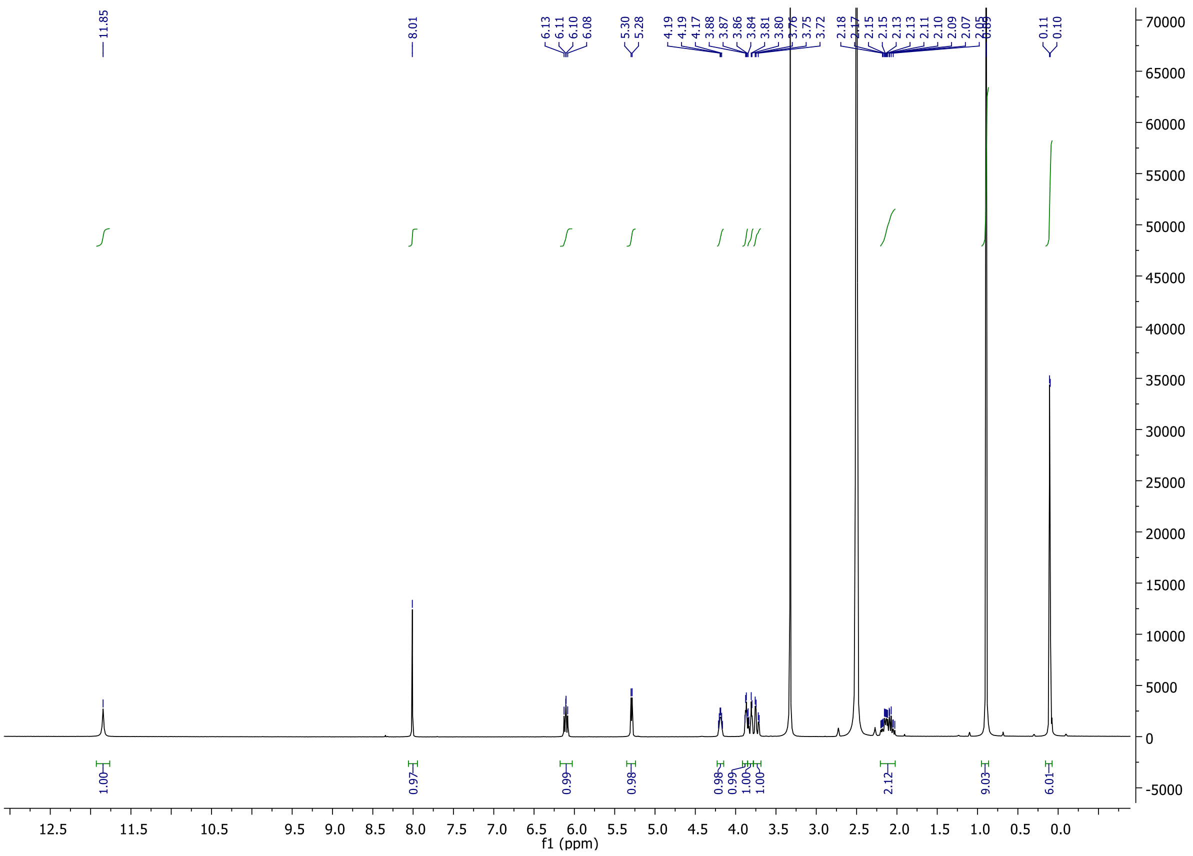

Compound 7 – APT ^{13}C NMR

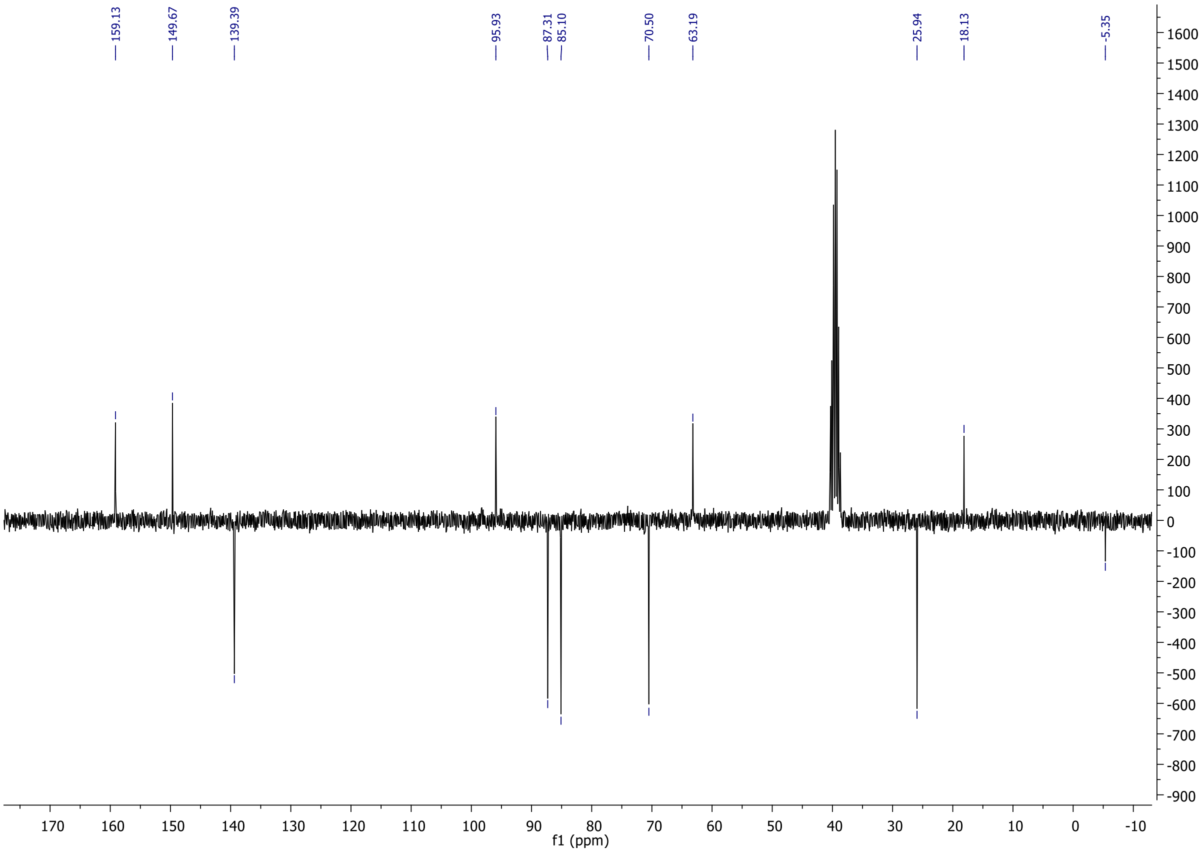

Compound 8 – ^{1}H NMR

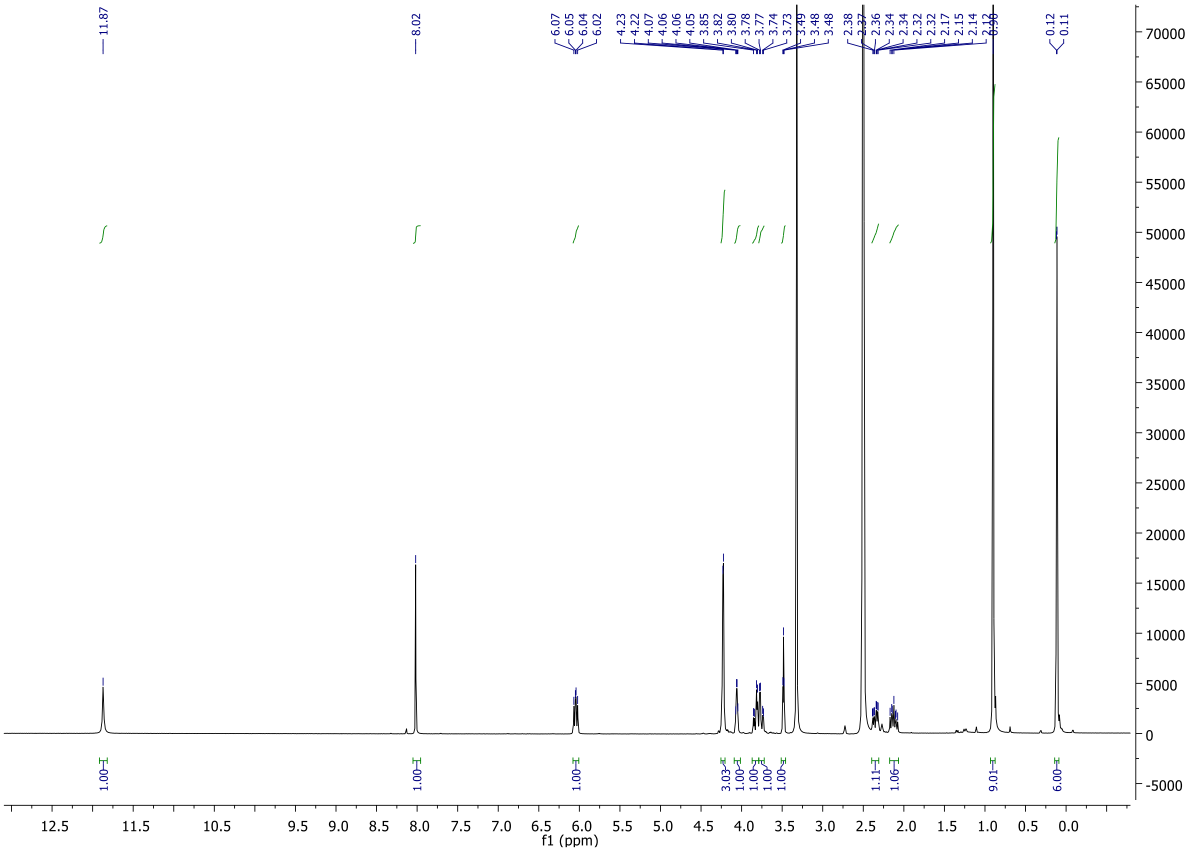

Compound 8 – APT ^{13}C NMR

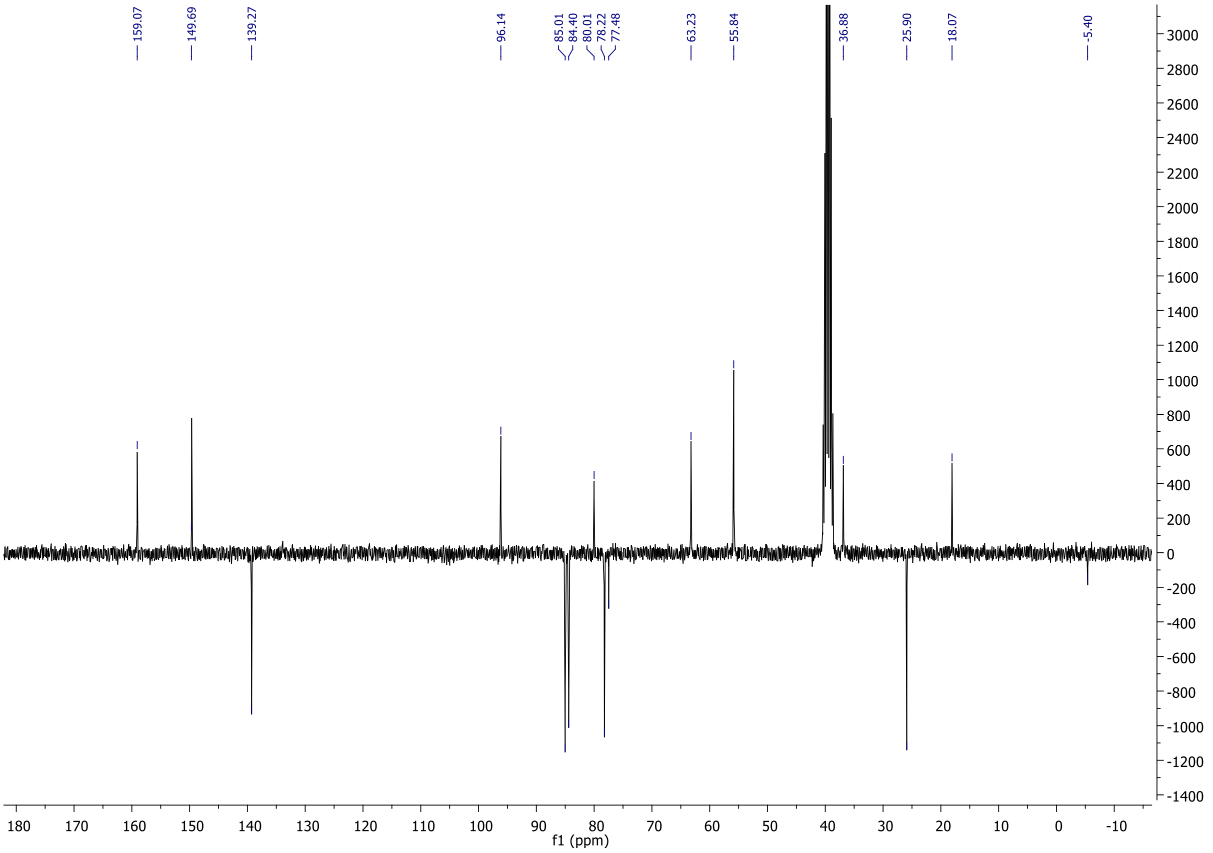

5-BrdU-Alk – ^{1}H NMR

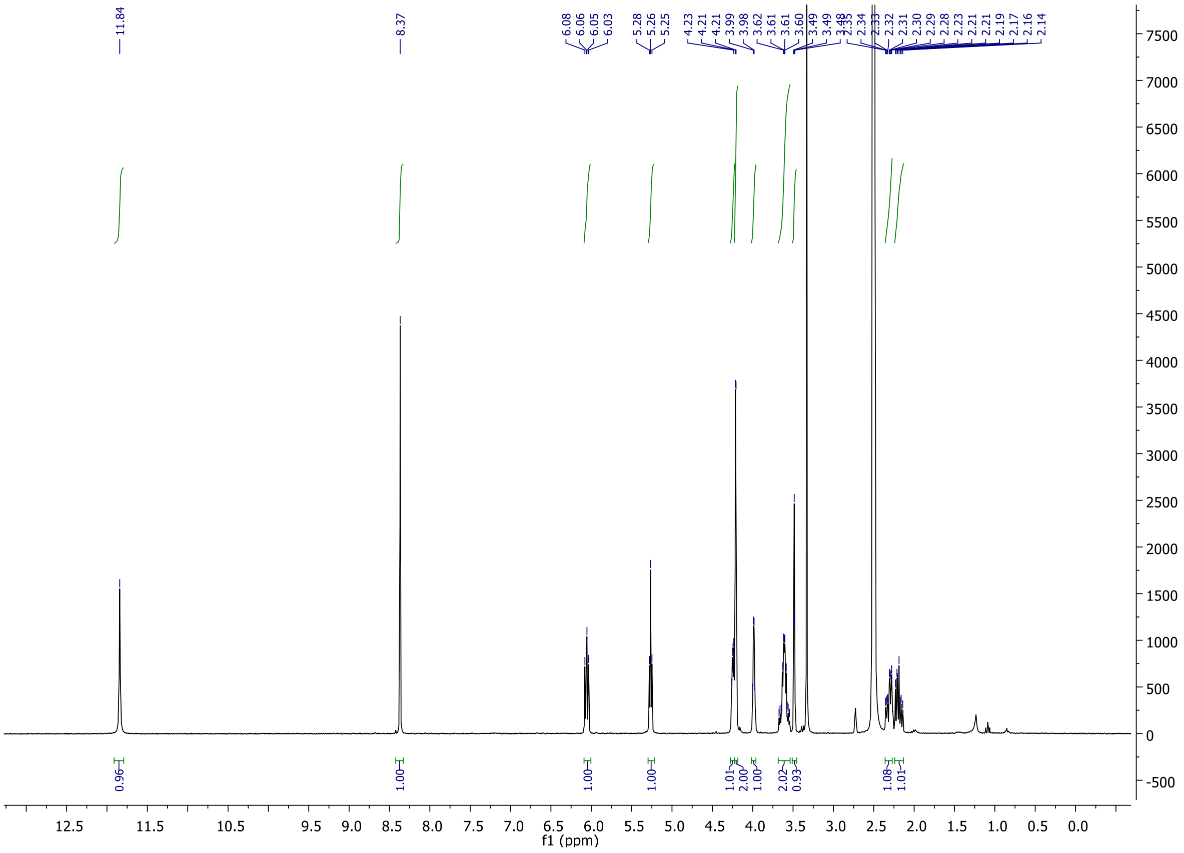

5-BrdU-Alk – APT ^{13}C NMR

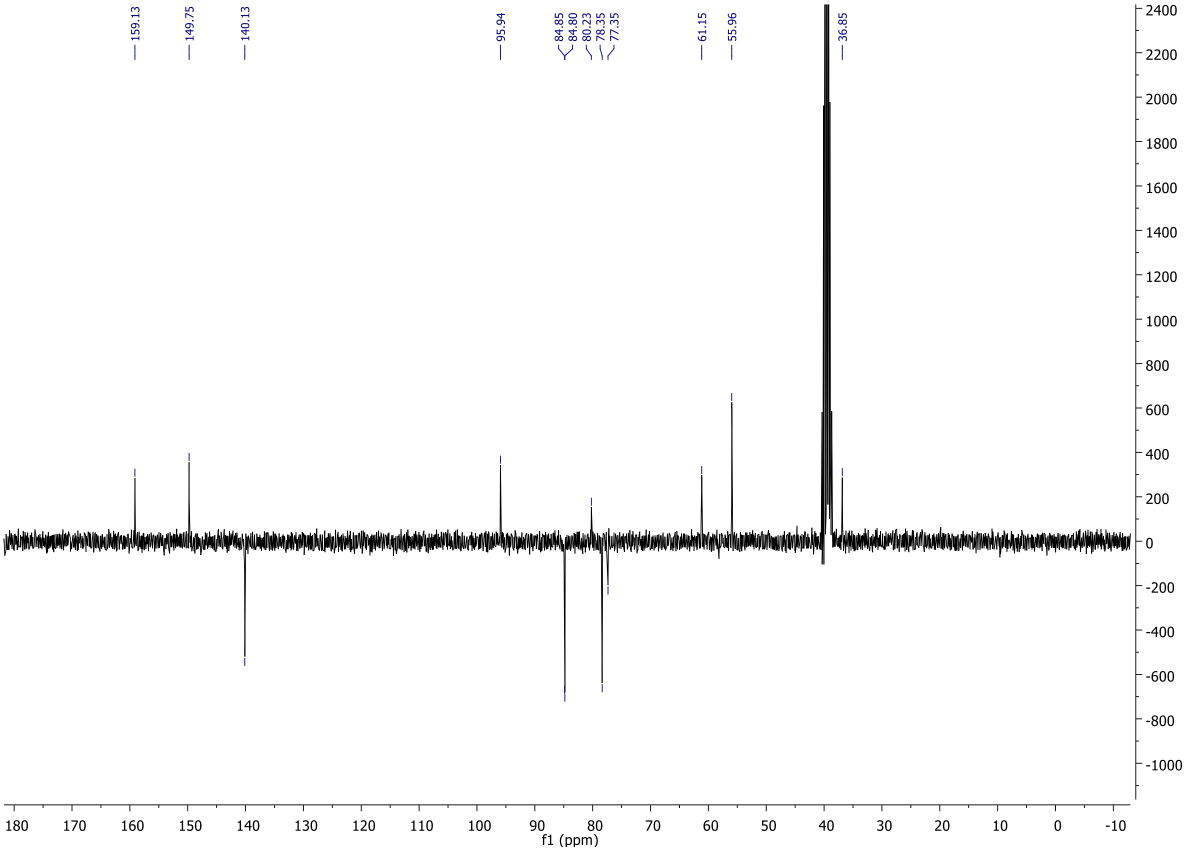

Compound 9 – ^{1}H NMR

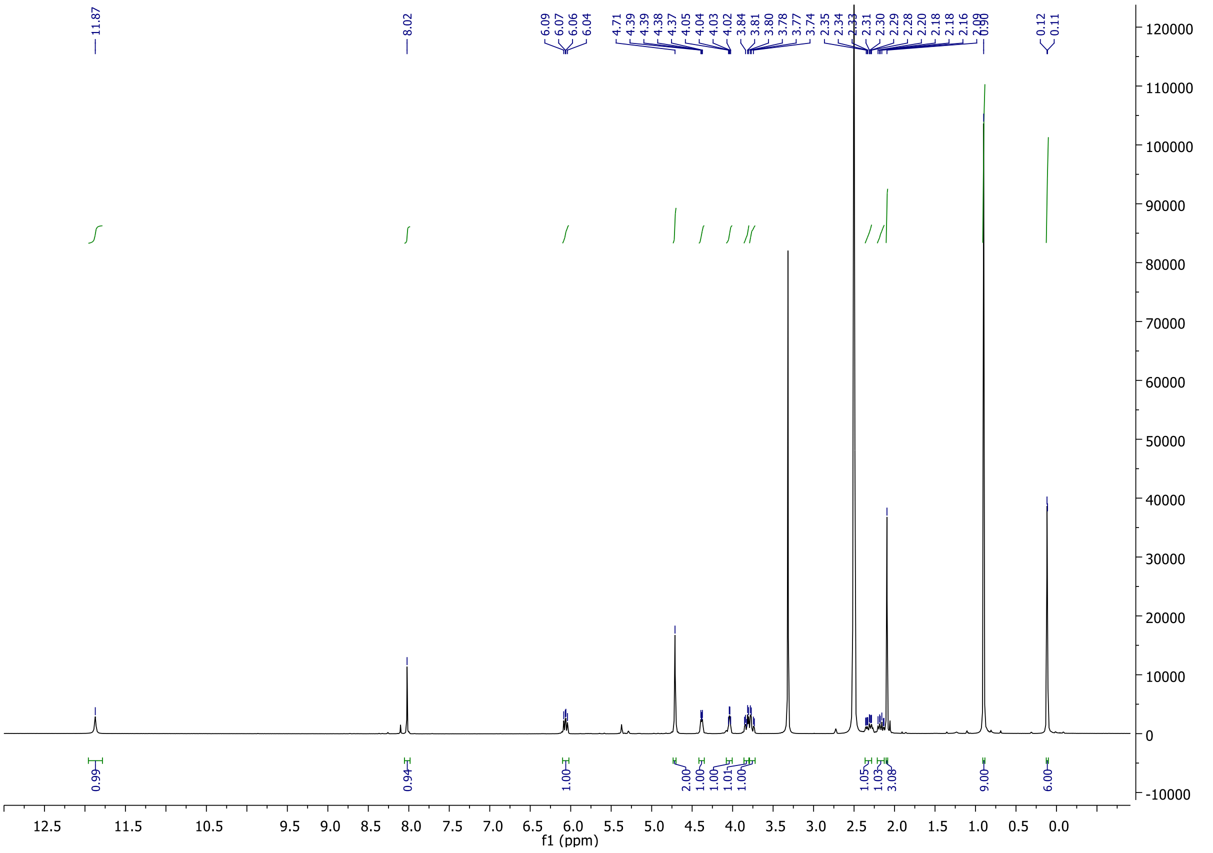

Compound 9 – APT ^{13}C NMR

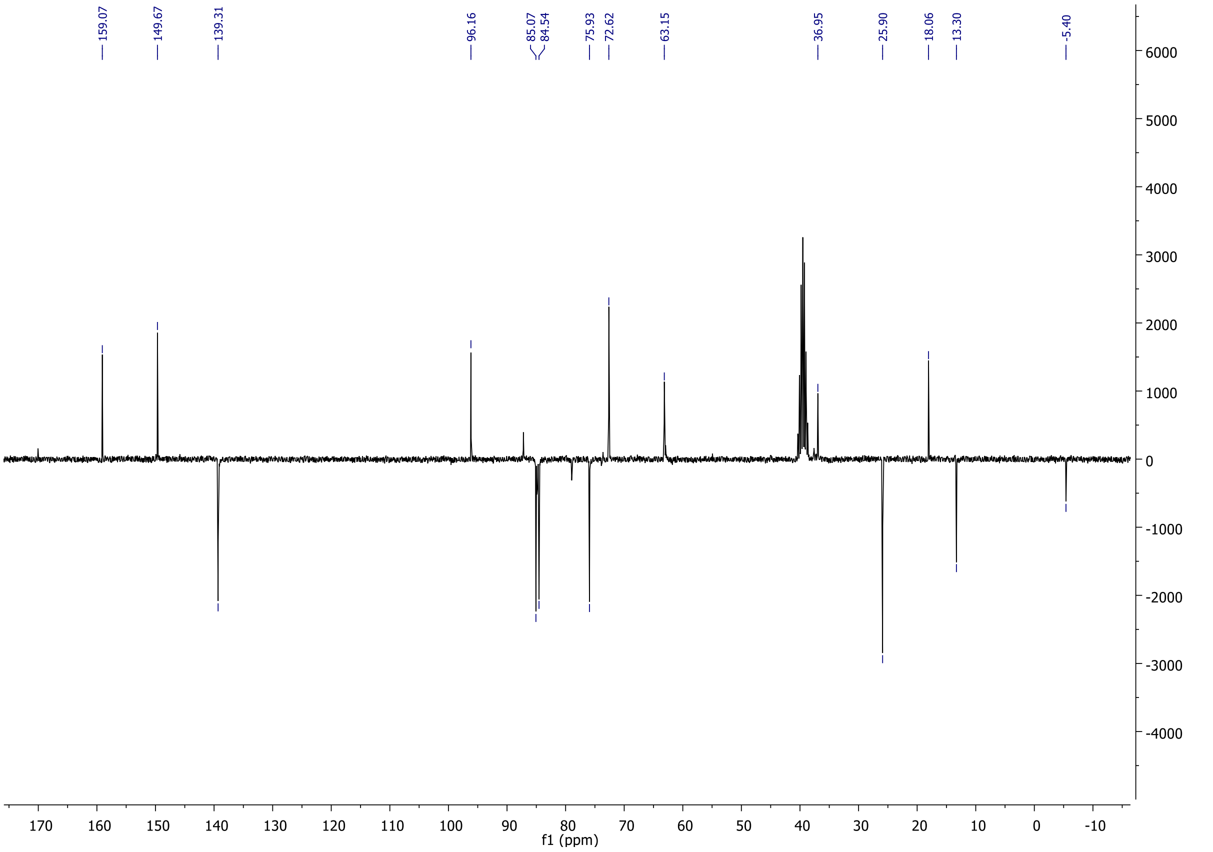

Compound 10 – ^{1}H NMR

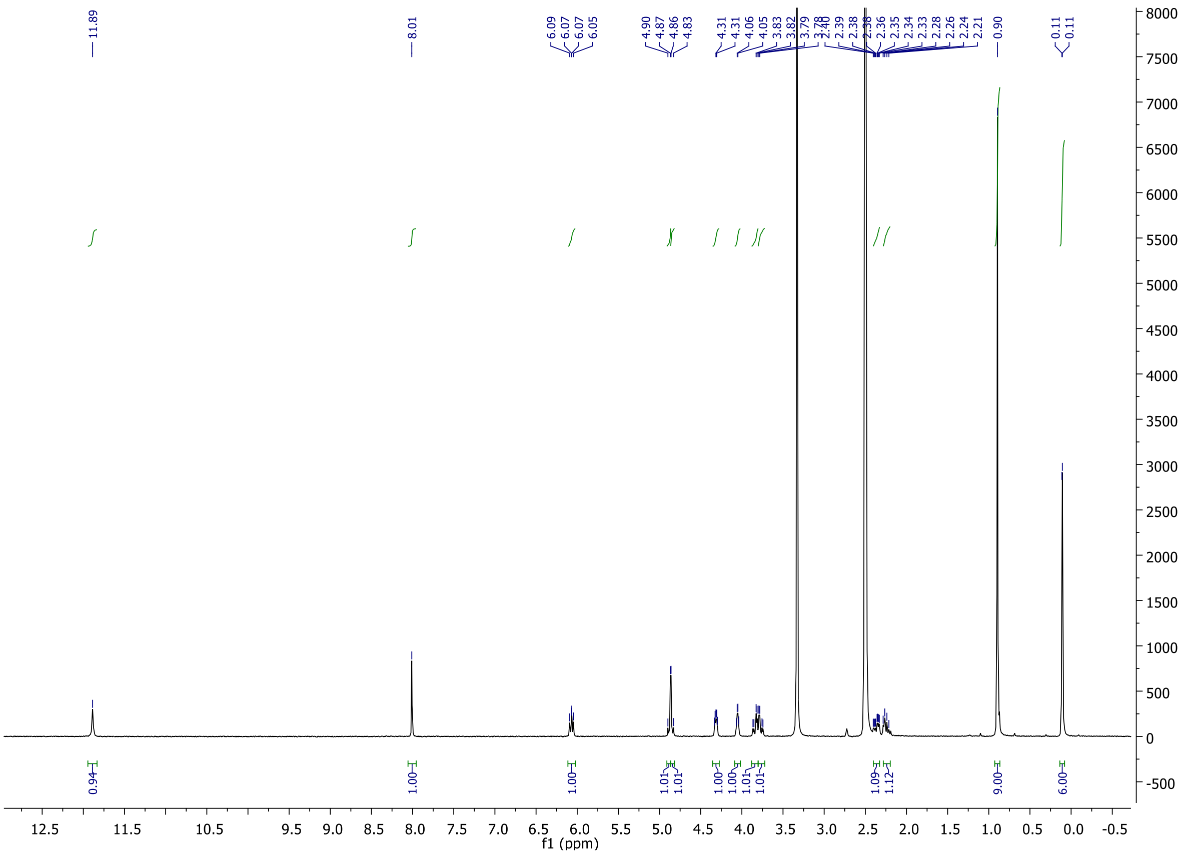

Compound 10 – APT ^{13}C NMR

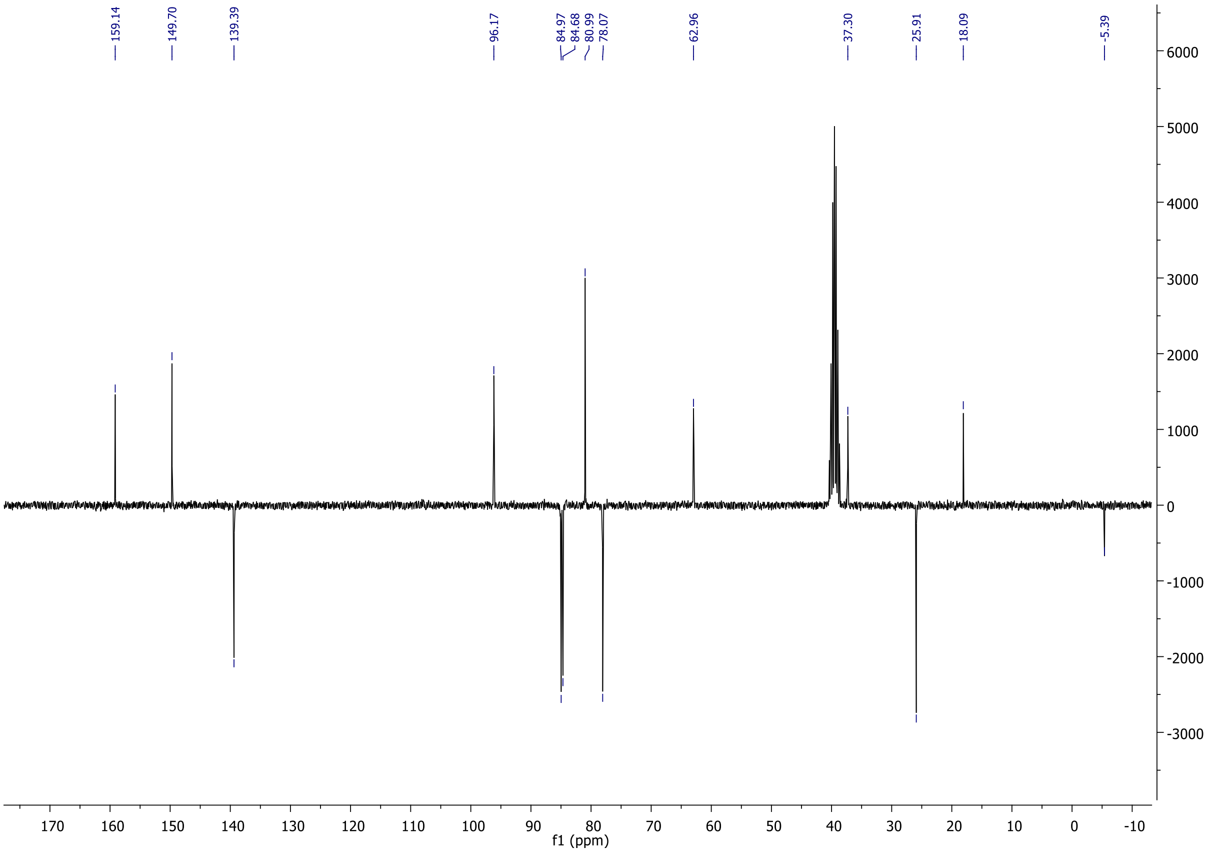

5-BrdU-N3 – ^{1}H NMR

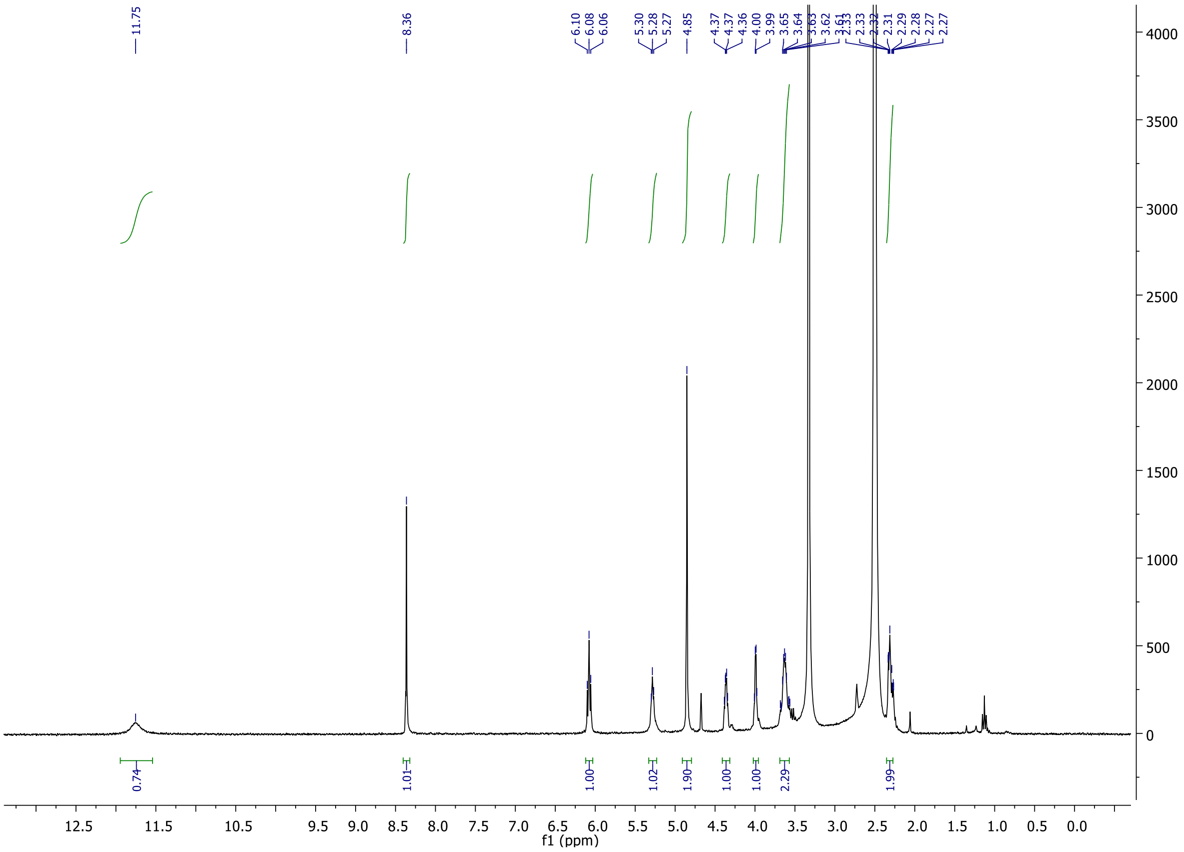

5-BrdU-N3 – APT ^{13}C NMR

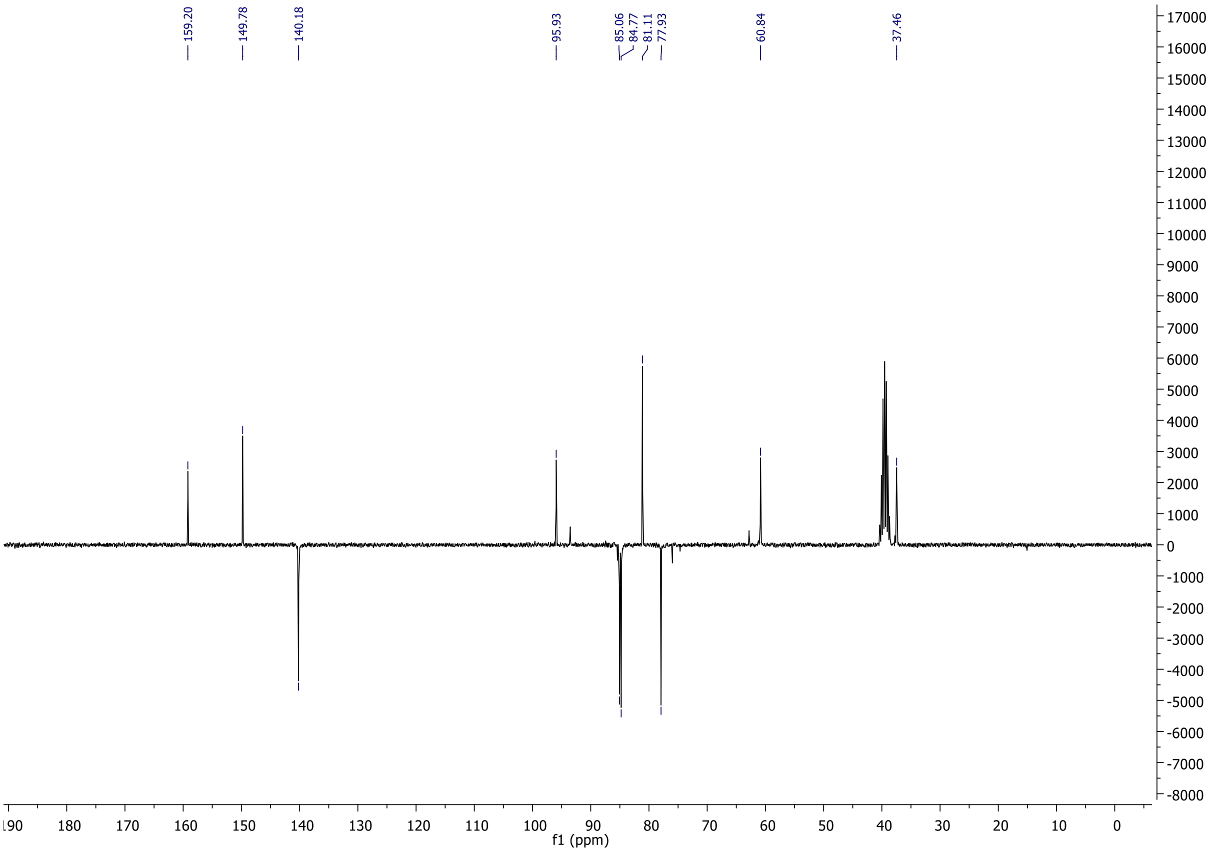

PDC-4,2-BrdU – ^{1}H NMR

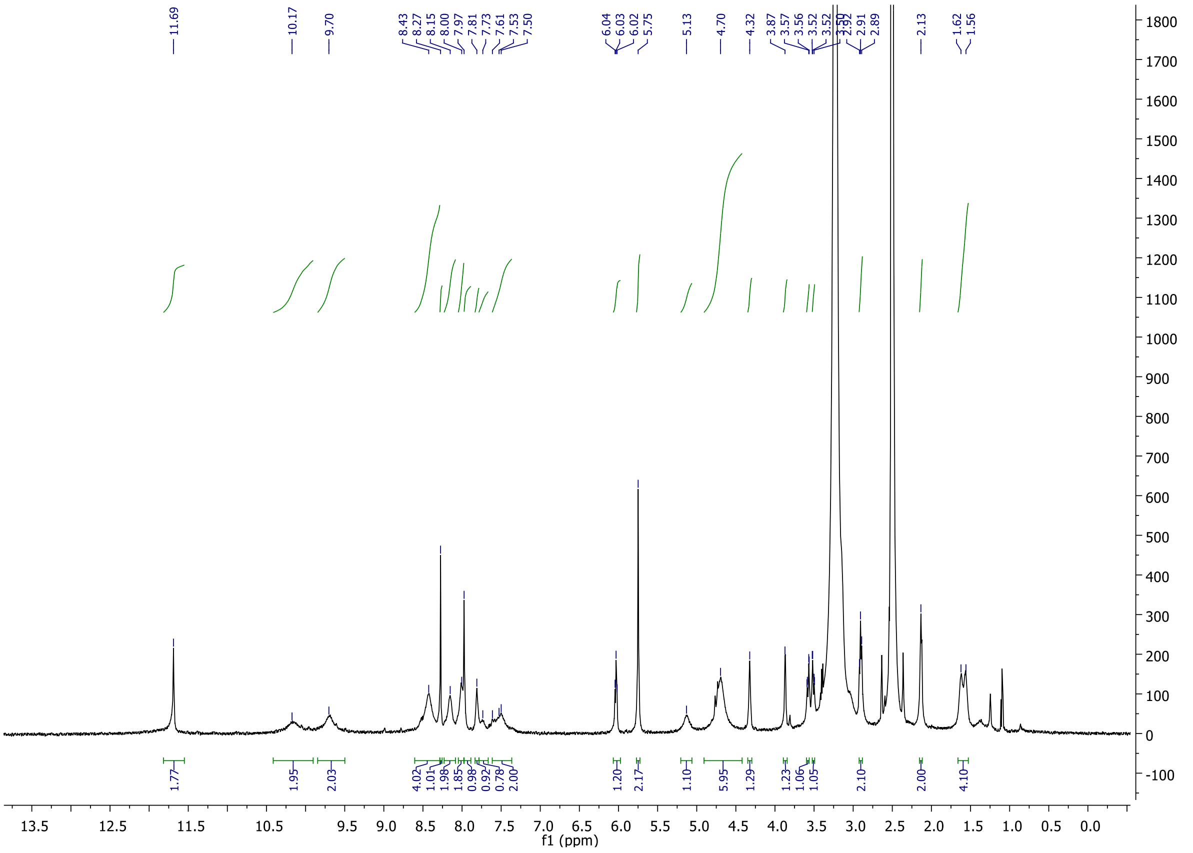

PDC-4,3-BrdU – ^{1}H NMR

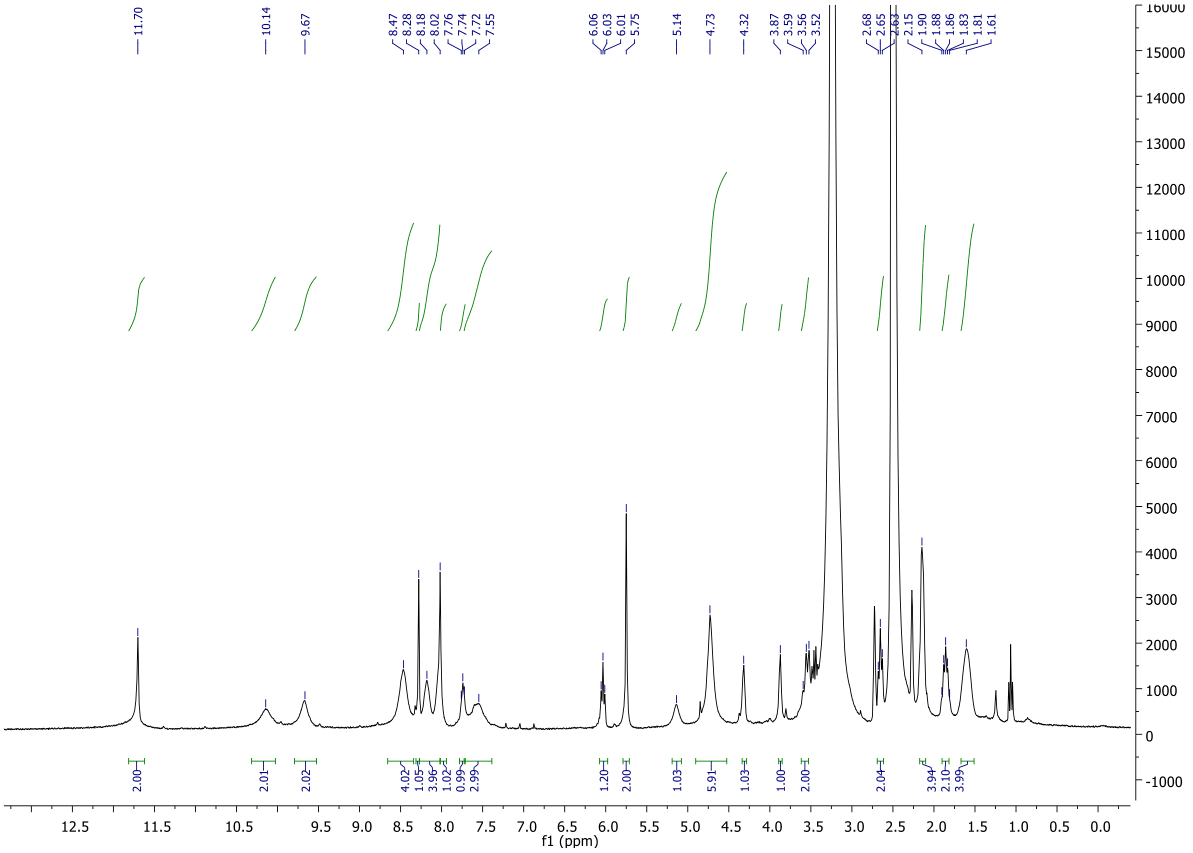

PDC-4,PEG-BrdU – ^{1}H NMR

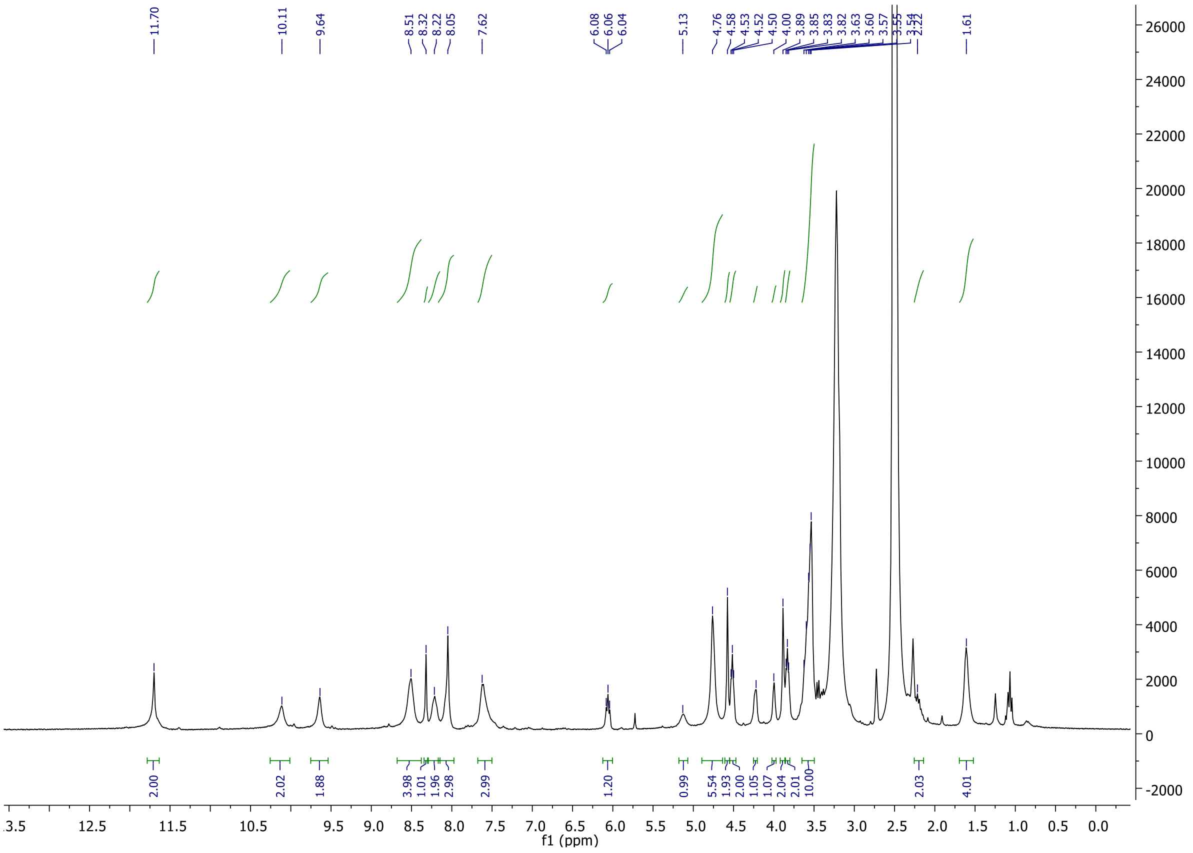

PDC-4,0-BrdU – ^{1}H NMR

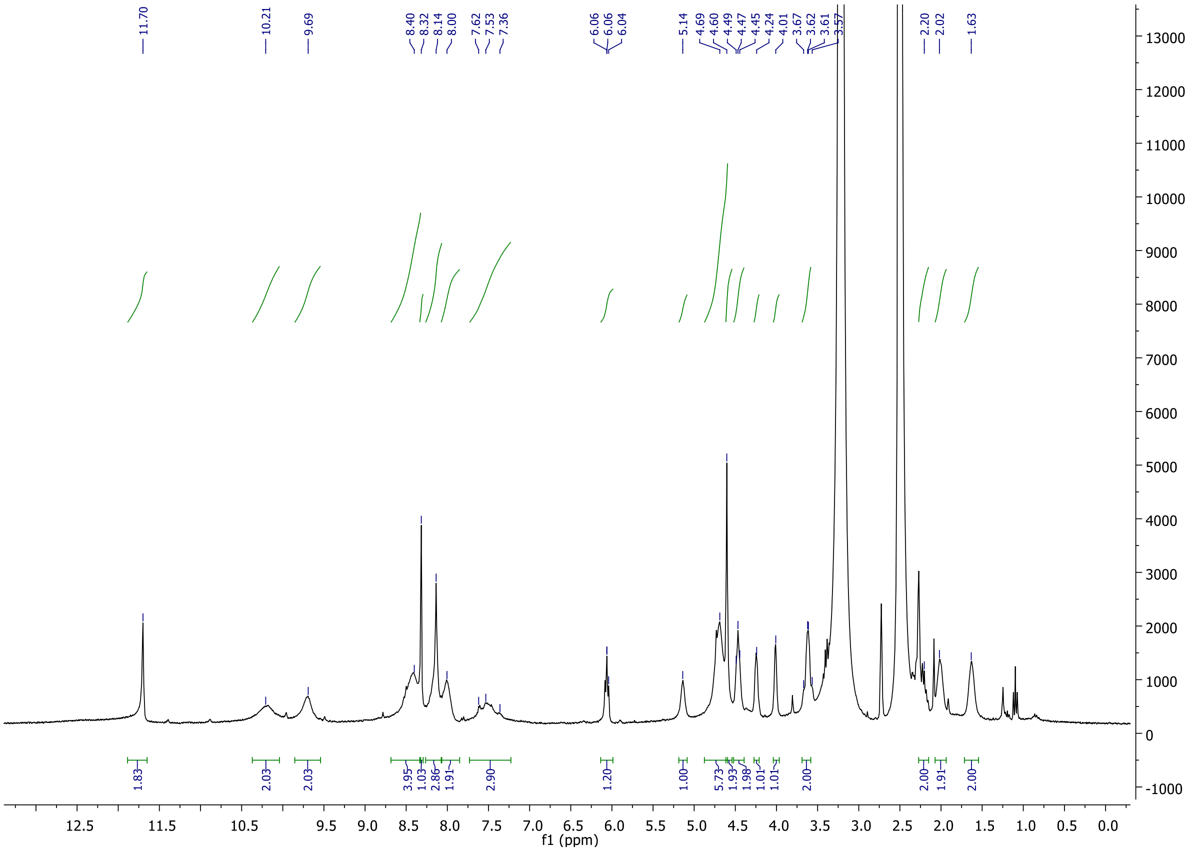

PDC-4,2-Alk - HPLC chromatogram

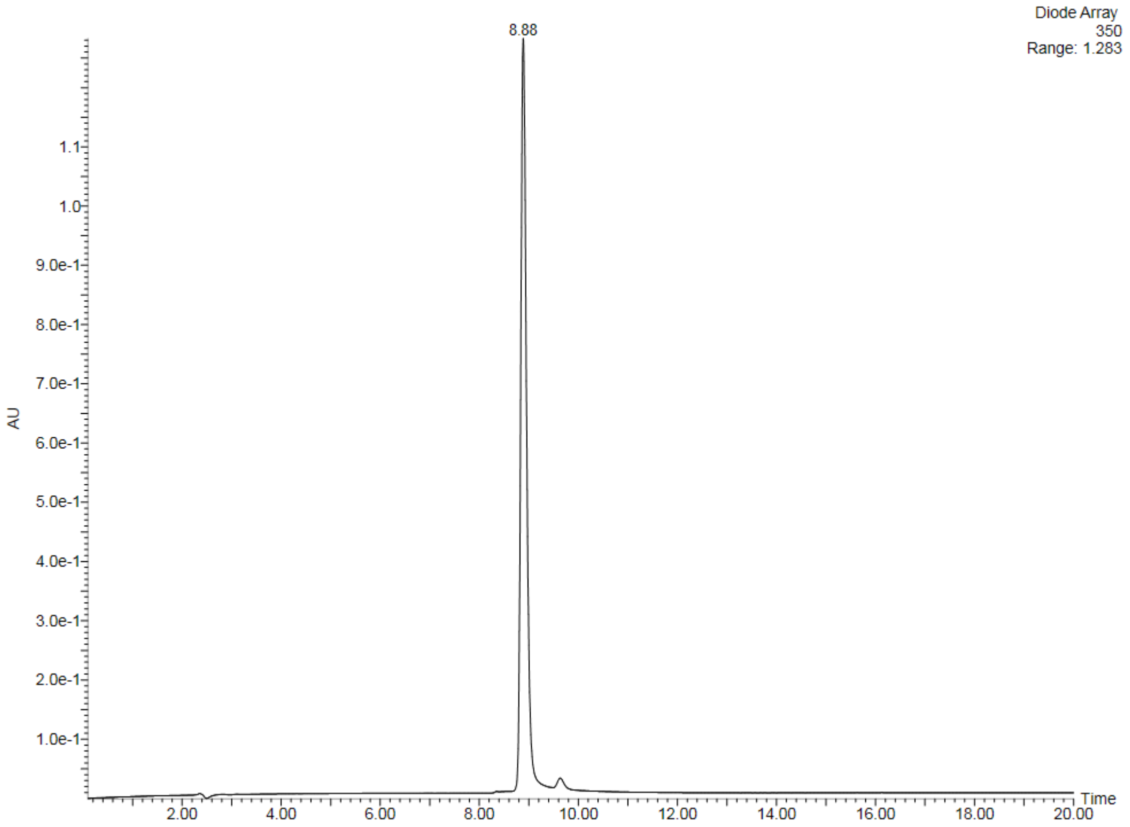

PDC-4,3-Alk - HPLC chromatogram

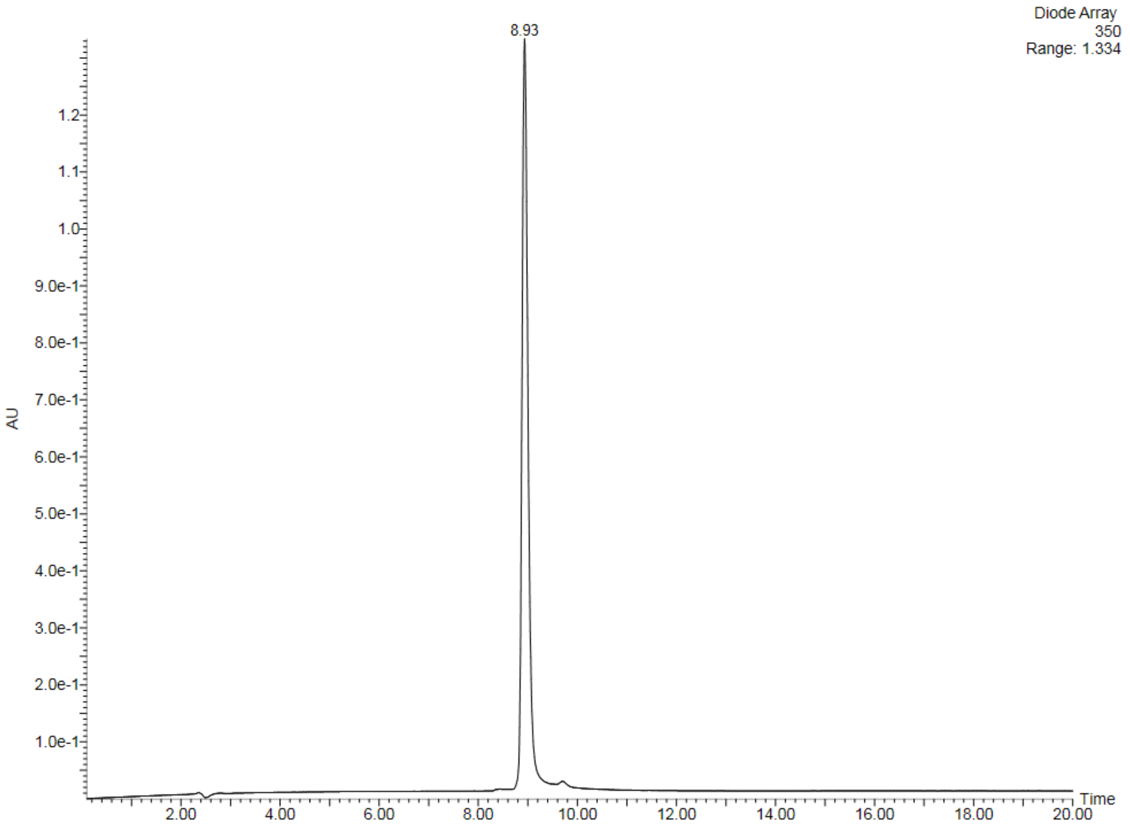

PDC-4,PEG-N3 - HPLC chromatogram

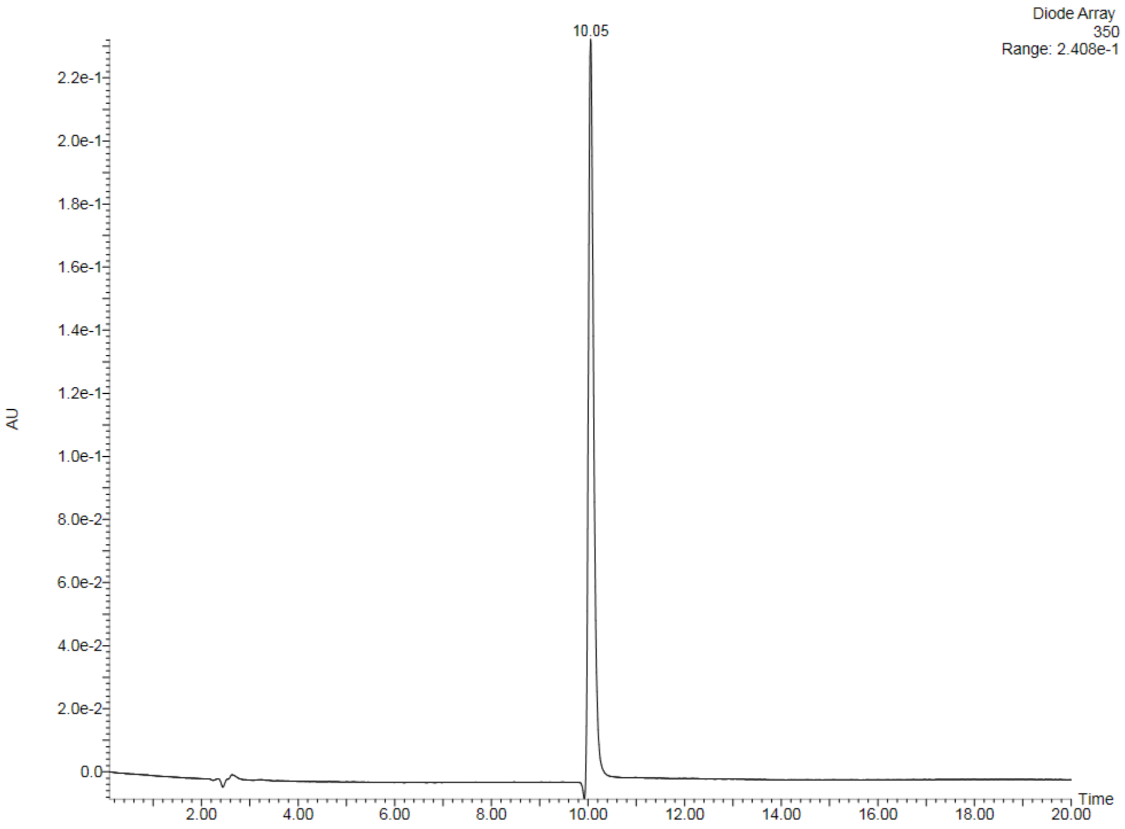

PDC-4,0-N3 - HPLC chromatogram

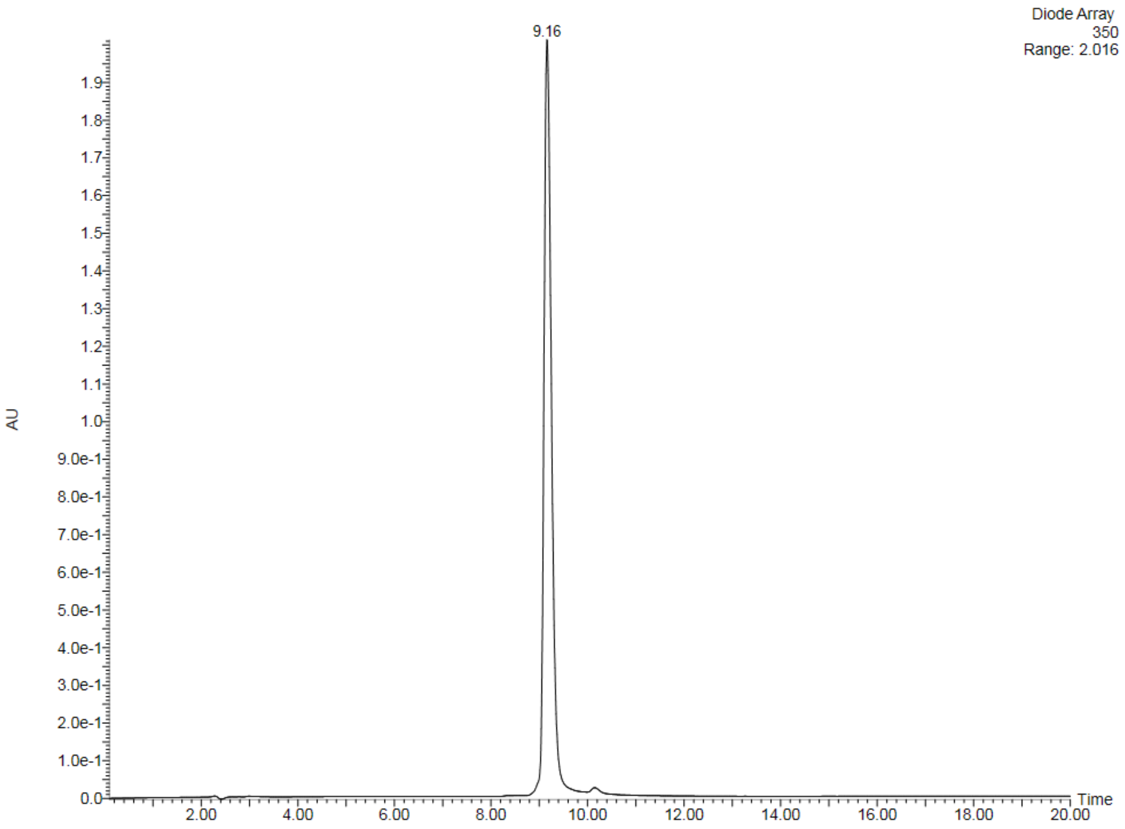

PDC-4,2-BrdU - HPLC chromatogram

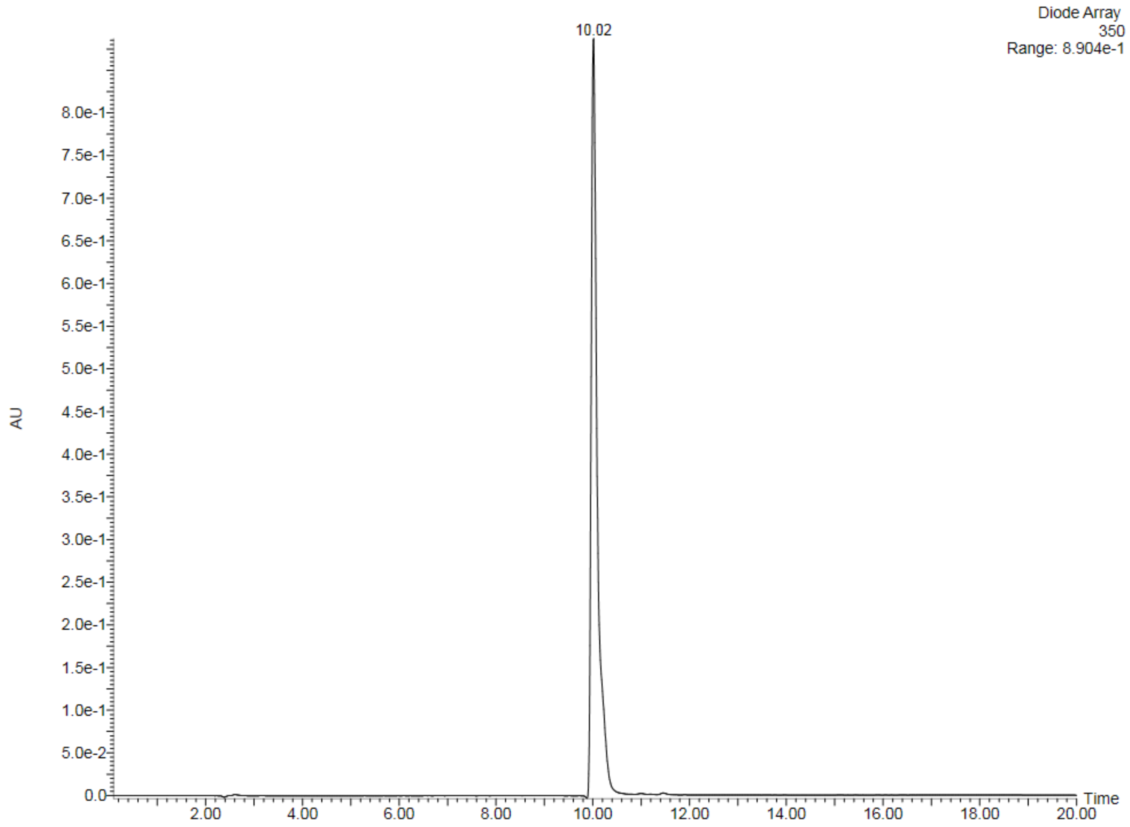

PDC-4,3-BrdU - HPLC chromatogram

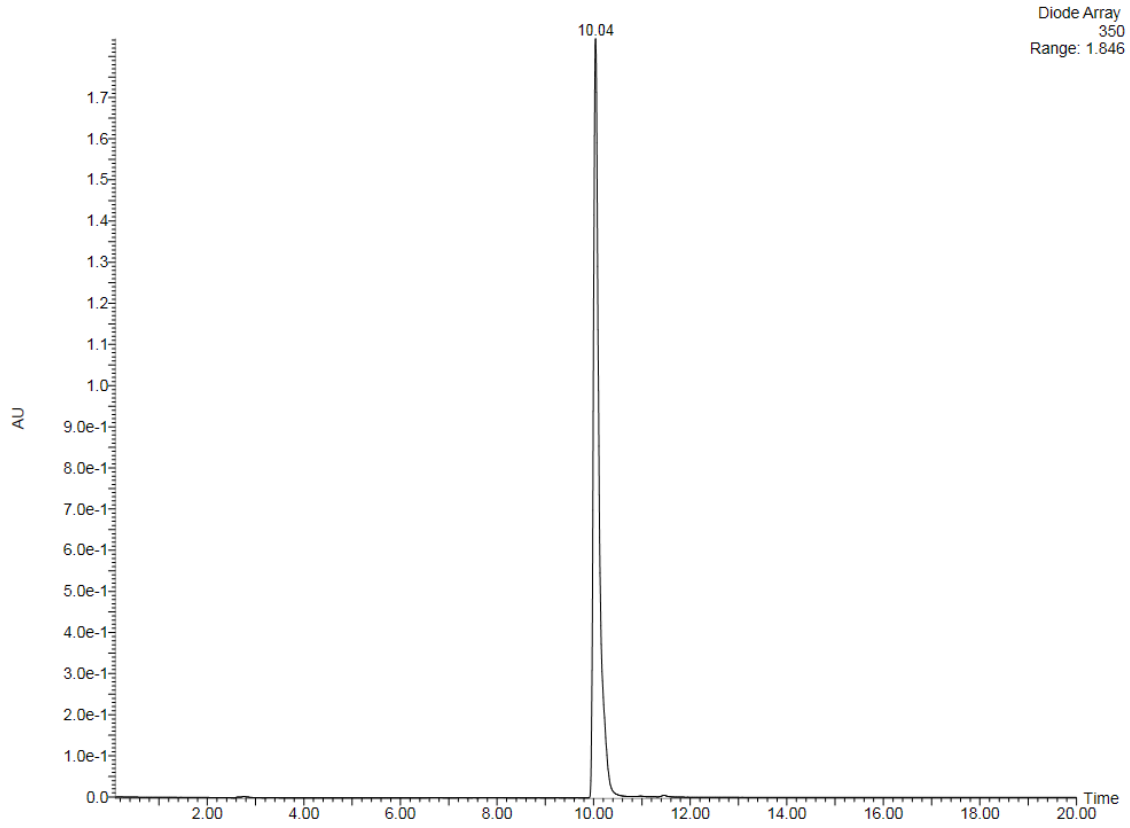

PDC-4,PEG-BrdU - HPLC chromatogram

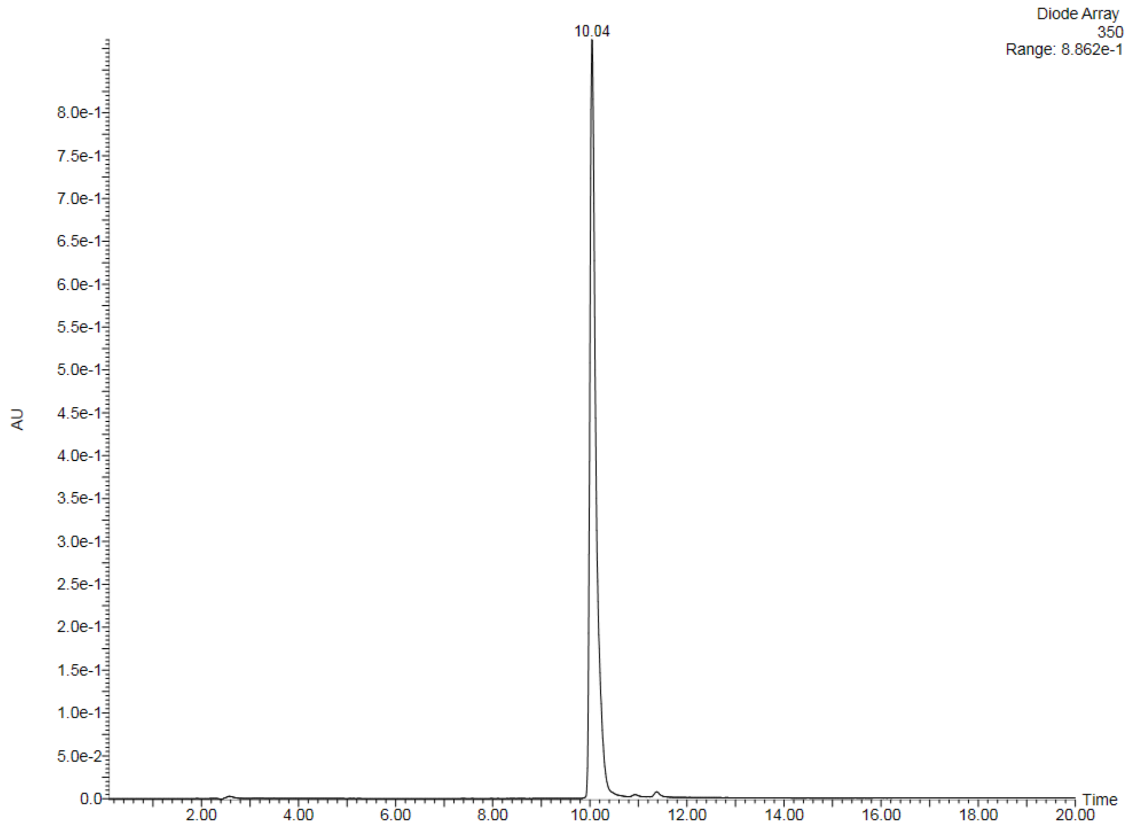

PDC-4,0-BrdU - HPLC chromatogram

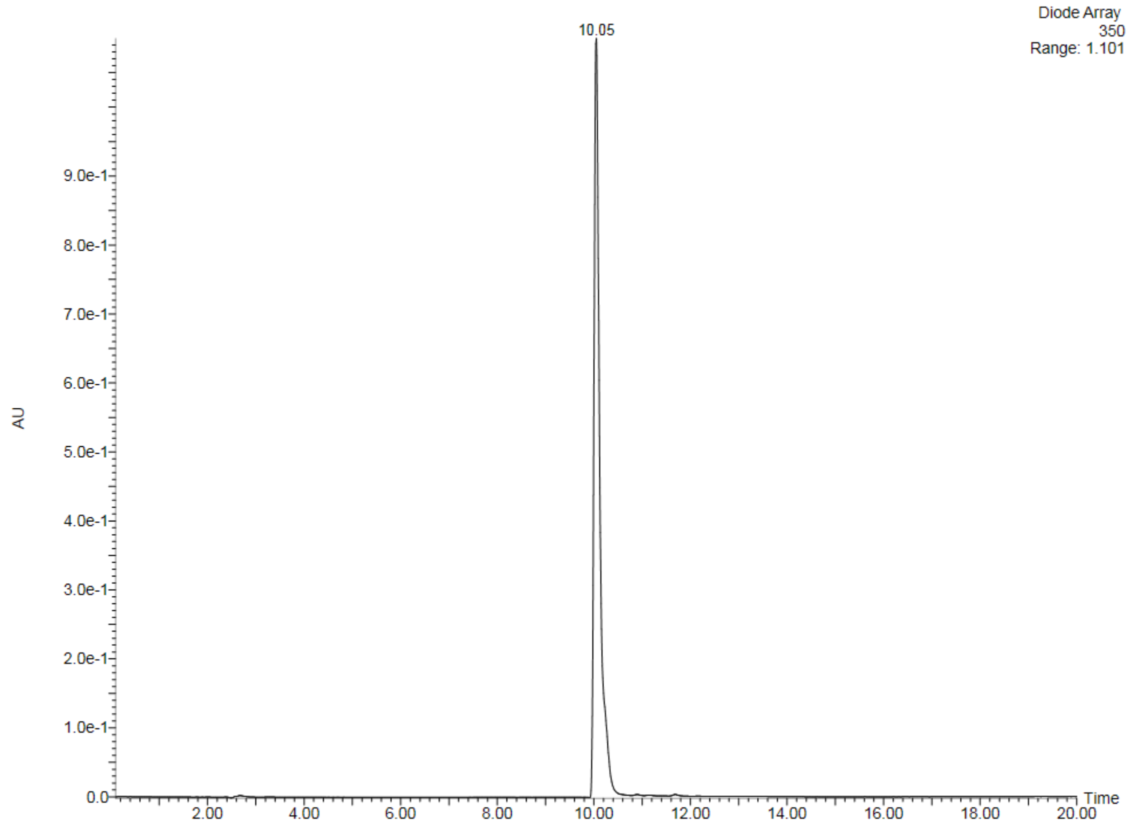

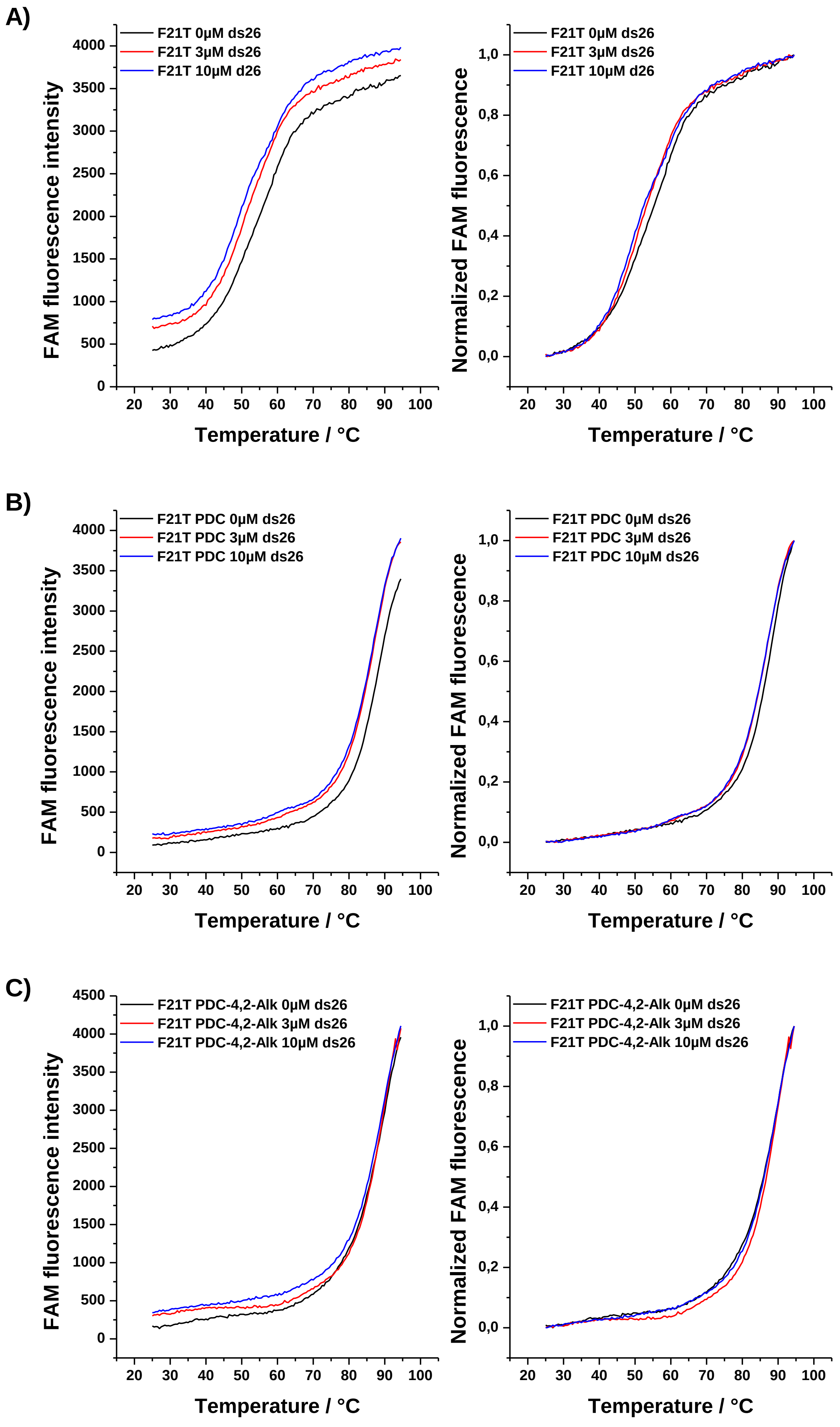
A)
F21T 0μM ds26
F21T 3μM ds26
F21T 10μM d26
FAM fluorescence intensity
Normalized FAM fluorescence
Temperature / °C
B)
F21T PDC 0μM ds26
F21T PDC 3μM ds26
F21T PDC 10μM ds26
FAM fluorescence intensity
Normalized FAM fluorescence
Temperature / °C
C)
F21T PDC-4,2-Alk 0μM ds26
F21T PDC-4,2-Alk 3μM ds26
F21T PDC-4,2-Alk 10μM ds26
FAM fluorescence intensity
Normalized FAM fluorescence
Temperature / °C

D)

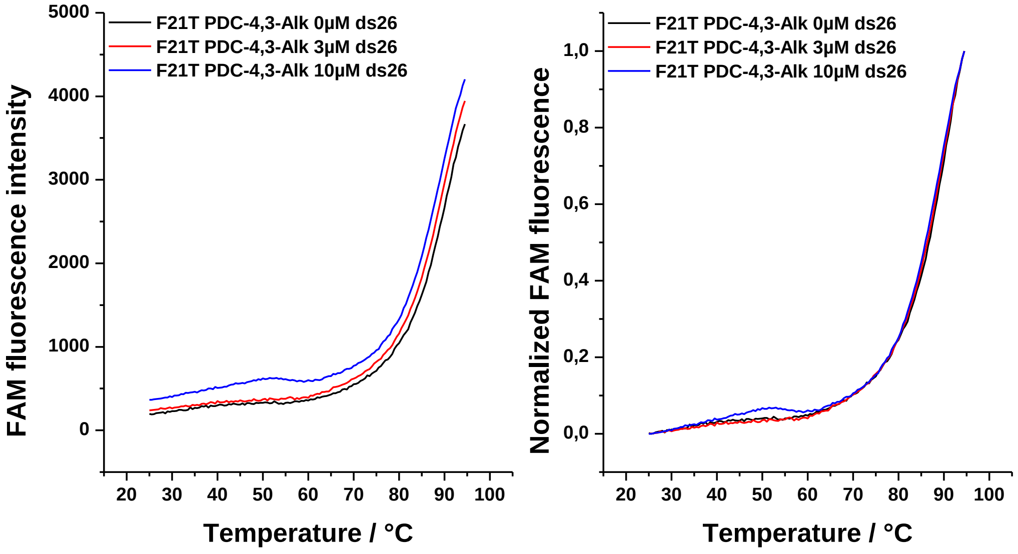
F21T PDC-4,3-Alk 0μM ds26
F21T PDC-4,3-Alk 3μM ds26
F21T PDC-4,3-Alk 10μM ds26
FAM fluorescence intensity
Temperature / °C
F21T PDC-4,3-Alk 0μM ds26
F21T PDC-4,3-Alk 3μM ds26
F21T PDC-4,3-Alk 10μM ds26
Normalized FAM fluorescence
Temperature / °C

E)

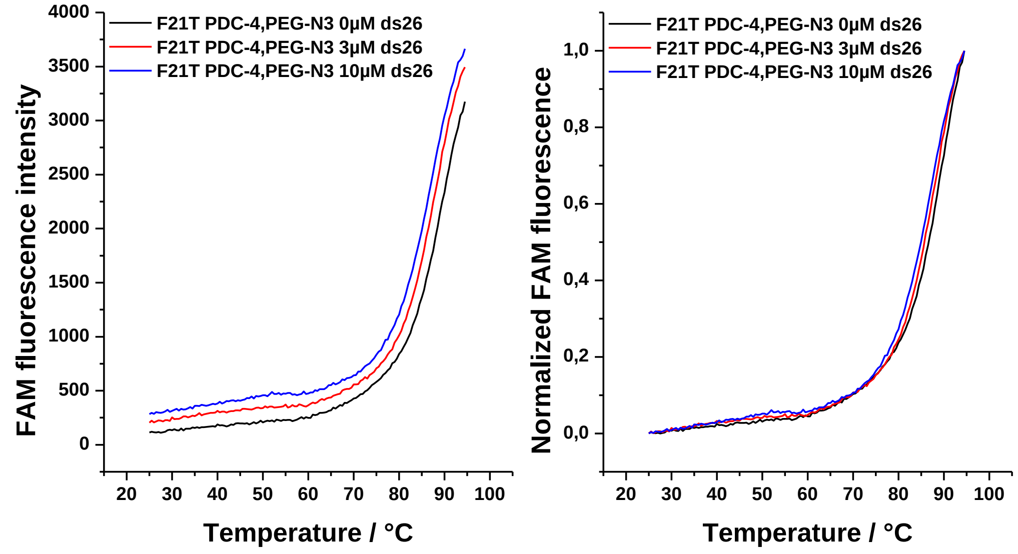
F21T PDC-4,PEG-N3 0μM ds26
F21T PDC-4,PEG-N3 3μM ds26
F21T PDC-4,PEG-N3 10μM ds26
FAM fluorescence intensity
Temperature / °C
F21T PDC-4,PEG-N3 0μM ds26
F21T PDC-4,PEG-N3 3μM ds26
F21T PDC-4,PEG-N3 10μM ds26
Normalized FAM fluorescence
Temperature / °C

F)

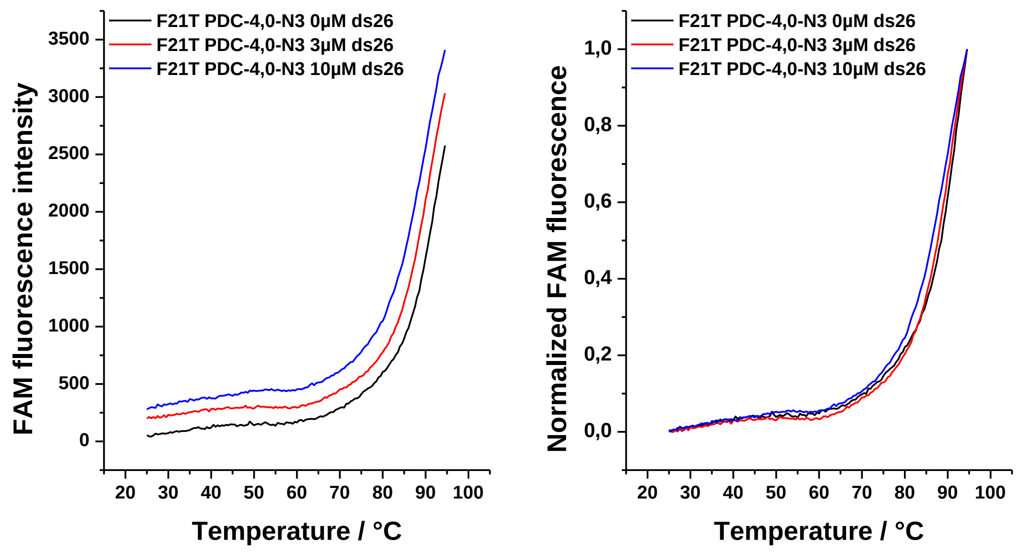
F21T PDC-4,0-N3 0μM ds26
F21T PDC-4,0-N3 3μM ds26
F21T PDC-4,0-N3 10μM ds26
FAM fluorescence intensity
Temperature / °C
F21T PDC-4,0-N3 0μM ds26
F21T PDC-4,0-N3 3μM ds26
F21T PDC-4,0-N3 10μM ds26
Normalized FAM fluorescence
Temperature / °C

G)

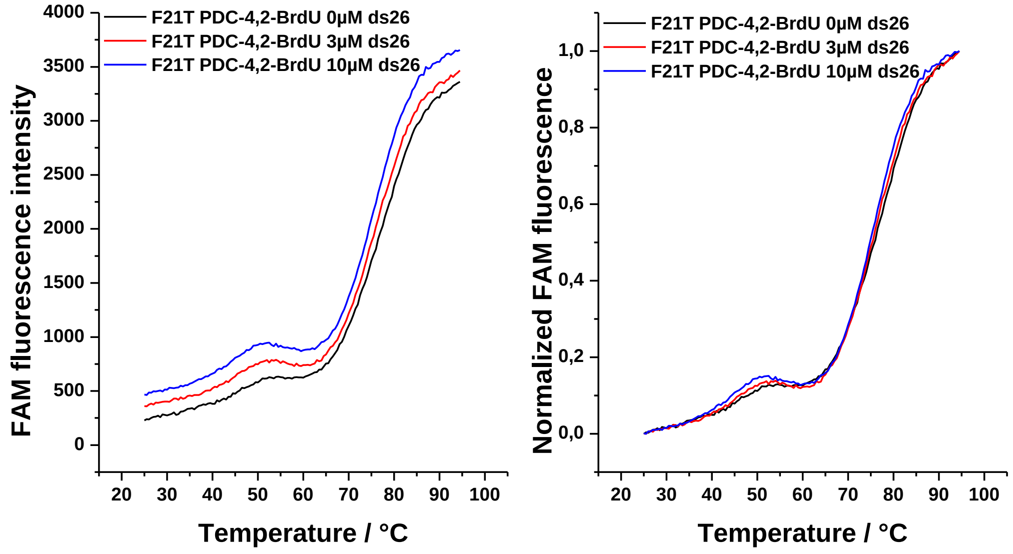
F21T PDC-4,2-BrdU 0µM ds26
F21T PDC-4,2-BrdU 3µM ds26
F21T PDC-4,2-BrdU 10µM ds26
FAM fluorescence intensity
Normalized FAM fluorescence
Temperature / °C
Temperature / °C

H)

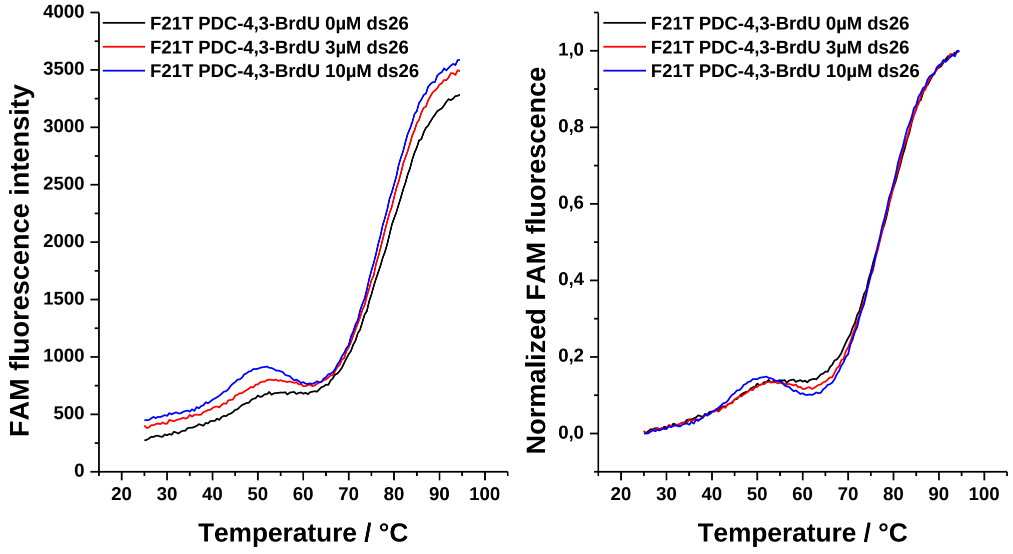
F21T PDC-4,3-BrdU 0µM ds26
F21T PDC-4,3-BrdU 3µM ds26
F21T PDC-4,3-BrdU 10µM ds26
FAM fluorescence intensity
Normalized FAM fluorescence
Temperature / °C
Temperature / °C

I)

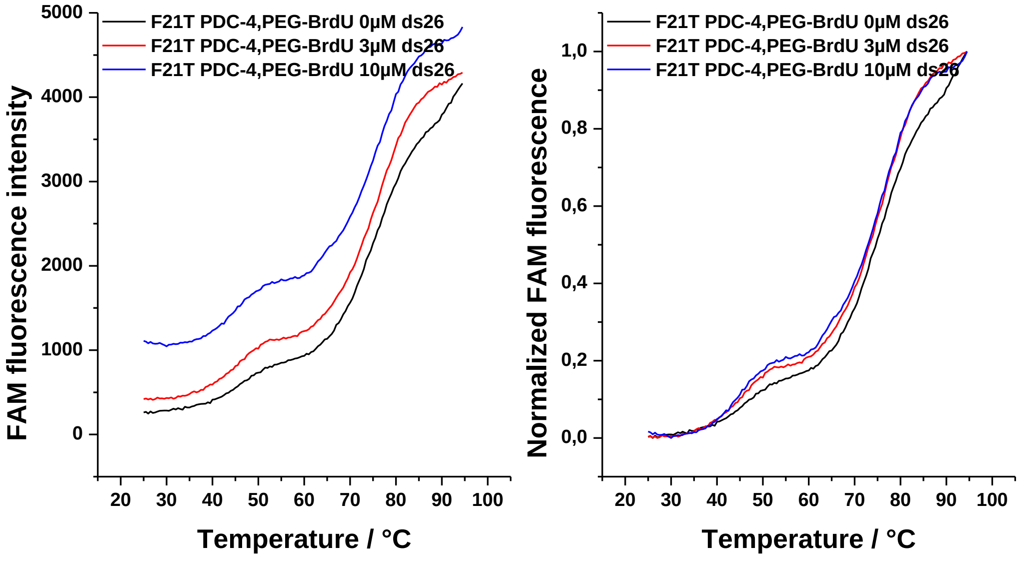
F21T PDC-4,PEG-BrdU 0µM ds26
F21T PDC-4,PEG-BrdU 3µM ds26
F21T PDC-4,PEG-BrdU 10µM ds26
FAM fluorescence intensity
Normalized FAM fluorescence
Temperature / °C
Temperature / °C

J)

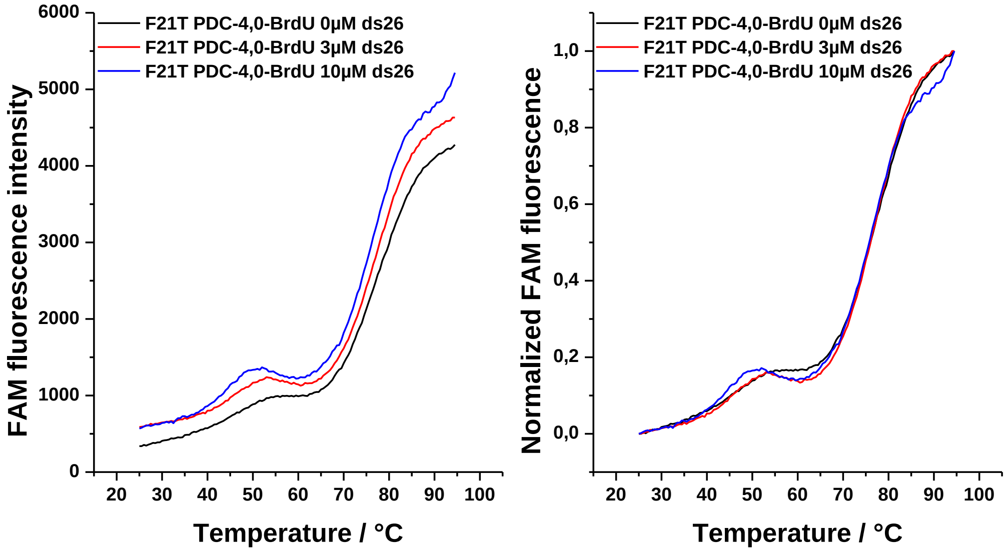

Figure S37: Examples of non-normalized and normalized FRET melting curves obtained for the human telomeric sequence F21T (0.2 μM). A) without ligand or with B) PDC (used as a reference), C) PDC-4,2-Alk, D) PDC-4,3-Alk, E) PDC-4,PEG-N3, F) PDC-4,0-N3, G) PDC-4,2-BrdU, H) PDC-4,3-BrdU, I) PDC-4,PEG-BrdU and J) PDC-4,0-BrdU (1.0 μM). FAM fluorescence (left) and normalized FAM fluorescence (right) are plotted as a function of the temperature. Experiments are carried out in 10 mM lithium cacodylate buffer (pH 7.2), 90 mM LiCl, and 10 mM KCl without (0 μM ds26, black curves) or with duplex competitor (3 μM ds26, red curves, and 10 μM ds26, blue curves) in a total volume of 25 μL.

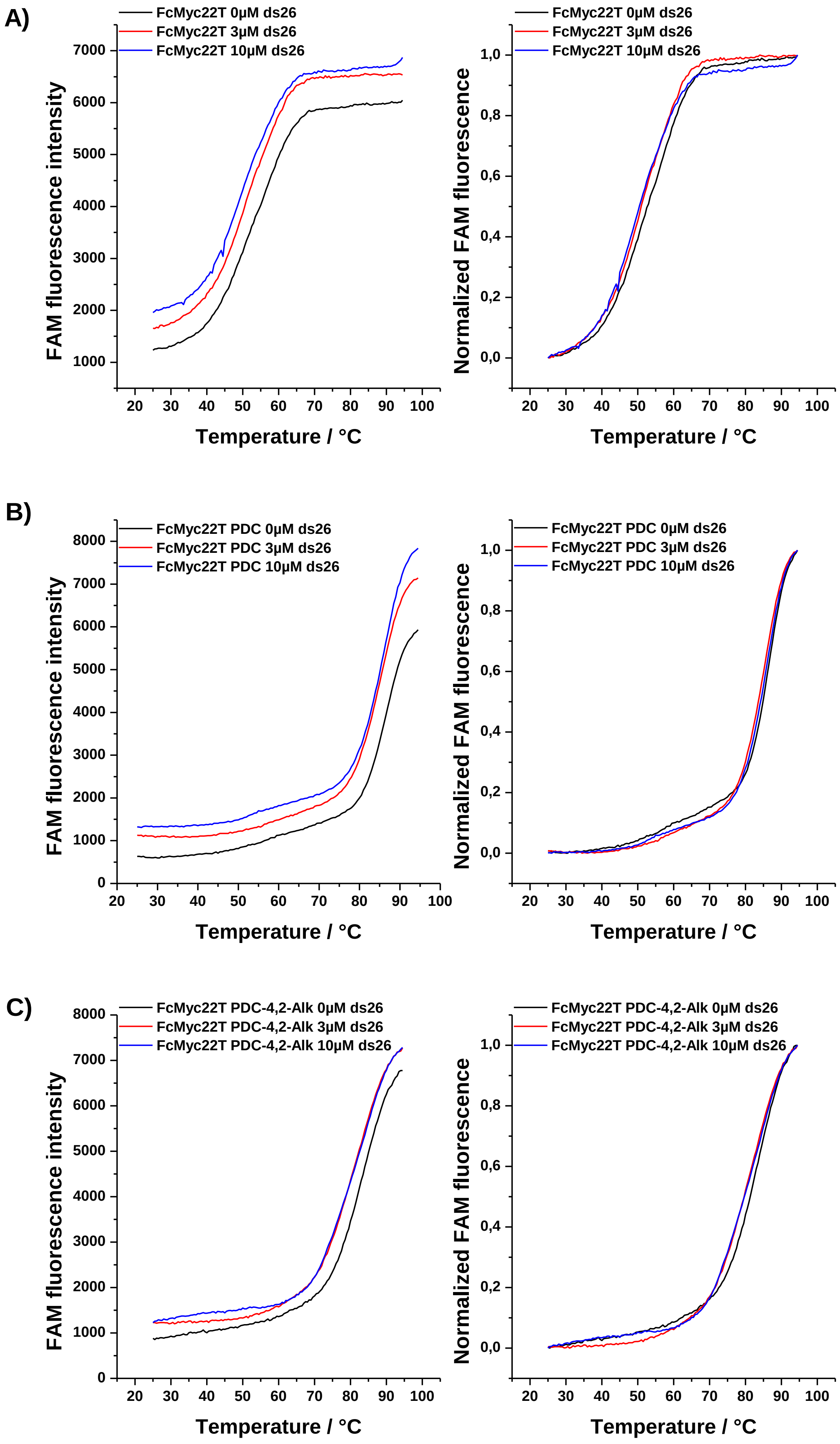
A)
FcMyc22T 0μM ds26
FcMyc22T 3μM ds26
FcMyc22T 10μM ds26
FAM fluorescence intensity
Normalized FAM fluorescence
Temperature / °C
B)
FcMyc22T PDC 0μM ds26
FcMyc22T PDC 3μM ds26
FcMyc22T PDC 10μM ds26
FAM fluorescence intensity
Normalized FAM fluorescence
Temperature / °C
C)
FcMyc22T PDC-4,2-Alk 0μM ds26
FcMyc22T PDC-4,2-Alk 3μM ds26
FcMyc22T PDC-4,2-Alk 10μM ds26
FAM fluorescence intensity
Normalized FAM fluorescence
Temperature / °C

D)

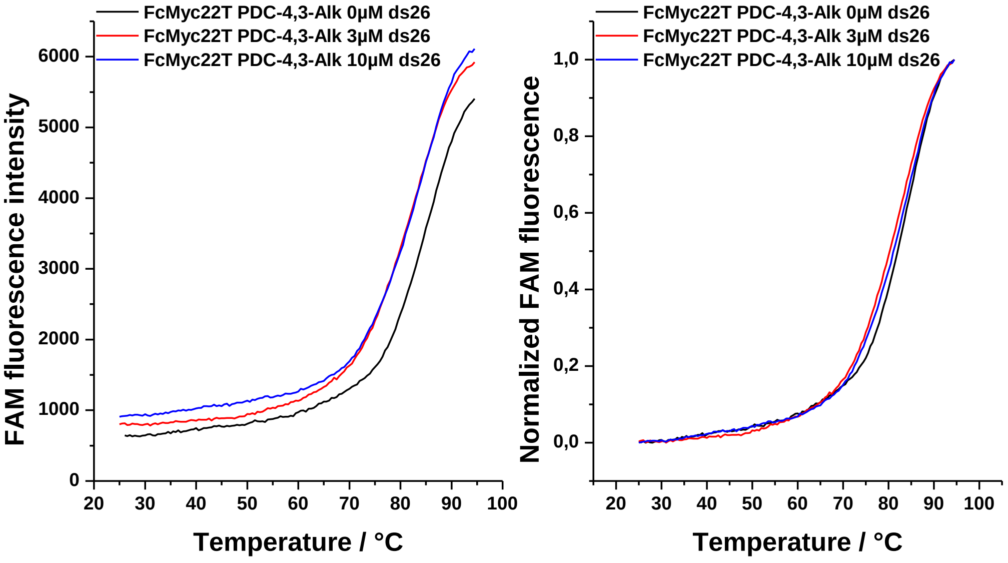
FcMyc22T PDC-4,3-Alk 0µM ds26
FcMyc22T PDC-4,3-Alk 3µM ds26
FcMyc22T PDC-4,3-Alk 10µM ds26
FAM fluorescence intensity
Temperature / °C
Normalized FAM fluorescence

E)

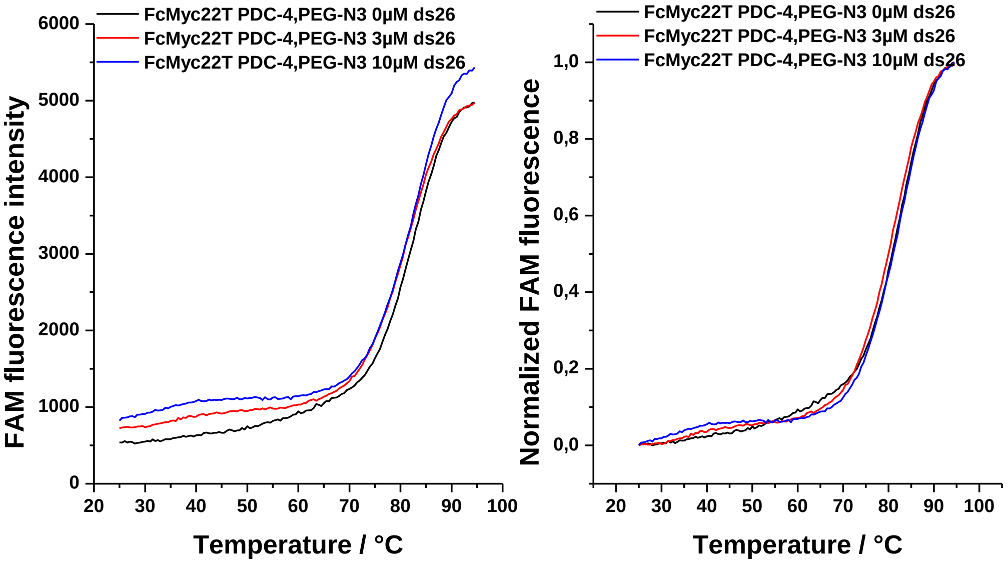
FcMyc22T PDC-4,PEG-N3 0µM ds26
FcMyc22T PDC-4,PEG-N3 3µM ds26
FcMyc22T PDC-4,PEG-N3 10µM ds26
FAM fluorescence intensity
Temperature / °C
Normalized FAM fluorescence

F)

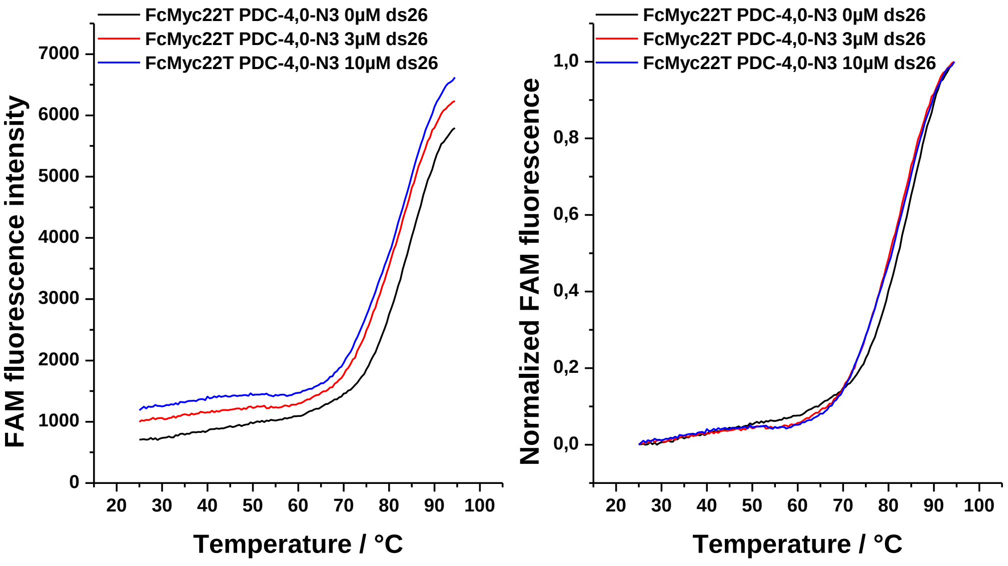
FcMyc22T PDC-4,0-N3 0µM ds26
FcMyc22T PDC-4,0-N3 3µM ds26
FcMyc22T PDC-4,0-N3 10µM ds26
FAM fluorescence intensity
Temperature / °C
Normalized FAM fluorescence

G)

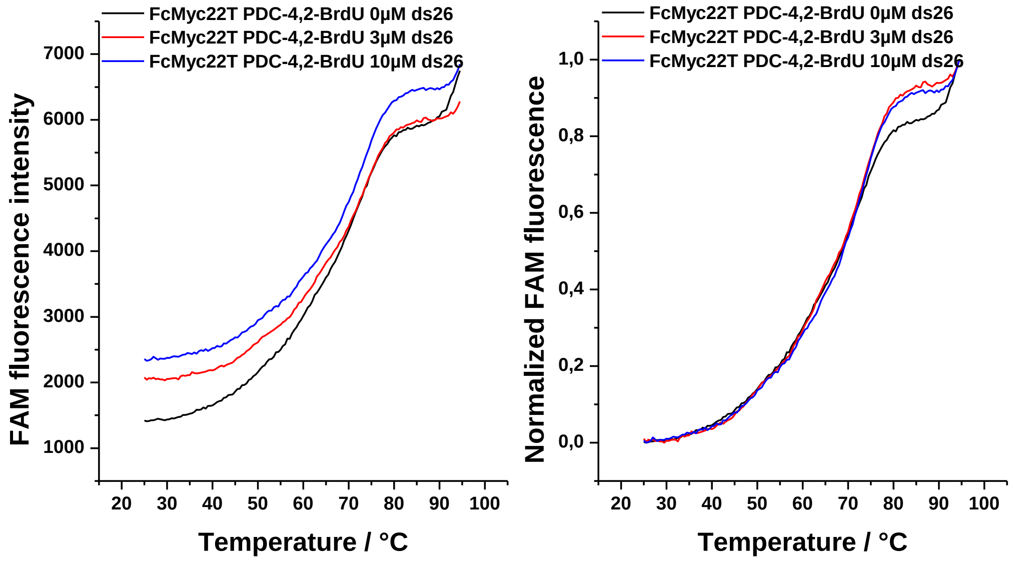
FcMyc22T PDC-4,2-BrdU 0µM ds26
FcMyc22T PDC-4,2-BrdU 3µM ds26
FcMyc22T PDC-4,2-BrdU 10µM ds26
FAM fluorescence intensity
Normalized FAM fluorescence
Temperature / °C

H)

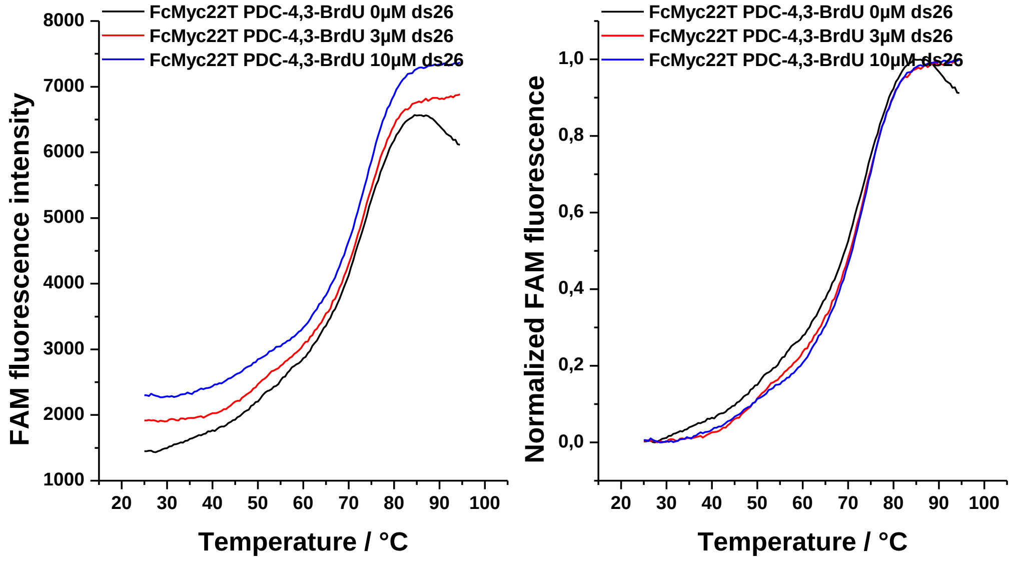
FcMyc22T PDC-4,3-BrdU 0µM ds26
FcMyc22T PDC-4,3-BrdU 3µM ds26
FcMyc22T PDC-4,3-BrdU 10µM ds26
FAM fluorescence intensity
Normalized FAM fluorescence
Temperature / °C

I)

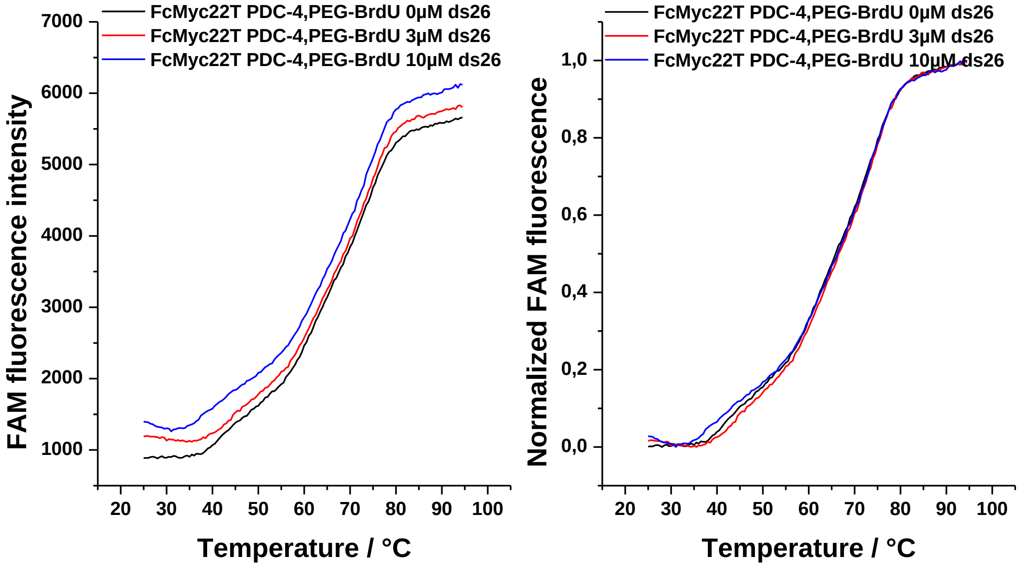
FcMyc22T PDC-4,PEG-BrdU 0µM ds26
FcMyc22T PDC-4,PEG-BrdU 3µM ds26
FcMyc22T PDC-4,PEG-BrdU 10µM ds26
FAM fluorescence intensity
Normalized FAM fluorescence
Temperature / °C

J)

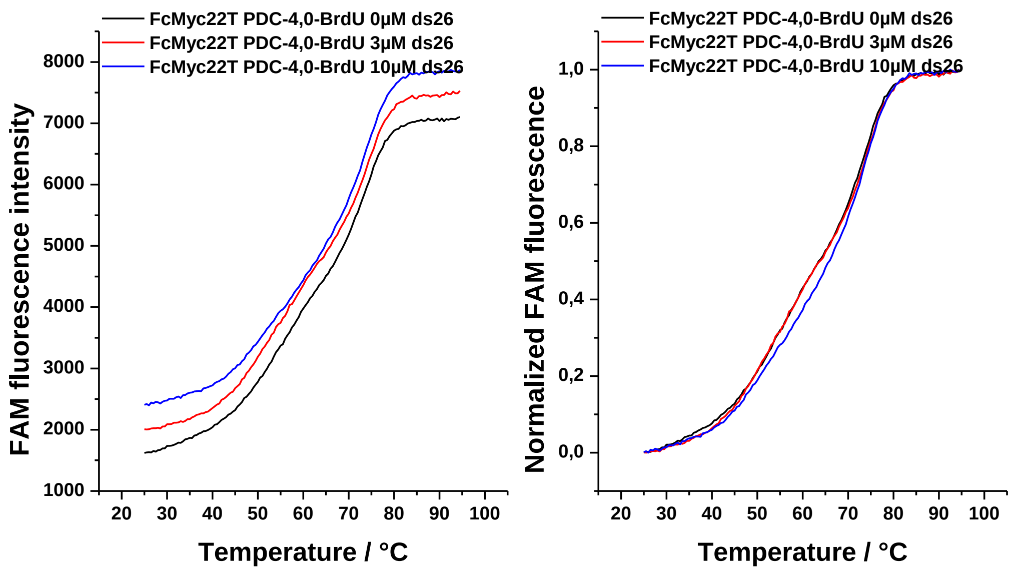

Figure S38: Examples of non-normalized and normalized FRET melting curves obtained for the c-Myc oncogene sequence FcMyc22T (0.2 µM). A) without ligand or with B) PDC (used as a reference), C) PDC-4,2-Alk, D) PDC-4,3-Alk, E) PDC-4,PEG-N3, F) PDC-4,0-N3, G) PDC-4,2-BrdU, H) PDC-4,3-BrdU, I) PDC-4,PEG-BrdU and J) PDC-4,0-BrdU (1.0 µM). FAM fluorescence (left) and normalized FAM fluorescence (right) are plotted as a function of the temperature. Experiments are carried out in 10 mM lithium cacodylate buffer (pH 7.2), 99 mM LiCl, and 1 mM KCl without (0 µM ds26, black curves) or with duplex competitor (3 µM ds26, red curves, and 10 µM ds26, blue curves) in a total volume of 25 µL.

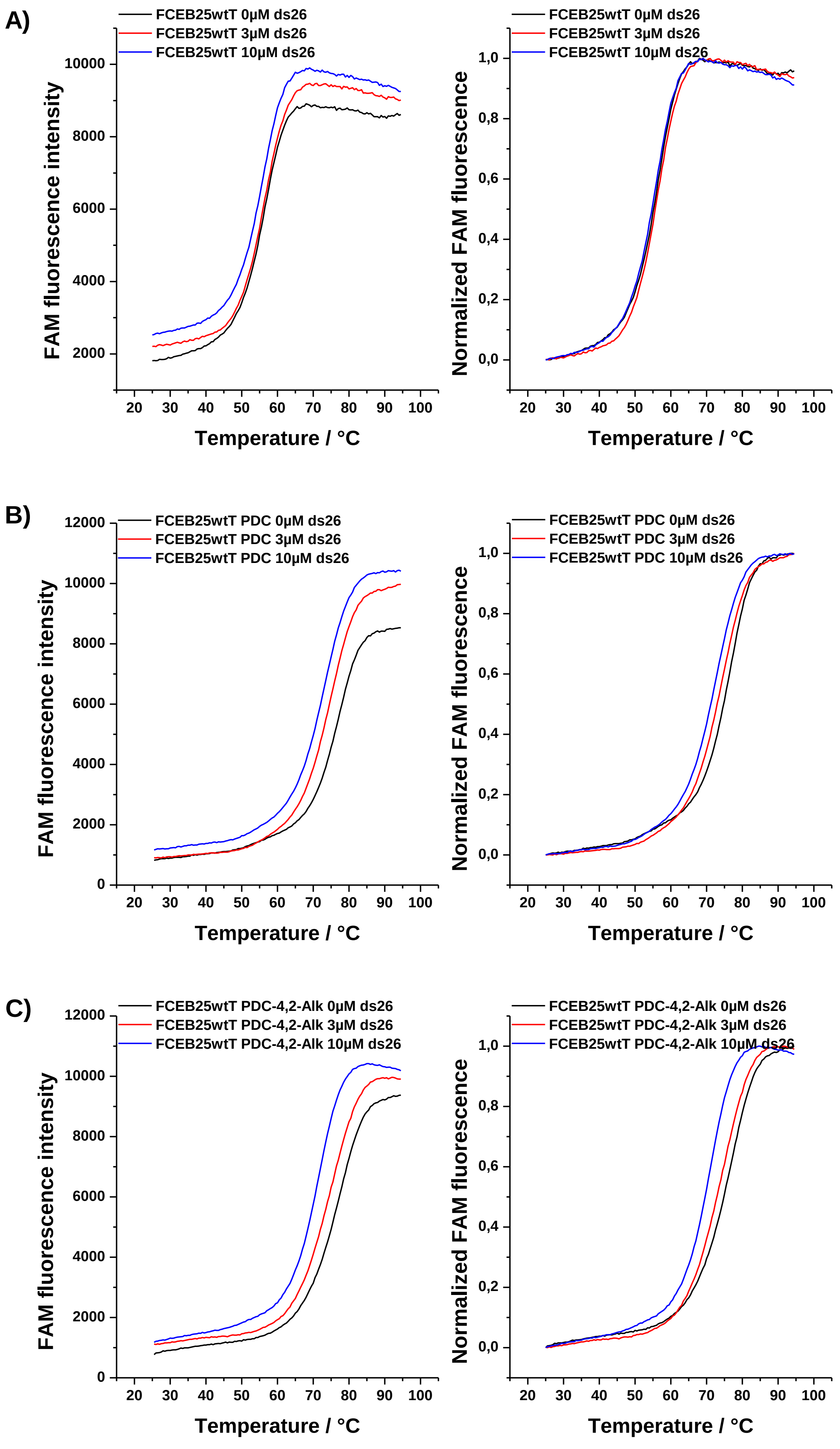
A)
FCEB25wtT 0μM ds26
FCEB25wtT 3μM ds26
FCEB25wtT 10μM ds26
FAM fluorescence intensity
Normalized FAM fluorescence
Temperature / °C
B)
FCEB25wtT PDC 0μM ds26
FCEB25wtT PDC 3μM ds26
FCEB25wtT PDC 10μM ds26
FAM fluorescence intensity
Normalized FAM fluorescence
Temperature / °C
C)
FCEB25wtT PDC-4,2-Alk 0μM ds26
FCEB25wtT PDC-4,2-Alk 3μM ds26
FCEB25wtT PDC-4,2-Alk 10μM ds26
FAM fluorescence intensity
Normalized FAM fluorescence
Temperature / °C

D)

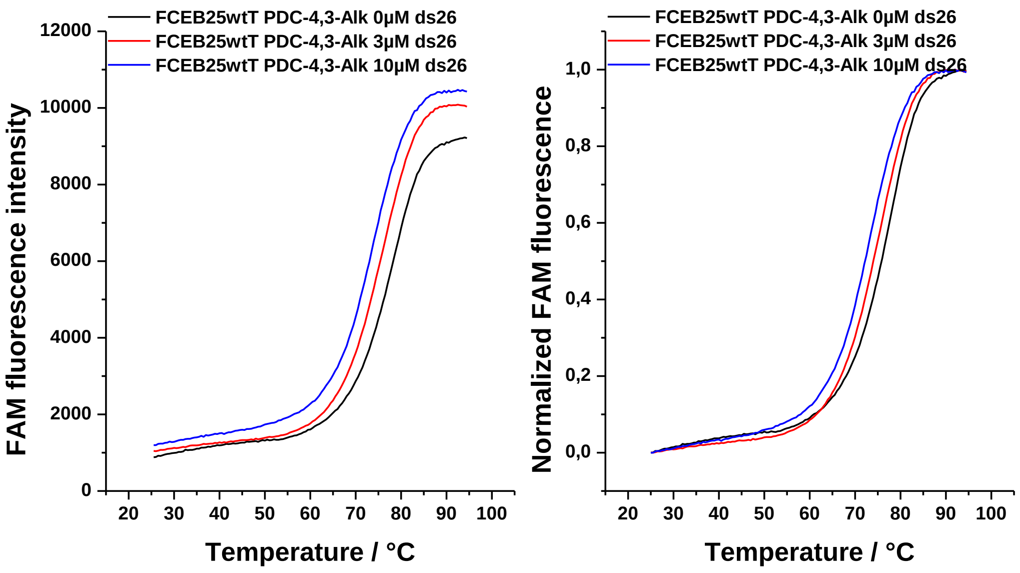
FCEB25wtT PDC-4,3-Alk 0µM ds26
FCEB25wtT PDC-4,3-Alk 3µM ds26
FCEB25wtT PDC-4,3-Alk 10µM ds26
FAM fluorescence intensity
Normalized FAM fluorescence
Temperature / °C

E)

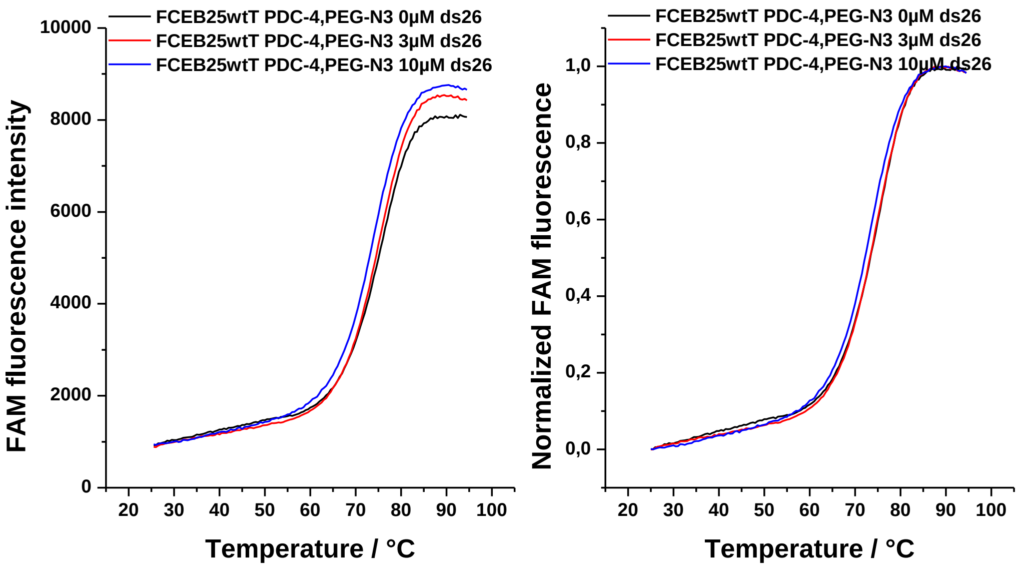
FCEB25wtT PDC-4,PEG-N3 0µM ds26
FCEB25wtT PDC-4,PEG-N3 3µM ds26
FCEB25wtT PDC-4,PEG-N3 10µM ds26
FAM fluorescence intensity
Normalized FAM fluorescence
Temperature / °C

F)

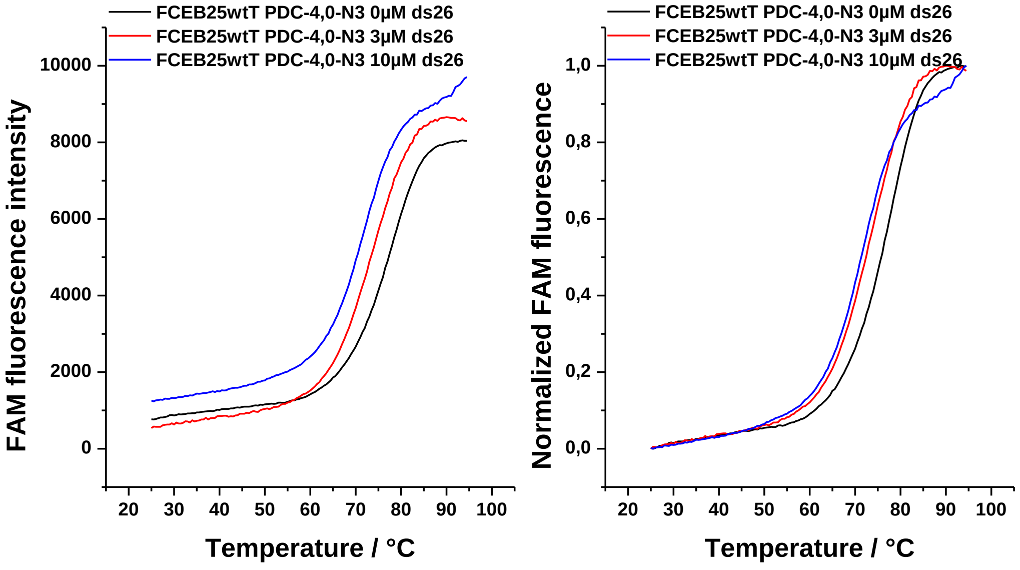
FCEB25wtT PDC-4,0-N3 0µM ds26
FCEB25wtT PDC-4,0-N3 3µM ds26
FCEB25wtT PDC-4,0-N3 10µM ds26
FAM fluorescence intensity
Normalized FAM fluorescence
Temperature / °C

G)

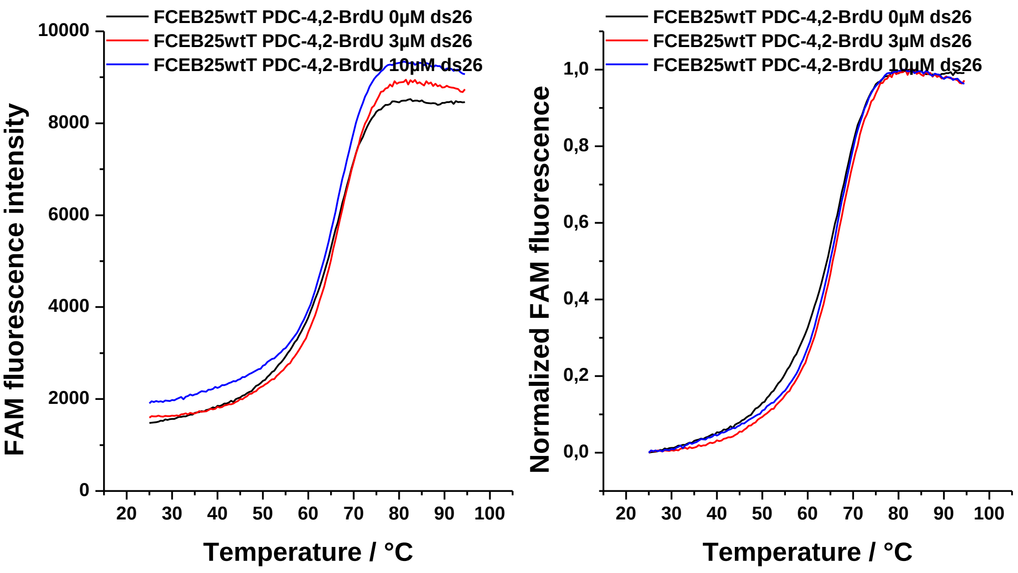
FCEB25wtT PDC-4,2-BrdU 0µM ds26
FCEB25wtT PDC-4,2-BrdU 3µM ds26
FCEB25wtT PDC-4,2-BrdU 10µM ds26
FAM fluorescence intensity
Normalized FAM fluorescence
Temperature / °C
Temperature / °C

H)

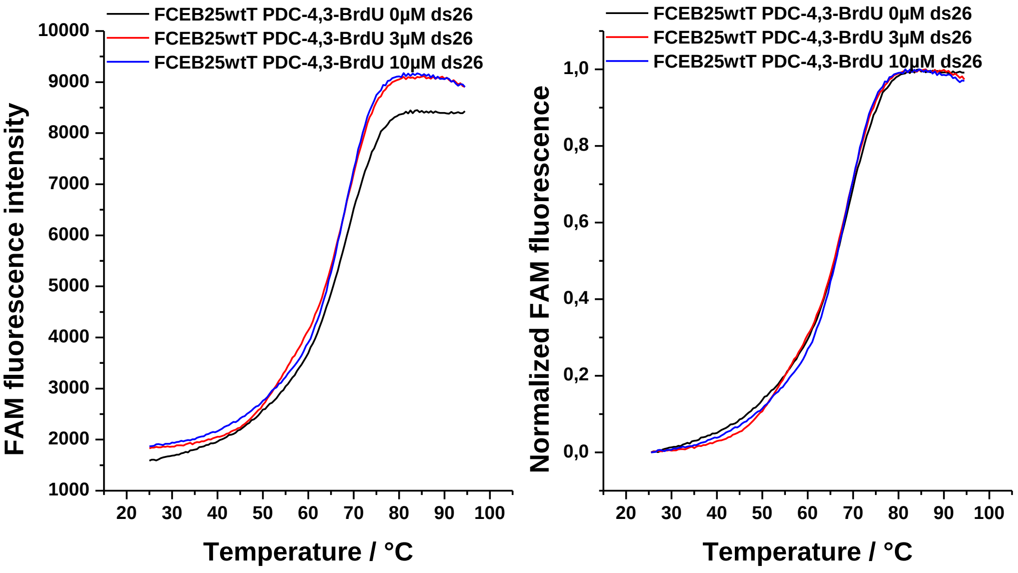
FCEB25wtT PDC-4,3-BrdU 0µM ds26
FCEB25wtT PDC-4,3-BrdU 3µM ds26
FCEB25wtT PDC-4,3-BrdU 10µM ds26
FAM fluorescence intensity
Normalized FAM fluorescence
Temperature / °C
Temperature / °C

I)

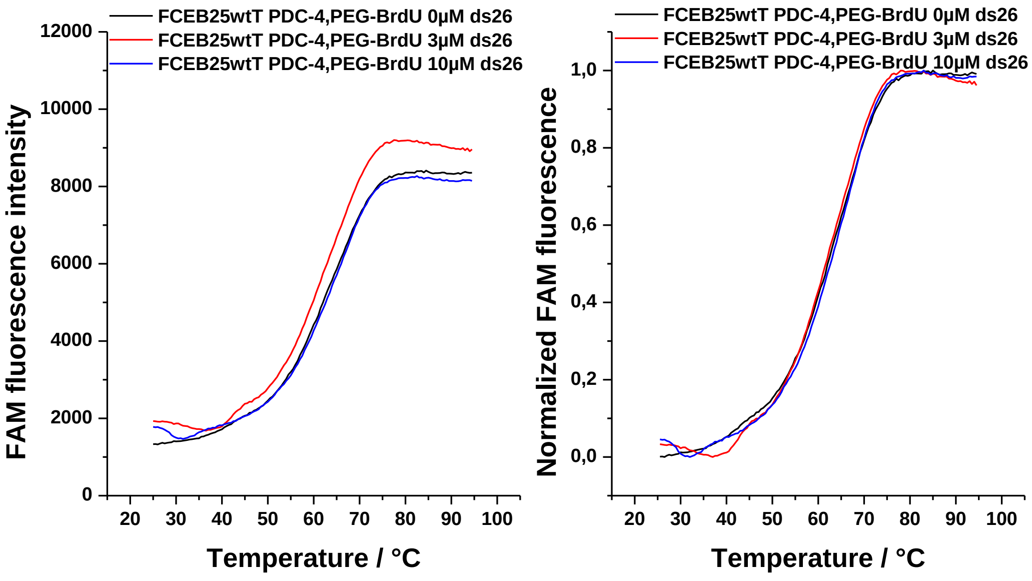
FCEB25wtT PDC-4,PEG-BrdU 0µM ds26
FCEB25wtT PDC-4,PEG-BrdU 3µM ds26
FCEB25wtT PDC-4,PEG-BrdU 10µM ds26
FAM fluorescence intensity
Normalized FAM fluorescence
Temperature / °C
Temperature / °C

J)

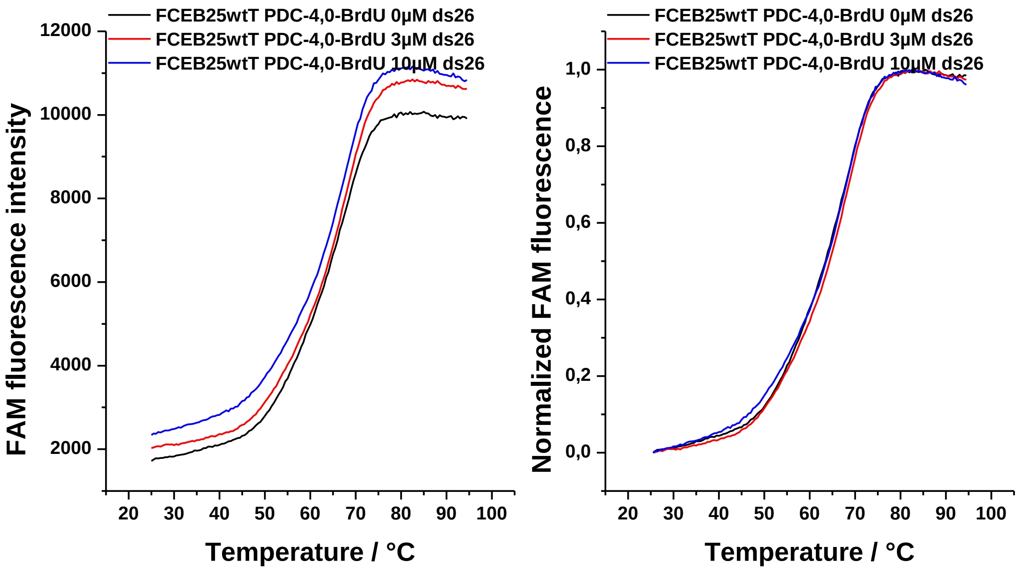

Figure S39: Examples of non-normalized and normalized FRET melting curves obtained for the human minisatellite native repeat sequence FCEB25wtT (0.2 µM). A) without ligand or with B) PDC (used as a reference), C) PDC-4,2-Alk, D) PDC-4,3-Alk, E) PDC-4,PEG-N3, F) PDC-4,0-N3, G) PDC-4,2-BrdU, H) PDC-4,3-BrdU, I) PDC-4,PEG-BrdU and J) PDC-4,0-BrdU (1.0 µM). FAM fluorescence (left) and normalized FAM fluorescence (right) are plotted as a function of the temperature. Experiments are carried out in 10 mM lithium cacodylate buffer (pH 7.2), 99 mM LiCl, and 1 mM KCl without (0 µM ds26, black curves) or with duplex competitor (3 µM ds26, red curves, and 10 µM ds26, blue curves) in a total volume of 25 µL.

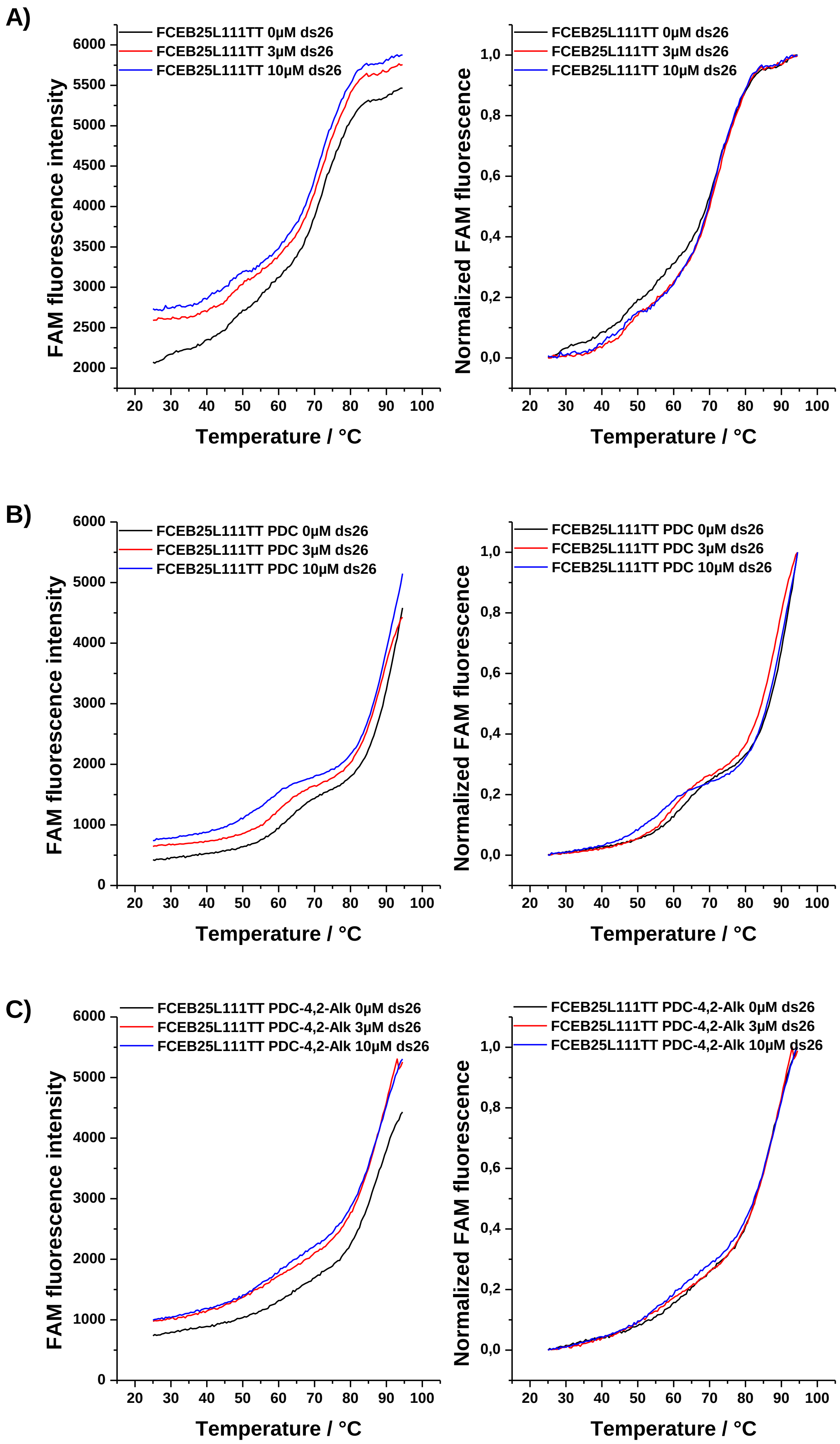
A)
FCEB25L111TT 0µM ds26
FCEB25L111TT 3µM ds26
FCEB25L111TT 10µM ds26
FAM fluorescence intensity
Normalized FAM fluorescence
Temperature / °C
B)
FCEB25L111TT PDC 0µM ds26
FCEB25L111TT PDC 3µM ds26
FCEB25L111TT PDC 10µM ds26
FAM fluorescence intensity
Normalized FAM fluorescence
Temperature / °C
C)
FCEB25L111TT PDC-4,2-Alk 0µM ds26
FCEB25L111TT PDC-4,2-Alk 3µM ds26
FCEB25L111TT PDC-4,2-Alk 10µM ds26
FAM fluorescence intensity
Normalized FAM fluorescence
Temperature / °C

D)

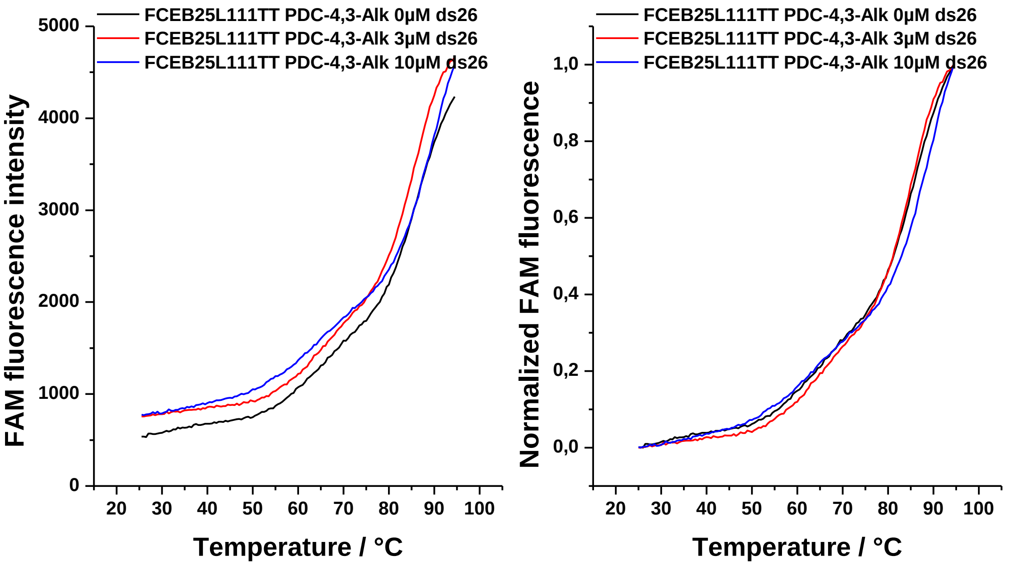
FCEB25L111TT PDC-4,3-Alk 0µM ds26
FCEB25L111TT PDC-4,3-Alk 3µM ds26
FCEB25L111TT PDC-4,3-Alk 10µM ds26
FAM fluorescence intensity
Temperature / °C
Normalized FAM fluorescence
Temperature / °C

E)

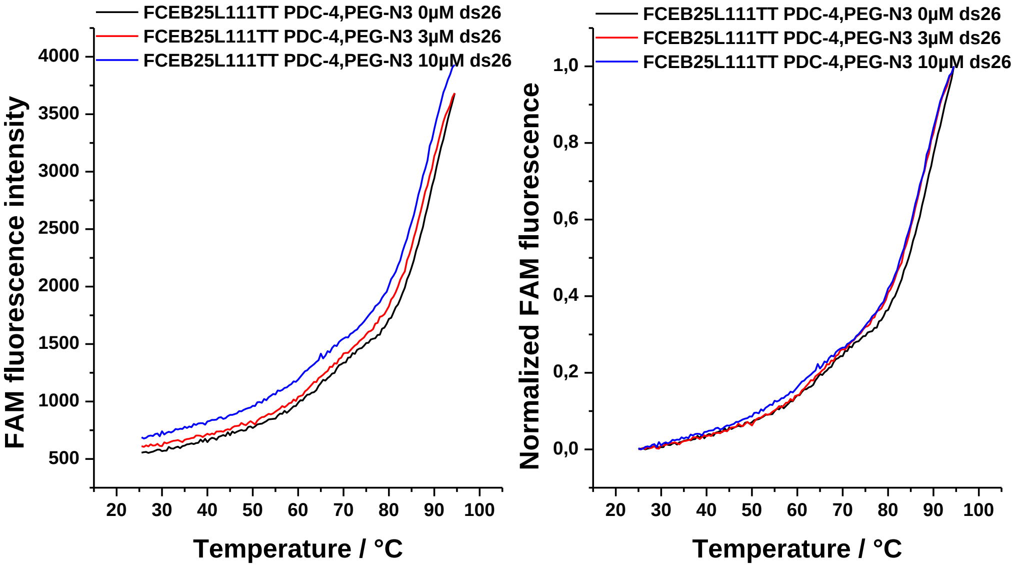
FCEB25L111TT PDC-4,PEG-N3 0µM ds26
FCEB25L111TT PDC-4,PEG-N3 3µM ds26
FCEB25L111TT PDC-4,PEG-N3 10µM ds26
FAM fluorescence intensity
Temperature / °C
Normalized FAM fluorescence
Temperature / °C

F)

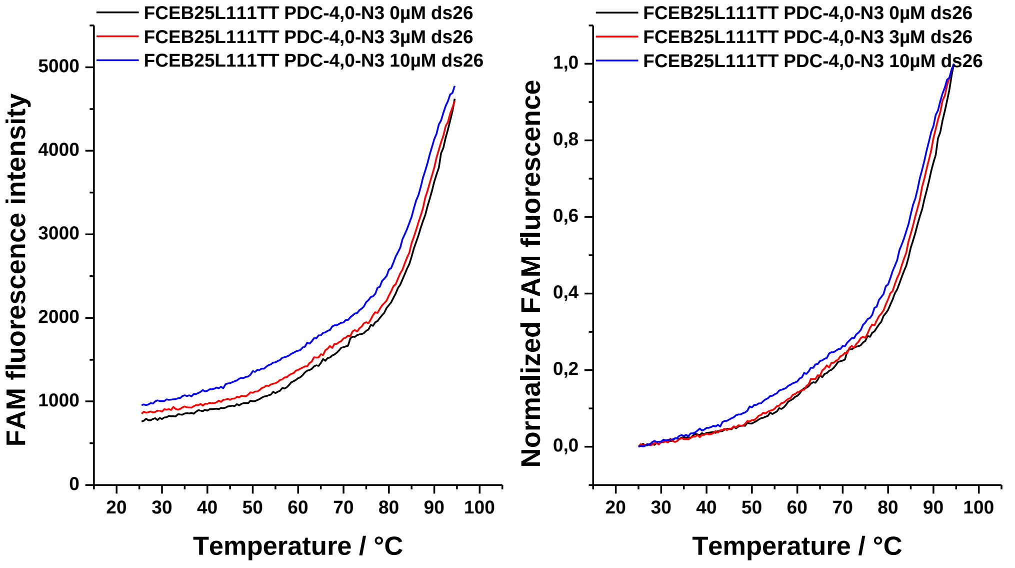
FCEB25L111TT PDC-4,0-N3 0µM ds26
FCEB25L111TT PDC-4,0-N3 3µM ds26
FCEB25L111TT PDC-4,0-N3 10µM ds26
FAM fluorescence intensity
Temperature / °C
Normalized FAM fluorescence
Temperature / °C

G)

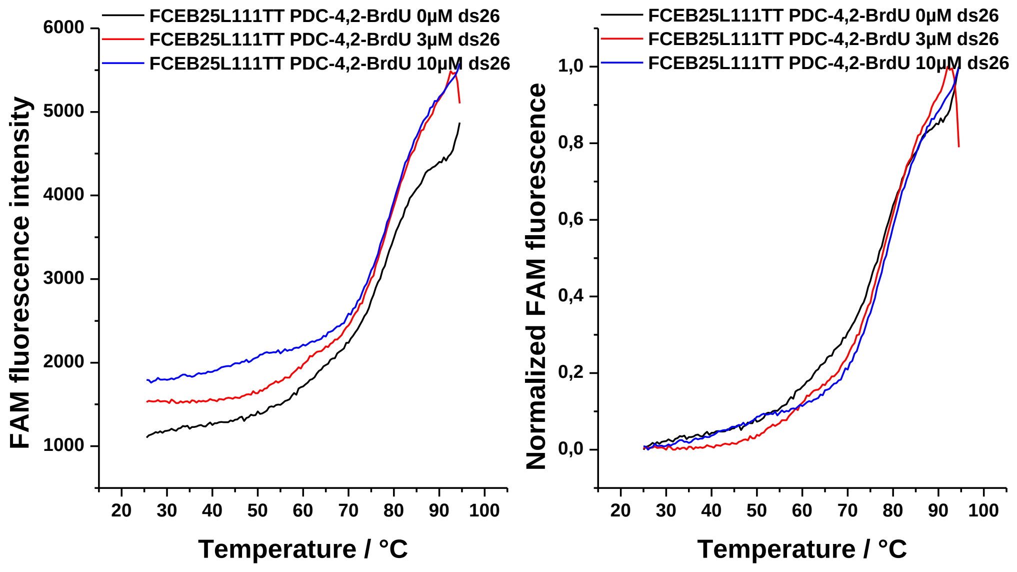
FCEB25L111TT PDC-4,2-BrdU 0µM ds26
FCEB25L111TT PDC-4,2-BrdU 3µM ds26
FCEB25L111TT PDC-4,2-BrdU 10µM ds26
FAM fluorescence intensity
Normalized FAM fluorescence
Temperature / °C

H)

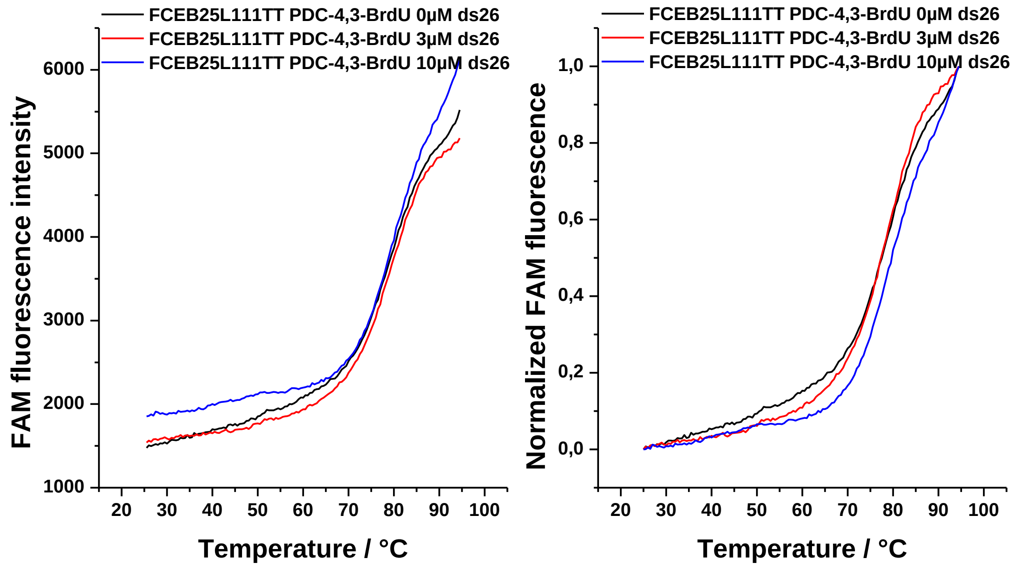
FCEB25L111TT PDC-4,3-BrdU 0µM ds26
FCEB25L111TT PDC-4,3-BrdU 3µM ds26
FCEB25L111TT PDC-4,3-BrdU 10µM ds26
FAM fluorescence intensity
Normalized FAM fluorescence
Temperature / °C

I)

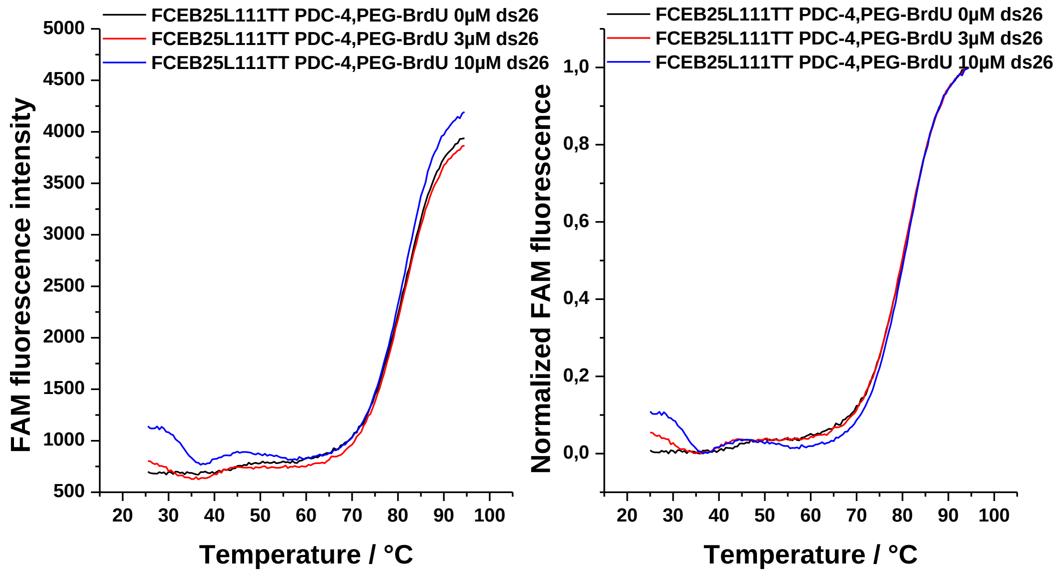
FCEB25L111TT PDC-4,PEG-BrdU 0µM ds26
FCEB25L111TT PDC-4,PEG-BrdU 3µM ds26
FCEB25L111TT PDC-4,PEG-BrdU 10µM ds26
FAM fluorescence intensity
Normalized FAM fluorescence
Temperature / °C

J)

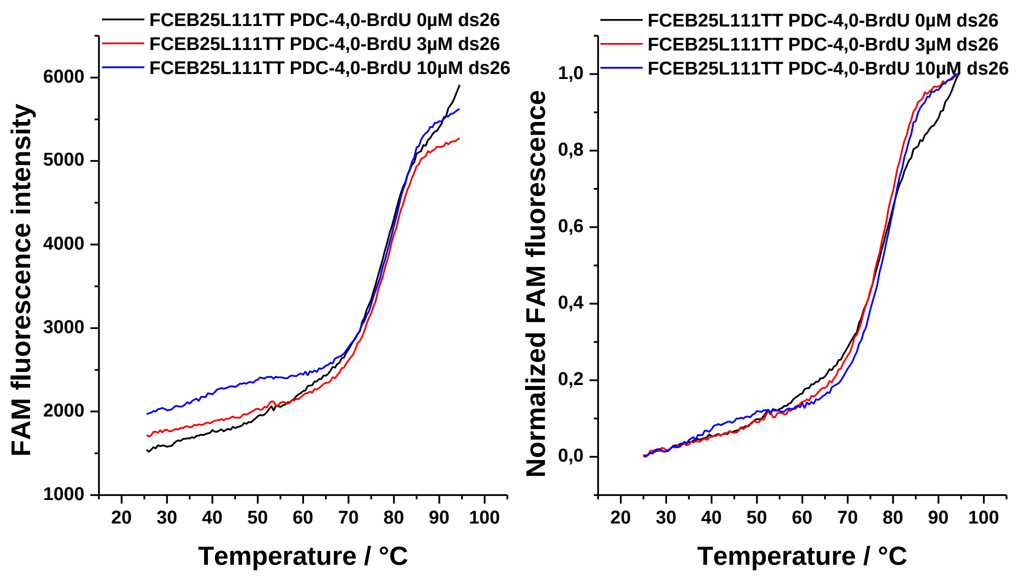

Figure S40: Examples of non-normalized and normalized FRET melting curves obtained for the human minisatellite repeat modified sequence FCEB25L111TT (0.2 µM). A) without ligand or with B) PDC (used as a reference), C) PDC-4,2-Alk, D) PDC-4,3-Alk, E) PDC-4,PEG-N3, F) PDC-4,0-N3, G) PDC-4,2-BrdU, H) PDC-4,3-BrdU, I) PDC-4,PEG-BrdU and J) PDC-4,0-BrdU (1.0 µM). FAM fluorescence (left) and normalized FAM fluorescence (right) are plotted as a function of the temperature. Experiments are carried out in 10 mM lithium cacodylate buffer (pH 7.2), 99 mM LiCl, and 1 mM KCl without (0 µM ds26, black curves) or with duplex competitor (3 µM ds26, red curves, and 10 µM ds26, blue curves) in a total volume of 25 µL.

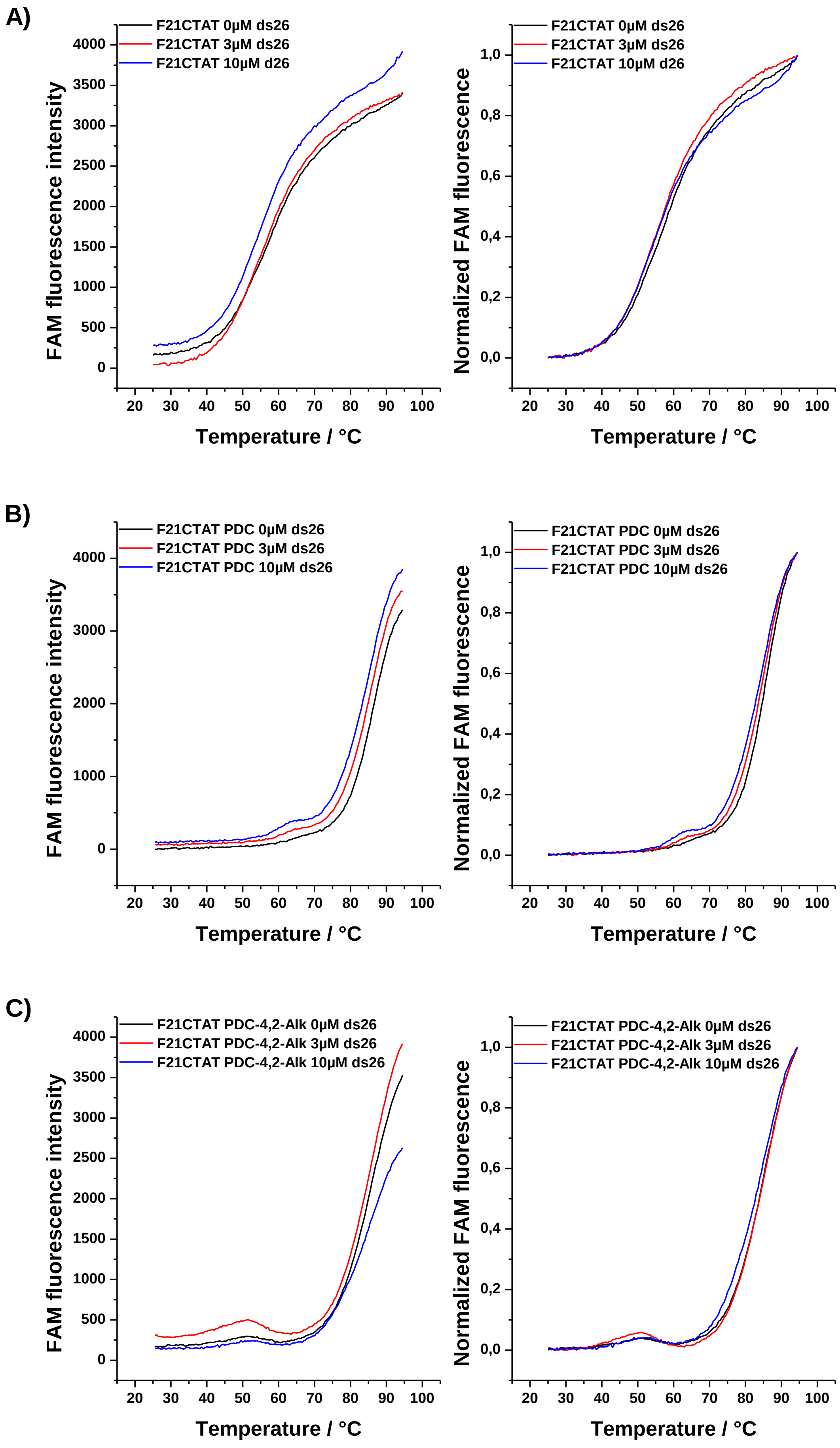
A)
F21CTAT 0µM ds26
F21CTAT 3µM ds26
F21CTAT 10µM d26
FAM fluorescence intensity
Normalized FAM fluorescence
Temperature / °C
B)
F21CTAT PDC 0µM ds26
F21CTAT PDC 3µM ds26
F21CTAT PDC 10µM ds26
FAM fluorescence intensity
Normalized FAM fluorescence
Temperature / °C
C)
F21CTAT PDC-4,2-Alk 0µM ds26
F21CTAT PDC-4,2-Alk 3µM ds26
F21CTAT PDC-4,2-Alk 10µM ds26
FAM fluorescence intensity
Normalized FAM fluorescence
Temperature / °C

D)

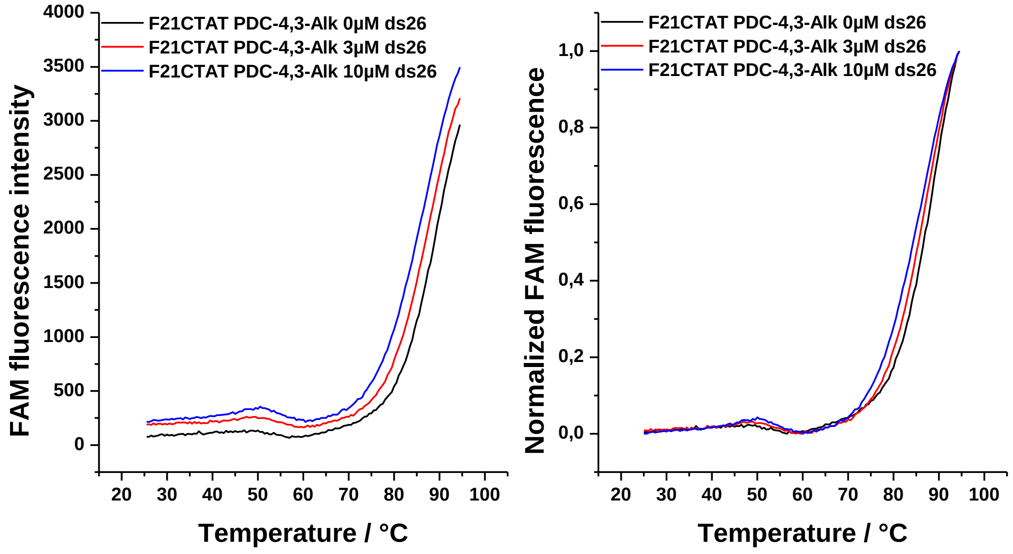
F21CTAT PDC-4,3-Alk 0μM ds26
F21CTAT PDC-4,3-Alk 3μM ds26
F21CTAT PDC-4,3-Alk 10μM ds26
FAM fluorescence intensity
Normalized FAM fluorescence
Temperature / °C

E)

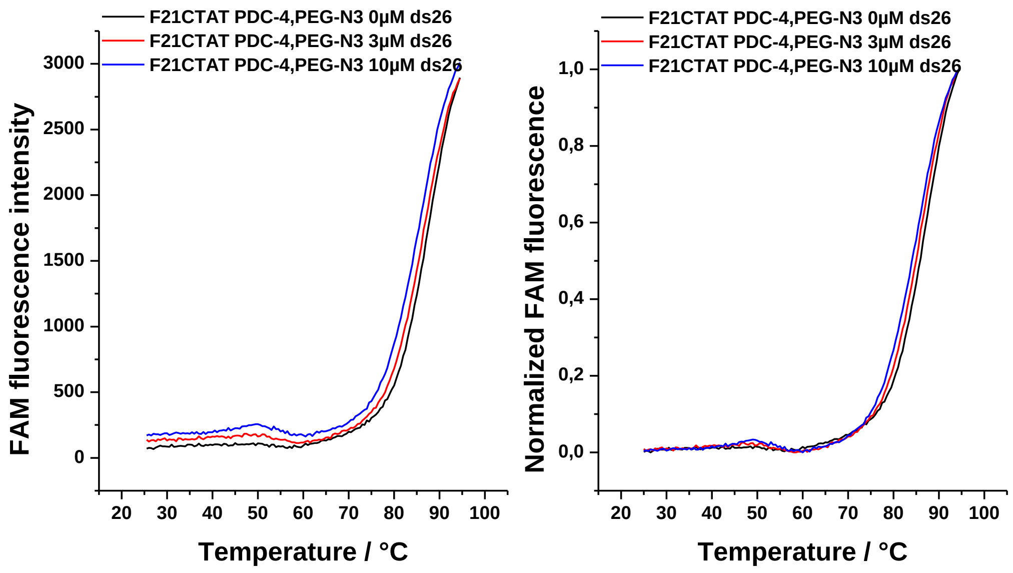
F21CTAT PDC-4,PEG-N3 0μM ds26
F21CTAT PDC-4,PEG-N3 3μM ds26
F21CTAT PDC-4,PEG-N3 10μM ds26
FAM fluorescence intensity
Normalized FAM fluorescence
Temperature / °C

F)

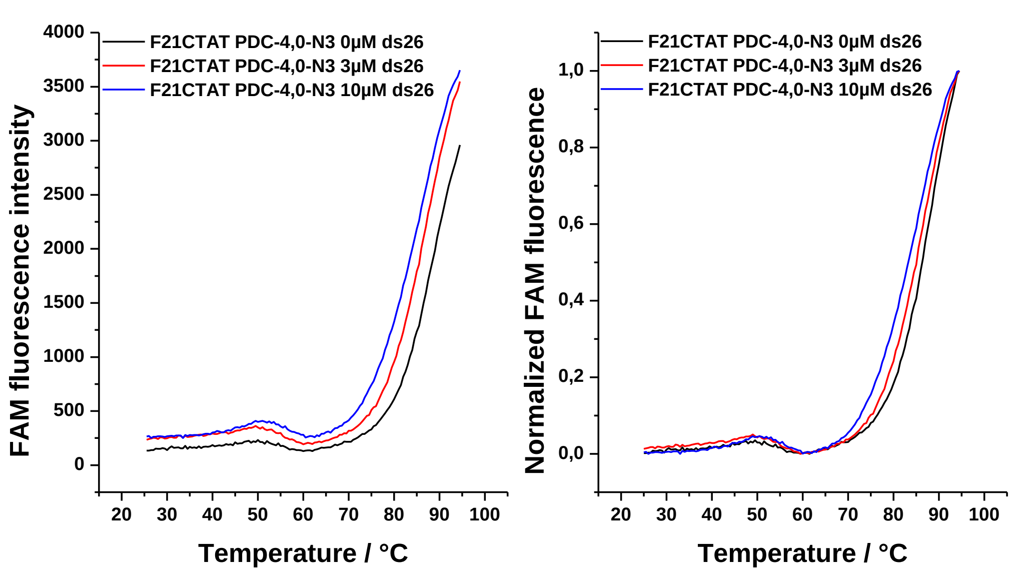
F21CTAT PDC-4,0-N3 0μM ds26
F21CTAT PDC-4,0-N3 3μM ds26
F21CTAT PDC-4,0-N3 10μM ds26
FAM fluorescence intensity
Normalized FAM fluorescence
Temperature / °C

G)

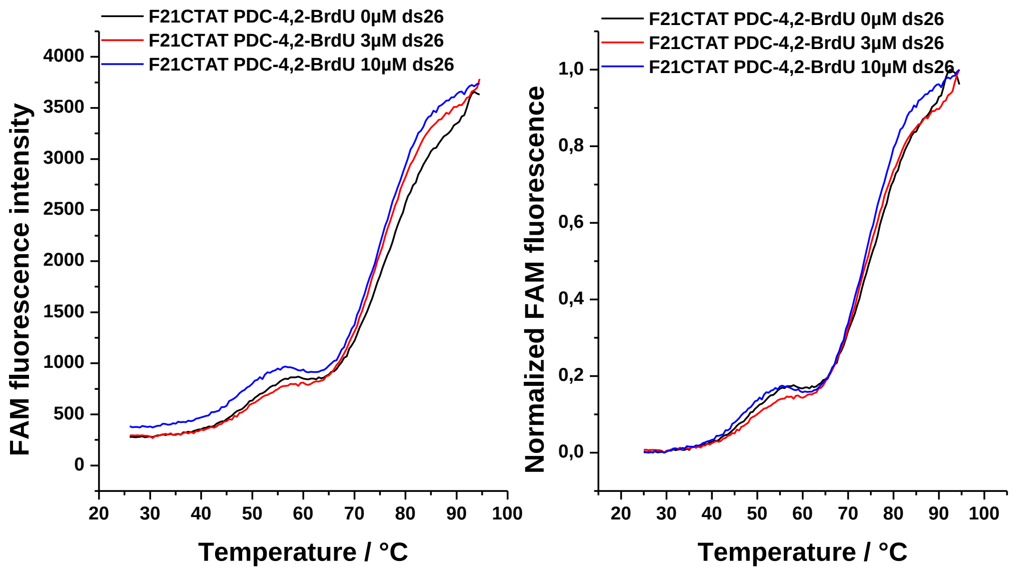
F21CTAT PDC-4,2-BrdU 0µM ds26
F21CTAT PDC-4,2-BrdU 3µM ds26
F21CTAT PDC-4,2-BrdU 10µM ds26
FAM fluorescence intensity
Temperature / °C
Normalized FAM fluorescence
Temperature / °C

H)

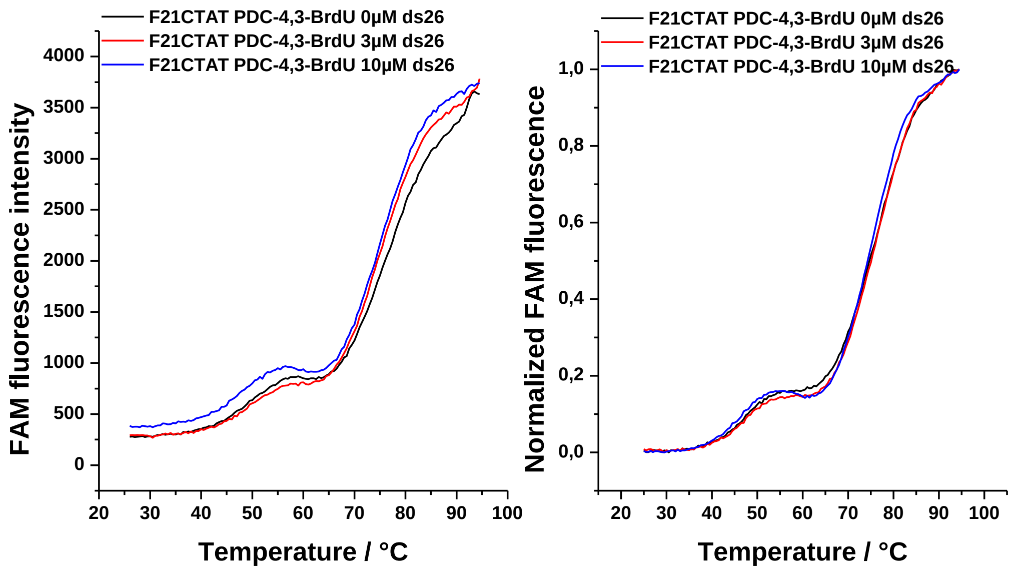
F21CTAT PDC-4,3-BrdU 0µM ds26
F21CTAT PDC-4,3-BrdU 3µM ds26
F21CTAT PDC-4,3-BrdU 10µM ds26
FAM fluorescence intensity
Temperature / °C
Normalized FAM fluorescence
Temperature / °C

I)

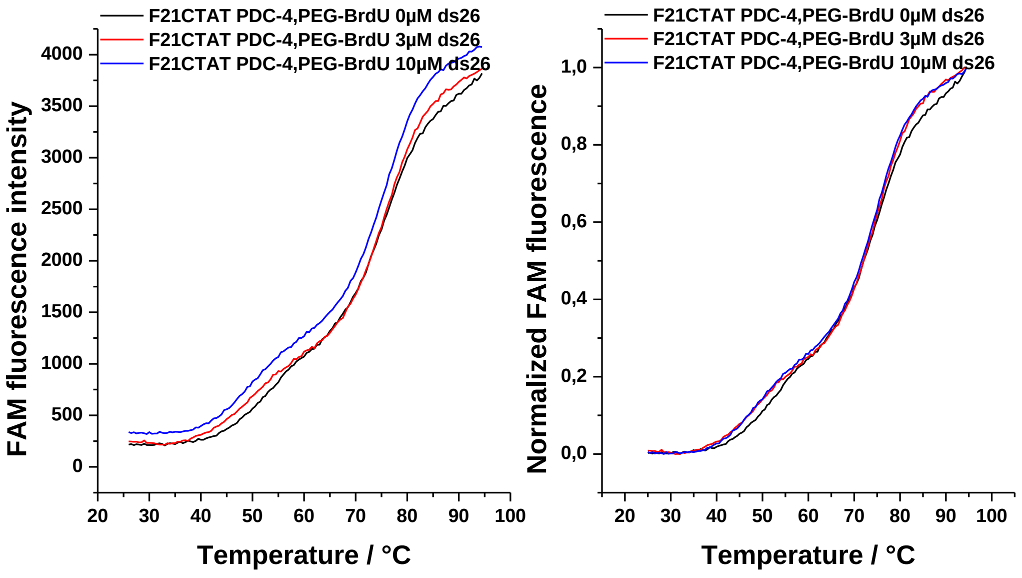
F21CTAT PDC-4,PEG-BrdU 0µM ds26
F21CTAT PDC-4,PEG-BrdU 3µM ds26
F21CTAT PDC-4,PEG-BrdU 10µM ds26
FAM fluorescence intensity
Temperature / °C
Normalized FAM fluorescence
Temperature / °C

J)

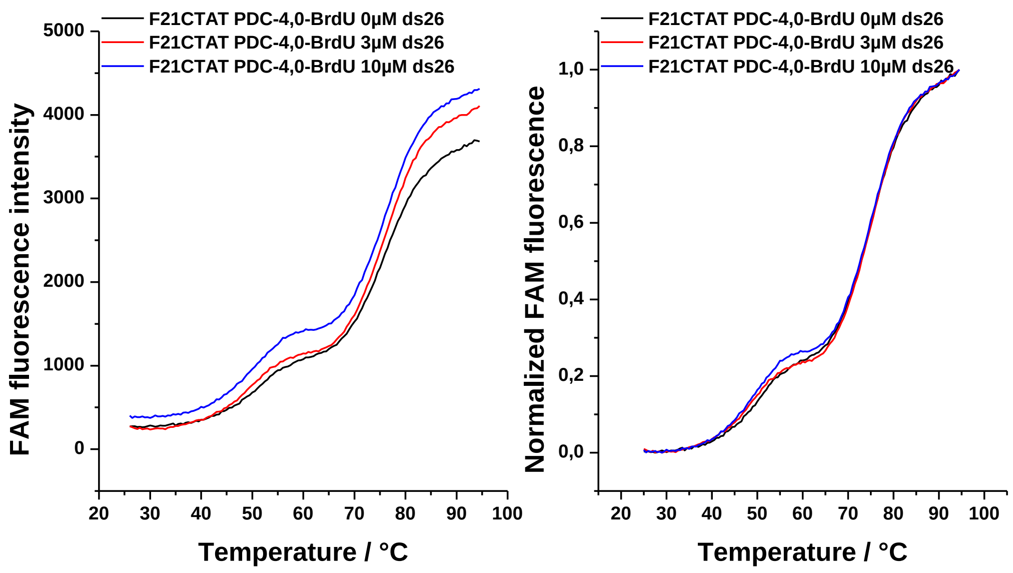

Figure S41: Examples of non-normalized and normalized FRET melting curves obtained for the human telomeric sequence variant F21CTAT (0.2 µM). A) without ligand or with B) PDC (used as a reference), C) PDC-4,2-Alk, D) PDC-4,3-Alk, E) PDC-4,PEG-N3, F) PDC-4,0-N3, G) PDC-4,2-BrdU, H) PDC-4,3-BrdU, I) PDC-4,PEG-BrdU and J) PDC-4,0-BrdU (1.0 µM). FAM fluorescence (left) and normalized FAM fluorescence (right) are plotted as a function of the temperature. Experiments are carried out in 10 mM lithium cacodylate buffer (pH 7.2), 90 mM LiCl, and 10 mM KCl without (0 µM ds26, black curves) or with duplex competitor (3 µM ds26, red curves, and 10 µM ds26, blue curves) in a total volume of 25 µL.

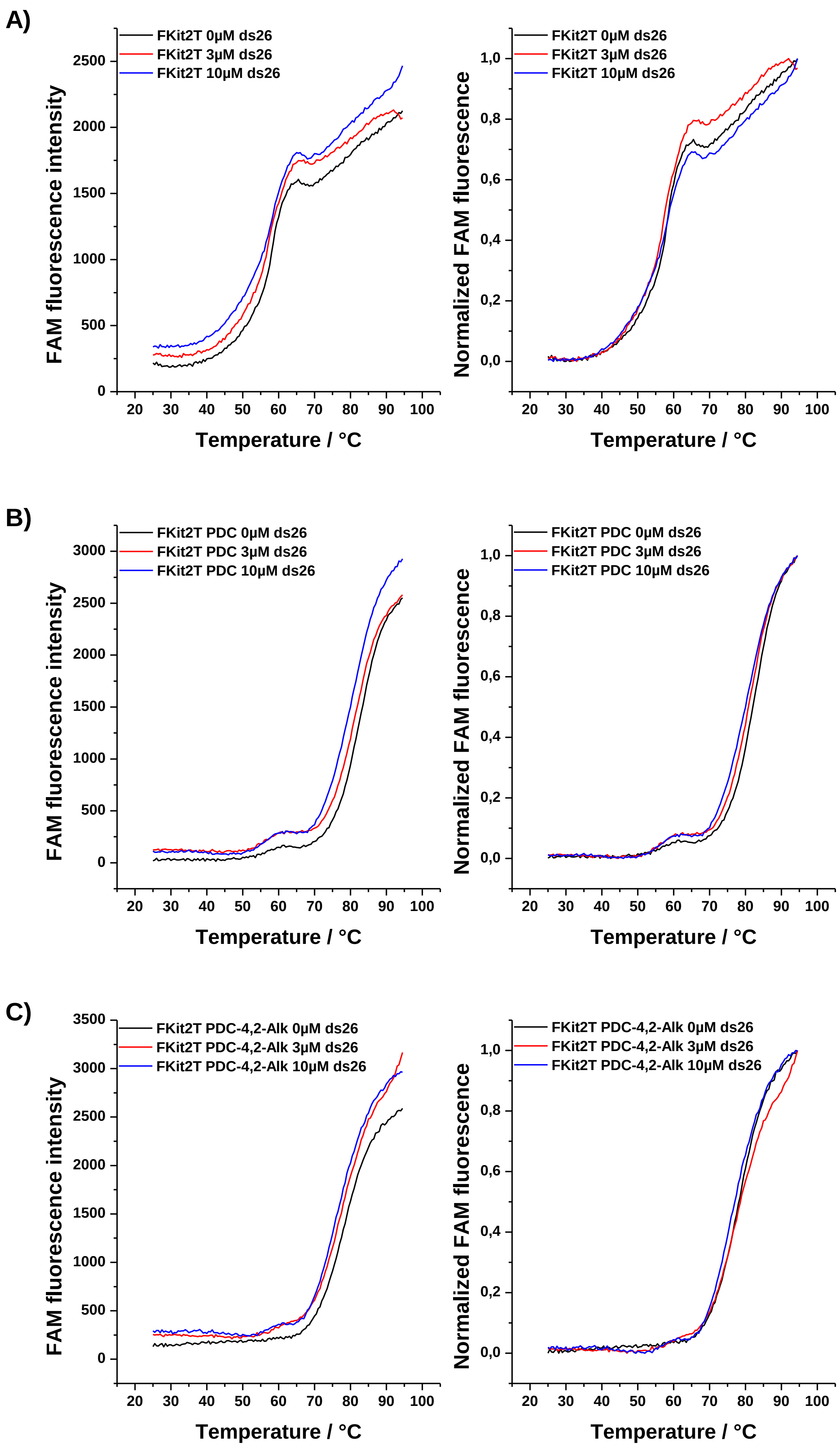

A)
FKit2T 0μM ds26
FKit2T 3μM ds26
FKit2T 10μM ds26
FAM fluorescence intensity
Normalized FAM fluorescence
Temperature / °C
B)
FKit2T PDC 0μM ds26
FKit2T PDC 3μM ds26
FKit2T PDC 10μM ds26
FAM fluorescence intensity
Normalized FAM fluorescence
Temperature / °C
C)
FKit2T PDC-4,2-Alk 0μM ds26
FKit2T PDC-4,2-Alk 3μM ds26
FKit2T PDC-4,2-Alk 10μM ds26
FAM fluorescence intensity
Normalized FAM fluorescence
Temperature / °C

D)

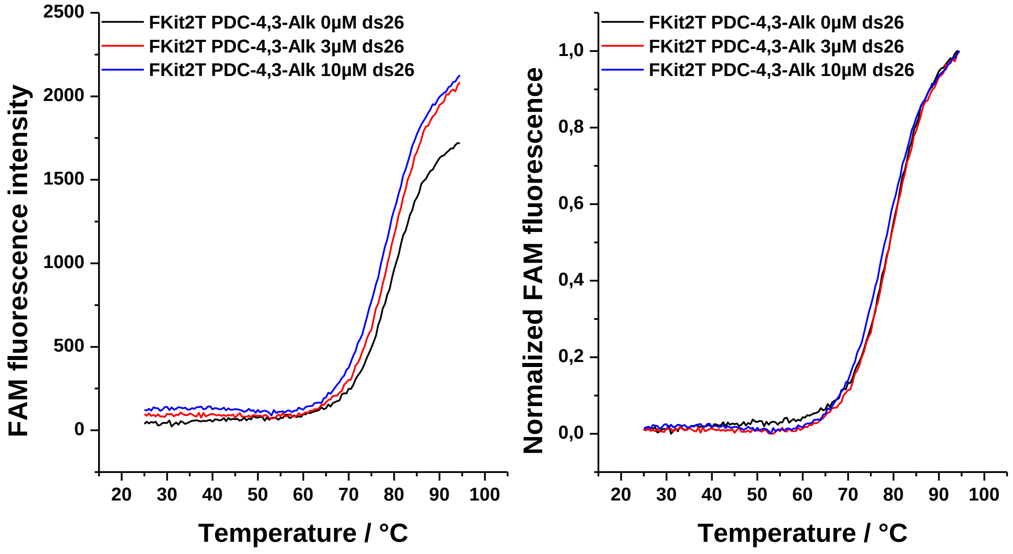
FKit2T PDC-4,3-Alk 0μM ds26
FKit2T PDC-4,3-Alk 3μM ds26
FKit2T PDC-4,3-Alk 10μM ds26
FAM fluorescence intensity
Normalized FAM fluorescence
Temperature / °C
Temperature / °C

E)

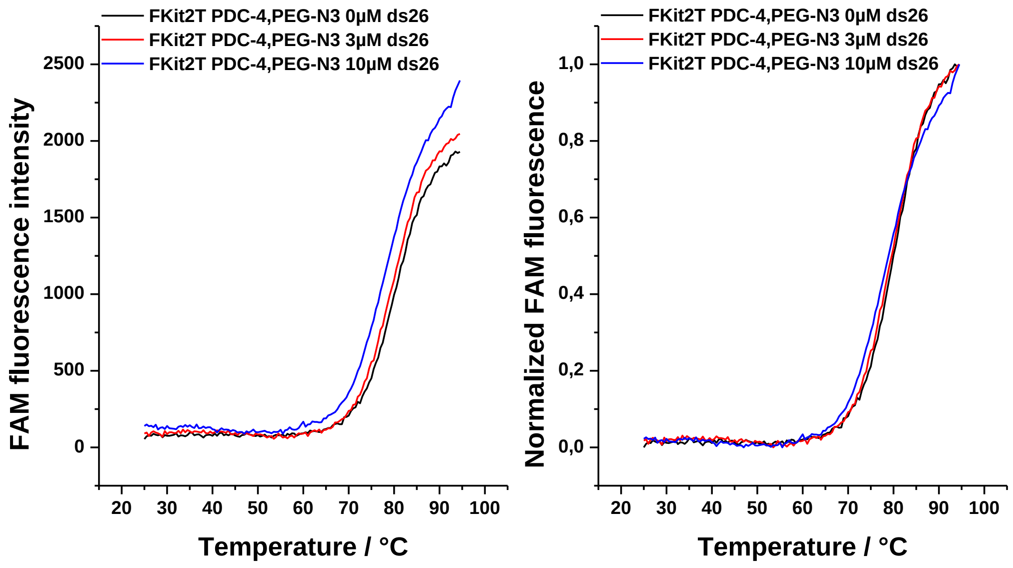
FKit2T PDC-4,PEG-N3 0μM ds26
FKit2T PDC-4,PEG-N3 3μM ds26
FKit2T PDC-4,PEG-N3 10μM ds26
FAM fluorescence intensity
Normalized FAM fluorescence
Temperature / °C
Temperature / °C

F)

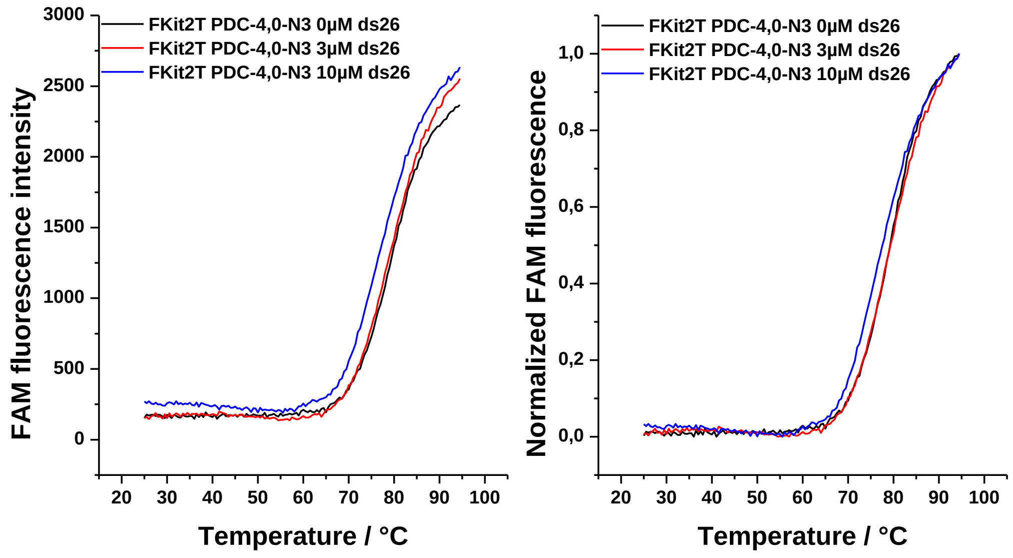
FKit2T PDC-4,0-N3 0μM ds26
FKit2T PDC-4,0-N3 3μM ds26
FKit2T PDC-4,0-N3 10μM ds26
FAM fluorescence intensity
Normalized FAM fluorescence
Temperature / °C
Temperature / °C

G)

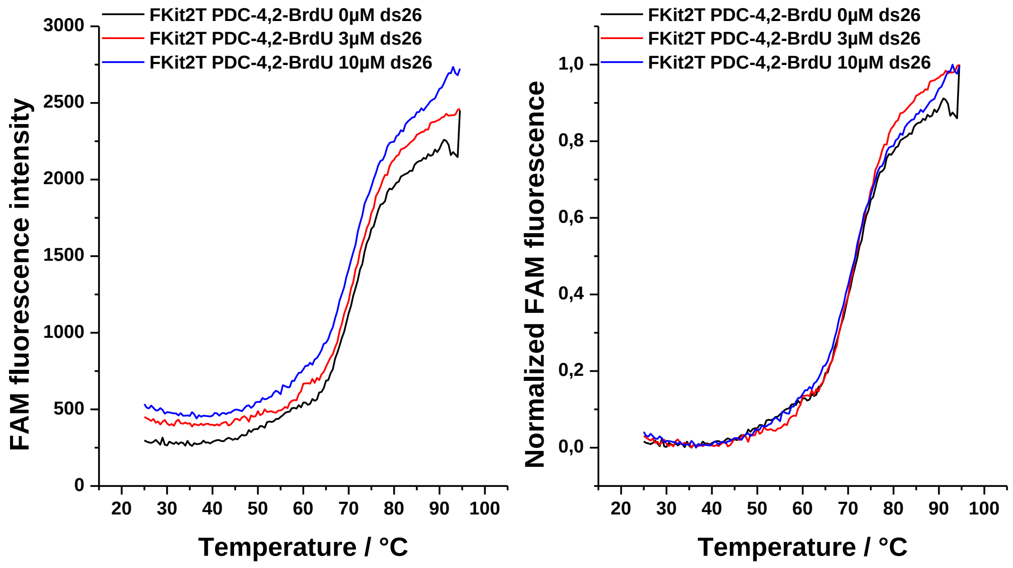
FKit2T PDC-4,2-BrdU 0µM ds26
FKit2T PDC-4,2-BrdU 3µM ds26
FKit2T PDC-4,2-BrdU 10µM ds26
FAM fluorescence intensity
Normalized FAM fluorescence
Temperature / °C

H)

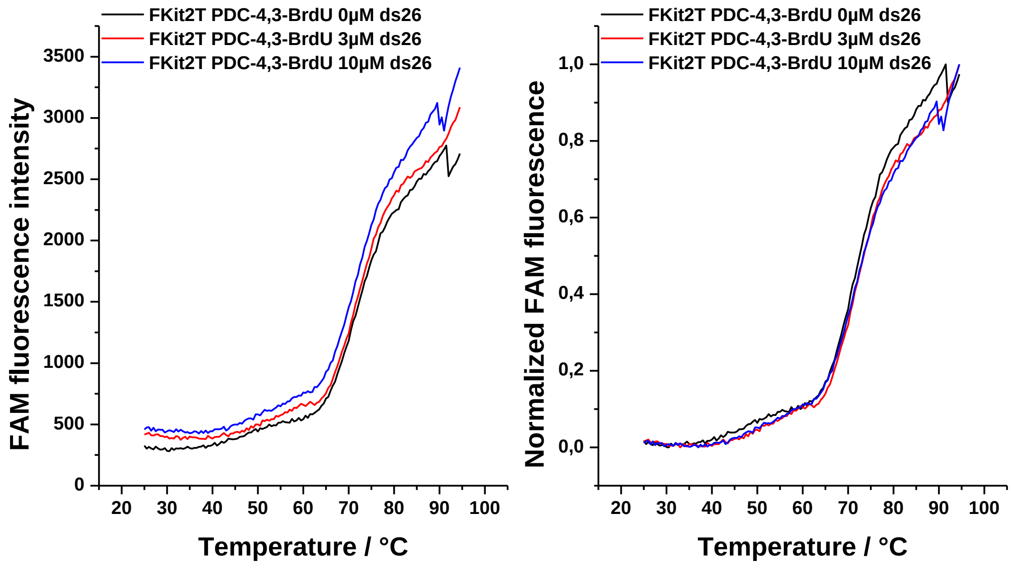
FKit2T PDC-4,3-BrdU 0µM ds26
FKit2T PDC-4,3-BrdU 3µM ds26
FKit2T PDC-4,3-BrdU 10µM ds26
FAM fluorescence intensity
Normalized FAM fluorescence
Temperature / °C

I)

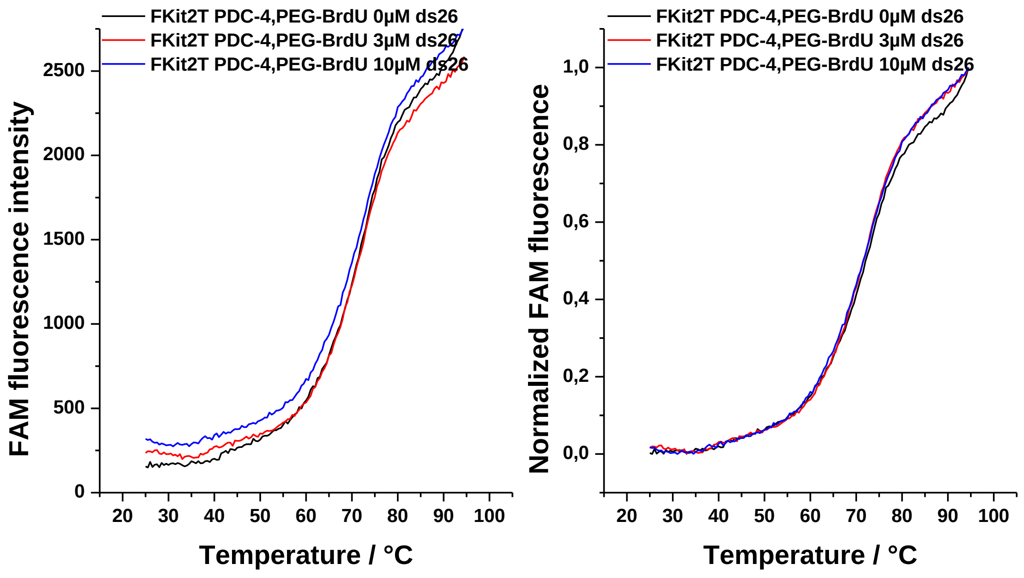
FKit2T PDC-4,PEG-BrdU 0µM ds26
FKit2T PDC-4,PEG-BrdU 3µM ds26
FKit2T PDC-4,PEG-BrdU 10µM ds26
FAM fluorescence intensity
Normalized FAM fluorescence
Temperature / °C

J)

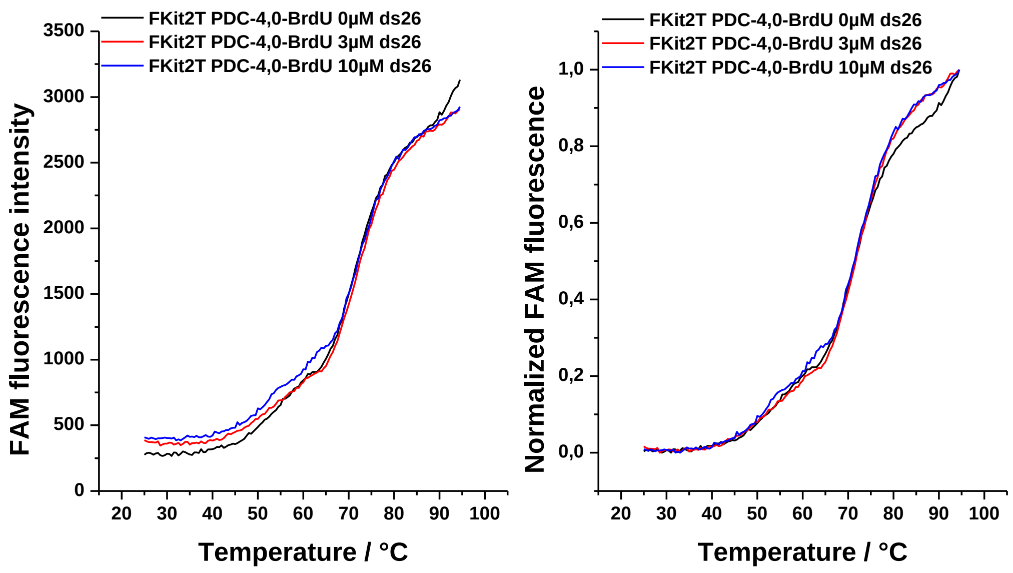

Figure S42: Examples of non-normalized and normalized FRET melting curves obtained for the c-kit2 oncogene promoter sequence Fkit2T (0.2 µM). A) without ligand or with B) PDC (used as a reference), C) PDC-4,2-Alk, D) PDC-4,3-Alk, E) PDC-4,PEG-N3, F) PDC-4,0-N3, G) PDC-4,2-BrdU, H) PDC-4,3-BrdU, I) PDC-4,PEG-BrdU and J) PDC-4,0-BrdU (1.0 µM). FAM fluorescence (left) and normalized FAM fluorescence (right) are plotted as a function of the temperature. Experiments are carried out in 10 mM lithium cacodylate buffer (pH 7.2), 99 mM LiCl, and 1 mM KCl without (0 µM ds26, black curves) or with duplex competitor (3 µM ds26, red curves, and 10 µM ds26, blue curves) in a total volume of 25 µL.

A)

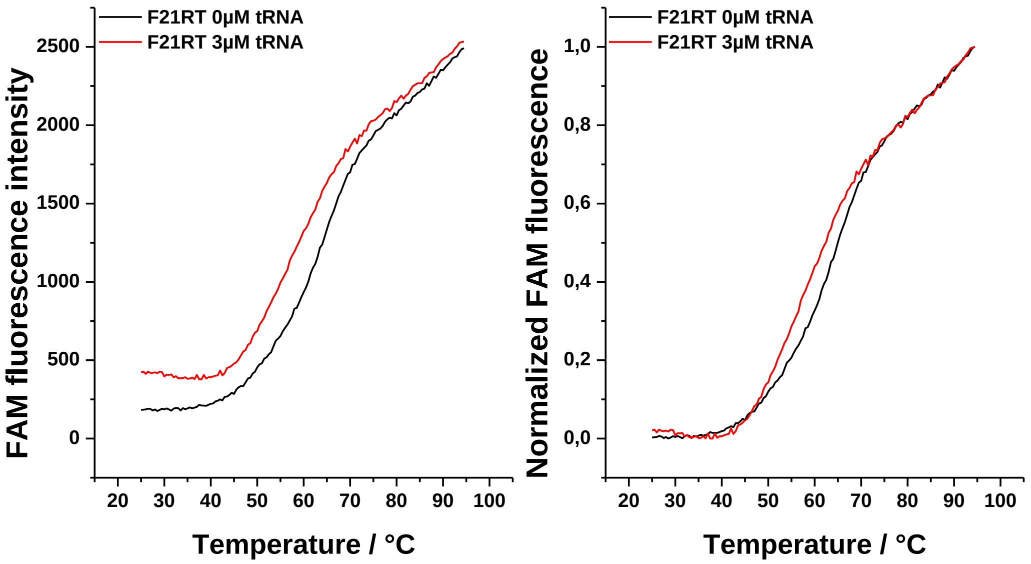
F21RT 0µM tRNA
F21RT 3µM tRNA
FAM fluorescence intensity
Normalized FAM fluorescence
Temperature / °C

B)

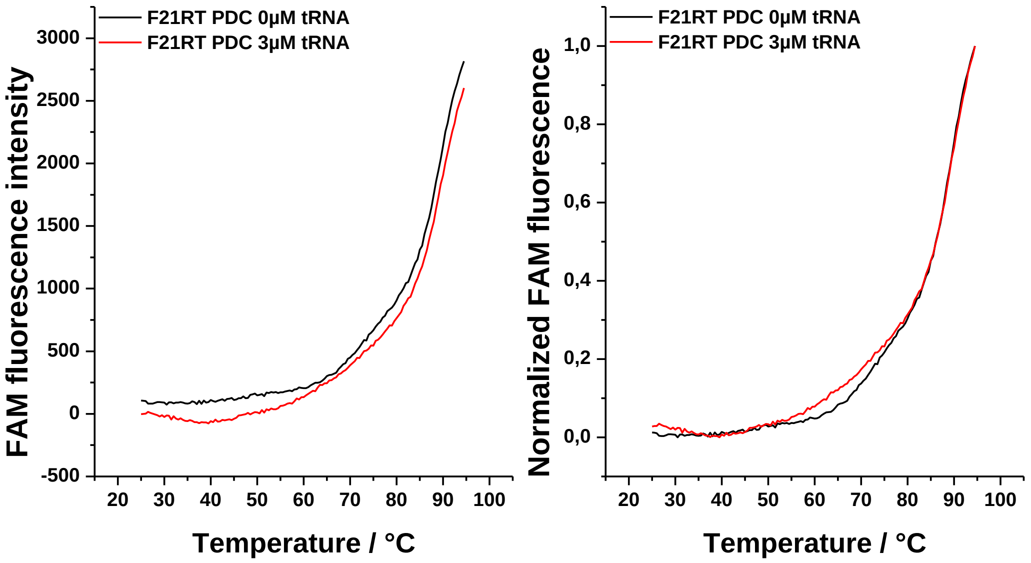
F21RT PDC 0µM tRNA
F21RT PDC 3µM tRNA
FAM fluorescence intensity
Normalized FAM fluorescence
Temperature / °C

C)

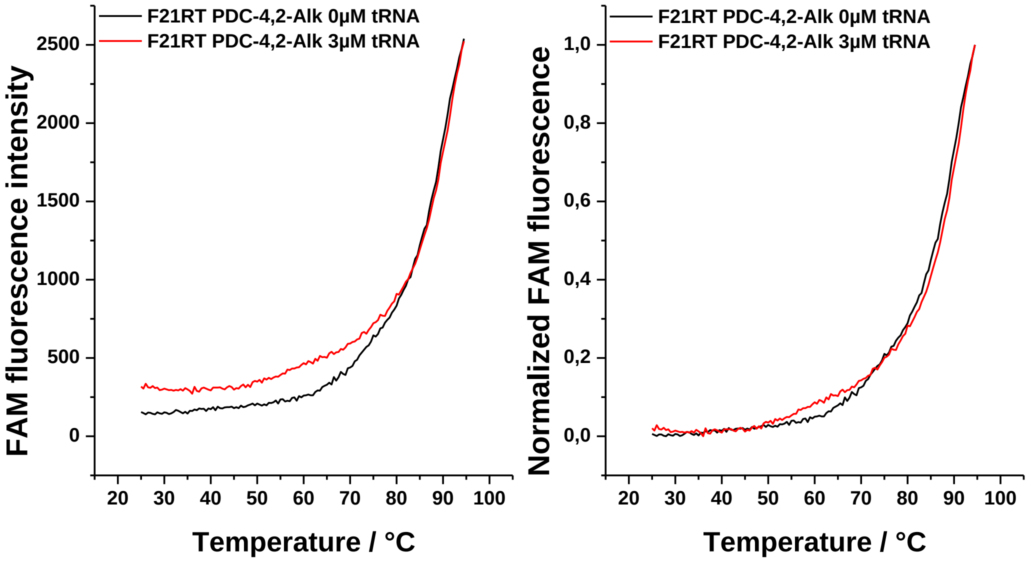
F21RT PDC-4,2-Alk 0µM tRNA
F21RT PDC-4,2-Alk 3µM tRNA
FAM fluorescence intensity
Normalized FAM fluorescence
Temperature / °C

D)

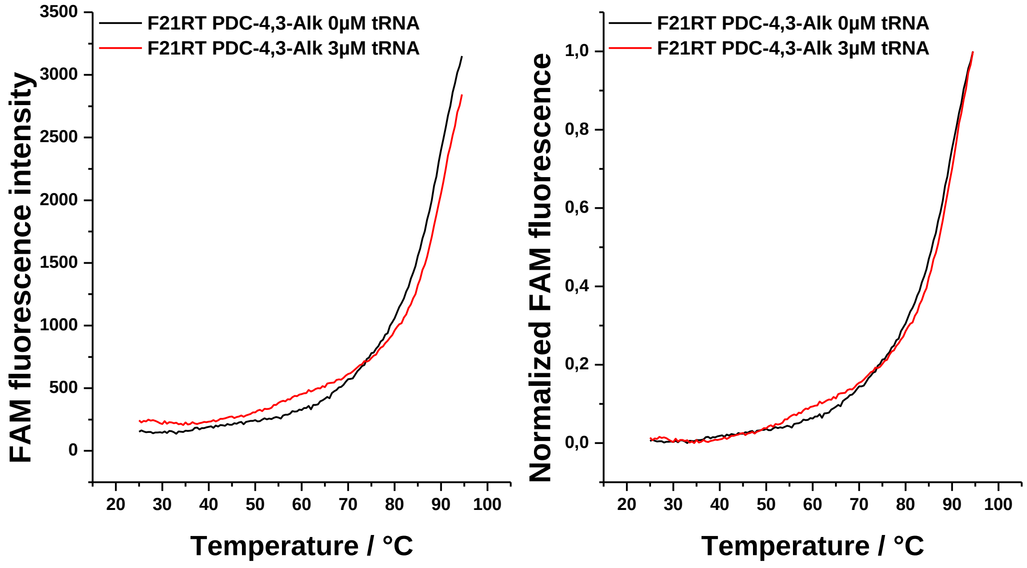
F21RT PDC-4,3-Alk 0μM tRNA
F21RT PDC-4,3-Alk 3μM tRNA
FAM fluorescence intensity
Normalized FAM fluorescence
Temperature / °C
3500
3000
2500
2000
1500
1000
500
0
1,0
0,8
0,6
0,4
0,2
0,0
20 30 40 50 60 70 80 90 100

E)

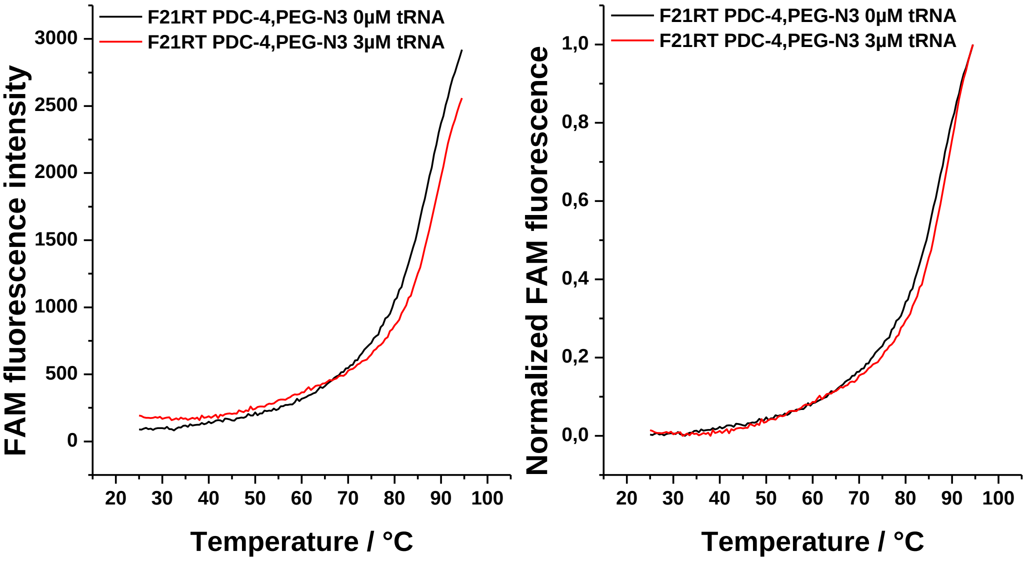
F21RT PDC-4,PEG-N3 0μM tRNA
F21RT PDC-4,PEG-N3 3μM tRNA
FAM fluorescence intensity
Normalized FAM fluorescence
Temperature / °C
3000
2500
2000
1500
1000
500
0
1,0
0,8
0,6
0,4
0,2
0,0
20 30 40 50 60 70 80 90 100

F)

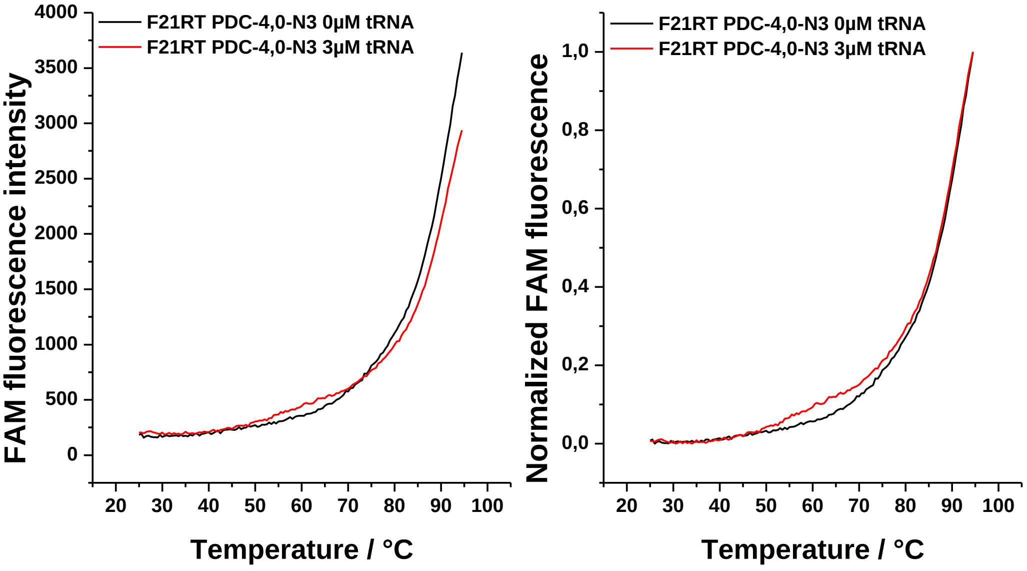
F21RT PDC-4,0-N3 0μM tRNA
F21RT PDC-4,0-N3 3μM tRNA
FAM fluorescence intensity
Normalized FAM fluorescence
Temperature / °C
4000
3500
3000
2500
2000
1500
1000
500
0
1,0
0,8
0,6
0,4
0,2
0,0
20 30 40 50 60 70 80 90 100

G)

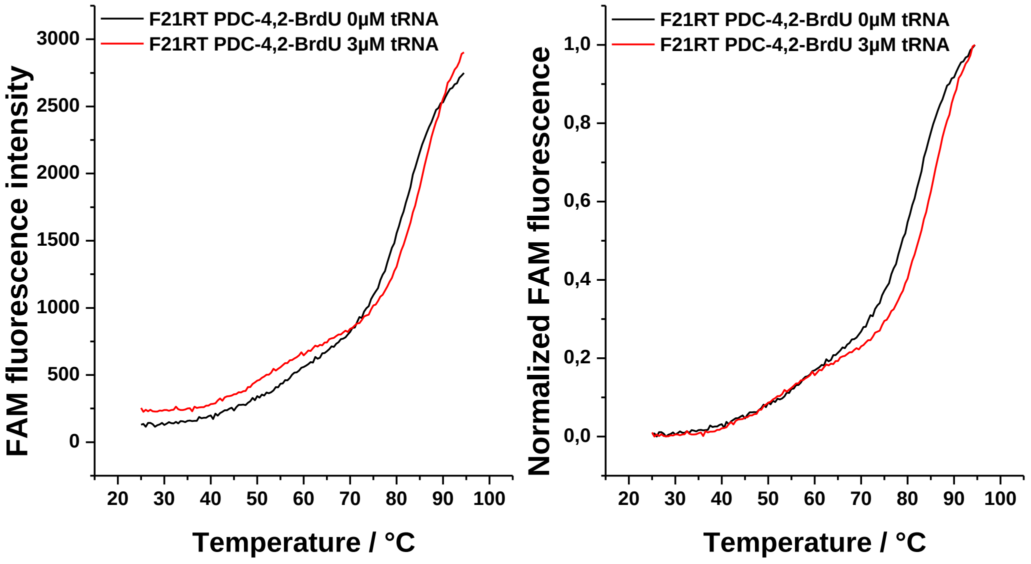
F21RT PDC-4,2-BrdU 0µM tRNA
F21RT PDC-4,2-BrdU 3µM tRNA
FAM fluorescence intensity
Normalized FAM fluorescence
Temperature / °C

H)

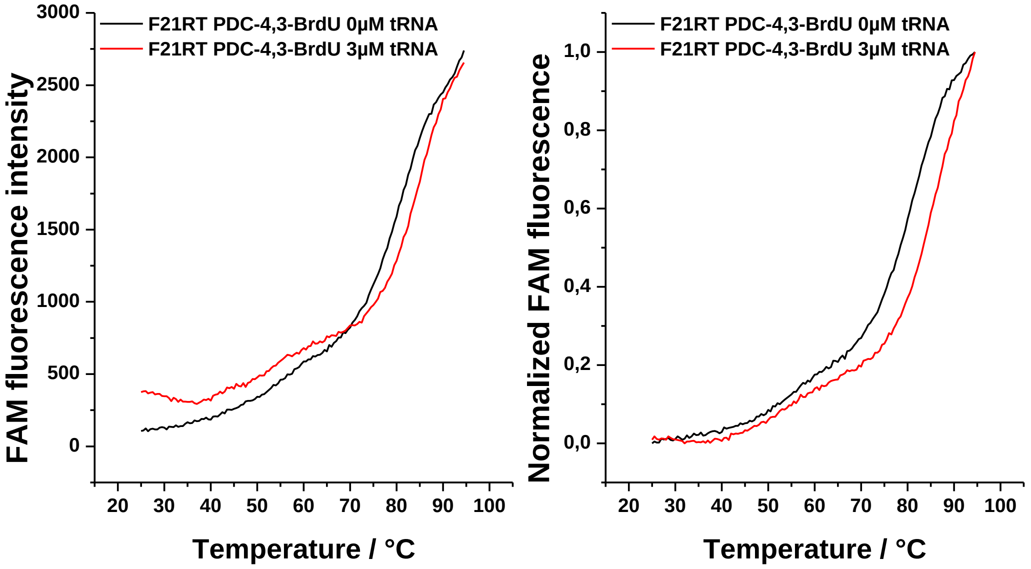
F21RT PDC-4,3-BrdU 0µM tRNA
F21RT PDC-4,3-BrdU 3µM tRNA
FAM fluorescence intensity
Normalized FAM fluorescence
Temperature / °C

I)

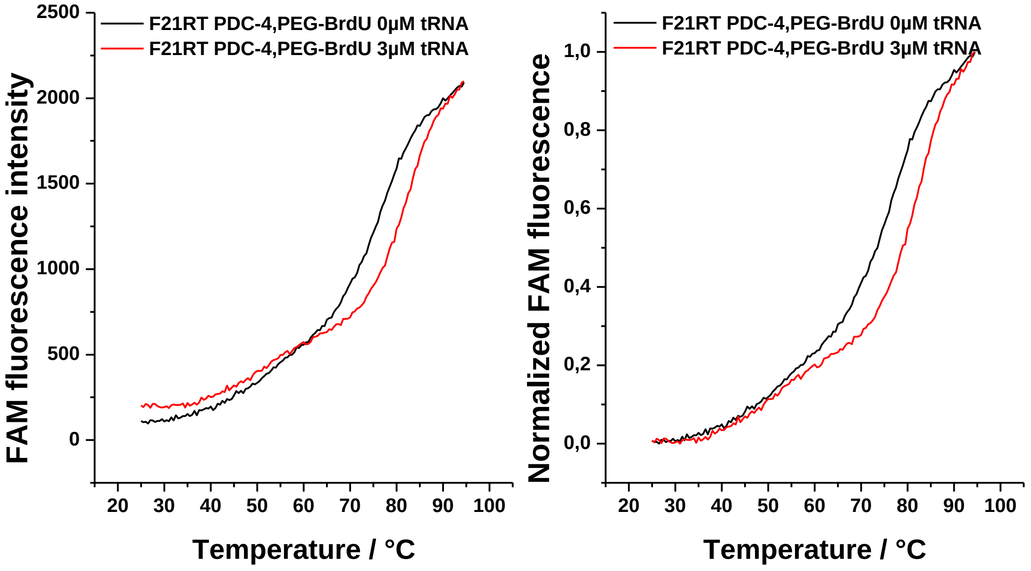
F21RT PDC-4,PEG-BrdU 0µM tRNA
F21RT PDC-4,PEG-BrdU 3µM tRNA
FAM fluorescence intensity
Normalized FAM fluorescence
Temperature / °C

J)

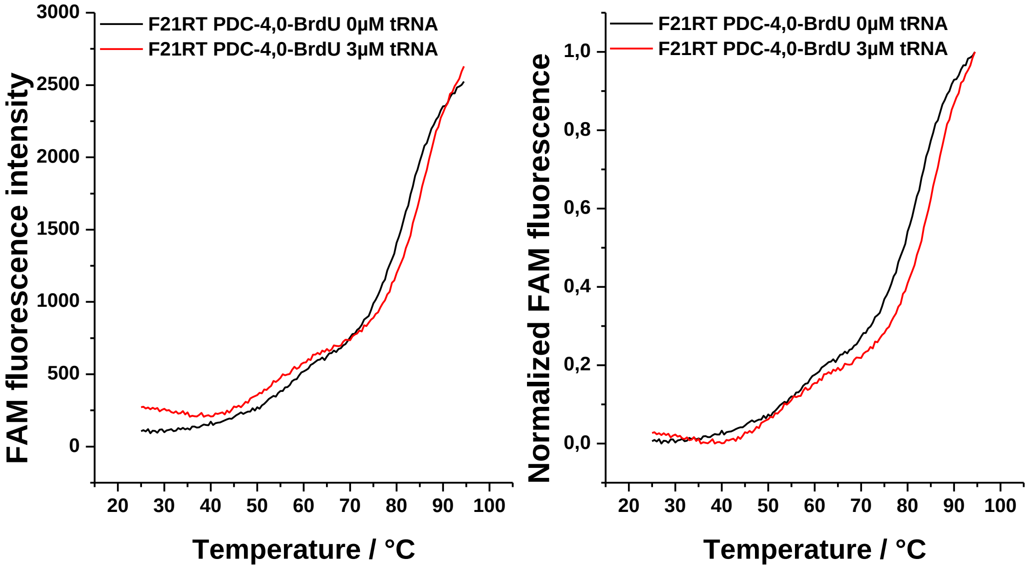

Figure S43: Examples of non-normalized and normalized FRET melting curves obtained for the RNA human telomeric sequence F21RT (0.2 µM). A) without ligand or with B) PDC (used as a reference), C) PDC-4,2-Alk, D) PDC-4,3-Alk, E) PDC-4,PEG-N3, F) PDC-4,0-N3, G) PDC-4,2-BrdU, H) PDC-4,3-BrdU, I) PDC-4,PEG-BrdU and J) PDC-4,0-BrdU (1.0 µM). FAM fluorescence (left) and normalized FAM fluorescence (right) are plotted as a function of the temperature. Experiments are carried out in 10 mM lithium cacodylate buffer (pH 7.2), 99 mM LiCl, and 1 mM KCl without (0 µM tRNA, black curves) or with ssRNA competitor (3 µM tRNA, red curves) in a total volume of 25 µL.

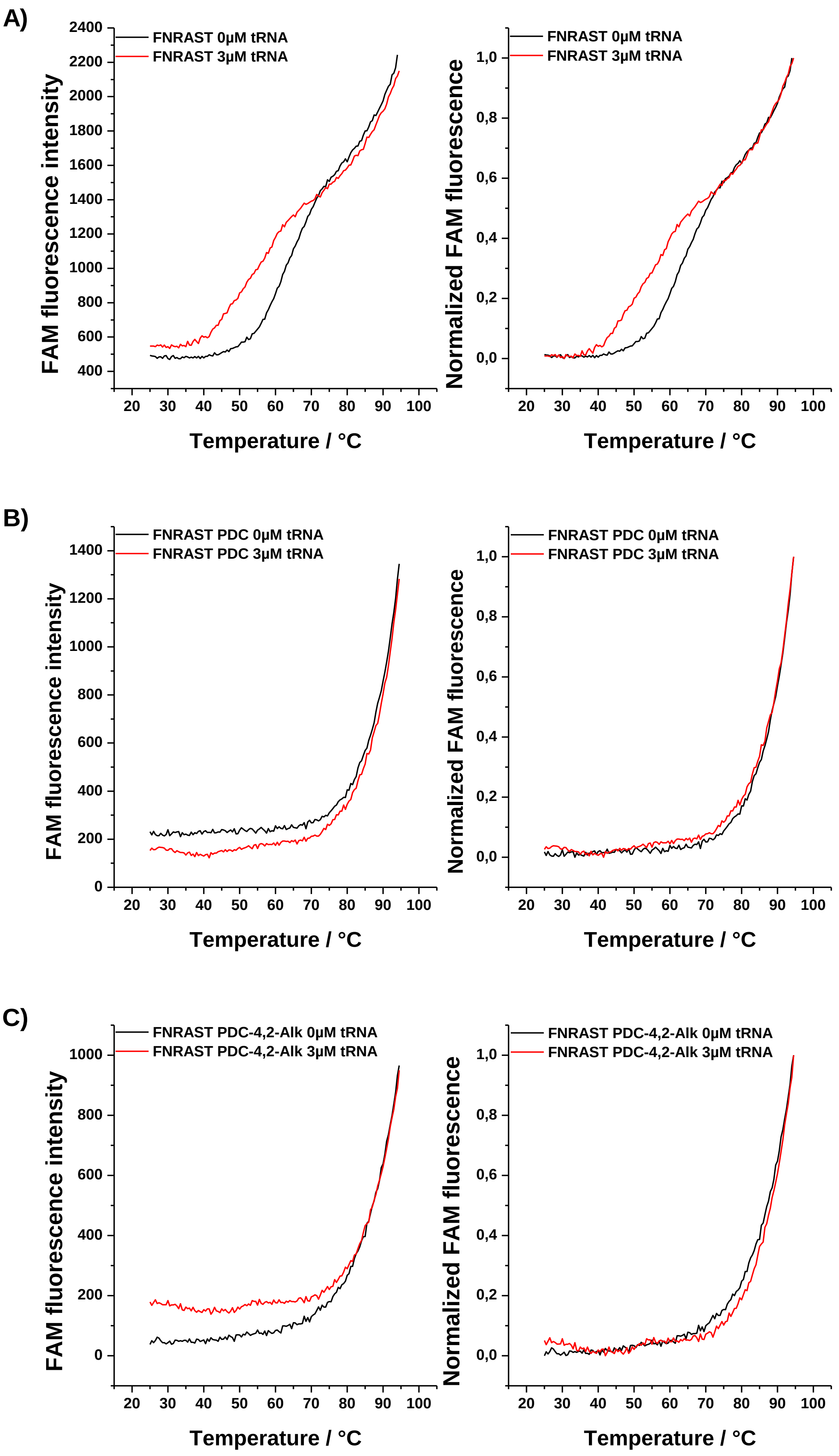

A)
FNRAST 0µM tRNA
FNRAST 3µM tRNA
FAM fluorescence intensity
Normalized FAM fluorescence
Temperature / °C
B)
FNRAST PDC 0µM tRNA
FNRAST PDC 3µM tRNA
FAM fluorescence intensity
Normalized FAM fluorescence
Temperature / °C
C)
FNRAST PDC-4,2-Alk 0µM tRNA
FNRAST PDC-4,2-Alk 3µM tRNA
FAM fluorescence intensity
Normalized FAM fluorescence
Temperature / °C

D)

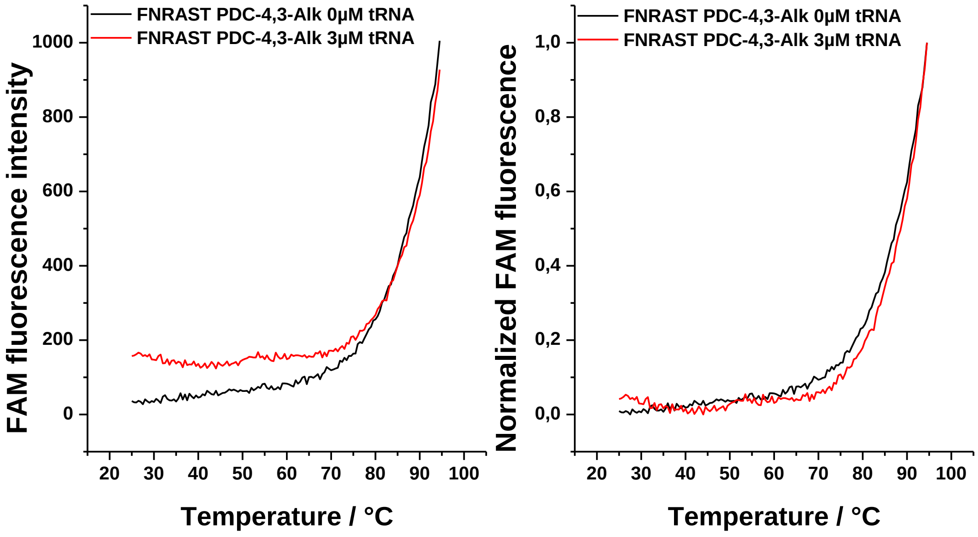
FNRAST PDC-4,3-Alk 0µM tRNA
FNRAST PDC-4,3-Alk 3µM tRNA
FAM fluorescence intensity
Normalized FAM fluorescence
Temperature / °C
Temperature / °C

E)

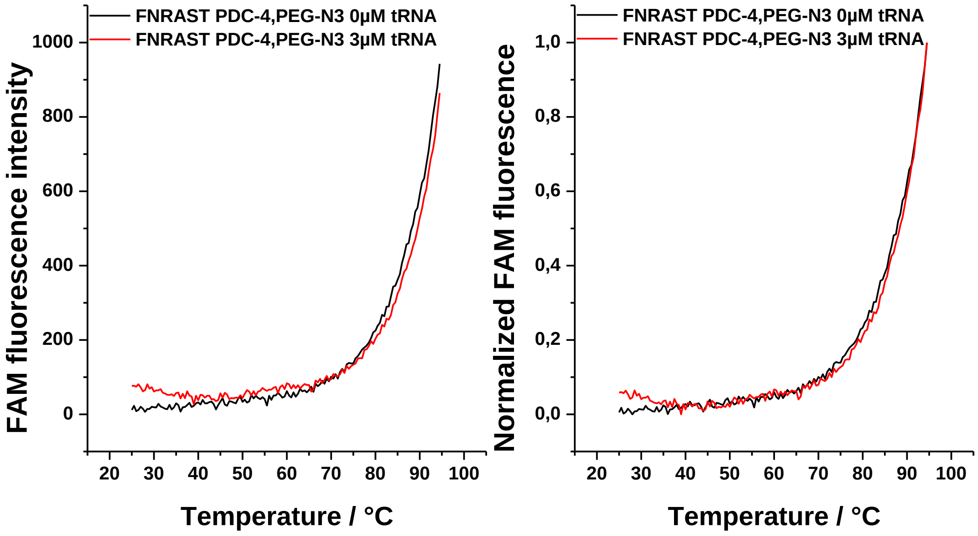
FNRAST PDC-4,PEG-N3 0µM tRNA
FNRAST PDC-4,PEG-N3 3µM tRNA
FAM fluorescence intensity
Normalized FAM fluorescence
Temperature / °C
Temperature / °C

F)

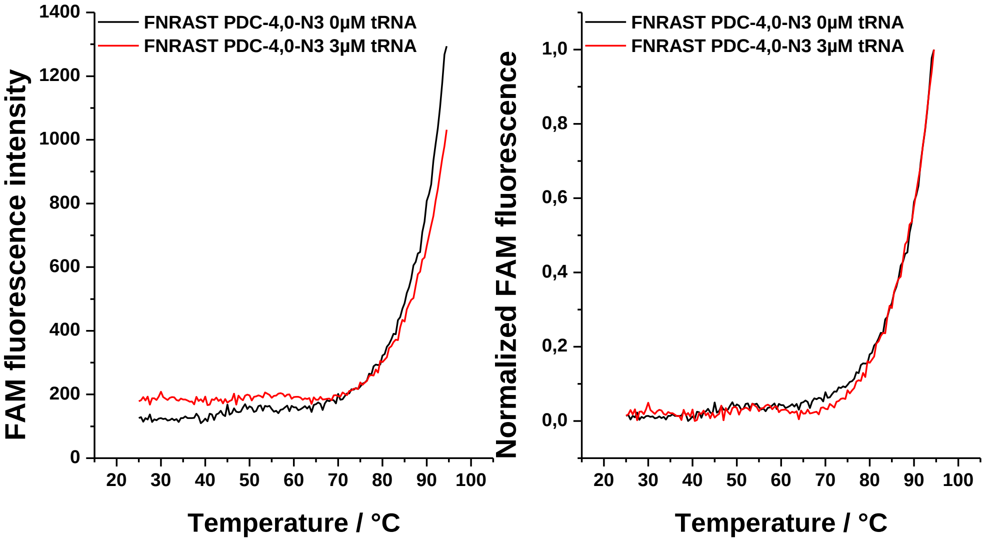
FNRAST PDC-4,0-N3 0µM tRNA
FNRAST PDC-4,0-N3 3µM tRNA
FAM fluorescence intensity
Normalized FAM fluorescence
Temperature / °C
Temperature / °C

G)

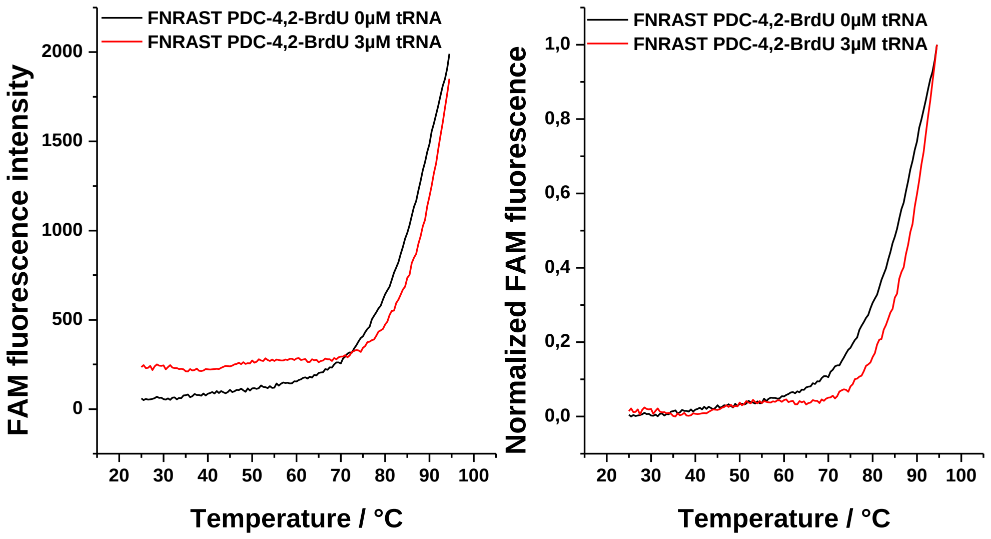
FNRAST PDC-4,2-BrdU 0µM tRNA
FNRAST PDC-4,2-BrdU 3µM tRNA
FAM fluorescence intensity
Temperature / °C
FNRAST PDC-4,2-BrdU 0µM tRNA
FNRAST PDC-4,2-BrdU 3µM tRNA
Normalized FAM fluorescence
Temperature / °C

H)

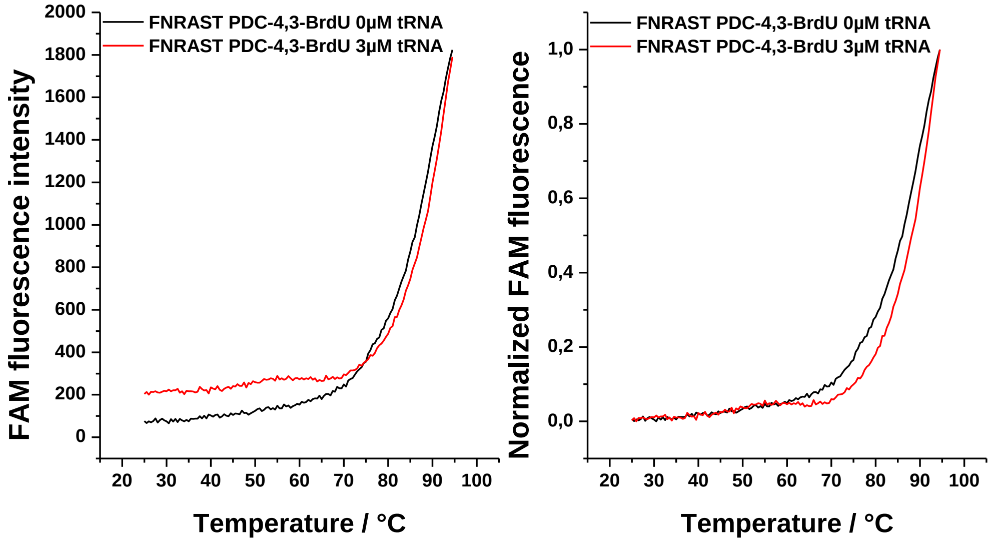
FNRAST PDC-4,3-BrdU 0µM tRNA
FNRAST PDC-4,3-BrdU 3µM tRNA
FAM fluorescence intensity
Temperature / °C
FNRAST PDC-4,3-BrdU 0µM tRNA
FNRAST PDC-4,3-BrdU 3µM tRNA
Normalized FAM fluorescence
Temperature / °C

I)

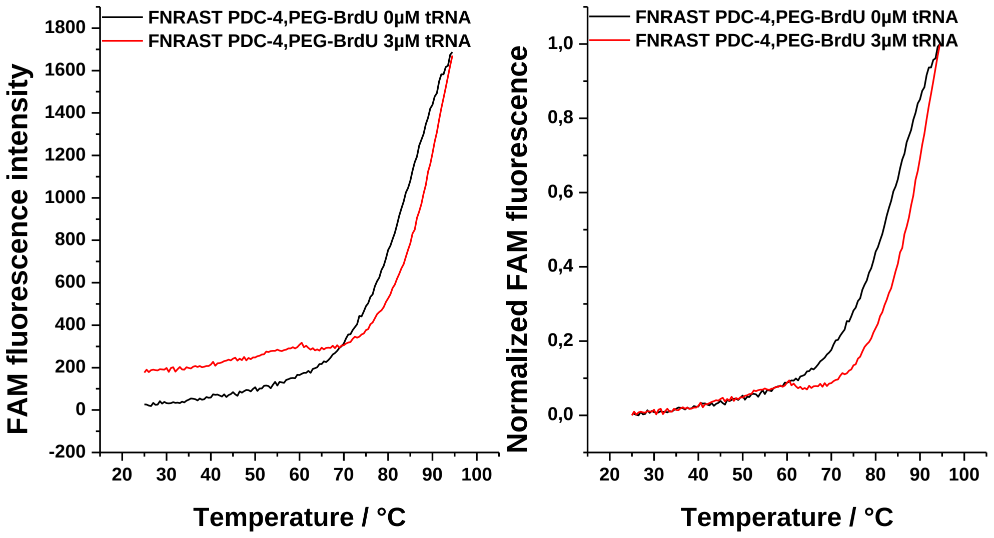
FNRAST PDC-4,PEG-BrdU 0µM tRNA
FNRAST PDC-4,PEG-BrdU 3µM tRNA
FAM fluorescence intensity
Temperature / °C
FNRAST PDC-4,PEG-BrdU 0µM tRNA
FNRAST PDC-4,PEG-BrdU 3µM tRNA
Normalized FAM fluorescence
Temperature / °C

J)

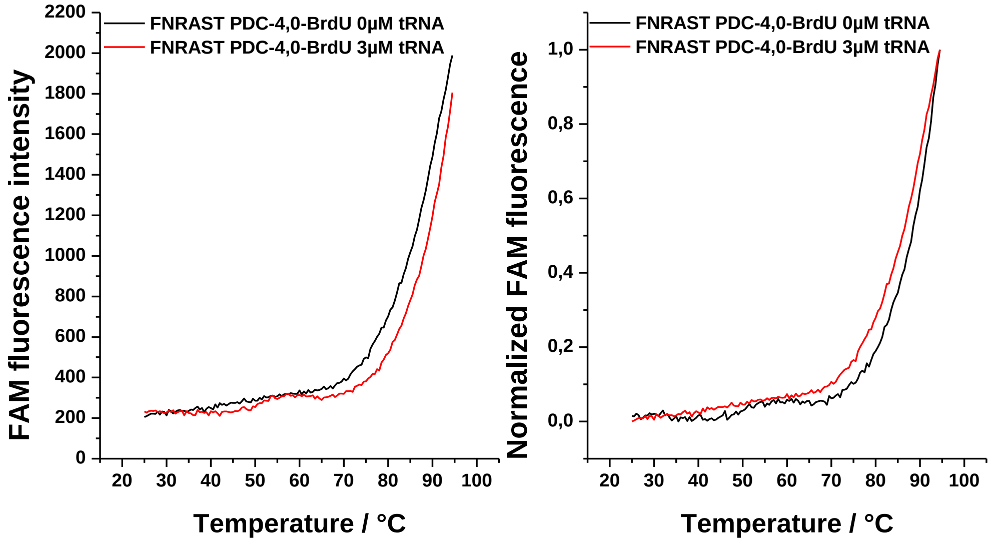

Figure S44: Examples of non-normalized and normalized FRET melting curves obtained for the RNA G4-forming sequence of the human NRAS proto-oncogene transcript FNRAST (0.2 µM). A) without ligand or with B) PDC (used as a reference), C) PDC-4,2-Alk, D) PDC-4,3-Alk, E) PDC-4,PEG-N3, F) PDC-4,0-N3, G) PDC-4,2-BrdU, H) PDC-4,3-BrdU, I) PDC-4,PEG-BrdU and J) PDC-4,0-BrdU (1.0 µM). FAM fluorescence (left) and normalized FAM fluorescence (right) are plotted as a function of the temperature. Experiments are carried out in 10 mM lithium cacodylate buffer (pH 7.2), 99 mM LiCl, and 1 mM KCl without (0 µM tRNA, black curves) or with ssRNA competitor (3 µM tRNA, red curves) in a total volume of 25 µL.
