# Supplementary material for: BrdU immuno-tagged G-quadruplex ligands: a new ligand-guided immunofluorescence approach for tracking G-quadruplexes in cells
Source: Nucleic Acids Res. 2021 Dec 7;49(22):12644–60. doi: 10.1093/nar/gkab1166 (PMC8682774; doi:10.1093/nar/gkab1166)
Supplement: gkab1166_Supplemental_Files [file gkab1166_supplemental_files.zip › Masson et al. NAR_2021_Supplementary Information_Revised.pdf]

Supplementary Information

BrdU immuno-tagged G-quadruplex ligands: a new ligand-guided immunofluorescence approach for tracking G-quadruplexes in cells

Thibaut Masson[1,2], Corinne Landras Guetta[1,2], Eugénie Laigre[1,2], Anne Cucchiarini[1,2], Patricia Duchambon[1,2], Marie-Paule Teulade-Fichou[1,2,*], and Daniela Verga[1,2,*]

[1] CNRS UMR9187, INSERM U1196, Institut Curie, PSL Research University, F-91405 Orsay, France
[2] CNRS UMR9187, INSERM U1196, Université Paris-Saclay, F-91405 Orsay, France

Present Address: Eugénie Laigre, CNRS, Department of Molecular Chemistry, University Grenoble Alpes, UMR 5250, 38000 Grenoble, France; Anne Cucchiarini, Laboratoire d'Optique et Biosciences, Ecole Polytechnique, CNRS, Inserm, Institut Polytechnique de Paris, 91128 Palaiseau, France.

Table of Contents

General experimental Synthetic chemistry and Biophysical methods **3**

Synthesis and characterization of compounds **5**

Synthetic pathway followed for the preparation of copper-catalyzed alkyne-azide cycloaddition (CuAAC) precursors **5**

Synthetic pathway followed for the preparation of final compounds PDC-4,2-BrdU, PDC-4,3-BrdU, PDC-4,PEG-BrdU, and PDC-4,0-BrdU **14**

Scheme S1. Synthetic pathway exploited for the preparation of final compounds PDC-4,2-BrdU, PDC-4,3-BrdU, PDC-4,0-BrdU and PDC-4,PEG-BrdU **14**

Table S1. Doubly-labeled sequences used for FRET experiments **17**

Figure S1: FRET-melting competition experiments with DNA sequences **18-19**

Figure S2: FRET-melting competition experiments with RNA sequences **20**

Table S2. G-quadruplex sequences for G4-FID assay **21**

Figure S3-S11: G4 Fluorescent Intercalator Displacement (FID) plots **22-26**

Table S3. DC_{50} values of PDC derivatives obtained by G4-FID assay **27**

Figure S12: PDC-4,2-Alk click reaction kinetics **28**

Figure S13-S15.Chromatographic traces for the CuAAC reaction in the presence of c-Myc22, 22AG, and TERRA **29-34**

Table S4. Cytotoxicity of the different synthesized ligands in A549 cell lines **35**

Figure S16. Control experiments of immunofluorescent staining **36**

Figure S17-S20. Immunofluorescence experiments PDC-4,2-BrdU, PDC-4,3-BrdU, PDC-4,PEG-BrdU, PDC-4,0-BrdU **37-38**

Figure S21-S24. Immunofluorescence experiments PDC-4,2-Alk, PDC-4,3-Alk, PDC-4,PEG-N3, PDC-4,0-N3 **39-42**

Figure S25. Immunofluorescence experiments PDC-4,3-Alk in A2780 cell line **43**

Figure S26. Cy5 control experiments **44**

Figure S27. Kinetics incorporation experiments PDC-4,3-Alk in A549 cells **45**

Figure S28-S31: RNase A treatment: PDC-4,2-BrdU, PDC-4,3-BrdU PDC-4,PEG-BrdU, PDC-4,0-BrdU **46-47**

Figure S32-S35: RNase A treatment: PDC-4,2-Alk, PDC-4,3-Alk PDC-4,PEG-N3, PDC-4,0- N3 **48-49**

Figure S36. Column scatter plots representing BG4 foci generated by PDC-4,3-BrdU **50**

References **51**

General experimental Synthetic chemistry and Biophysical methods

All commercially available chemicals were bought from Sigma-Aldrich, Acros, Fluorochem, Fisher Scientific, TCI and Alfa Aesar as reagent grade and were used without further purification. Anhydrous solvents (DMF, THF and DCM) were obtained from MBRAUN Solvent Purification System. Chelex® resin 100 sodium form (50 – 100 mesh particle) was purchased from Sigma-Aldrich. Melting points were determined on a Kofler system type WME from Wagner & Munz. Thin-layer chromatography and flash chromatography were performed using pre-coated silica gel plates (Merck 60 F_{254}) with visualization at 254 and 366 nm. Flash chromatography was performed with silica gel 60 (40-63 µm, Merk). Deuterated solvents DMSO-d_6 and MeOH-d_4 were purchased from Eurisotop. All ^{1}H NMR and APT ^{13}C NMR spectra were recorded on a Bruker Advance 300 MHz spectrometer except ^{1}H NMR of PDC-4,2-BrdU which was recorded on a 500 MHz Bruker Avance III spectrometer, using deuterated solvents and TMS as internal standard. The spectra are reported in ppm and referenced to deuterated DMSO (2.50 ppm for ^{1}H, 39.52 ppm for ^{13}C) or deuterated MeOH (4.87 ppm for ^{1}H). Data for ^{1}H NMR are reported as follows: chemical shift (δ ppm), multiplicity (s = singlet, d = doublet, t = triplet, q = quartet, quint = quintet, dd = doublet of doublets, ddd = doublet of doublet of doublets, dt = doublet of triplets, m = multiplet, bs = broad singlet), coupling constant (Hz), integration. All compounds showed > 95% purity, assessed by HPLC analysis using either MS or UV absorbance or diode array detectors. Synthetic compounds were analyzed by LC-MS experiments carried out on a HPLC Alliance 2695 unit (Waters, USA) equipped with a Phenomenex Luna Omega Polar C18 column (50x3 mm i.d, 3 µm) and coupled to a low resolution mass spectrometry (ESI-MS) micromass ZQ 2000 (Waters).

Gradient HPLC injection:

Method A = flow 0.6 mL/min; gradient elution: eluent A% H_2O 0.1% FA (formic acid), eluent B% MeCN 0.1% FA: 0 min (95/5), 7 min (5/95), 9 min (5/95), 9 min 10 s (95/5), 15 min (95/5).

Method B = flow 0.6 mL/min; gradient elution: eluent A% H_2O 0.1% FA (formic acid), eluent B% MeCN 0.1% FA: 0 min (95/5), 7 min (0/100), 10 min (95/5).

Analytical method to control final compound purity (Column HPLC: XBridge C18 Column (150x3.0 i.d., 3 µm)):

Method C = flow 0.4 mL/min; gradient elution: eluent A% H_2O 0.1% FA (formic acid), eluent B% MeCN 0.1% FA: 0 min (95/5), 2 min (95/5), 8 min (0/100), 16 min (0/100), 16 min 30 s (95/5), 20 min (95/5)

When needed, DMSO was evaporated using a SpeedVac Vacuum Concentrator Plus from Eppendorf.

This work has benefited from the facilities and expertise of the Small Molecule Mass Spectrometry platform and of the High-field NMR platform of ICSN (Centre de Recherche de Gif - www.icsn.cnrs-gif.fr).

PDC, PDC-4,2-Alk, PDC-4,3-Alk, PDC-4,0-N3, and PDC-4,PEG-N3 were dissolved in DMSO to obtain solutions at 2 mM concentration and stored in the dark at -20°C. PDC-4,2-BrdU, PDC-4,3-BrdU,

PDC-4,0-BrdU, and PDC-4,PEG-BrdU were dissolved in DMSO to obtain solutions at 2 mM concentration and used directly. $CuSO_4.5H_2O$ and sodium ascorbate were freshly prepared in water to obtain a solution at 100 mM concentration.

Oligonucleotide sequences were purchased from Eurogentec as dried samples purified by HPLC-RP. tRNA sequence from bovine liver was purchased from Sigma as dried sample. Oligonucleotides were dissolved in MilliQ water, stored at -20 °C and concentrations were determined by absorption measurements at 260 nm at 95 °C using the molar extinction coefficient provided by Eurogentec. tRNA was dissolved at ≃ 200 mM in a 10mM Caco.Li buffer previously sterilized. The final concentration was calculated using 321.47 g/mol as the average MW of a *ss*RNA nucleobase and 85 the average number of nucleobases of the sequence.

Synthesis and characterization of compounds

Synthetic pathway followed for the preparation of copper-catalyzed alkyne-azide cycloaddition (CuAAC) precursors

Synthetic protocol followed for the preparation of ***PDC-4,2-Alk****,* ***PDC-4,3-Alk*** *and* ***PDC-4,PEG-N3***

Compound **1** and compound **2** were synthesized as previously reported.(1), (2) Compound **PDC-4,2-Alk** was previously published, but in here we modified slightly the synthetic protocol and reported the full characterization.

*Compounds **3a**, **3b**, and **3c** were synthesized following pathway A:*
Compound **2** (1.0 equiv) was mixed with the corresponding carboxylic acid (1.5 equiv), EDCI (1.5 equiv), and HOBt (0.15 equiv) in dry DMF. The resulting orange solution was stirred at room temperature under argon atmosphere for 16 h. The mixture was then concentrated under reduced pressure and the obtained solid was washed 3x with water and dried under vacuum. The crude product was purified by SiO_2 chromatography.

Compound 3a

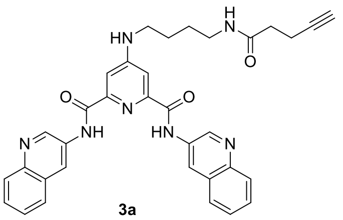

3a

Compound **2** (400 mg, 0.791 mmol), 4-pentynoic acid (116.4 mg, 1.187 mmol), EDCI (184.2 mg, 1.187 mmol), HOBt (16.0 mg, 0.119 mmol), DMF (8 mL).
Purification: DCM/MeOH from 100/0 to 94/6, leading to compound **3a** as a white solid (355 mg, 77%).

Mp = 250 °C; **^{1}H NMR** (300 MHz, DMSO-*d6*) δ (ppm): 11.28 (s, 2H), 9.36 (d, *J* = 2.4 Hz, 2H), 8.96 (d, *J* = 2.3 Hz, 2H), 8.04 (d, *J* = 8.6 Hz, 4H), 7.92 (t, *J* = 5.6 Hz, 1H), 7.72 (dt, *J* = 7.5, 1.4 Hz, 2H), 7.64 (dt, *J* = 7.5, 1.0 Hz, 2H), 7.59 – 7.48 (m, 3H), 3.25 (dt, *J* = 6.3, 5.5 Hz, 2H), 3.10 (dt, *J* = 6.4, 5.9 Hz, 2H), 2.75 (t, *J* = 2.5 Hz, 1H), 2.39 – 2.32 (m, 2H), 2.27 (t, *J* = 6.8 Hz, 2H), 1.72 – 1.47 (m, 4H); **^{13}C NMR** (75 MHz, DMSO-*d6*) δ (ppm): 170.14, 163.16, 156.29, 146.09, 144.66, 131.96, 128.69, 128.32, 127.90, 127.75, 127.18, 124.13, 83.81, 71.30, 41.74, 38.14, 34.29, 26.74, 25.57, 14.34; **LC/MS** (Method A): Retention time 6.20 min, m/z = 586.5 $[M-H]^+$.

Compound 3b

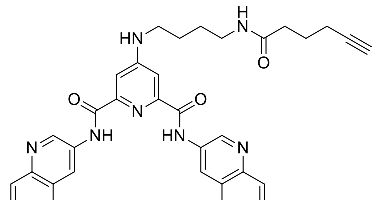

3b

Compound **2** (400 mg, 0.791 mmol), 5-hexynoic acid (133.0 mg, 1.187 mmol), EDCI (184.2 mg, 1.187 mmol), HOBt (16.0 mg, 0.119 mmol), DMF (8 mL).

Purification: DCM/MeOH from 100/0 to 94/6, leading to compound **3b** as a white solid (330 mg, 70%).

Mp = 198 °C; **^{1}H NMR** (300 MHz, DMSO-*d6*) δ (ppm): 11.28 (s, 2H), 9.36 (d, *J* = 2.4 Hz, 2H), 8.97 (d, *J* = 2.2 Hz, 2H), 8.05 (d, *J* = 8.6 Hz, 4H), 7.87 (t, *J* = 5.3 Hz, 1H), 7.72 (dt, *J* = 7.6, 1.2 Hz, 2H), 7.64 (dt, *J* = 7.6, 1.0 Hz, 2H), 7.60 – 7.48 (m, 3H), 3.26 (dt, *J* = 6.0, 5.5 Hz, 2H), 3.11 (dt, *J* = 6.2, 5.8 Hz, 2H), 2.78 (t, *J* = 2.6 Hz, 1H), 2.22 – 2.11 (m, 4H), 1.66 (quint, *J* = 7.3 Hz, 2H), 1.64 – 1.49 (m, 4H); **^{13}C NMR** (75 MHz, DMSO-*d6*) δ (ppm): 171.35, 163.16, 156.28, 146.09, 144.66, 131.98, 128.70, 128.31, 127.90, 127.76, 127.17, 124.11, 84.12, 71.50, 41.76, 38.09, 34.22, 26.78, 25.61, 24.34, 17.44; **LC/MS** (Method A): Retention time 6.09 min, m/z = 600.5 $[M-H]^+$.

Compound 3c

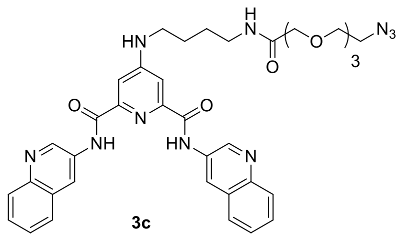

3c

Compound **2** (663.3 mg, 1.312 mmol), 11-azido-3,6,9-trioxaundecanoic acid (459 mg, 1.968 mmol), EDCI (305.5 mg, 1.968 mmol), HOBt (26.9 mg, 0.199 mmol), DMF (13mL). The reaction needs to be carried out by protecting the round bottom flask from the light.

Purification: DCM/EtOH from 100/0 to 90/10, leading to compound **3c** as a white solid (577 mg, 61%). **Mp** = dec. > 48 °C; **^{1}H NMR** (300 MHz, DMSO-*d6*) δ (ppm): 11.28 (s, 2H), 9.36 (d, *J* = 2.4 Hz, 2H), 8.96 (d, *J* = 2.2 Hz, 2H), 8.04 (d, *J* = 8.4 Hz, 4H), 7.78 – 7.68 (m, 3H), 7.63 (dt, *J* = 7.5, 1.0 Hz, 2H), 7.59 – 7.48 (m, 3H), 3.88 (s, 2H), 3.63 – 3.53 (m, 10H), 3.37 (t, *J* = 5.1 Hz, 2H), 3.29 – 3.14 (m, 4H), 1.66 – 1.53 (m, 4H); **^{13}C NMR** (75 MHz, DMSO-*d6*) δ (ppm): 169.14, 163.16, 156.27, 146.08, 144.66, 131.98, 128.70, 128.29, 127.88, 127.76, 127.16, 124.09, 70.25, 70.01, 69.79, 69.68, 69.60, 69.25, 49.98, 41.74, 37.74, 26.84, 25.56; **LC/MS** (Method A): Retention time 6.29 min, m/z = 721.5 $[M-H]^+$.

*Final copper-catalyzed alkyne-azide cycloaddition (CuAAC) precursors **PDC-4,2-Alk**, **PDC-4,3-Alk**, and **PDC-4,PEG-N3** were obtained following the same synthetic protocol*

Compound **3a**, **3b**, or **3c** (1.0 equiv) was dissolved in dry DMF warmed up at 40 °C. Iodomethane (235 equiv) was added and the resulting mixture was stirred at 40 °C for 16 h under argon atmosphere, until completion of the reaction. The reaction mixture was concentrated under reduced pressure and the resulting orange solid was suspended in EtOH, filtered, washed 2x with EtOH, and then 2x with Et_2O.

PDC-4,2-Alk

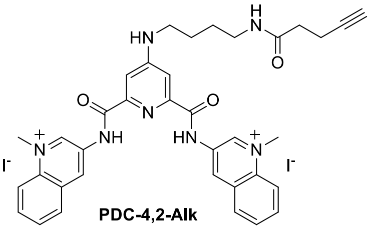

Compound **3a** (300 mg, 0.512 mmol), DMF (3.8 mL), MeI (7.5 mL, 120.4 mmol), leading to **PDC-4,2-Alk** as a yellow solid (425 mg, 95%).

Mp = dec. > 218 °C; **^{1}H NMR** (300 MHz, DMSO-*d6*) δ (ppm): 11.71 (s, 2H), 10.11 (d, *J* = 1.8 Hz, 2H), 9.64 (d, *J* = 1.5 Hz, 2H), 8.58 – 8.52 (m, 4H), 8.24 (dt, *J* = 8.0, 1.0 Hz, 2H), 8.07 (t, *J* = 7.7 Hz, 2H), 7.94 (t, *J* = 5.5 Hz, 1H), 7.70 (t, *J* = 5.1 Hz, 1H), 7.59 (bs, 2H), 4.78 (s, 6H), 3.28 (dt, *J* = 6.0, 5.7 Hz, 2H), 3.13 (dt, *J* = 6.2, 5.9 Hz, 2H), 2.75 (t, *J* = 2.5 Hz, 1H), 2.41 – 2.31 (m, 2H), 2.31 – 2.21 (m, 2H), 1.70 – 1.48 (m, 4H); **^{13}C NMR** (75 MHz, DMSO-*d6*) δ (ppm): 170.15, 163.21, 156.55, 144.73, 135.68, 134.13, 133.92, 132.34, 130.34, 129.86, 129.22, 119.23, 99.51, 83.79, 71.29, 46.17, 41.75, 38.06, 34.27, 26.74, 25.33, 14.32; **LC/MS** (Method A): Retention time 5.07 min, m/z = 614.5 $[M-H]^+$; **HRMS** (ESI, *m/z*) calcd. for formula $C_{36}H_{37}N_7O_3I$ $[M-I]^+$ = 742.2003; found = 742.1992.

PDC-4,3-Alk

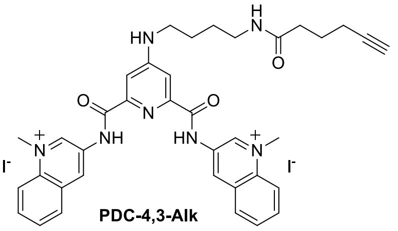

Compound **3b** (300 mg, 0.500 mmol), DMF (3.8 mL), MeI (7.3 mL, 117.6 mmol), leading to **PDC-4,3-Alk** as a yellow solid (411 mg, 93%).

Mp = dec. > 218 °C; **^{1}H NMR** (300 MHz, DMSO-*d6*) δ (ppm): 11.71 (s, 2H), 10.11 (d, *J* = 1.8 Hz, 2H), 9.64 (d, *J* = 1.5 Hz, 2H), 8.55 (d, *J* = 9.3 Hz, 4H), 8.24 (dt, *J* = 8.0, 1.0 Hz, 2H), 8.08 (t, *J* = 7.0 Hz, 2H), 7.88 (t, *J* = 5.6 Hz, 1H), 7.71 (t, *J* = 5.1 Hz, 1H), 7.60 (bs, 2H), 4.78 (s, 6H), 3.26 (dt, *J* = 6.0, 5.6 Hz, 2H), 3.11 (dt, *J* = 6.3, 5.9 Hz, 2H), 2.78 (t, *J* = 2.6 Hz, 1H), 2.22 – 2.09 (m, 4H), 1.67 (quint, *J* = 7.3 Hz, 2H), 1.63 – 1.50 (m, 4H); **^{13}C NMR** (75 MHz, DMSO-*d6*) δ (ppm): 171.34, 163.16, 156.50, 144.69, 135.62, 134.02, 133.88, 132.33, 130.29, 129.83, 129.18, 119.18, 84.07, 71.50, 46.16, 41.74, 38.01, 34.18, 26.77, 25.47, 24.30, 17.39; **LC/MS** (Method A): Retention time 4.57 min, m/z = 628.5 $[M-H]^+$; **HRMS** (ESI, *m/z*) calcd. for formula $C_{37}H_{39}N_7O_3I$ $[M-I]^+$ = 756.2159; found = 756.2161.

PDC-4,PEG-N3

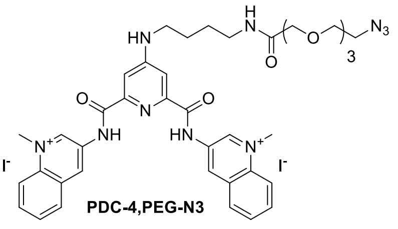

Compound **3c** (450 mg, 0.624 mmol), DMF (4.5 mL), MeI (9.1 mL, 146.7 mmol), leading to **PDC-4,PEG-N$_3$** as a yellow solid (478 mg, 76%). The reaction needs to be carried out by protecting the round bottom flask from the light.

Mp = dec. > 185 °C; **^{1}H NMR** (300 MHz, DMSO-*d6*) δ (ppm): 11.71 (s, 2H), 10.13 (d, *J* = 0.9 Hz, 2H), 9.65 (d, *J* = 0.9 Hz, 2H), 8.55 (d, *J* = 8.7 Hz, 4H), 8.23 (t, *J* = 7.9 Hz, 2H), 8.08 (t, *J* = 7.5 Hz, 2H), 7.79 – 7.66 (m, 2H), 7.59 (bs, 2H), 4.78 (s, 6H), 3.89 (s, 2H), 3.63 – 3.53 (m, 10H), 3.39 (t, *J* = 4.9 Hz, 2H), 3.30 – 3.25 (m, 2H), 3.23 – 3.15 (m, 2H), 1.60 (m, 4H); **^{13}C**

NMR (75 MHz, DMSO-*d6*) δ (ppm): 169.11, 163.19, 156.53, 144.72, 135.67, 134.11, 133.91, 132.37, 130.33, 129.84, 129.21, 119.21, 99.49, 70.22, 69.97, 69.76, 69.64, 69.55, 69.21, 49.95, 46.14, 41.72, 37.66, 26.80, 25.43; **LC/MS** (Method A): Retention time 4.79 min, m/z = 749.5 $[M-H]^+$; **HRMS** (ESI, *m/z*) calcd. for formula $C_{39}H_{46}N_{10}O_6I$ $[M-I]^+$ = 877.2647; found = 877.2668.

Synthetic protocol followed for the preparation of ***PDC-4,0-N3***

Compound 4

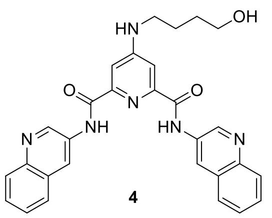

Compound **1** (600 mg, 1.322 mmol, 1.0 equiv) mixed with 4-amino-1-butanol (2.3 mL, 24.77 mmol, 18.7 equiv) and TEA (0.5 mL, 3.597 mmol, 2.7 equiv) was heated at 90 °C under argon atmosphere for 16 h. After completion of the reaction, the crude product was directly purified by SiO_2 gel chromatography and eluted with a gradient of DCM/MeOH from 100/0 to 90/10, affording compound **4** as a white solid (441 mg, 66%).

Mp = 150 °C; ^{1}**H NMR** (300 MHz, DMSO-*d6*) δ (ppm): 11.23 (s, 2H), 9.36 (d, *J* = 2.3 Hz, 2H), 8.94 (d, *J* = 1.9 Hz, 2H), 8.08 – 7.99 (m, 4H), 7.71 (t, *J* = 7.6 Hz, 2H), 7.63 (t, *J* = 7.4 Hz, 2H), 7.57 (bs, 2H), 7.44 (t, *J* = 5.0 Hz, 1H), 4.38 (t, *J* = 4.8 Hz, 1H), 3.48 (dt, *J* = 5.8, 5.2 Hz, 2H), 3.30 – 3.25 (m, 2H), 1.73 – 1.52 (m, 4H); ^{13}**C NMR** (75 MHz, DMSO-*d6*) δ (ppm): 163.20, 156.33, 146.13, 144.68, 131.99, 128.72, 128.36, 127.93, 127.78, 127.22, 124.17, 60.44, 42.00, 29.95, 24.96; **LC/MS** (Method A): Retention time 5.48 min, m/z = 507.5 $[M-H]^+$.

Compound 5

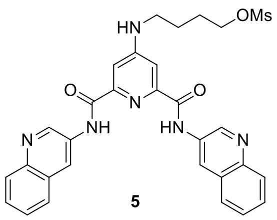

Compound **4** (40 mg, 0.079 mmol, 1.0 equiv) was suspended in dry DCM (2.4 mL) at 0 °C under argon atmosphere and freshly distilled TEA (0.099 mL, 0.712 mmol, 9.0 equiv) was added under stirring. Reaction mixture was cooled down to 0°C and MsCl (0.027 mL, 0.355 mmol, 4.5 equiv) was added dropwise. The mixture was stirred at 0°C for 0.5h and then left to warm up to room temperature. The reaction was quenched by addition of ice water and a precipitate formed. The latter was filtered and the resulting white solid washed 3x with small portions of water and then once with Et_2O. The solid was dried under vacuum, yielding compound **5** as a white solid (31 mg, 67%). Once the ^{1}H NMR was recorded, the product was directly submitted to the next reaction.

^{1}**H NMR** (300 MHz, DMSO-*d6*) δ (ppm): 11.36 (s, 2H), 9.42 (d, *J* = 2.3 Hz, 2H), 9.03 (d, *J* = 1.8 Hz, 2H), 8.10 – 8.03 (m, 4H), 7.75 (dt, *J* = 7.1, 1.0 Hz, 2H), 7.66 (t, *J* = 7.2 Hz, 2H), 7.62 – 7.52 (m, 3H), 4.28 (t, *J* = 6.1 Hz, 2H), 3.34 – 3.26 (m, 2H), 3.20 (s, 3H), 1.89 – 1.65 (m, 4H); **LC/MS** (Method A): Retention time 6.14 min, m/z = 585.5 $[M-H]^+$.

Compound 6

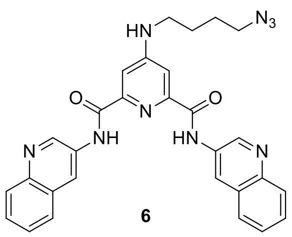

Compound **5** (350 mg, 0.599 mmol, 1.0 equiv) was directly submitted to nucleophilic substitution with NaN_3 (389.2 mg, 5.99 mmol, 10 equiv) in dry DMF (23 mL) at 100 °C for 1 h 30 min. The reaction must be protected from light. DMF was removed under reduced pressure and the resulting yellow solid was washed 3x with water and then once with Et_2O. The crude product was purified by SiO_2 gel chromatography using a gradient of DCM/EtOH from 100/0 to 90/10, yielding compound **6** as a pale yellow solid (332 mg, 77%).

Mp = 132 °C; **^{1}H NMR** (300 MHz, DMSO-*d6*) δ (ppm): 11.29 (s, 2H), 9.35 (d, *J* = 2.5 Hz, 2H), 8.96 (d, *J* = 2.3 Hz, 2H), 8.04 (d, *J* = 9.3 Hz, 4H), 7.72 (dt, *J* = 7.6, 1.4 Hz, 2H), 7.63 (dt, *J* = 7.8, 1.0 Hz, 2H), 7.60 – 7.50 (m, 3H), 3.43 (t, *J* = 6.3 Hz, 2H), 3.32 – 3.24 (m, 2H), 1.75 – 1.62 (m, 4H); **^{13}C NMR** (75 MHz, DMSO-*d6*) δ (ppm): 163.14, 156.30, 146.09, 144.65, 131.95, 128.68, 128.32, 127.89, 127.74, 127.18, 124.13, 50.39, 41.51, 25.91, 25.41; **LC/MS** (Method A): Retention time 6.88 min, m/z = 532.5 $[M-H]^+$.

PDC-4,0-N3

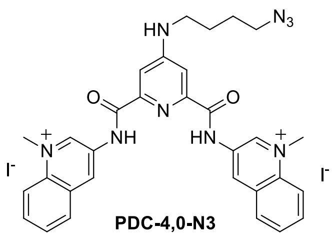

Compound **6** (220 mg, 0.414 mmol, 1.0 equiv) was dissolved in dry DMF (3.0 mL) at 40 °C under argon atmosphere and protected from light. Iodomethane (6.4 mL, 103.5 mmol, 250 equiv) was then added. The mixture was stirred at 40 °C for 16 h till completion of the reaction. The mixture was concentrated under reduced pressure and the resulting orange solid was suspended in EtOH, filtered, washed 2x with EtOH, and then 2x with Et_2O, leading to **PDC-4,0-N3** as a yellow solid (292 mg, 87%).

Mp = dec. > 215 °C; **^{1}H NMR** (300 MHz, DMSO-*d6*) δ (ppm): 11.66 (s, 2H), 10.12 (d, *J* = 1.9 Hz, 2H), 9.64 (s, 2H), 8.60 – 8.48 (m, 4H), 8.24 (dt, *J* = 8.0, 1.0 Hz, 2H), 8.08 (t, *J* = 7.6 Hz, 2H), 7.65 (t, *J* = 5.4 Hz, 1H), 7.64 – 7.58 (m, 2H), 4.79 (s, 6H), 3.43 (t, *J* = 5.9 Hz, 2H), 3.35 – 3.28 (m, 2H), 1.75 – 1.65 (m, 4H); **^{13}C NMR** (75 MHz, DMSO-*d6*) δ (ppm) 163.21, 156.56, 144.78, 135.66, 134.12, 133.92, 132.38, 130.34, 129.87, 129.21, 119.22, 50.39, 46.15, 41.55, 25.90, 25.33; **LC/MS** (Method A): Retention time 5.17 min, m/z = 560.5 $[M-H]^+$; **HRMS** (ESI, *m/z*) calcd. for formula $C_{31}H_{31}N_9O_2I$ $[M-I]^+$ = 688.1645; found = 688.1622.

*Synthetic protocol followed for the preparation of **5-BrdU-Alk***

Compound 7

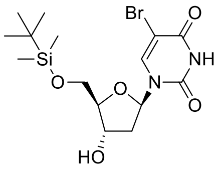
7

Commercially available 5-bromo-2'-deoxyuridine (**5-BrdU**, 1.00 g, 3.256 mmol, 1.0 equiv) was mixed with DMAP (51.7 mg, 0.423 mmol, 0.13 equiv) and imidazole (731.5 mg, 10.75 mmol, 3.3 equiv) in dry DMF (10 mL) under argon atmosphere. The mixture was cooled down to 0 °C and TBDMSCl (539.9 mg, 3.582 mmol, 1.1 equiv) dissolved in dry DMF (5 mL) was added dropwise. The reaction mixture was stirred at 0 °C for 30 min and then slowly warmed up to room temperature and stirred for 16 h. Afterwards, a saturated solution of $NaHCO_3$ was added and the aqueous phase was extracted 3x with AcOEt. The combined organic layers were washed once with water, dried over $MgSO_4$, filtered, and concentrated under reduced pressure. The resulting crude solid was purified by SiO_2 gel chromatography using a gradient of DCM/EtOH from 100/0 to 95/5, yielding compound **7** as a white solid (1.081 g, 79%).

Mp = dec. > 72 °C; **^{1}H NMR** (300 MHz, DMSO-*d6*) δ (ppm): 11.85 (s, 1H), 8.01 (s, 1H), 6.10 (dd, *J* = 7.3, 6.3 Hz, 1H), 5.29 (d, *J* = 4.1 Hz, 1H), 4.22 – 4.15 (m, 1H), 3.87 (dd, *J* = 5.3, 2.7 Hz, 1H), 3.82 (dd, *J* = 11.5, 2.7 Hz, 1H), 3.73 (dd, *J* = 11.5, 3.3 Hz, 1H), 2.21 – 2.02 (m, 2H), 0.89 (s, 9H), 0.10 (d, *J* = 2.5 Hz, 6H); **^{13}C NMR** (75 MHz, DMSO-*d6*) δ (ppm): 159.13, 149.67, 139.39, 95.93, 87.31, 85.10, 70.50, 63.19, 25.94, 18.13, -5.35; **LC/MS** (Method A): Retention time 6.58 min, m/z = 423.2 $[M-H]^+$.

159.13, 149.67, 139.39, 95.93, 87.31, 85.10, 70.50, 63.19, 25.94, 18.13, -5.35

Compound 8

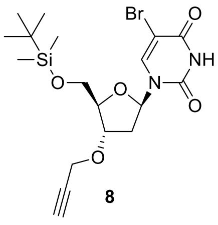
8

Compound **7** (500 mg, 1.187 mmol, 1.0 equiv) was dissolved in anhydrous THF (22.5 mL) at room temperature and under argon atmosphere. NaH (71.2 mg, 2.967 mmol, 2.5 equiv) was then added portion wise. The mixture was stirred 30 min and propargyl bromide (0.264 mL, 2.967 mmol, 2.5 equiv, 80 wt % in toluene) was added dropwise. The reaction mixture was stirred for additional 16 h. EtOH was added to quench the reaction and the solvent was removed under reduced pressure. The resulting crude product was purified by SiO_2 gel chromatography using a gradient of DCM/EtOH from 100/0 to 95/5, affording compound **8** as a white solid (450 mg, 83%).

Mp = dec. > 45 °C; **^{1}H NMR** (300 MHz, DMSO-*d6*) δ (ppm): 11.87 (s, 1H), 8.02 (s, 1H), 6.04 (dd, *J* = 8.2, 5.8 Hz, 1H), 4.26 – 4.21 (m, 3H), 4.06 (dd, *J* = 5.1, 3.1 Hz, 1H), 3.83 (dd, *J* = 11.4, 3.4 Hz, 1H), 3.75 (dd, *J* = 11.4, 3.3 Hz, 1H), 3.48 (t, *J* = 2.3 Hz, 1H), 2.35 (ddd, *J* = 13.6, 5.7, 1.6 Hz, 1H), 2.12 (ddd, *J* = 13.9, 8.2, 5.9 Hz, 1H), 0.90 (s, 9H), 0.12 (d, *J* = 2.0 Hz, 6H); **^{13}C NMR** (75 MHz, DMSO-*d6*) δ (ppm): 159.07, 149.69, 139.27, 96.14, 85.01, 84.40, 80.01, 78.22, 77.48, 63.23, 55.84, 36.88, 25.90, 18.07, -5.40; **LC/MS** (Method B): Retention time 7.10 min, m/z = 461.2 $[M-H]^+$.

5-BrdU-Alk

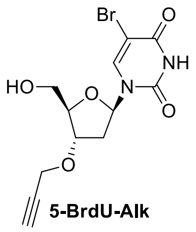

5-BrdU-Alk

Compound **8** (400 mg, 0.871 mmol, 1.0 equiv) was solubilized in THF anhydrous (8 mL) and TBAF (1.05 mL, 1.045 mmol, 1.2 equiv, 1M in THF) was slowly added at room temperature. The resulting mixture was stirred under argon atmosphere for 16 h. The solvent was then removed under vacuum and the crude was purified by SiO_2 gel chromatography, eluted with a gradient of DCM/EtOH from 100/0 to 90/10, affording **5-BrdU-Alk** as a white solid (224.5 mg, 75%).

Mp = 157 °C; **^{1}H NMR** (300 MHz, DMSO-*d6*) δ (ppm): 11.84 (s, 1H), 8.37 (s, 1H), 6.06 (dd, *J* = 7.6, 6.1 Hz, 1H), 5.26 (t, *J* = 4.8 Hz, 1H), 4.28 – 4.22 (m, 1H), 4.21 (d, *J* = 2.3 Hz, 2H), 3.99 (dd, *J* = 2.9, 2.6 Hz, 1H), 3.70 – 3.54 (m, 2H), 3.49 (t, *J* = 2.4 Hz, 1H), 2.32 (ddd, *J* = 13.9, 6.1, 2.6 Hz, 1H), 2.19 (ddd, *J* = 13.7, 7.6, 6.0 Hz, 1H); **^{13}C NMR** (75 MHz, DMSO-*d6*) δ (ppm): 159.13, 149.75, 140.13, 95.94, 84.85, 84.80, 80.23, 78.35, 77.35, 61.15, 55.96, 36.85; **LC/MS** (Method A): Retention time 4.72 min, m/z = 347.1 $[M-H]^+$; **HRMS** (ESI, *m/z*) calcd. for formula $C_{12}H_{14}N_2O_5Br$ $[M-H]^+$ = 345.0086; found = 345.0096.

Synthetic protocol followed for the preparation of ***5-BrdU-N3***

Compound 9

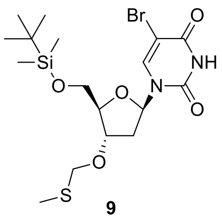

9

To compound **7** (1.00 g, 2.373 mmol, 1.0 equiv) was added a mixture of DMSO (7.52 mL, 105.8 mmol, 44.6 equiv), acetic acid (1.58 mL, 27.6 mmol, 11.6 equiv), and acetic anhydride (5.14 mL, 54.75 mmol, 23.1 equiv). The resulting mixture was stirred at room temperature for 48 h. To quench the reaction, the mixture was poured into a cold saturated solution of $NaHCO_3$. The pH was adjusted to 10 by adding portions of solid $NaHCO_3$ and the resulting aqueous solution was extracted 3x with DCM. The combined organic layers were then dried over $MgSO_4$, filtered, and the solvent removed under reduced pressure. The resulting crude product was purified by SiO_2 gel chromatography eluted with a gradient of DCM/EtOH from 100/0 to 99/1, yielding compound **9** as a white solid (987 mg, 86%).

Mp = dec. > 45 °C; **^{1}H NMR** (300 MHz, DMSO-*d6*) δ (ppm): 11.87 (s, 1H), 8.02 (s, 1H), 6.06 (dd, *J* = 8.1, 5.9 Hz, 1H), 4.71 (s, 2H), 4.41 – 4.35 (m, 1H), 4.04 (dd, *J* = 5.3, 3.1 Hz, 1H), 3.83 (dd, *J* = 11.3, 3.7 Hz, 1H), 3.76 (dd, *J* = 11.4, 3.5 Hz, 1H), 2.32 (ddd, *J* = 13.8, 5.9, 2.1 Hz, 1H), 2.21 – 2.12 (m, 1H), 2.09 (s, 3H), 0.90 (s, 9H), 0.11 (d, *J* = 1.8 Hz, 6H); **^{13}C NMR** (75 MHz, DMSO-*d6*) δ (ppm): 159.07, 149.67, 139.31, 96.16, 85.07, 84.54, 75.93, 72.62, 63.15, 36.95, 25.90, 18.06, 13.30, -5.40[2] **LC/MS** (Method A): Retention time 7.97 min, m/z = 483.2 $[M-H]^+$.

Compound 10

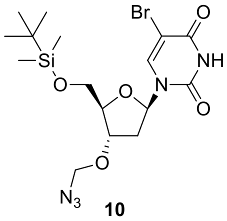

10

To a stirred solution of compound **9** (380 mg, 0.789 mmol, 1.0 equiv) in dry DCM (2.4 mL) cooled at -10 °C was added dropwise a solution of Br_2 (48.7 µL, 0.947 mmol, 1.2 equiv) in dry DCM (1.2 mL). The mixture was stirred at -10 °C for 30 min sheltered from light. A suspension of NaN_3 (308 mg, 4.735 mmol, 6.0 equiv) in dry DMF (15 mL) was then added. The reaction mixture was slowly warmed up to room temperature and kept under stirring for 16 h. The reaction was quenched by addition of a saturated solution of $NaHCO_3$ and the resulting solution was extracted 3x with DCM. The combined organic layers were then dried over $MgSO_4$, filtered, and the solvent was removed under reduced pressure. The resulting yellow oil was then purified by SiO_2 gel chromatography eluted with a gradient of DCM/EtOH from 100/0 to 99/1, yielding compound **10** as a colorless oil (206 mg, 55%).

^{1}H NMR (300 MHz, DMSO-*d6*) δ (ppm): 11.89 (s, 1H), 8.01 (s, 1H), 6.07 (dd, *J* = 7.4, 6.2 Hz, 1H), 4.88 (d, *J* = 9.0 Hz, 1H), 4.85 (d, *J* = 9.0 Hz, 1H), 4.34 – 4.29 (m, 1H), 4.05 (dd, *J* = 5.7, 3.0 Hz, 1H), 3.84 (dd, *J* = 11.4, 3.5 Hz, 1H), 3.77 (dd, *J* = 11.4, 3.5 Hz, 1H), 2.37 (ddd, *J* = 13.7, 5.9, 2.5 Hz, 1H), 2.30 – 2.18 (m, 1H), 0.90 (s, 9H), 0.11 (d, *J* = 1.4 Hz, 6H); **^{13}C NMR** (75 MHz, DMSO-*d6*) δ (ppm): 159.14, 149.70, 139.39, 96.17, 84.97, 84.68, 80.99, 78.07, 62.96, 37.30, 25.91, 18.09, -5.39; **LC/MS** (Method B): Retention time 7.25 min, m/z = 478.3 $[M-H]^+$.

5-BrdU-N3

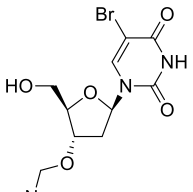

5-BrdU-N3

Compound **10** (160 mg, 0.336 mmol, 1.0 equiv) was solubilized in anhydrous THF (3.2 mL) and TBAF (0.403 mL, 0.403 mmol, 1.2 equiv, 1M in THF) was slowly added at room temperature. The resulting mixture was stirred under argon atmosphere protected from the light for 16 h. The solvent was then removed under vacuum and the crude product was purified by SiO_2 gel chromatography, eluted with a gradient of DCM/EtOH from 100/0 to 90/10, affording **5-BrdU-N3** as a white solid (75 mg, 62%).

Mp = 156 °C; **^{1}H NMR** (300 MHz, DMSO-*d6*) δ (ppm): 11.75 (bs, 1H), 8.36 (s, 1H), 6.08 (t, *J* = 6.6 Hz, 1H), 5.28 (t, *J* = 4.6 Hz, 1H), 4.85 (s, 2H), 4.39 – 4.33 (m, 1H), 3.99 (dd, *J* = 6.3, 3.0 Hz, 1H), 3.70 – 3.57 (m, 2H), 2.35 – 2.26 (m, 2H); **^{13}C NMR** (75 MHz, DMSO-*d6*) δ (ppm) 159.20, 149.78, 140.18, 95.93, 85.06, 84.77, 81.11, 77.93, 60.84, 37.46; **LC/MS** (Method A): Retention time 4.78 min, m/z = 364.0 $[M\text{-}H]^+$; **HRMS** (ESI, *m/z*) calcd. for formula $C_{10}H_{13}N_5O_5Br$ $[M\text{-}H]^+$ = 362.0100; found = 362.0098.

Synthetic pathway followed for the preparation of final compounds PDC-4,0-BrdU, PDC-4,2-BrdU, PDC-4,3-BrdU, and PDC-4,PEG-BrdU obtained from copper-catalyzed alkyne-azide cycloaddition (CuAAC)

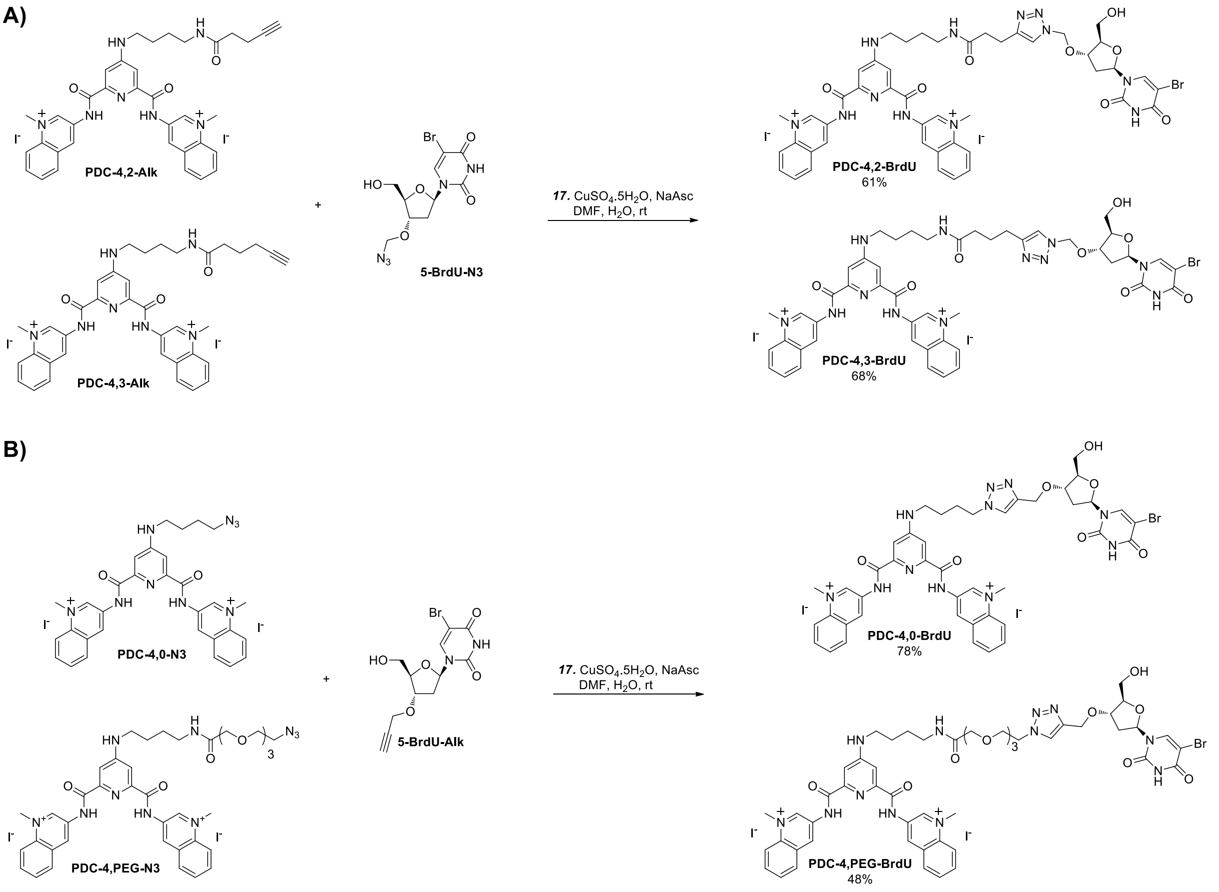

Scheme S1. Synthetic pathway exploited for the preparation of final compounds A) PDC-4,2-BrdU and PDC-4,3-BrdU and B) PDC-4,0-BrdU and PDC-4,PEG-BrdU.

Reagents and conditions: $CuSO_4.5H_2O$ (0.3 equiv), NaAsc (1.2 equiv), DMF, H_2O, rt, 4 - 16 h, under argon atmosphere.

NaAsc = sodium ascorbate

All final compounds were obtained using the following general protocol:

PDC CuAAC precursor (1.0 equiv) was mixed with the appropriate 5-BrdU derivative (1.2 equiv) in DMF/distilled water 6/2 mL (3/1 (v/v) after addition of every reagents). The mixture was protected from light, stirred and purged with argon for 15 min. A solution of sodium ascorbate in water (1.2 equiv, 0.1 M) was then added followed by a solution of $CuSO_4.5H_2O$ in water (0.3 equiv, 0.1 M). The solution was purged for additional 15 min with argon and then stirred under argon atmosphere until completion of the reaction. Copper(II) ions were removed from the reaction mixture by adding Chelex® resin 100 Na-form, previously conditioned to pH = 7 with triethylammonium acetate (50 mM). The suspension was gently shaken for 30 min and then filtered through a cotton pad. The Chelex resin was washed twice

with DMF and the resulting mixture was concentrated under reduced pressure using a SpeedVac Vacuum Concentrator to give the crude product as a brown sticky oil. Crude product was precipitated in DCM, filtered, washed several times with DCM and then with water. The brown solid was then dried under vacuum yielding the expected compound.

PDC-4,0-BrdU

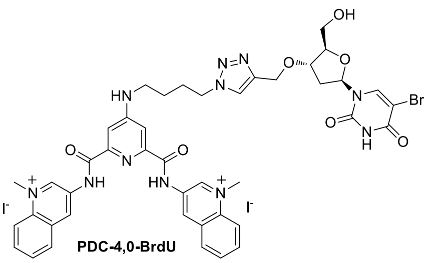

PDC-4,0-N3 (70 mg, 0.085 mmol, 1.0 equiv), **5-BrdU-Alk** (35.6 mg, 0.102 mmol, 1.2 equiv), DMF (6 mL), water (0.75 mL), sodium ascorbate (1.02 mL, 0.10 mmol, 1.2 equiv, 0.1 M), $CuSO_4.5H_2O$ (257.5 µL, 0.026 mmol, 0.3 equiv, 0.1 M). The reaction was complete within 4 h. Work up steps led to final compound **PDC-4,0-BrdU** as a brown solid (78 mg, 78%).

Mp = dec. > 200 °C; **^{1}H NMR** (300 MHz, DMSO-d6) δ (ppm): 11.70 (s, 2H), 10.21 (bs, 2H), 9.69 (bs, 2H), 8.40 (bs, 4H), 8.32 (s, 1H), 8.24 – 8.10 (m, 3H), 8.00 (bs, 2H), 7.70 – 7.30 (m, 3H), 6.06 (t, *J* = 6.7 Hz, 1H), 5.14 (bs, 1H), 4.69 (bs, 6H), 4.60 (s, 2H), 4.47 (t, *J* = 6.7 Hz, 2H), 4.24 (bs, 1H), 4.01 (bs, 1H), 3.68 – 3.56 (m, 2H), 2.16 – 2.25 (m, 2H), 2.08 – 1.95 (2H), 1.72 – 1.54 (m, 2H); **LC/MS** (Method B): Retention time 4.61 min, m/z = 906.4 $[M]^+$; **HRMS** (ESI, *m/z*) calcd. for formula $C_{43}H_{44}N_{11}O_7BrI$ $[M-I]^+$ = 1032.1653; found = 1032.1667.

PDC-4,2-BrdU

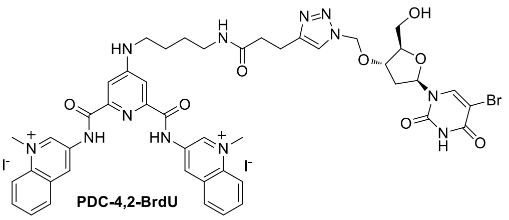

PDC-4,2-Alk (70 mg, 0.081 mmol, 1.0 equiv), **5-BrdU-N3** (35.0 mg, 0.086 mmol, 1.2 equiv), DMF (6 mL), water (0.75 mL), sodium ascorbate (0.97 mL, 0.97 mmol, 1.2 equiv, 0.1 M), $CuSO_4.5H_2O$ (241 µL, 0.024 mmol, 0.3 equiv, 0.1 M). The reaction was complete within 16 h. Work up steps led to final compound **PDC-4,2-BrdU** as a brown solid (60 mg, 61%).

Mp = dec. > 190 °C; **^{1}H** NMR (500 MHz, DMSO-d6) δ (ppm): 11.69 (s, 2H), 10.17 (bs, 2H), 9.70 (bs, 2H), 8.43 (bs, 4H), 8.27 (s, 1H), 8.15 (bs, 2H), 8.00 (bs, 2H), 7.97 (s, 1H), 7.81 (bs, 1H), 7.73 (bs, 1H), 7.65 – 7.40 (m, 2H), 6.03 (t, *J* = 6.6 Hz, 1H), 5.75 (s, 2H), 5.13 (bs, 1H), 4.70 (bs, 6H), 4.32 (bs, 1H), 3.87 (bs, 1H), 3.58 (dd, *J* = 11.6, 2,8 Hz, 1H), 3.51 (dd, *J* = 11.6, 2.3 Hz, 1H), 2.91 (t, *J* = 7.2 Hz, 2H), 2.16 – 2.11 (m, 2H), 1.67 – 1.51 (m, 4H); **LC/MS** (Method B): Retention time 4.56 min, m/z = 977.6 $[M]^+$; **HRMS** (ESI, *m/z*) calcd. for formula $C_{46}H_{49}N_{12}O_8BrI$ $[M-I]^+$ = 1103,2024; found = 1103.2054.

PDC-4,3-BrdU

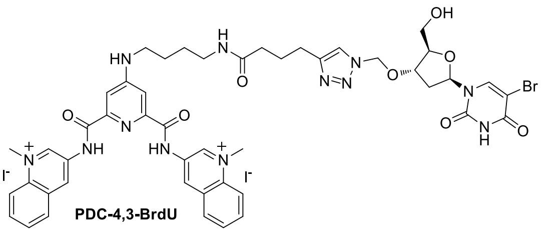

PDC-4,3-Alk (80 mg, 0.091 mmol, 1.0 equiv), **5-BrdU-N3** (39.4 mg, 0.108 mmol, 1.2 equiv, DMF (6 mL), water (0.65 mL), sodium ascorbate (1.08 mL, 0.108 mmol, 1.2 equiv, 0.1 M), $CuSO_4.5H_2O$ (271 µL, 0.027 mmol, 0.3 equiv, 0.1 M). The reaction was complete within 16 h. Work up steps led to final compound **PDC-4,3-BrdU** as a brown solid (77 mg, 68%).

Mp = dec. > 182 °C; **^{1}H NMR** (300 MHz, DMSO-d6) δ (ppm): 11.70 (s, 2H), 10.14 (bs, 2H), 9.67 (bs, 2H), 8.47 (bs, 4H), 8.28 (s, 1H), 8.24 – 8.04 (m, 4H), 8.02 (s, 1H), 7.74 (t, *J* = 5.3 Hz, 1H), 7.70 – 7.40 (m, 3H), 6.03 (t, *J* = 6.7 Hz, 1H), 5.75 (s, 2H), 5.14 (bs, 1H), 4.73 (bs, 6H), 4.32 (bs, 1H), 3.87 (bs, 1H), 3.60 – 3.50 (m, 2H), 2.65 (t, *J* = 7.4 Hz, 2H), 2.19 – 2.10 (m, 4H), 1.86 (quint, *J* = 7.3 Hz, 2H), 1.70 – 1.50 (m, 4H); **LC/MS** (Method B): Retention time 4.66 min, m/z = 991.7 $[M]^+$; **HRMS** (ESI, *m/z*) calcd. for formula $C_{47}H_{51}N_{12}O_8BrI$ $[M-I]^+$ = 1117.2181; found = 1117.2233.

PDC-4,PEG-BrdU

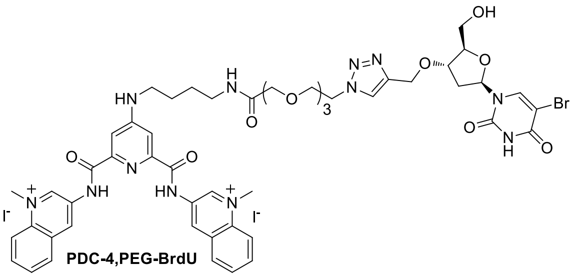

PDC-4,PEG-N3 (70 mg, 0.070 mmol, 1.0 equiv), **5-BrdU-Alk** (28.9 mg, 0.084 mmol, 1.2 equiv), DMF (6 mL), water (1.0 mL), sodium ascorbate (0.836 mL, 0.836 mmol, 1.2 equiv, 0.1 M), $CuSO_4.5H_2O$ (209 µL, 0.021 mmol, 0.3 equiv, 0.1 M). The reaction was complete within 6 h. Work up steps led to final compound **PDC-4,PEG-BrdU** as a brown solid (45 mg, 48%).

Mp = dec. > 148 °C; **^{1}H** NMR (300 MHz, DMSO-d6) δ (ppm): 11.70 (s, 2H), 10.11 (bs, 2H), 9.64 (bs, 2H), 8.51 (bs, 4H), 8.32 (s, 1H), 8.22 (bs, 2H), 8.10 – 8.00 (m, 3H), 7.70 – 7.50 (m, 3H), 6.06 (t, *J* = 6.6 Hz, 1H), 5.13 (bs, 1H), 4.76 (bs, 6H), 4.58 (bs, 2H), 4.52 (t, *J* = 5.1 Hz, 2H), 4.22 (bs, 1H), 4.00 (bs, 1H), 3.89 (bs, 2H), 3.83 (t, *J* = 4.90 Hz, 2H), 3.65 – 3.50 (m, 10H), 2.25 – 2.15 (m, 2H), 1.70 – 1.52 (m, 4H); **LC/MS** (Method B): Retention time 4.64 min, m/z = 1095.6 $[M]^+$; **HRMS** (ESI, *m/z*) calcd. for formula $C_{51}H_{59}N_{12}O_{11}BrI$ $[M-I]^+$ = 1221.2654; found = 1221.2644.

Table S1. Doubly-labeled sequences used for FRET experiments

Sequence name	Sequence (5'→3')
F21T	F-GGGTTAGGGTTAGGGTTAGGG-T(3)
FcMyc22T (G14T-G23T)	F-TTGAGGGTGGGTAGGGTGGGTAA-T(4)
FCEB25wtT	F-AGGGTGGGTGTAAGTGTGGGTGGG-T(5)
FCEB25L111TT	F-AGGGTGGGTGGGTGGGT-T(6)
F21CTAT	F-GGGCTAGGGCTAGGGCTAGGG-T(7)
FKit2T	F-GGGCGGGCGCGAGGGAGGGG-T(8)
F21RT	F-r(GGGUUAGGGUUAGGGUUAGGG)-T(9)
F-NRAS-T	F-r(GGGAGGGGCGGGUCUGGG)-T(10)

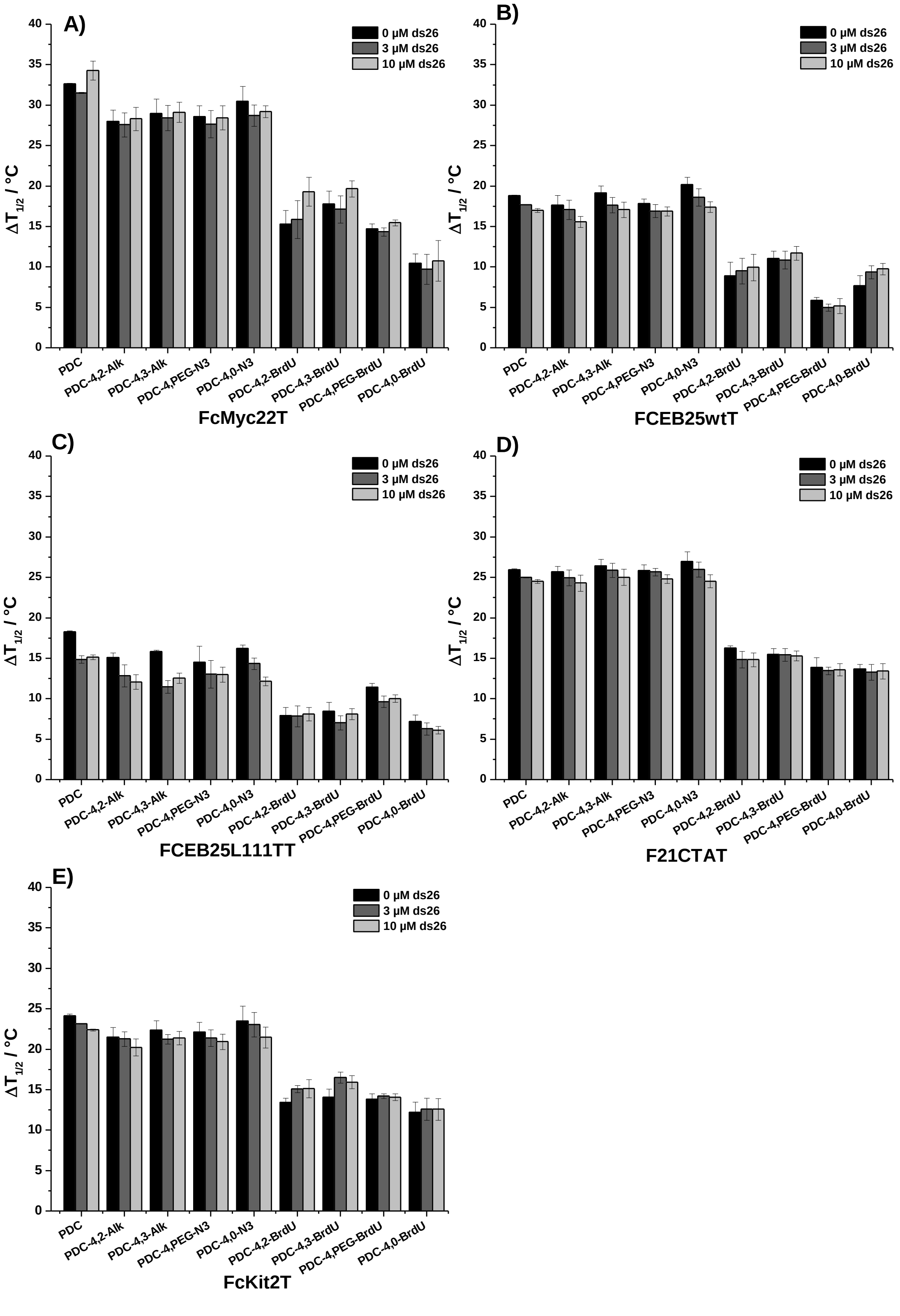

Figure S1: Quantitative analysis of the FRET-melting competition experiments with A) c-Myc oncogene sequence FcMyc22T, B) human minisatellite repeat native sequence FCEB25wtT, C) human minisatellite repeat modified sequence FCEB25L111TT, D) human telomeric sequence variant F21CTAT, and E) c-kit2 oncogene promoter (Fkit2T). G4 structure concentration 0.2 µM. Stabilization in K^+-rich buffer is indicated for the reference compound PDC, and compounds PDC-4,2-Alk, PDC-4,3-Alk, PDC-4,PEG-N3, PDC-4,0-N3, PDC-4,2-BrdU,

PDC-4,3-BrdU, PDC-4,PEG-BrdU, and PDC-4,0-BrdU in the absence (dark bars) or in the presence of double-stranded DNA (ds26) at 3 µM (grey bars) or 10 µM (light grey bars). Concentration analyzed compounds: 1 µM. Error bars corresponds to SD of three independent experiments. Experiments were performed in D) 10 mM lithium cacodylate buffer (pH 7.2), 90 mM LiCl, and 10 mM KCl and A-C) and E) 10 mM lithium cacodylate buffer (pH 7.2), 99 mM LiCl, and 1 mM KCl.

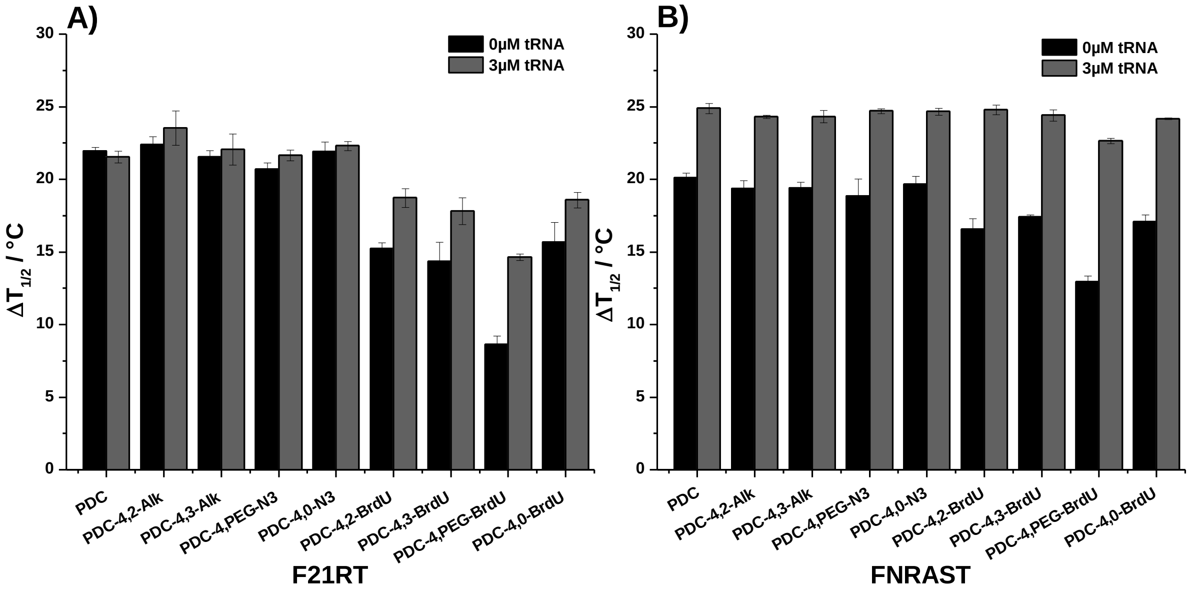

Figure S2: Quantitative analysis of the FRET-melting competition experiments with A) RNA human telomeric sequence F21RT, and B) RNA G4-forming sequence of the human NRAS proto-oncogene transcript FNRAST. G4 structure concentration 0.2 µM. Stabilization in K^+-rich buffer is indicated for the reference compound PDC, and compounds PDC-4,2-Alk, PDC-4,3-Alk, PDC-4,PEG-N3, PDC-4,0-N3, PDC-4,2-BrdU, PDC-4,3-BrdU, PDC-4,PEG-BrdU, and PDC-4,0-BrdU in the absence (black bars) or in the presence of *ss*RNA competitor (tRNA) at 3 µM (grey bars). Concentration analyzed compounds: 1 µM. Error bars corresponds to SD of three independent experiments. Experiments were performed in 10 mM lithium cacodylate buffer (pH 7.2), 99 mM LiCl, and 1 mM KCl.

Table S2. Sequences used for G4-FID experiments

Sequence name	Sequence (5’→3’)	Conformation
22AG	AGGGTTAGGGTTAGGGTTAGGG	Hybrid G4(3)
c-Myc22 (G14T-G23T)	TGAGGGTGGGTAGGGTGGGTAA	Parallel(4)
CEB25wt	AAGGGTGGGTGTAAGTGTGGGTGGGT	Parallel(5)
CEB25L111T	AAGGGTGGGTGGGTGGGT	Parallel(6)
21CTA	GGGCTAGGGCTAGGGCTAGGG	Anti-parallel(7)
cKit2	GGGCGGGCGCGAGGGAGGGG	Parallel(8)
TERRA	r(AGGGUUAGGGUUAGGGUUAGGG)	Parallel RNA(9)
NRAS	r(GGGAGGGGCGGGUCUGGG)	Parallel RNA(10)
ds26	CAATCGGATCGAATTCGATCCGATTG	Duplex DNA(4)

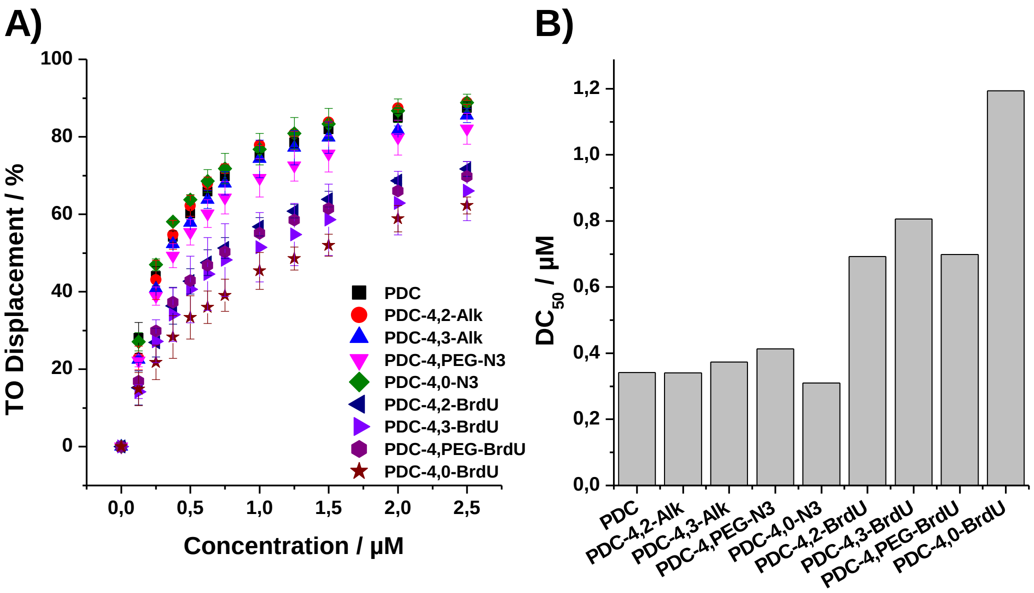

Figure S3: A) G4 Fluorescent Intercalator Displacement (FID) plot obtained for compounds PDC (used as a reference), PDC-4,2-Alk, PDC-4,3-Alk, PDC-4,PEG-N3, PDC-4,0-N3, PDC-4,2-BrdU, PDC-4,3-BrdU, PDC-4,PEG-BrdU, and PDC-4,0-BrdU (from 0 µM to 2.5 µM) in the presence of the human telomeric sequence 22AG (0.25 µM) and Thiazole Orange fluorescent probe (TO, 0.5 µM, 2 molar equiv). Affinities of ligands are expressed by TO displacement as indicated in material and methods. Error bars correspond to SD of two independent experiments. Buffer used is K^+100 (10 mM lithium cacodylate and 100 mM KCl, pH = 7.3). B) Bar chart representing the required concentration for each ligand to displace 50% of TO (DC_{50}).

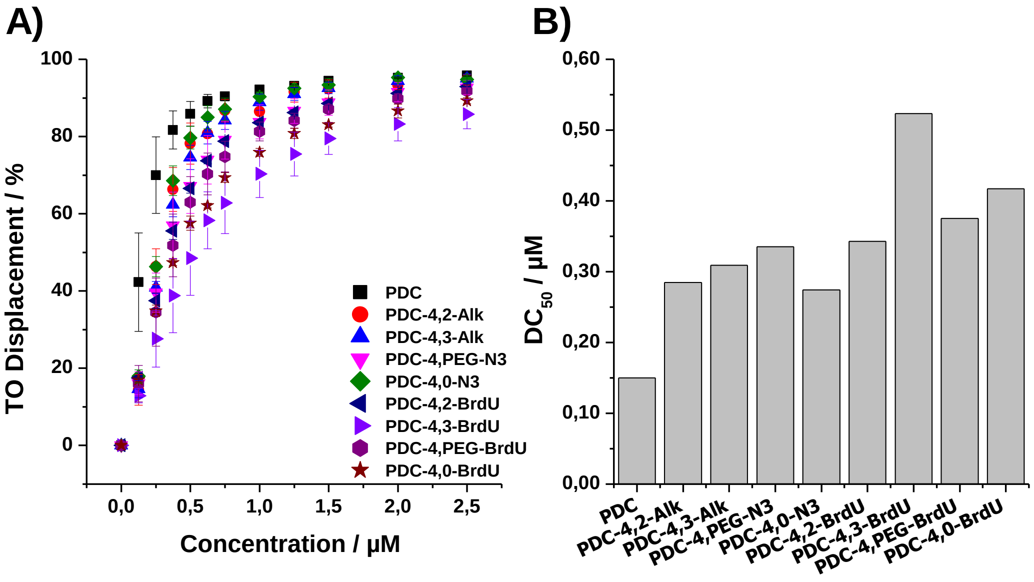

Figure S4: A) G4 Fluorescent Intercalator Displacement (FID) plot obtained for compounds PDC (used as a reference), PDC-4,2-Alk, PDC-4,3-Alk, PDC-4,PEG-N3, PDC-4,0-N3, PDC-4,2-BrdU, PDC-4,3-BrdU, PDC-4,PEG-BrdU, and PDC-4,0-BrdU (from 0 µM to 2.5 µM) in the presence of the c-Myc oncogene sequence c-Myc22 (0.25 µM) and Thiazole Orange fluorescent probe (TO, 0.5 µM, 2 molar equiv). Affinities of ligands are expressed by TO displacement as indicated in material and methods. Error bars correspond to SD of two

independent experiments. Buffer used is K^+100 (10 mM lithium cacodylate and 100 mM KCl, pH = 7.3). B) Bar chart representing the required concentration for each ligand to displace 50% of TO (DC_{50}).

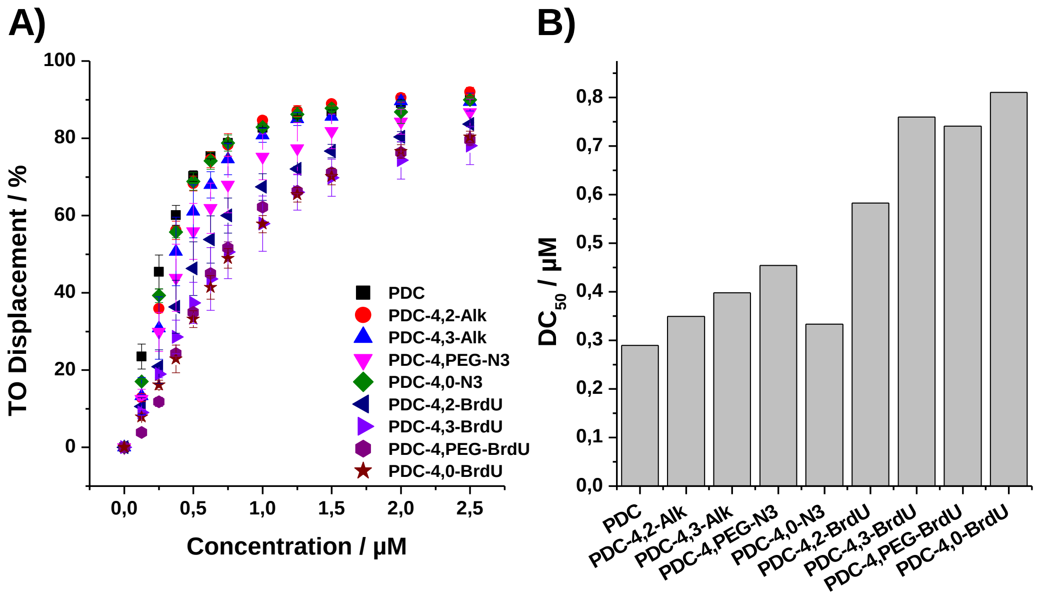

Figure S5: A) G4 Fluorescent Intercalator Displacement (FID) plot obtained for compounds PDC (used as a reference), PDC-4,2-Alk, PDC-4,3-Alk, PDC-4,PEG-N3, PDC-4,0-N3, PDC-4,2-BrdU, PDC-4,3-BrdU, PDC-4,PEG-BrdU, and PDC-4,0-BrdU (from 0 µM to 2.5 µM) in the presence of the human minisatellite repeat native sequence CEB25wt (0.25 µM) and Thiazole Orange fluorescent probe (TO, 0.5 µM, 2 molar equiv). Affinities of ligands are expressed by TO displacement as indicated in material and methods. Error bars correspond to SD of two independent experiments. Buffer used is K^+100 (10 mM lithium cacodylate and 100 mM KCl, pH = 7.3). B) Bar chart representing the required concentration for each ligand to displace 50% of TO (DC_{50}).

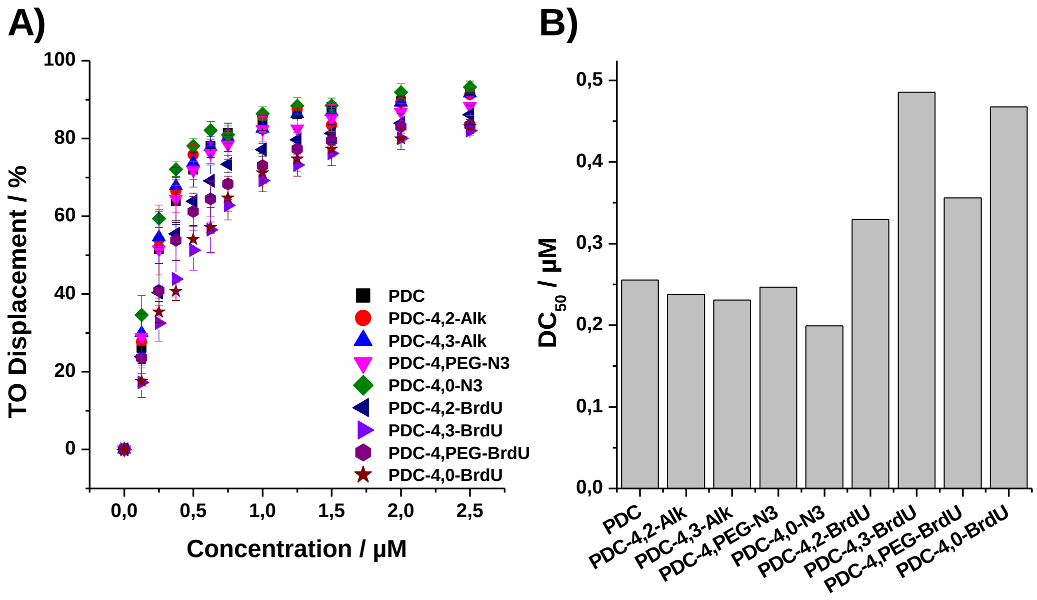

Figure S6: A) G4 Fluorescent Intercalator Displacement (FID) plot obtained for compounds PDC (used as a reference), PDC-4,2-Alk, PDC-4,3-Alk, PDC-4,PEG-N3, PDC-4,0-N3, PDC-4,2-BrdU, PDC-4,3-BrdU, PDC-

4,PEG-BrdU, and PDC-4,0-BrdU (from 0 µM to 2.5 µM) in the presence of the human minisatellite repeat modified sequence CEB25L111T (0.25 µM) and Thiazole Orange fluorescent probe (TO, 0.5 µM, 2 molar equiv). Affinities of ligands are expressed by TO displacement as indicated in material and methods. Error bars correspond to SD of two independent experiments. Buffer used is K^+100 (10 mM lithium cacodylate and 100 mM KCl, pH = 7.3). B) Bar chart representing the required concentration for each ligand to displace 50% of TO (DC_{50}).

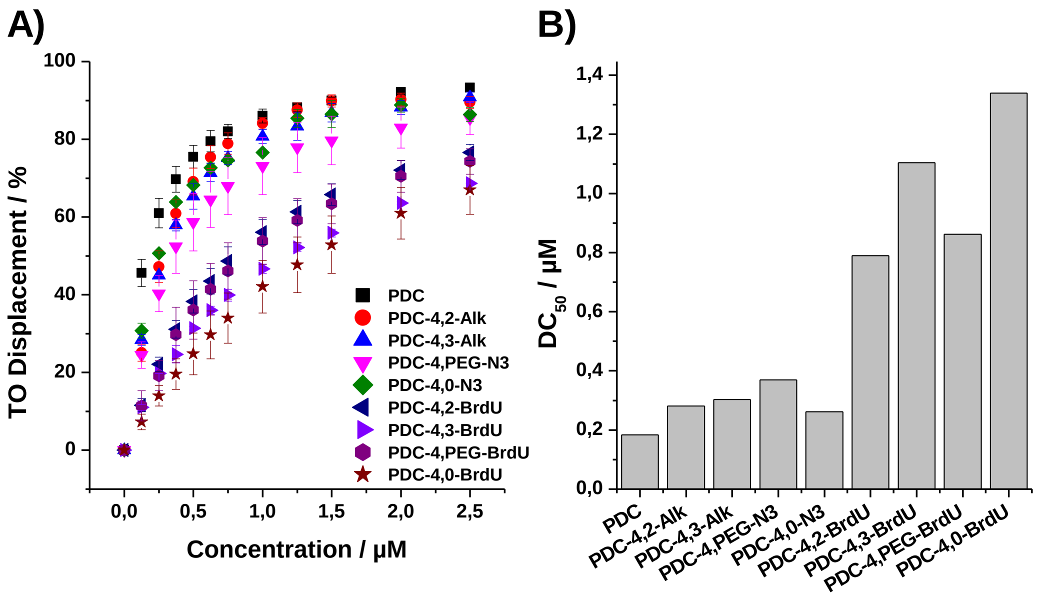

Figure S7: A) G4 Fluorescent Intercalator Displacement (FID) plot obtained for compounds PDC (used as a reference), PDC-4,2-Alk, PDC-4,3-Alk, PDC-4,PEG-N3, PDC-4,0-N3, PDC-4,2-BrdU, PDC-4,3-BrdU, PDC-4,PEG-BrdU, and PDC-4,0-BrdU (from 0 µM to 2.5 µM) in the presence of the modified human telomeric sequence 21CTA (0.25 µM) and Thiazole Orange fluorescent probe (TO, 0.5 µM, 2 molar equiv). Affinities of ligands are expressed by TO displacement as indicated in material and methods. Error bars correspond to SD of two independent experiments. Buffer used is K^+100 (10 mM lithium cacodylate and 100 mM KCl, pH = 7.3). B) Bar chart representing the required concentration for each ligand to displace 50% of TO (DC_{50}).

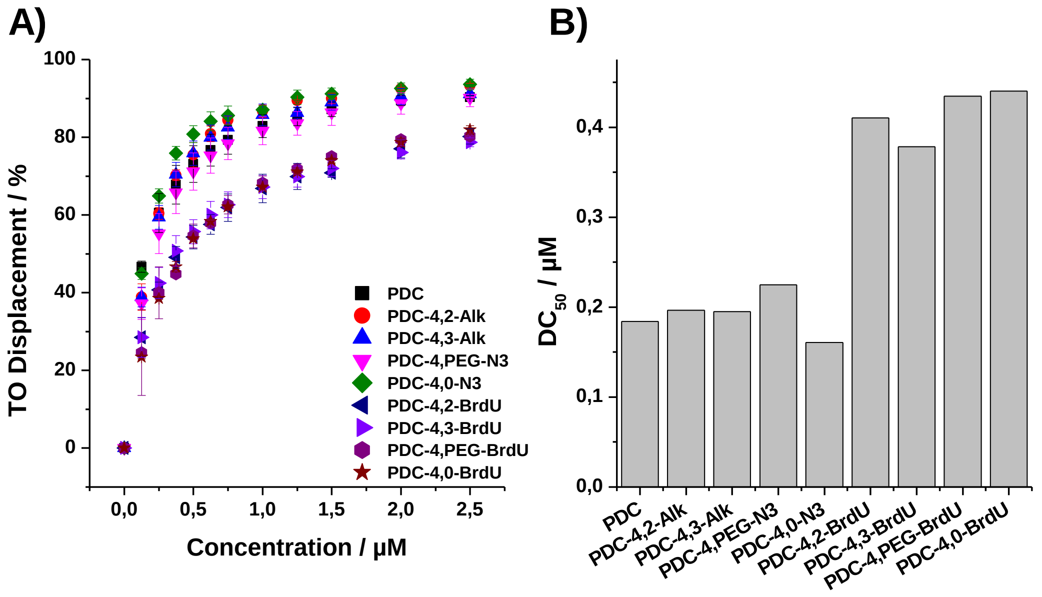

Figure S8: A) G4 Fluorescent Intercalator Displacement (FID) plot obtained for compounds PDC (used as a reference), PDC-4,2-Alk, PDC-4,3-Alk, PDC-4,PEG-N3, PDC-4,0-N3, PDC-4,2-BrdU, PDC-4,3-BrdU, PDC-4,PEG-BrdU, and PDC-4,0-BrdU (from 0 µM to 2.5 µM) in the presence of the c-kit2 oncogene promoter sequence cKit2 (0.25 µM) and Thiazole Orange fluorescent probe (TO, 0.5 µM, 2 molar equiv). Affinities of ligands are expressed by TO displacement as indicated in material and methods. Error bars correspond to SD of two independent experiments. Buffer used is K^+100 (10 mM lithium cacodylate and 100 mM KCl, pH = 7.3). B) Bar chart representing the required concentration for each ligand to displace 50% of TO (DC_{50}).

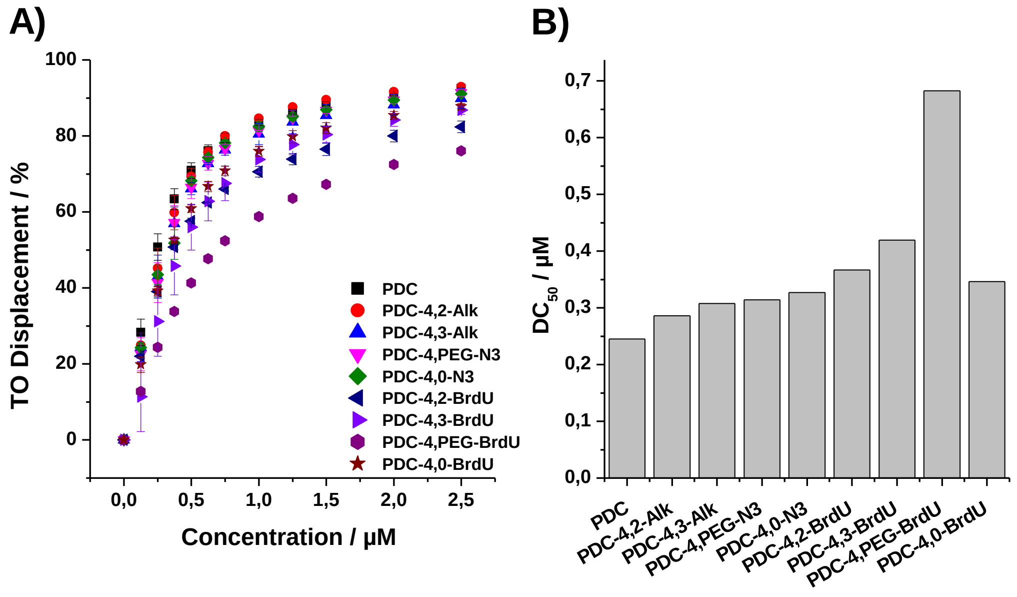

Figure S9: A) G4 Fluorescent Intercalator Displacement (FID) plot obtained for compounds PDC (used as a reference), PDC-4,2-Alk, PDC-4,3-Alk, PDC-4,PEG-N3, PDC-4,0-N3, PDC-4,2-BrdU, PDC-4,3-BrdU, PDC-4,PEG-BrdU, and PDC-4,0-BrdU (from 0 µM to 2.5 µM) in the presence of the RNA human telomeric sequence TERRA (0.25 µM) and Thiazole Orange fluorescent probe (TO, 0.5 µM, 2 molar equiv). Affinities of ligands are expressed by TO displacement as indicated in material and methods. Error bars correspond to SD of two

independent experiments. Buffer used is K^+100 (10 mM lithium cacodylate and 100 mM KCl, pH = 7.3). B) Bar chart representing the required concentration for each ligand to displace 50% of TO (DC_{50}).

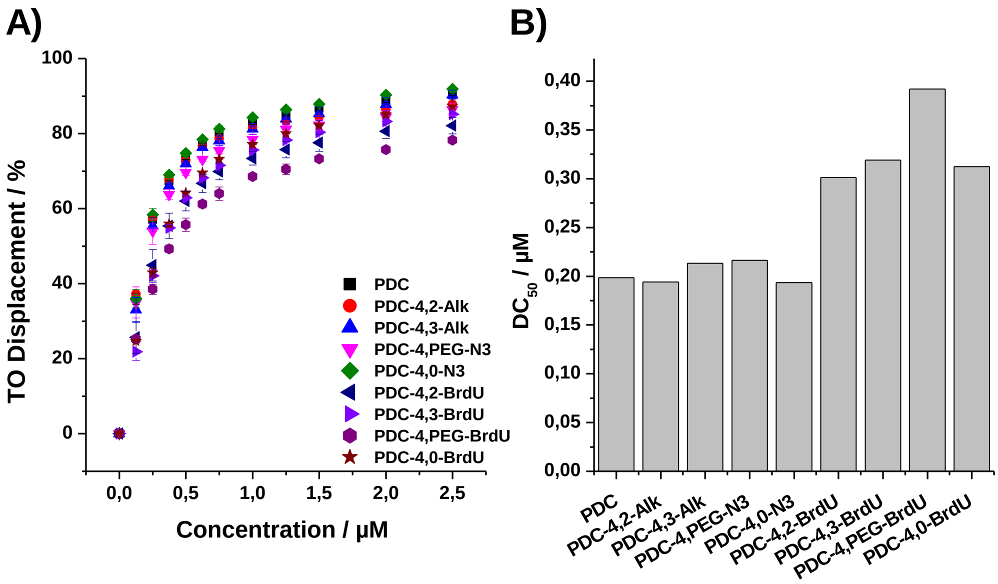

Figure S10: A) G4 Fluorescent Intercalator Displacement (FID) plot obtained for compounds PDC (used as a reference), PDC-4,2-Alk, PDC-4,3-Alk, PDC-4,PEG-N3, PDC-4,0-N3, PDC-4,2-BrdU, PDC-4,3-BrdU, PDC-4,PEG-BrdU, and PDC-4,0-BrdU (from 0 µM to 2.5 µM) in the presence of the RNA G4-forming sequence of the human NRAS proto-oncogene transcript NRAS (0.25 µM) and Thiazole Orange fluorescent probe (TO, 0.5 µM, 2 molar equiv). Affinities of ligands are expressed by TO displacement as indicated in material and methods. Error bars correspond to SD of two independent experiments. Buffer used is K^+100 (10 mM lithium cacodylate and 100 mM KCl, pH = 7.3). B) Bar chart representing the required concentration for each ligand to displace 50% of TO (DC_{50}).

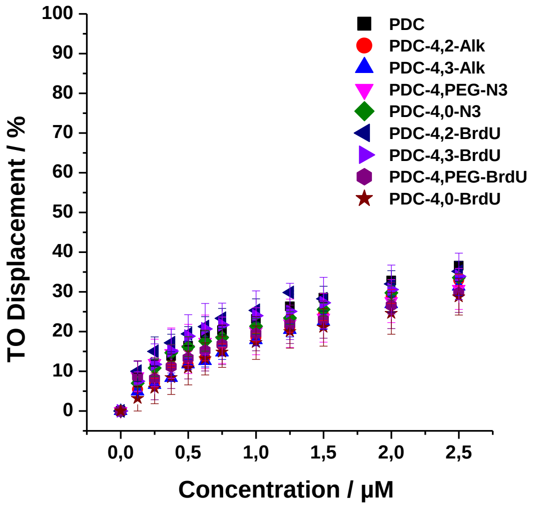

Figure S11: G4 Fluorescent Intercalator Displacement (FID) plot obtained for compounds PDC (used as a reference), PDC-4,2-Alk, PDC-4,3-Alk, PDC-4,PEG-N3, PDC-4,0-N3, PDC-4,2-BrdU, PDC-4,3-BrdU, PDC-4,PEG-BrdU, and PDC-4,0-BrdU (from 0 µM to 2.5 µM) in the presence of a duplex DNA sequence ds26 (0.25

µM) and Thiazole Orange fluorescent probe (TO, 0.75 µM, 3 molar equiv). Affinities of ligands are expressed by TO displacement as indicated in material and methods. Error bars correspond to SD of two independent experiments. Buffer used is K^+100 (10 mM lithium cacodylate and 100 mM KCl, pH = 7.3).

Table S3. DC_{50} values of PDC derivatives obtained by G4-FID assay

	DC_{50} (µM)							
	22AG	21CTA	c-Myc22	c-kit2	CEB25wt	CEB25L111T	TERRA	NRAS
PDC	0.34	0.18	0.15	0.18	0.29	0.26	0,25	0,20
PDC-4,2-Alk	0.34	0.28	0.28	0.20	0.35	0.24	0,29	0,19
PDC-4,3-Alk	0.37	0.30	0.31	0.20	0.40	0.23	0,31	0,21
PDC-4,PEG-N3	0.41	0.37	0.34	0.22	0.45	0.25	0,31	0,22
PDC-4,0-N3	0.31	0.26	0.27	0.16	0.33	0.20	0,33	0,19
PDC-4,2-BrdU	0.69	0.79	0.34	0.41	0.58	0.33	0,37	0,30
PDC-4,3-BrdU	0.80	1.10	0.52	0.38	0.76	0.48	0,42	0,32
PDC-4,PEG-BrdU	0.70	0.86	0.38	0.43	0.74	0.36	0,68	0,39
PDC-4,0-BrdU	1.19	1.34	0.42	0.44	0.81	0.47	0,35	0,31

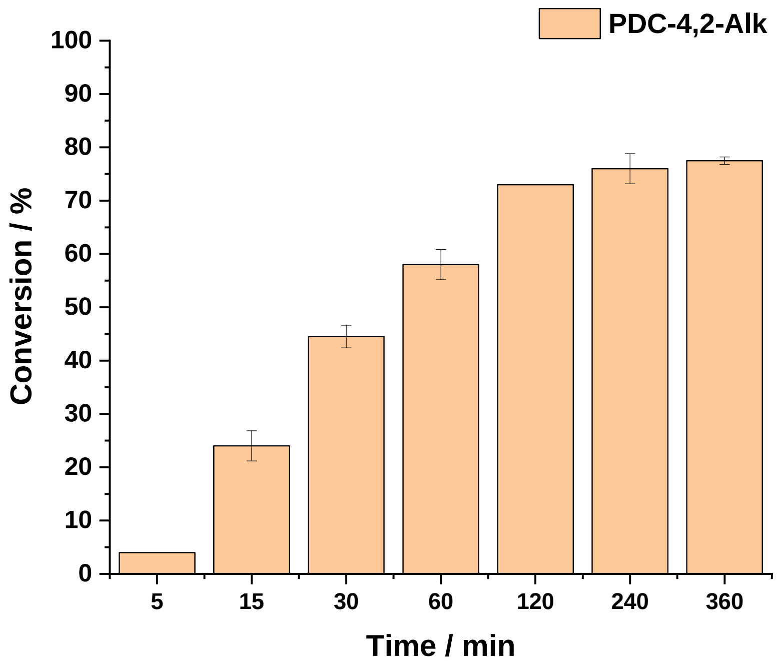

Figure S12. PDC-4,2-Alk Copper(I)-catalyzed Alkyne/Azide Cycloaddition reaction kinetics analysis. Reaction conversion was followed by HPLC after 5 min, 15 min, 30, 60, 120, 240, and 360 min of reaction. The graph shows the % of conversion of PDC-4,2-Alk into PDC-4,2-BrdU in function of the reaction time.

A)

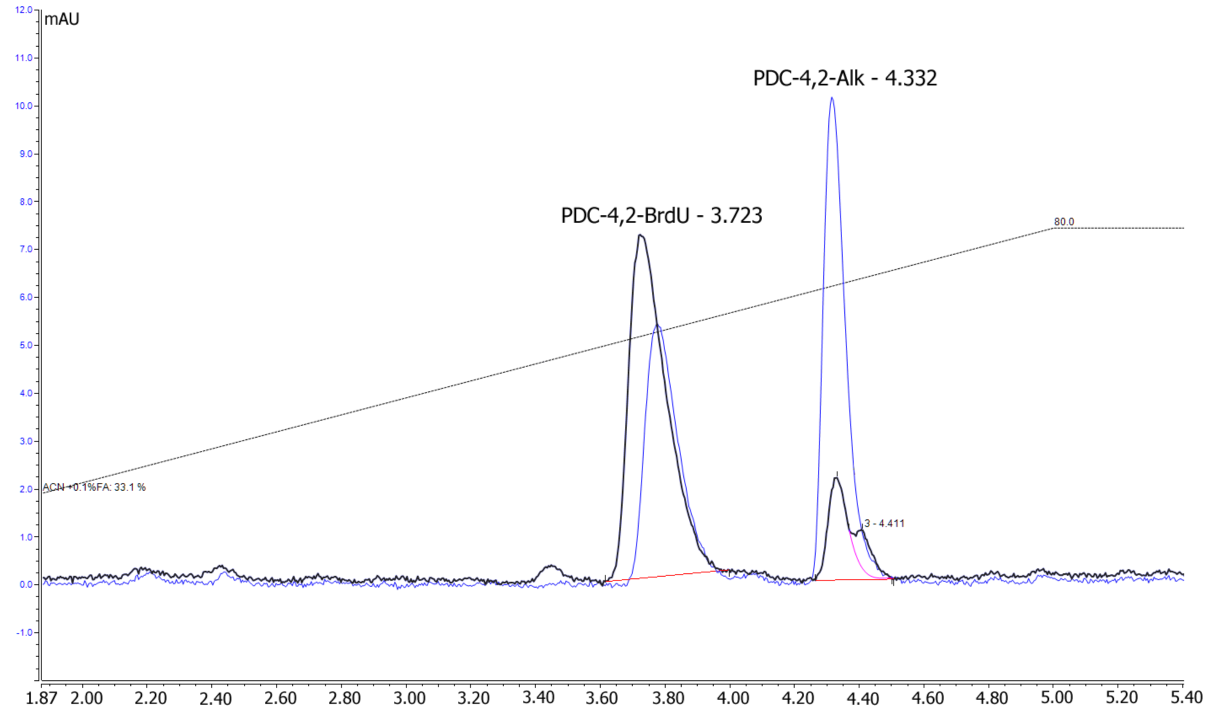
mAU
PDC-4,2-Alk - 4.332
PDC-4,2-BrdU - 3.723
80.0
ACN +0.1%FA: 33.1 %
3 - 4.411
1.87 2.00 2.20 2.40 2.60 2.80 3.00 3.20 3.40 3.60 3.80 4.00 4.20 4.40 4.60 4.80 5.00 5.20 5.40

B)

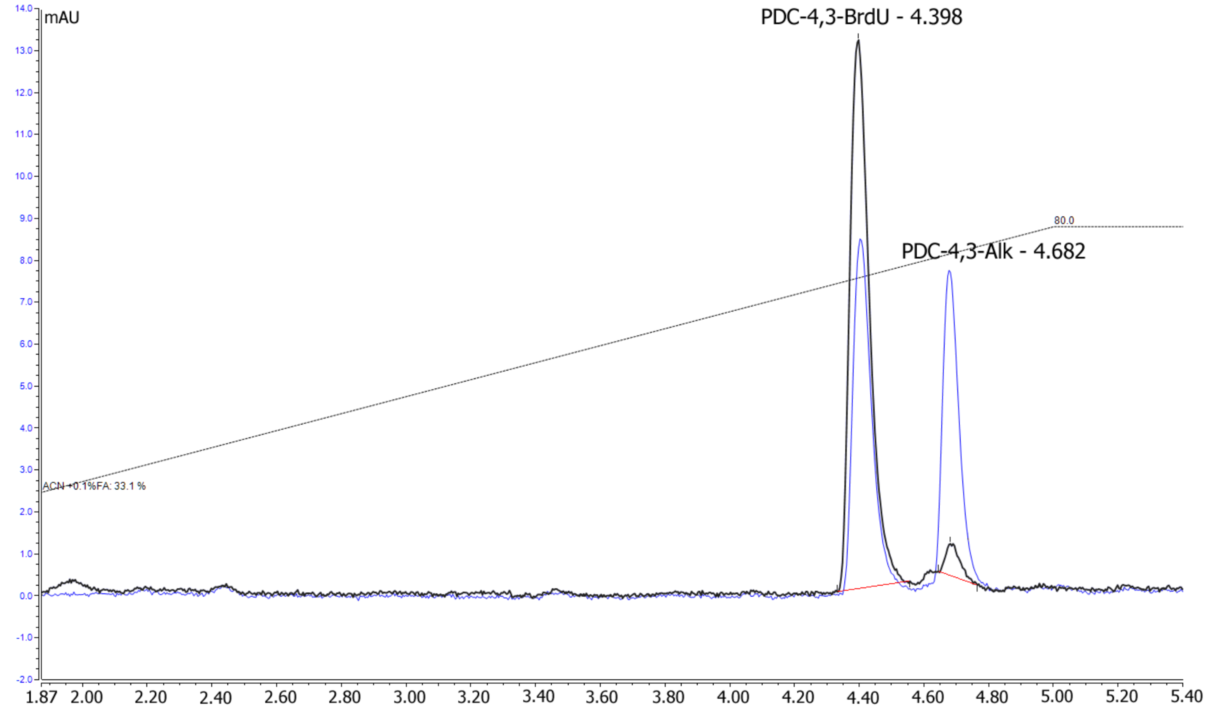
mAU
PDC-4,3-BrdU - 4.398
80.0
PDC-4,3-Alk - 4.682
ACN +0.1%FA: 33.1 %
1.87 2.00 2.20 2.40 2.60 2.80 3.00 3.20 3.40 3.60 3.80 4.00 4.20 4.40 4.60 4.80 5.00 5.20 5.40

C)

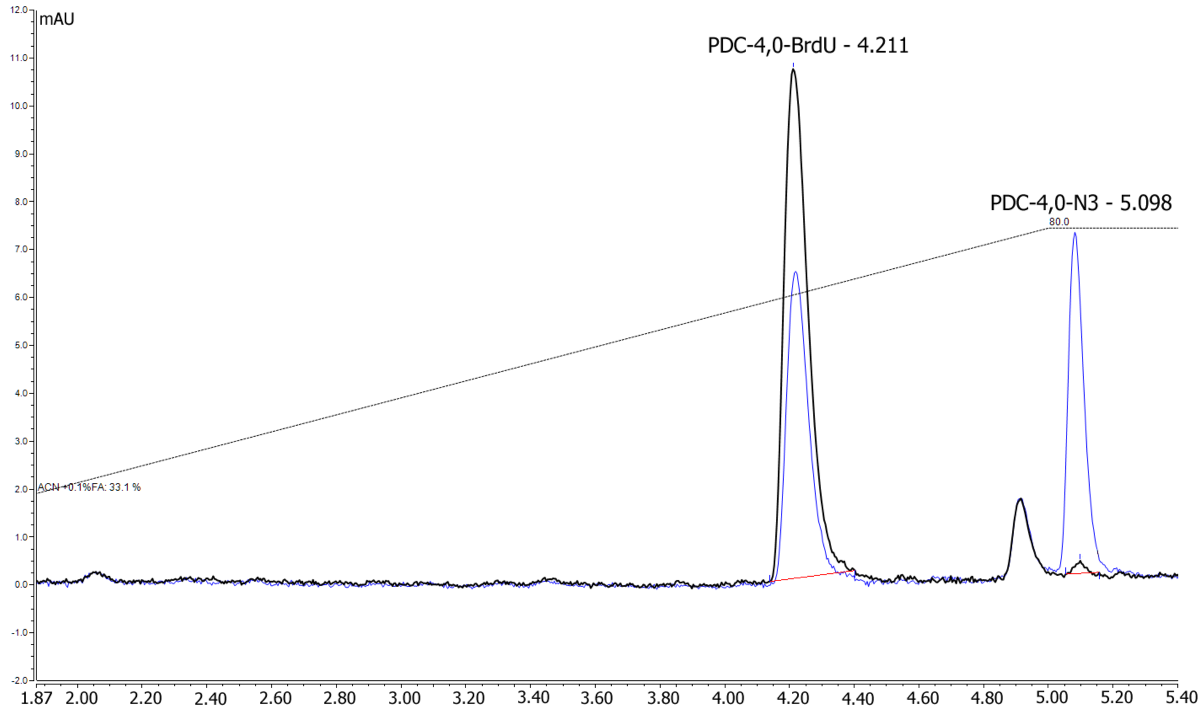

D)

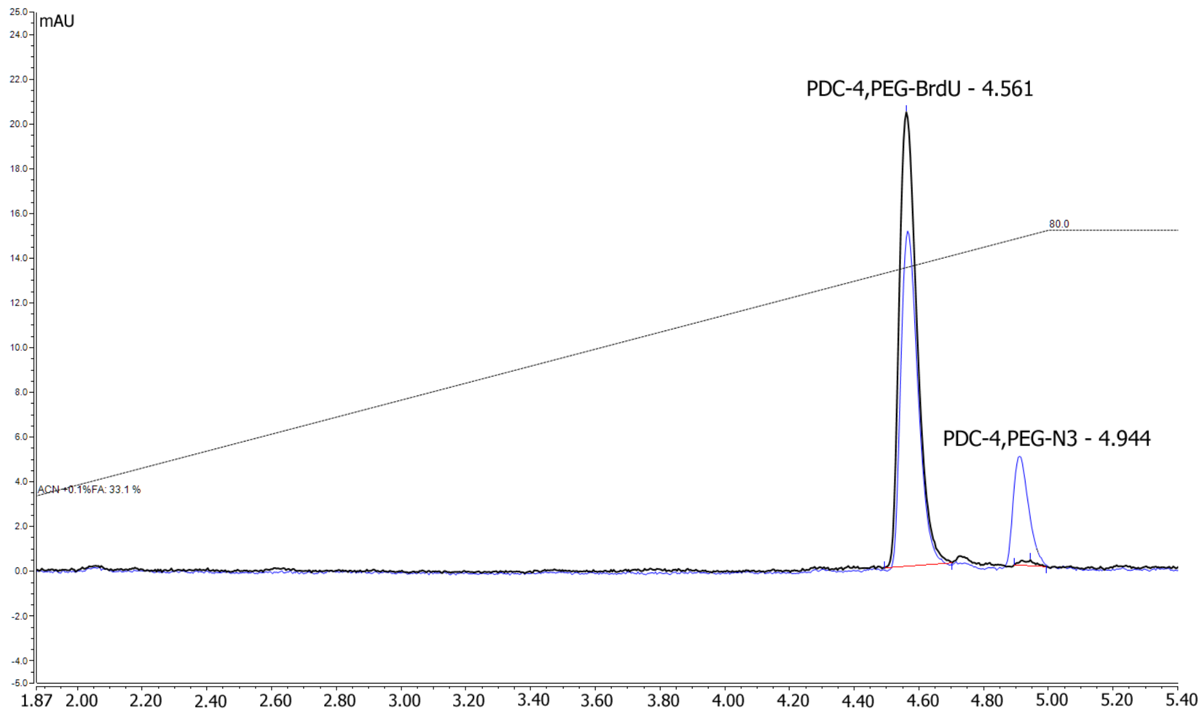

Figure S13: Chromatographic traces for the Cu-catalyzed 1,3-dipolar cycloaddition obtained in the presence (black trace) or in the absence (blue trace) of c-Myc22 (G14T-G23T). Reaction conditions: c-Myc22 (G14T-G23T) (1 equiv), PDC CuAAC precursor (1 equiv), 5-BrdU appropriate partner (2 equiv), $CuSO_4.5H_2O$ (20 equiv), THPTA (100 equiv), and sodium ascorbate (80 equiv). A) PDC-4,2-Alk and 5-BrdU-N3, B) PDC-4,3-Alk and 5-BrdU-N3, C) PDC-4,0-N3 and 5-BrdU-Alk, and D) PDC-4,PEG-N3 and 5-BrdU-Alk.

A)

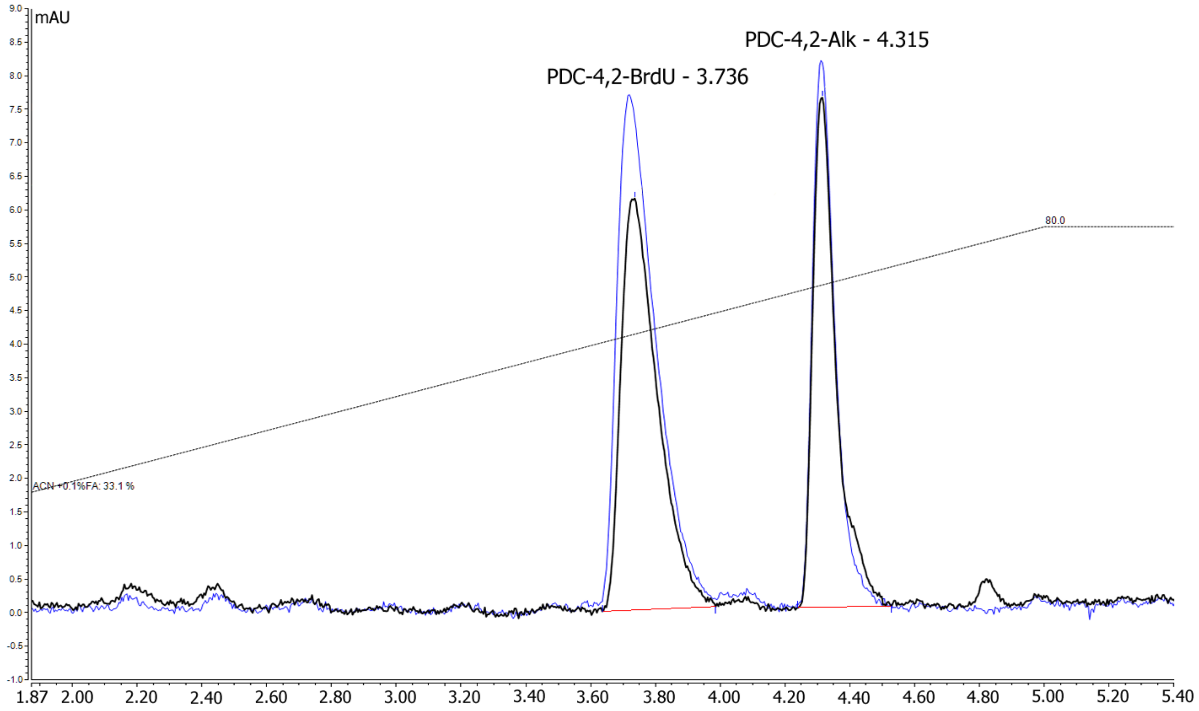
mAU
PDC-4,2-Alk - 4.315
PDC-4,2-BrdU - 3.736
80.0
ACN +0.1%FA: 33.1 %
9.0
8.5
8.0
7.5
7.0
6.5
6.0
5.5
5.0
4.5
4.0
3.5
3.0
2.5
2.0
1.5
1.0
0.5
0.0
-0.5
-1.0
1.87 2.00 2.20 2.40 2.60 2.80 3.00 3.20 3.40 3.60 3.80 4.00 4.20 4.40 4.60 4.80 5.00 5.20 5.40

B)

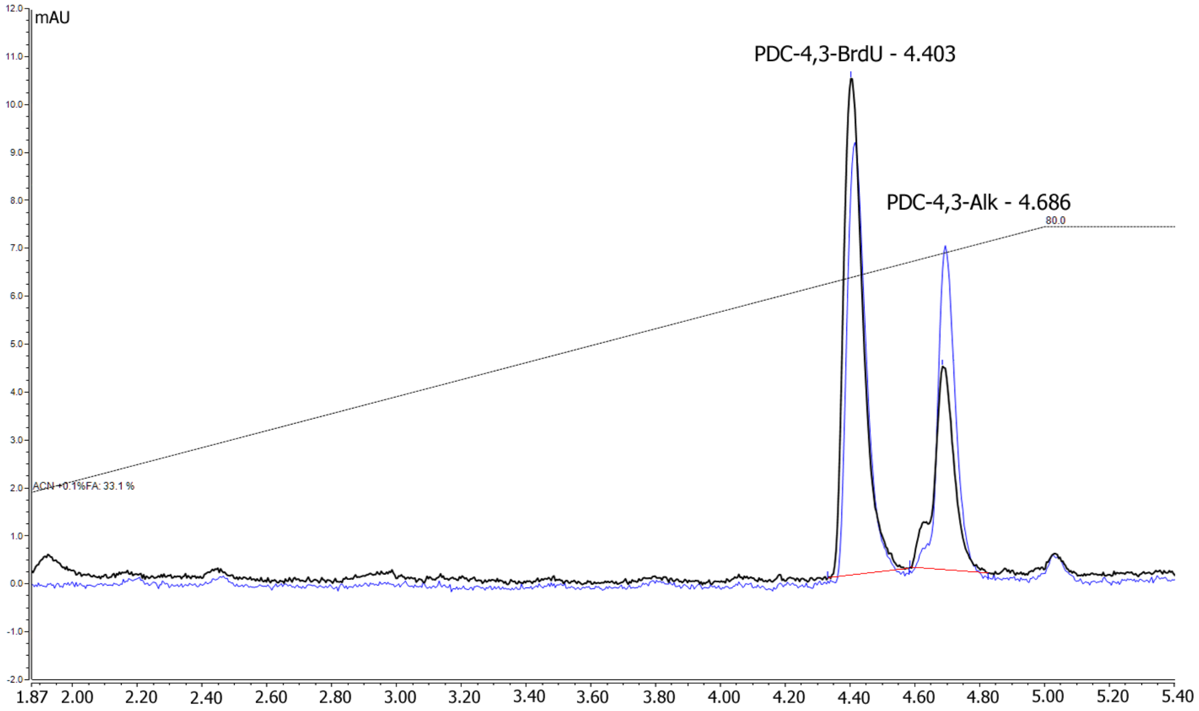
mAU
PDC-4,3-BrdU - 4.403
PDC-4,3-Alk - 4.686
80.0
ACN +0.1%FA: 33.1 %
12.0
11.0
10.0
9.0
8.0
7.0
6.0
5.0
4.0
3.0
2.0
1.0
0.0
-1.0
-2.0
1.87 2.00 2.20 2.40 2.60 2.80 3.00 3.20 3.40 3.60 3.80 4.00 4.20 4.40 4.60 4.80 5.00 5.20 5.40

C)

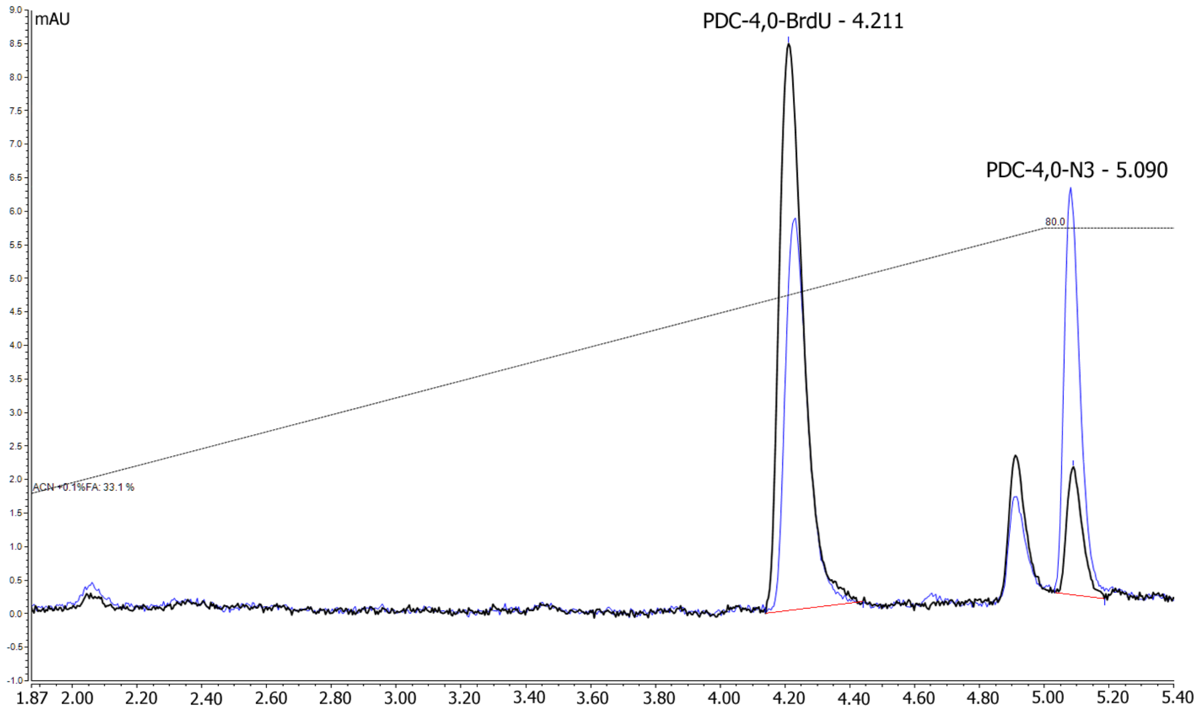

D)

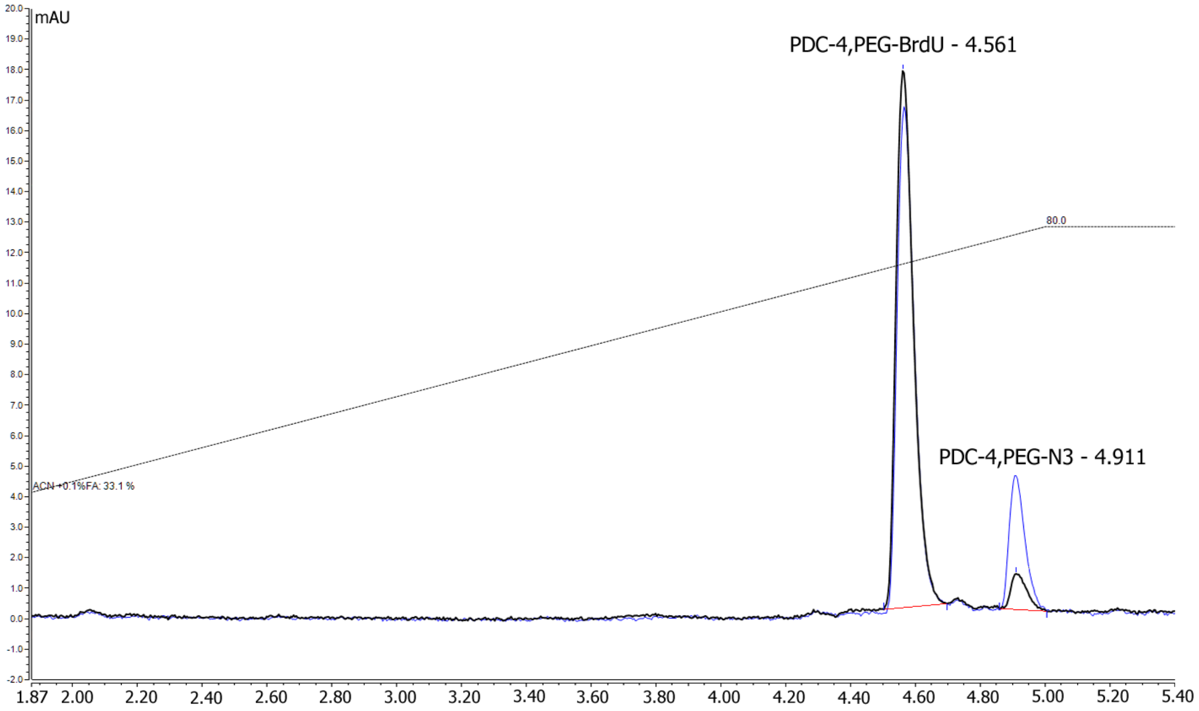

Figure S14: Chromatographic traces for the Cu-catalyzed 1,3-dipolar cycloaddition obtained in the presence (black trace) or in the absence (blue trace) of 22AG. Reaction conditions: 22AG (1 equiv), PDC CuAAC precursor (1 equiv), 5-BrdU appropriate partner (2 equiv), $CuSO_4.5H_2O$ (20 equiv), THPTA (100 equiv), and sodium ascorbate (80 equiv). A) PDC-4,2-Alk and 5-BrdU-N3, B) PDC-4,3-Alk and 5-BrdU-N3, C) PDC-4,0-N3 and 5-BrdU-Alk, and D) PDC-4,PEG-N3 and 5-BrdU-Alk.

A)

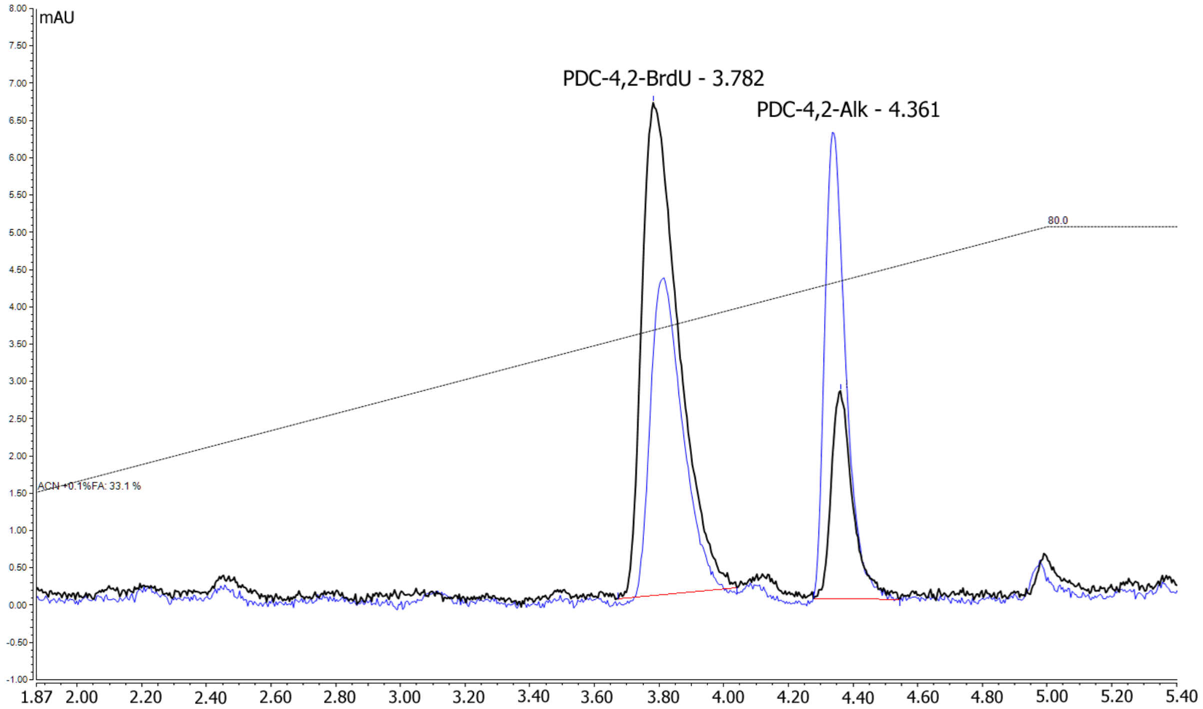

mAU
PDC-4,2-BrdU - 3.782
PDC-4,2-Alk - 4.361
80.0
ACN +0.1%FA: 33.1 %
1.87 2.00 2.20 2.40 2.60 2.80 3.00 3.20 3.40 3.60 3.80 4.00 4.20 4.40 4.60 4.80 5.00 5.20 5.40

B)

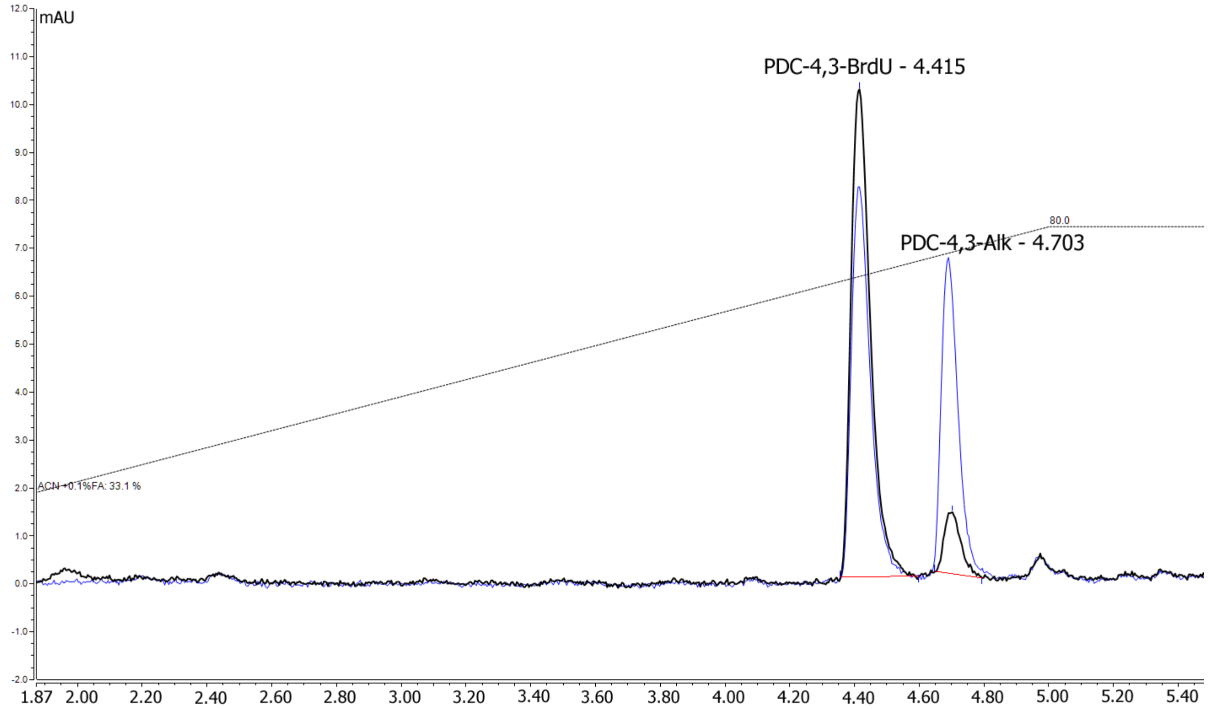

mAU
PDC-4,3-BrdU - 4.415
PDC-4,3-Alk - 4.703
80.0
ACN +0.1%FA: 33.1 %
1.87 2.00 2.20 2.40 2.60 2.80 3.00 3.20 3.40 3.60 3.80 4.00 4.20 4.40 4.60 4.80 5.00 5.20 5.40

C)

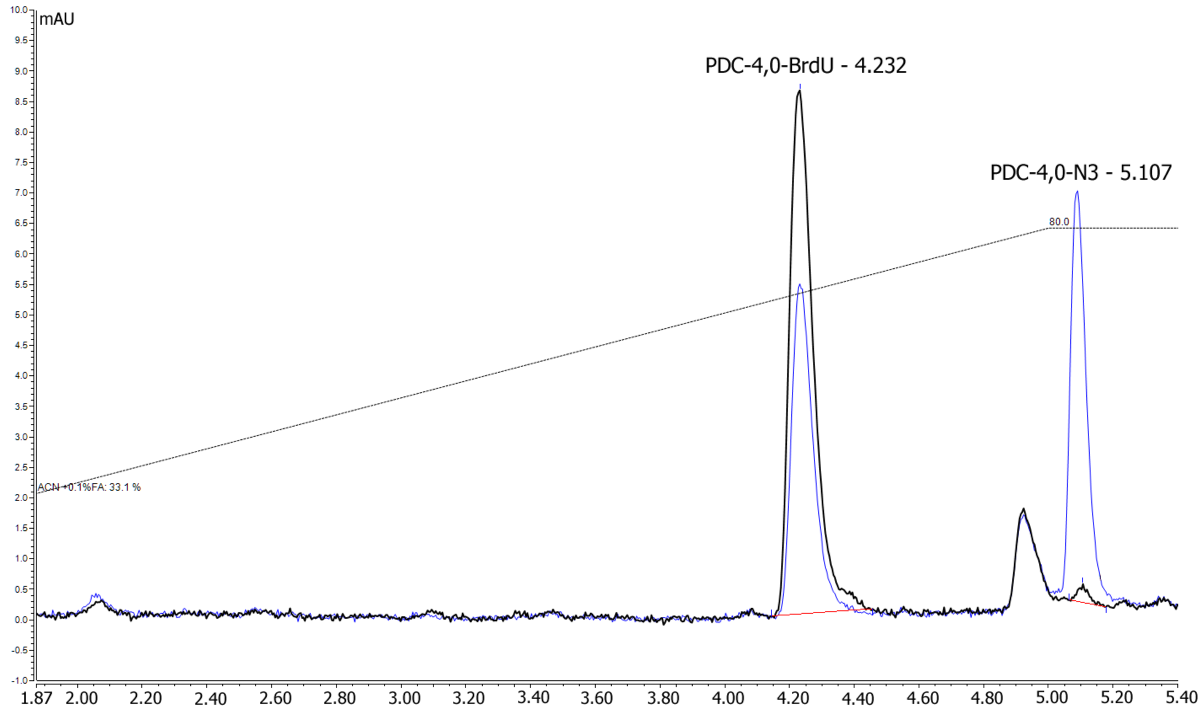

D)

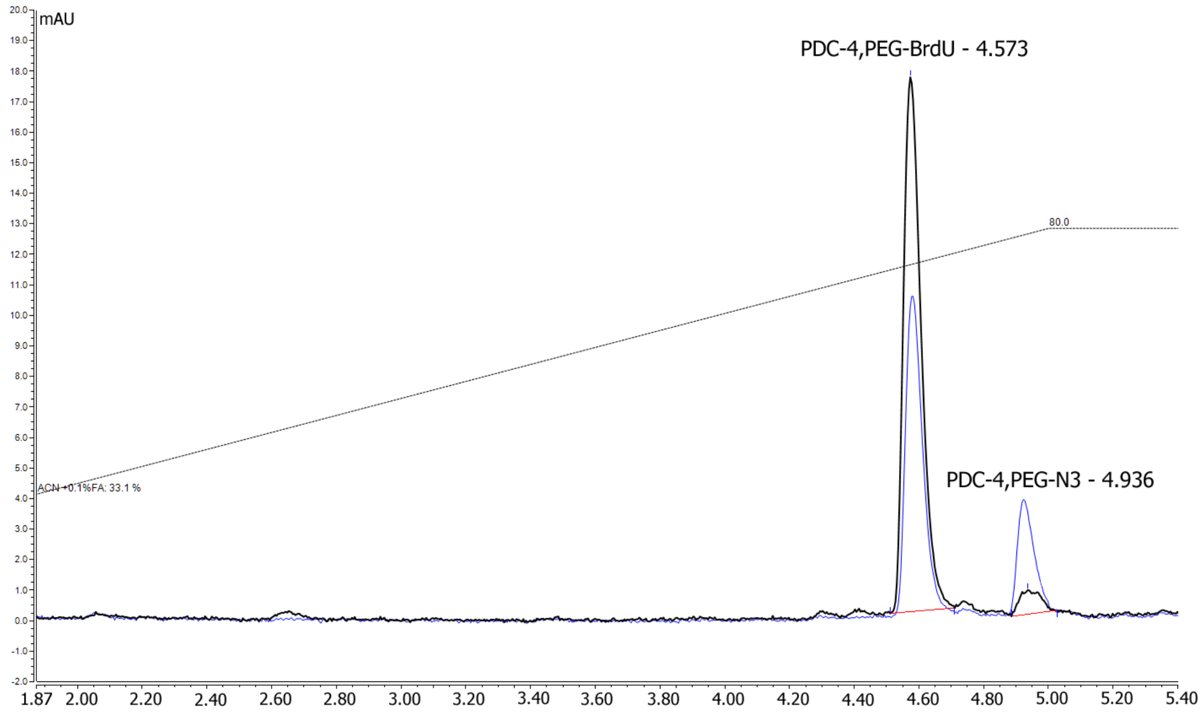

Figure S15: Chromatographic traces for the Cu-catalyzed 1,3-dipolar cycloaddition obtained in the presence (black trace) or in the absence (blue trace) of TERRA. Reaction conditions: TERRA (1 equiv), PDC CuAAC precursor (1 equiv), 5-BrdU appropriate partner (2 equiv), $CuSO_4.5H_2O$ (20 equiv), THPTA (100 equiv), and sodium ascorbate (80 equiv). A) PDC-4,2-Alk and 5-BrdU-N3, B) PDC-4,3-Alk and 5-BrdU-N3, C) PDC-4,0-N3 and 5-BrdU-Alk, and D) PDC-4,PEG-N3 and 5-BrdU-Alk.

Table S4. Cytotoxicity of the different synthesized ligands in A549 cell lines.

	**A549 IC50 (μM)**
**PDC-4,2-Alk**	27.8 ± 1.56
**PDC-4,3-Alk**	45.8 ± 0.56
**PDC-4,0-N3**	16.1 ± 0.21
**PDC-4,PEG-N3**	> 100
**PDC-4,2-BrdU**	53.0 ± 7.97
**PDC-4,3-BrdU**	63.5 ± 19.31
**PDC-4,0-BrdU**	21.2 ± 1.10
**PDC-4,PEG-BrdU**	> 100

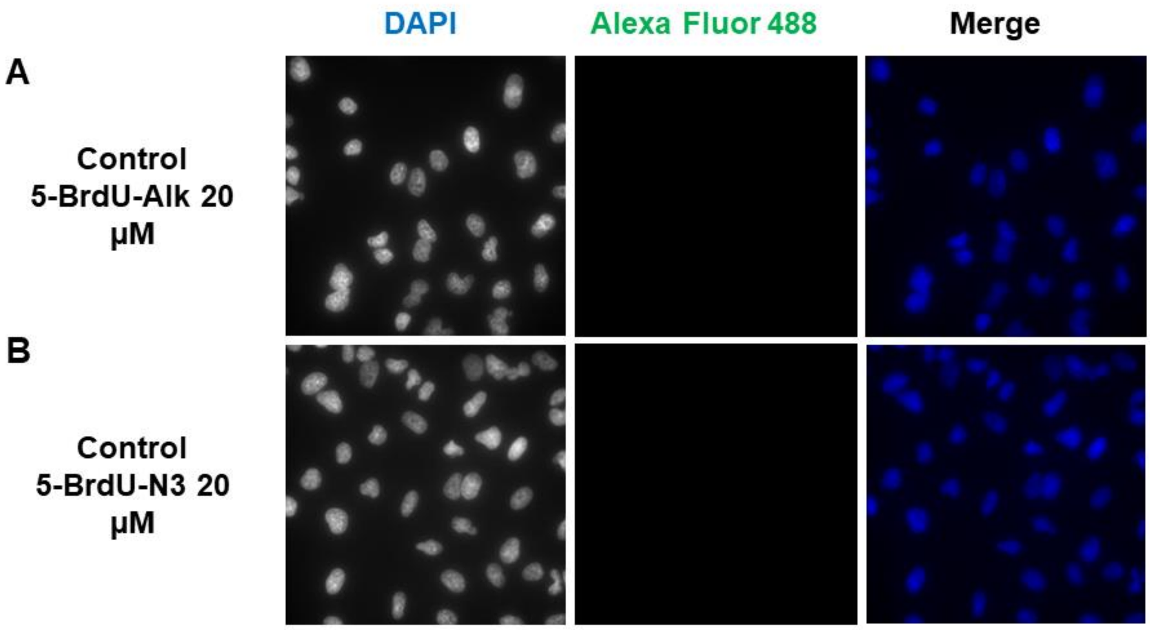

Figure S16. Control samples of immunofluorescent staining experiments in A549 cells and nucleus visualized with DAPI. Fluorescence wide-field microscopy images of A549 cells: after 16 h incubation with (**A**) 20 µM 5-BrdU-Alk, and (**B**) 20 µM 5-BrdU-N3 after incubation with mouse anti 5-BrdU antibody and Alexa Fluor 488 conjugated goat anti mouse secondary antibody. For each image, DAPI and Alexa Fluor 488 (FITC) channels are represented separately. Merged images are shown on the right. Images are represented as a Z-projection. Scale bar: 20 µm.

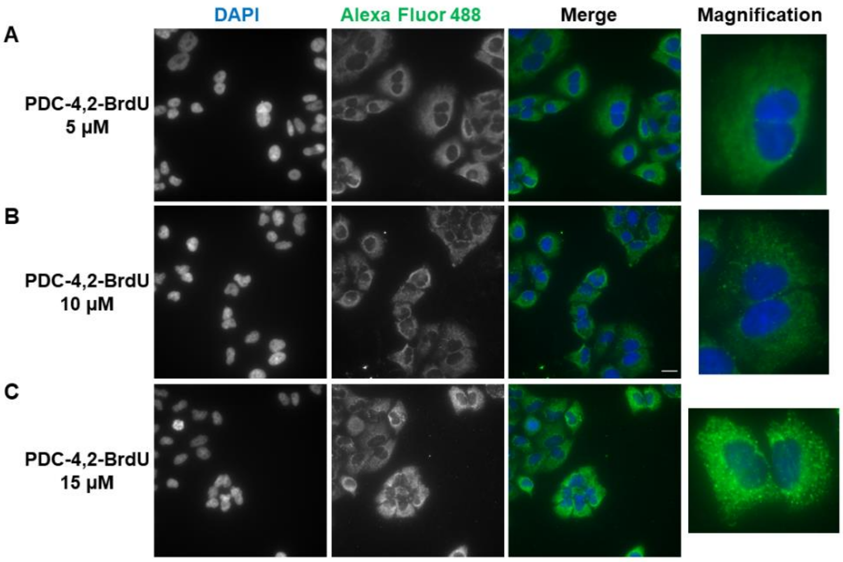

Figure S17. G4 ligand immunofluorescent staining in A549 cells and nucleus visualized with DAPI. Fluorescence wide-field microscopy images of A549 cells after 16 h incubation with (**A**) 5 µM PDC-4,2-BrdU, (**B**) 10 µM PDC-4,2-BrdU, and (**C**) 15 µM PDC-4,2-BrdU after incubation with mouse anti 5-BrdU antibody and Alexa Fluor 488 conjugated goat anti mouse secondary antibody. For each image, DAPI and Alexa Fluor 488 (FITC) channels are represented separately. Merged images are shown on the right. Images are represented as a Z-projection. Scale bar: 20 µm.

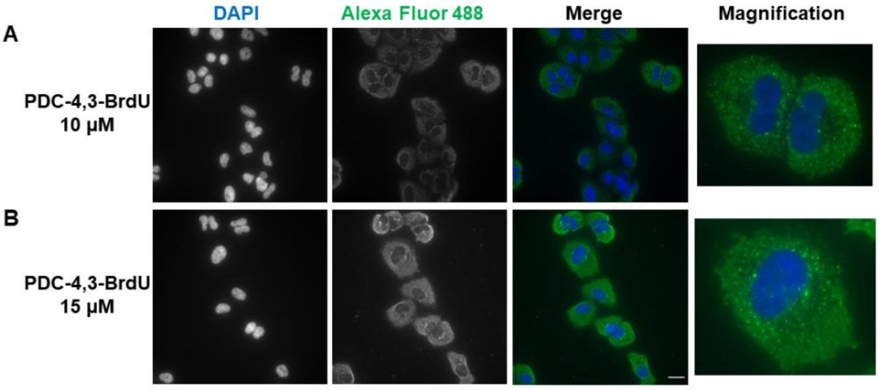

Figure S18. G4 ligand immunofluorescent staining in A549 cells and nucleus visualized with DAPI. Fluorescence wide-field microscopy images of A549 cells after 16 h incubation with (**A**) 10 µM PDC-4,3-BrdU, and (**B**) 15 µM PDC-4,3-BrdU after incubation with mouse anti 5-BrdU antibody and Alexa Fluor 488 conjugated goat anti mouse secondary antibody. For each image, DAPI and Alexa Fluor 488 (FITC) channels are represented separately. Merged images are shown on the right. Images are represented as a Z-projection. Scale bar: 20 µm.

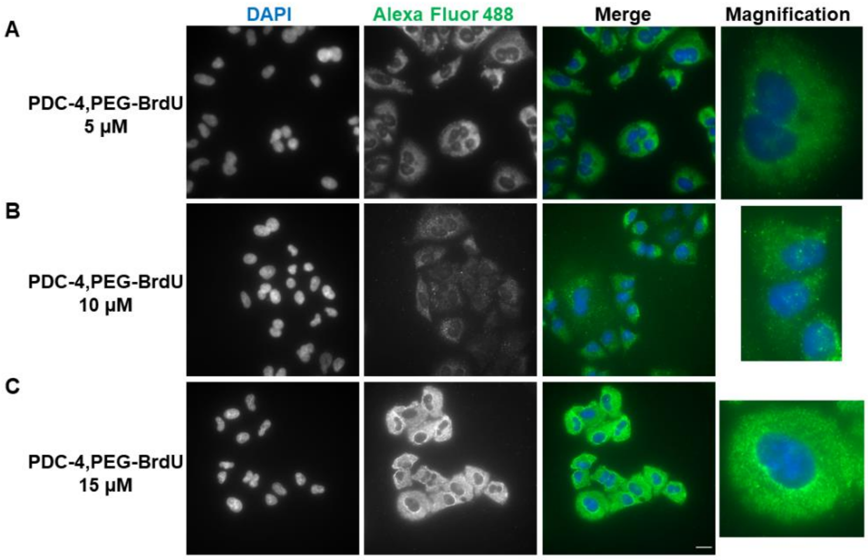

Figure S19. G4 ligand immunofluorescent staining in A549 cells and nucleus visualized with DAPI. Fluorescence wide-field microscopy images of A549 cells after 16 h incubation with (**A**) 5 µM PDC-4,PEG-BrdU, (**B**) 10 µM PDC-4,PEG-BrdU, and (**C**) 15 µM PDC-4,PEG-BrdU after incubation with mouse anti 5-BrdU antibody and Alexa Fluor 488 conjugated goat anti mouse secondary antibody. For each image, DAPI and Alexa Fluor 488 (FITC) channels are represented separately. Merged images are shown on the right. Images are represented as a Z-projection. Scale bar: 20 µm.

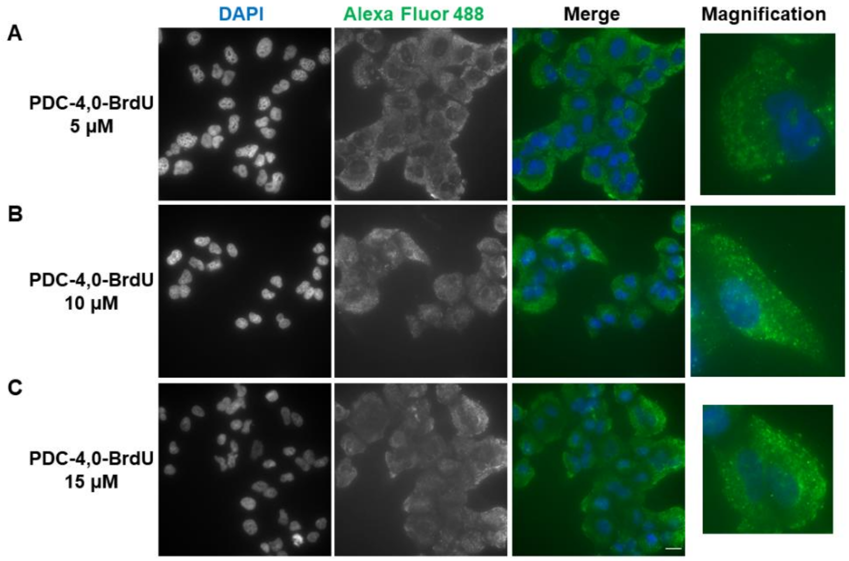

Figure S20. G4 ligand immunofluorescent staining in A549 cells and nucleus visualized with DAPI. Fluorescence wide-field microscopy images of A549 cells after 16 h incubation with (**A**) 5 µM PDC-4,0-BrdU, (**B**) 10 µM PDC-4,0-BrdU, and (**C**) 15 µM PDC-4,0-BrdU after incubation with mouse anti 5-BrdU antibody and Alexa Fluor 488 conjugated goat anti mouse secondary antibody. For each image, DAPI and Alexa Fluor 488 (FITC) channels are represented separately. Merged images are shown on the right. Images are represented as a Z-projection. Scale bar: 20 µm.

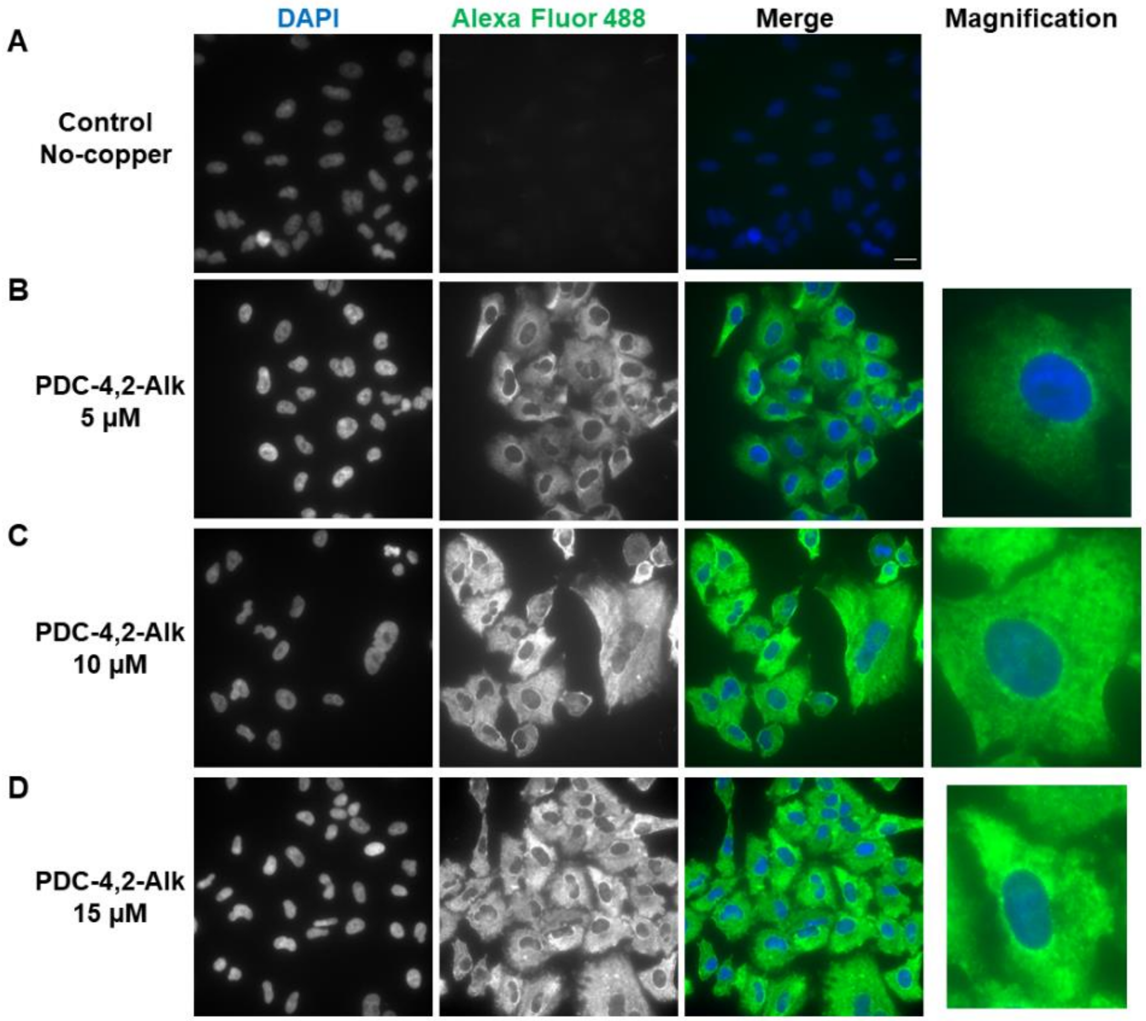

Figure S21. G4 ligand immunofluorescent staining in A549 cells and nucleus visualized with DAPI. Fluorescence wide-field microscopy images of A549 cells after 16 h incubation with (**A**) 15 µM PDC-4,2-Alk control, obtained by treatment with 5-BrdU-N3 in the absence of copper, and (**B**) 5 µM PDC-4,2-Alk, (**C**) 10 µM PDC-4,2-Alk, and (**D**) 15 µM PDC-4,2-Alk copper-based click reaction in the presence of 50 µM 5-BrdU-N3, 100 µM $CuSO_4{\cdot}5H_2O$, 500 µM THPTA, and 5 mM NaAsc. Images were obtained after incubation with mouse anti 5-BrdU antibody and Alexa Fluor 488 conjugated goat anti mouse secondary antibody. For each image, DAPI and Alexa Fluor 488 (FITC) channels are represented separately. Merged images are shown on the right. Images are represented as a Z-projection. Scale bar: 20 µm.

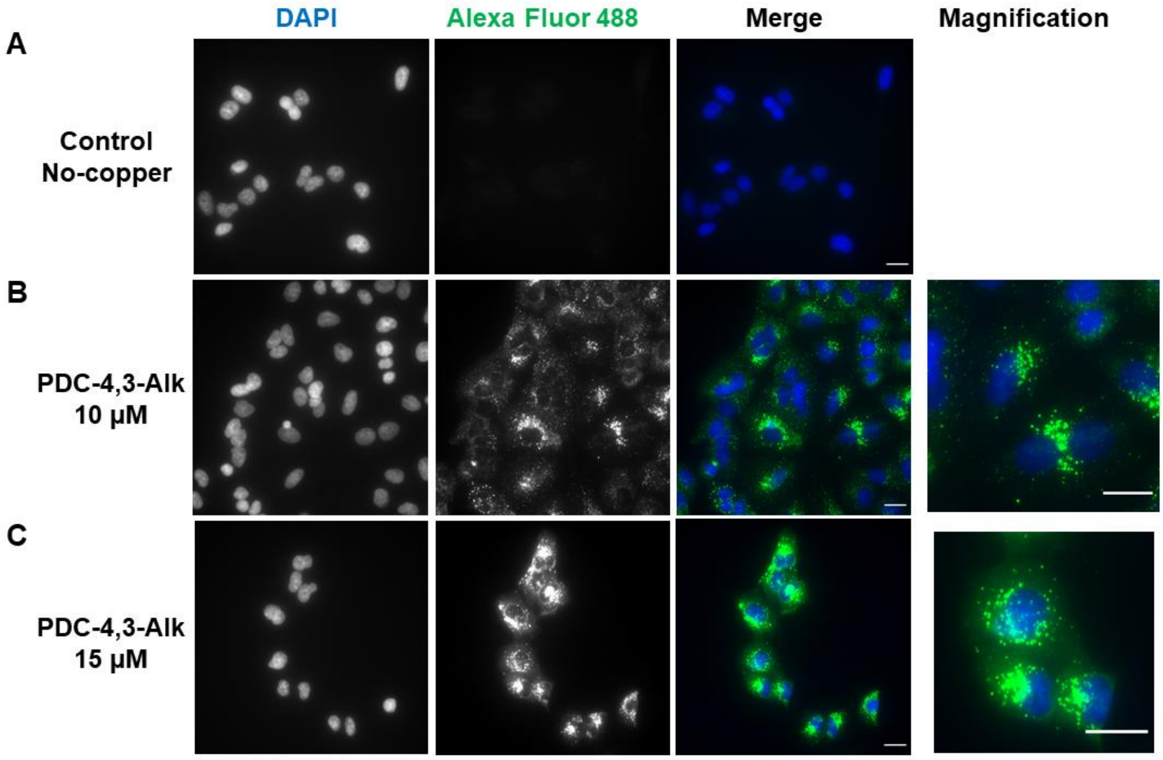

Figure S22. G4 ligand immunofluorescent staining in A549 cells and nucleus visualized with DAPI. Fluorescence wide-field microscopy images of A549 cells after 16 h incubation with (**A**) 15 µM PDC-4,3-Alk control, obtained by treatment with 5-BrdU-N3 in the absence of copper, and (**B**) 10 µM PDC-4,3-Alk, and (**C**) 15 µM PDC-4,3-Alk copper-based click reaction in the presence of 50 µM 5-BrdU-N3, 100 µM $CuSO_4{\cdot}5H_2O$, 500 µM THPTA, and 5 mM NaAsc. Images were obtained after incubation with mouse anti 5-BrdU antibody and Alexa Fluor 488 conjugated goat anti mouse secondary antibody. For each image, DAPI and Alexa Fluor 488 (FITC) channels are represented separately. Merged images are shown on the right. Images are represented as a Z-projection. Scale bar: 20 µm.

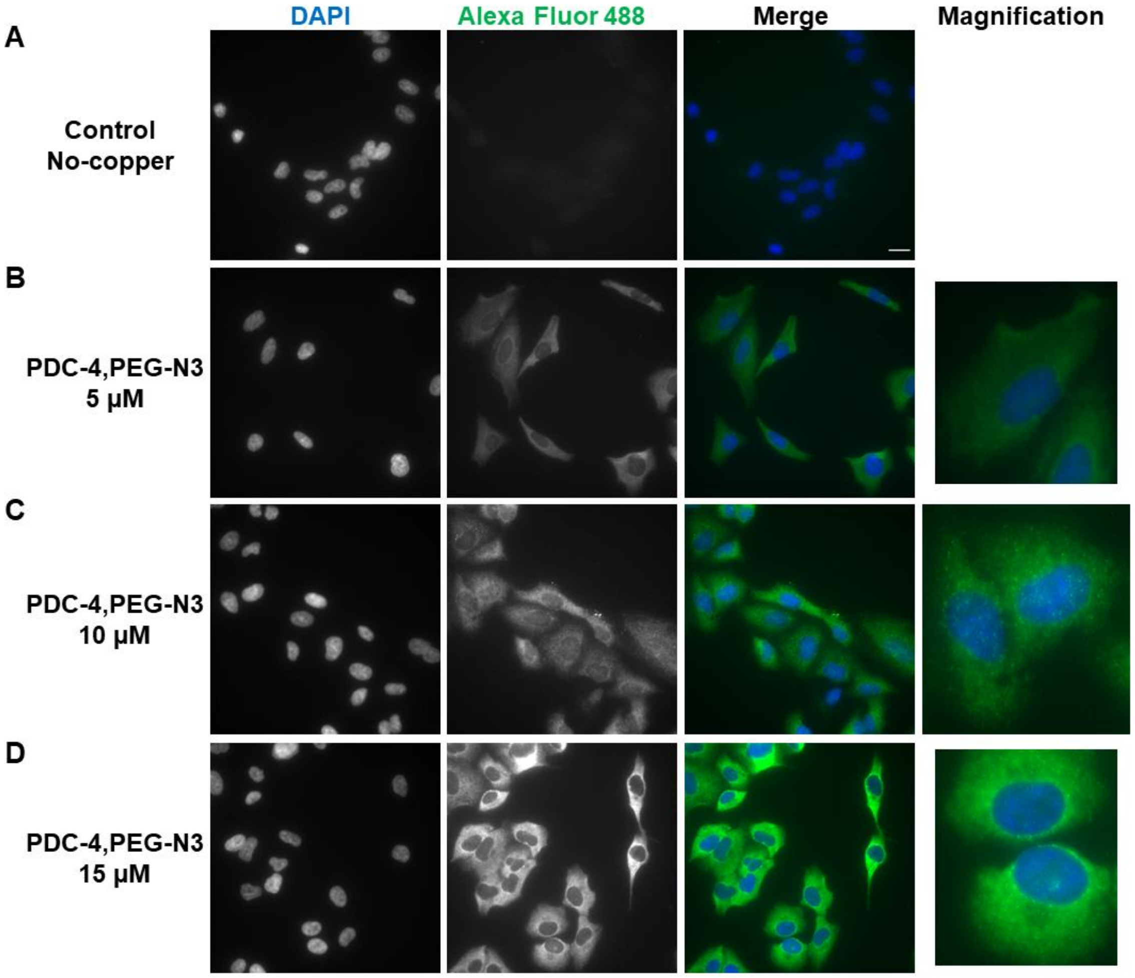

Figure S23. G4 ligand immunofluorescent staining in A549 cells and nucleus visualized with DAPI. Fluorescence wide-field microscopy images of A549 cells after 16 h incubation with (**A**) 15 µM PDC-4,PEG-N3 control, obtained by treatment with 5-BrdU-Alk in the absence of copper, and (**B**) 5 µM PDC-4,PEG-N3, (**C**) 10 µM PDC-4,PEG-N3, and (**D**) 15 µM PDC-4,PEG-N3 copper-based click reaction in the presence of 50 µM 5-BrdU-Alk, 100 µM $CuSO_4{\cdot}5H_2O$, 500 µM THPTA, and 5 mM NaAsc. Images were obtained after incubation with mouse anti 5-BrdU antibody and Alexa Fluor 488 conjugated goat anti mouse secondary antibody. For each image, DAPI and Alexa Fluor 488 (FITC) channels are represented separately. Merged images are shown on the right. Images are represented as a Z-projection. Scale bar: 20 µm.

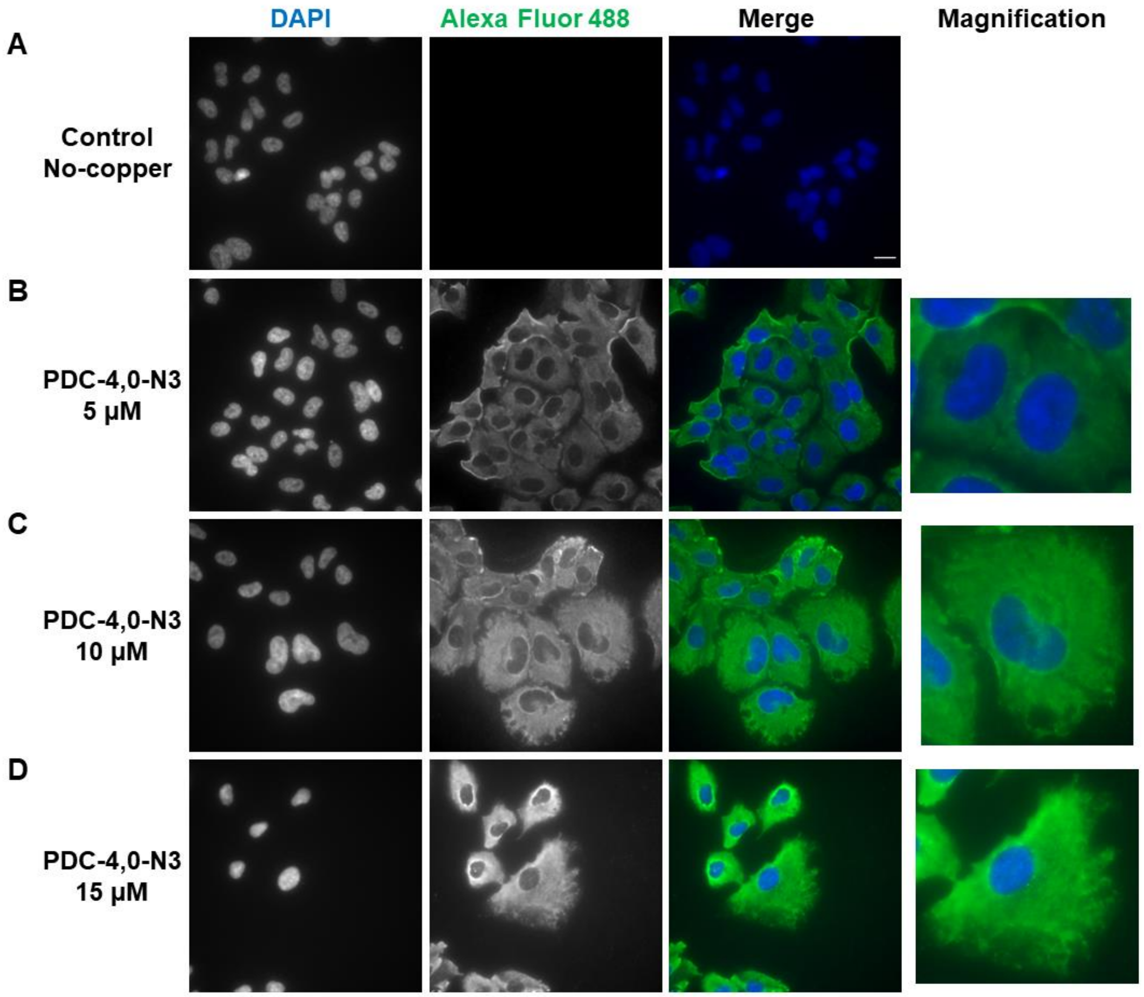

Figure S24. G4 ligand immunofluorescent staining in A549 cells and nucleus visualized with DAPI. Fluorescence wide-field microscopy images of A549 cells after 16 h incubation with (**A**) 15 µM PDC-4,0-N3 control, obtained by treatment with 5-BrdU-Alk in the absence of copper, and (**B**) 5 µM PDC-4,0-N3, (**C**) 10 µM PDC-4,0-N3, and (**D**) 15 µM PDC-4,0-N3 copper-based click reaction in the presence of 50 µM 5-BrdU-Alk, 100 µM $CuSO_4{\cdot}5H_2O$, 500 µM THPTA, and 5 mM NaAsc. Images were obtained after incubation with mouse anti 5-BrdU antibody and Alexa Fluor 488 conjugated goat anti mouse secondary antibody. For each image, DAPI and Alexa Fluor 488 (FITC) channels are represented separately. Merged images are shown on the right. Images are represented as a Z-projection. Scale bar: 20 µm.

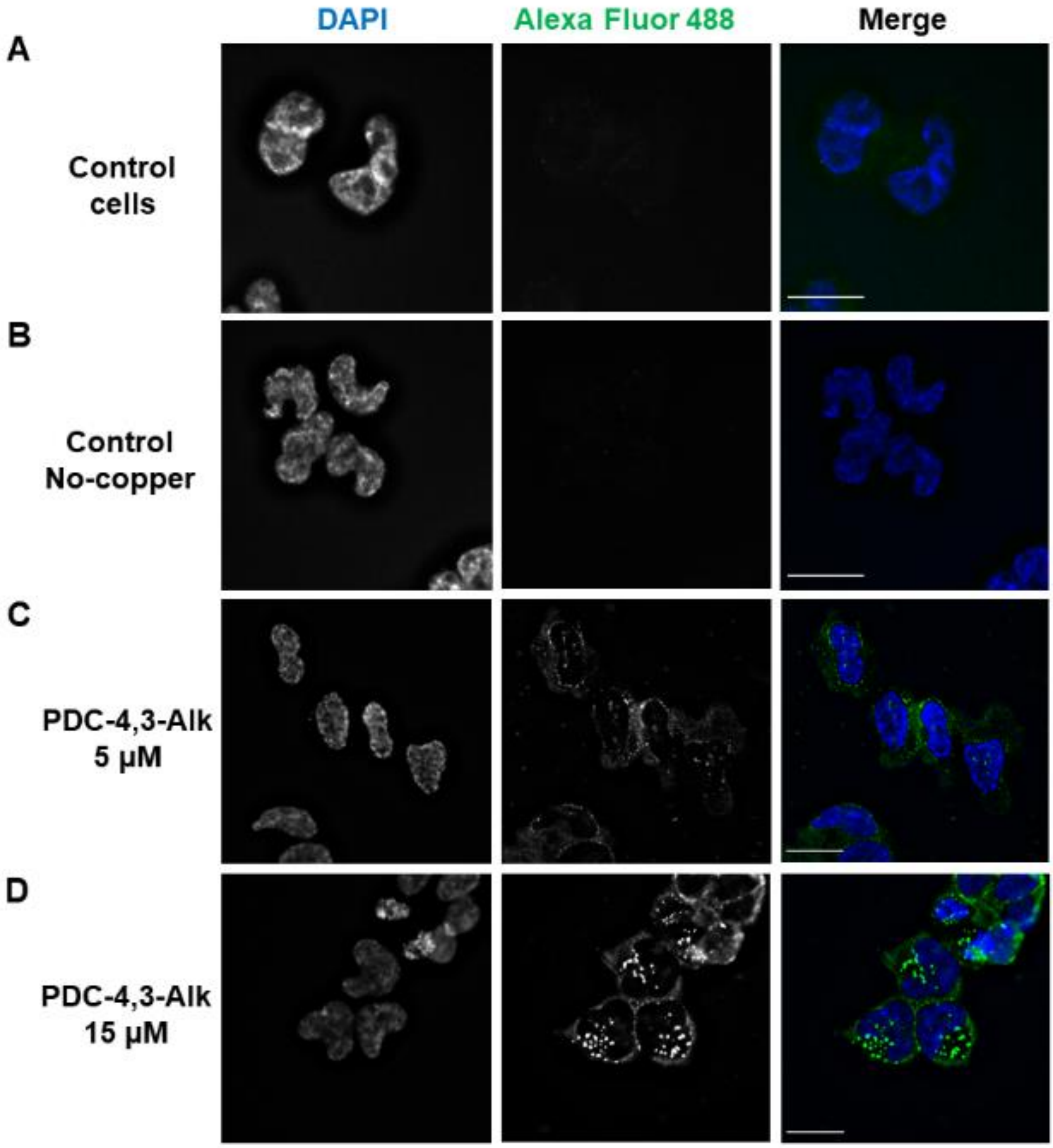

Figure S25. G4 ligand immunofluorescent staining in A2780 cells and nucleus visualized with DAPI. Fluorescence wide-field microscopy images of A2780 cells after 16 h incubation with (**A**) untreated cells, (**B**) 15 µM PDC-4,3-Alk control, obtained by treatment with 5-BrdU-N3 in the absence of copper, and (**C**) 5 µM PDC-4,3-Alk, and (**D**) 15 µM PDC-4,3-Alk, copper-based click reaction in the presence of 50 µM 5-BrdU-N3, 100 µM $CuSO_4 \cdot 5H_2O$, 500 µM THPTA, and 5 mM NaAsc. Images were obtained after incubation with mouse anti 5-BrdU antibody and Alexa Fluor 488 conjugated goat anti mouse secondary antibody. For each image, DAPI and Alexa Fluor 488 (FITC) channels are represented separately. Merged images are shown on the right. Images are represented as a Z-projection. Scale bar: 20 µm.

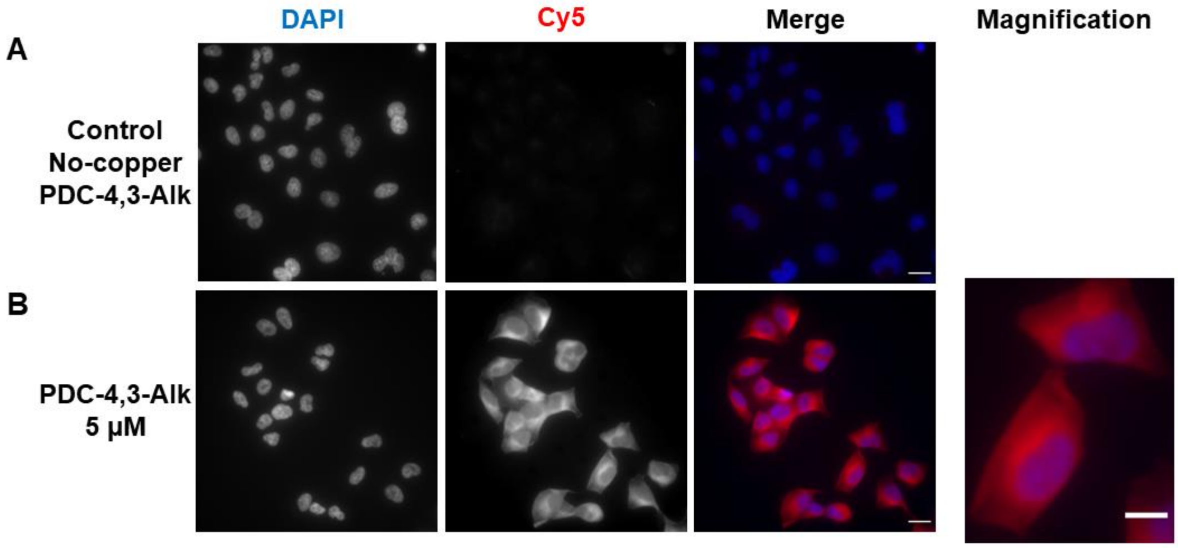

Figure S26. G4 ligand visualization in A549 cells and nucleus visualized with DAPI. Fluorescence wide-field microscopy images of A549 cells after 16 h incubation. (**A**) 5 µM PDC-4,3-Alk control, obtained by treatment with Cy5-N3 in the absence of copper, (**B**) 5 µM PDC-4,3-Alk copper-based click reaction in the presence of 10 µM Cy5-N3, 100 µM $CuSO_4{\cdot}5H_2O$, 500 µM THPTA, and 5 mM NaAsc. For each image, DAPI and Cy5 channels are represented separately. Merged images are shown on the right. Images are represented as a Z-projection. Scale bar: 20 µm. For magnification: scale bar: 10 µm.

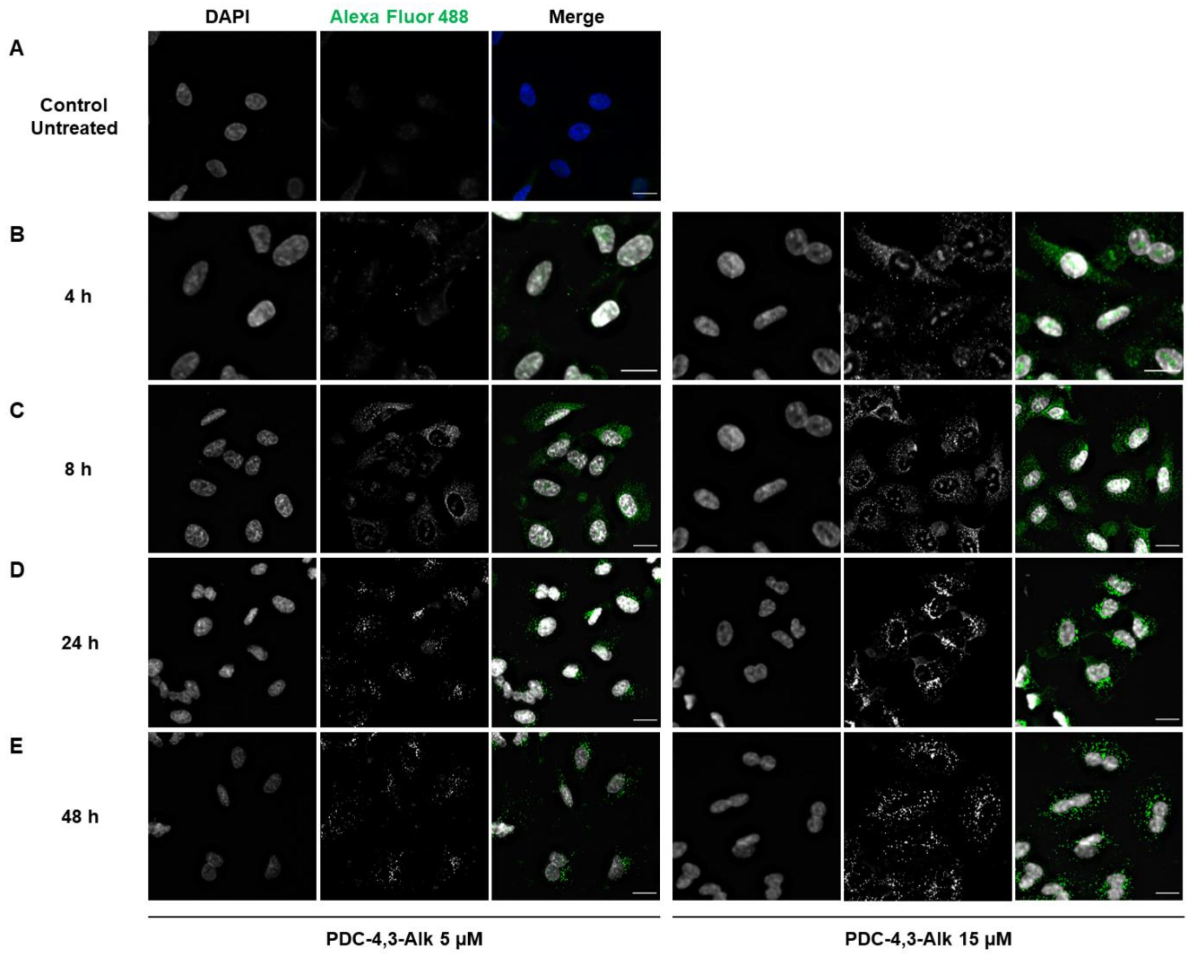

Figure S27. G4 ligand immunofluorescent staining in A549 cells and nucleus visualized with DAPI. Fluorescence wide-field microscopy images of A549 cells (**A**) control and after (**B**) 4 h, (**C**) 8 h, (**D**) 24 h, and (**E**) 48 h incubation with 5 µM PDC-4,3-Alk (right) and 15 µM PDC-4,3-Alk (left) and copper-based click reaction in the presence of 50 µM 5-BrdU-N3, 100 µM $CuSO_4{\cdot}5H_2O$, 500 µM THPTA, and 5 mM NaAsc. Images were obtained after incubation with mouse anti 5-BrdU antibody and Alexa Fluor 488 conjugated goat anti mouse secondary antibody. For each image, DAPI and Alexa Fluor 488 (FITC) channels are represented separately. Merged images are shown on the right. Images are represented as a Z-projection. Scale bar: 20 µm. To facilitate the visualization of PDC-4,3-Alk in merged images, DAPI is represented in grey.

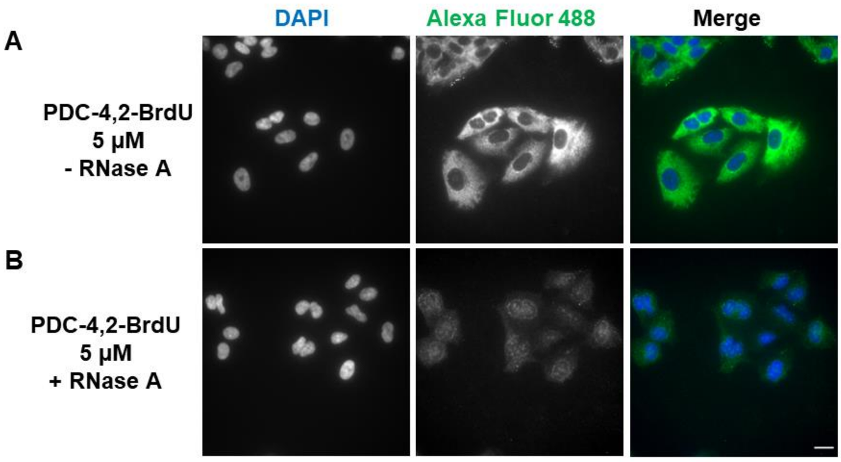

Figure S28. Comparison of untreated and RNase A treated A549 cells after incubation with 5 µM PDC-4,2-BrdU. Fluorescence wide-field microscopy images of A549 cells after 16 h incubation with 5 µM of PDC-4,2-BrdU: (**A**) non-RNase A treatment and (**B**) 0.1 mg/mL RNase A treatment at 37 °C for 1 h. Images were obtained after incubation with mouse anti 5-BrdU antibody and Alexa Fluor 488 conjugated goat anti mouse secondary antibody. For each image, DAPI and Alexa Fluor 488 (FITC) channels are represented separately. Merged images are shown on the right. Images are represented as a Z-projection. Scale bar: 20 µm.

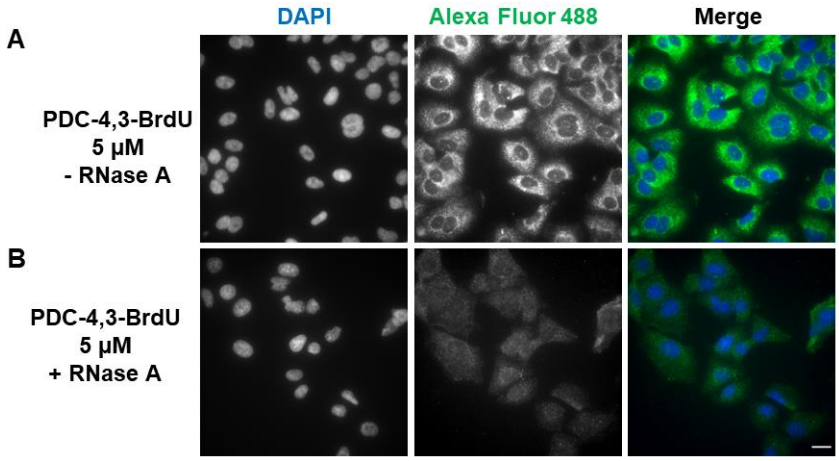

Figure S29. Comparison of untreated and RNase A treated A549 cells after incubation with 5 µM PDC-4,3-BrdU. Fluorescence wide-field microscopy images of A549 cells after 16 h incubation with 5 µM of PDC-4,3-BrdU: (**A**) non-RNase A treatment and (**B**) 0.1 mg/mL RNase A treatment at 37 °C for 1 h. Images were obtained after incubation with mouse anti 5-BrdU antibody and Alexa Fluor 488 conjugated goat anti mouse secondary antibody. For each image, DAPI and Alexa Fluor 488 (FITC) channels are represented separately. Merged images are shown on the right. Images are represented as a Z-projection. Scale bar: 20 µm.

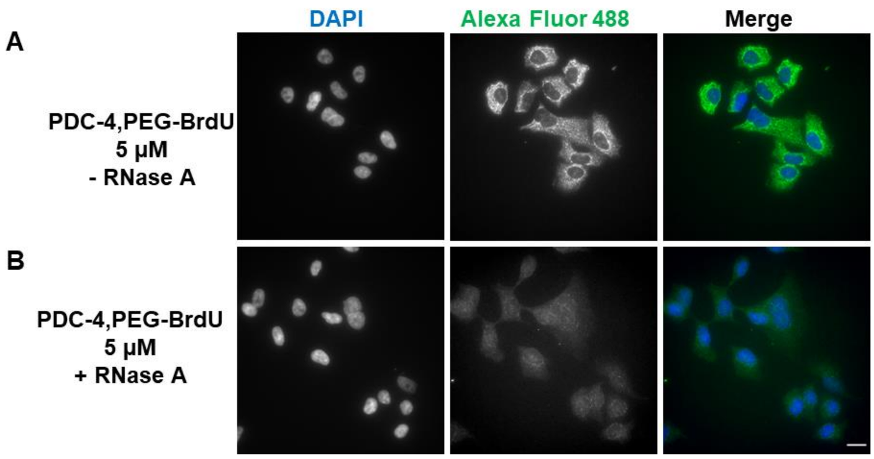

Figure S30. Comparison of untreated and RNase A treated A549 cells after incubation with 5 µM PDC-4,PEG-BrdU. Fluorescence wide-field microscopy images of A549 cells after 16 h incubation with 5 µM of PDC-4,PEG-BrdU: (**A**) non-RNase A treatment and (**B**) 0.1 mg/mL RNase A treatment at 37 °C for 1 h. Images were obtained after incubation with mouse anti 5-BrdU antibody and Alexa Fluor 488 conjugated goat anti mouse secondary antibody. For each image, DAPI and Alexa Fluor 488 (FITC) channels are represented separately. Merged images are shown on the right. Images are represented as a Z-projection. Scale bar: 20 µm.

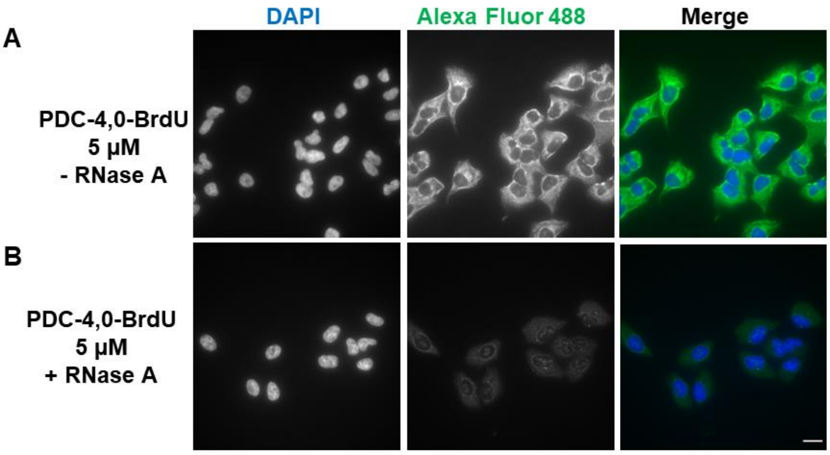

Figure S31. Comparison of untreated and RNase A treated A549 cells after incubation with 5 µM PDC-4,0-BrdU. Fluorescence wide-field microscopy images of A549 cells after 16 h incubation with 5 µM of PDC-4,0-BrdU: (**A**) non-RNase A treatment and (**B**) 0.1 mg/mL RNase A treatment at 37 °C for 1 h. Images were obtained after incubation with mouse anti 5-BrdU antibody and Alexa Fluor 488 conjugated goat anti mouse secondary antibody. For each image, DAPI and Alexa Fluor 488 (FITC) channels are represented separately. Merged images are shown on the right. Images are represented as a Z-projection. Scale bar: 20 µm.

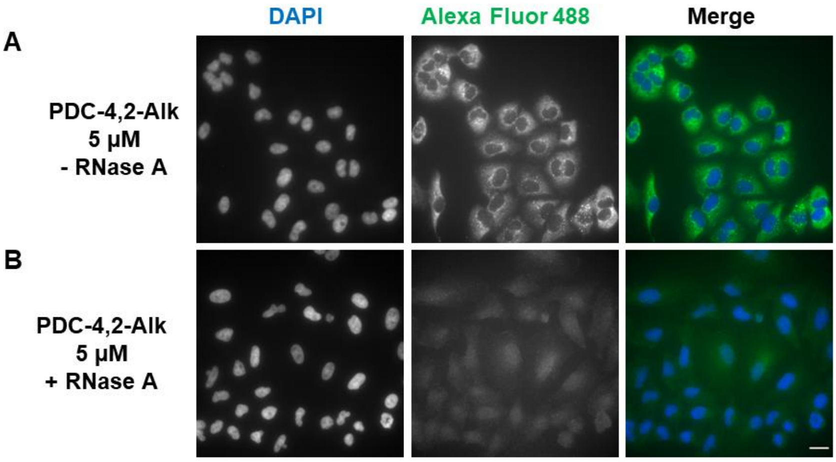

Figure S32. Comparison of untreated and RNase A treated A549 cells after incubation with 5 µM PDC-4,2-Alk. Fluorescence wide-field microscopy images of A549 cells after 16 h incubation with 5 µM of PDC-4,2-Alk and CuAAC reaction: (**A**) non-RNase A treatment and (**B**) 0.1 mg/mL RNase A treatment at 37 °C for 1 h. Images were obtained after incubation with mouse anti 5-BrdU antibody and Alexa Fluor 488 conjugated goat anti mouse secondary antibody. For each image, DAPI and Alexa Fluor 488 (FITC) channels are represented separately. Merged images are shown on the right. Images are represented as a Z-projection. Scale bar: 20 µm.

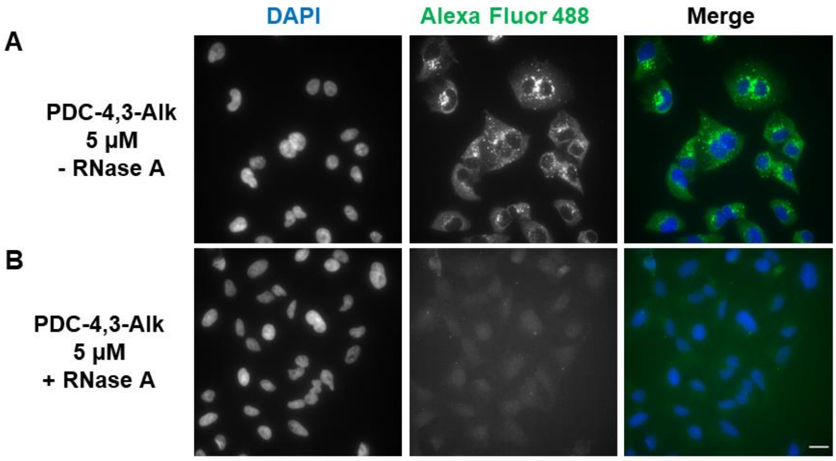

Figure S33. Comparison of untreated and RNase A treated A549 cells after incubation with 5 µM PDC-4,3-Alk. Fluorescence wide-field microscopy images of A549 cells after 16 h incubation with 5 µM of PDC-4,3-Alk and CuAAC reaction: (**A**) non-RNase A treatment and (**B**) 0.1 mg/mL RNase A treatment at 37 °C for 1 h. Images were obtained after incubation with mouse anti 5-BrdU antibody and Alexa Fluor 488 conjugated goat anti mouse secondary antibody. For each image, DAPI and Alexa Fluor 488 (FITC) channels are represented separately. Merged images are shown on the right. Images are represented as a Z-projection. Scale bar: 20 µm.

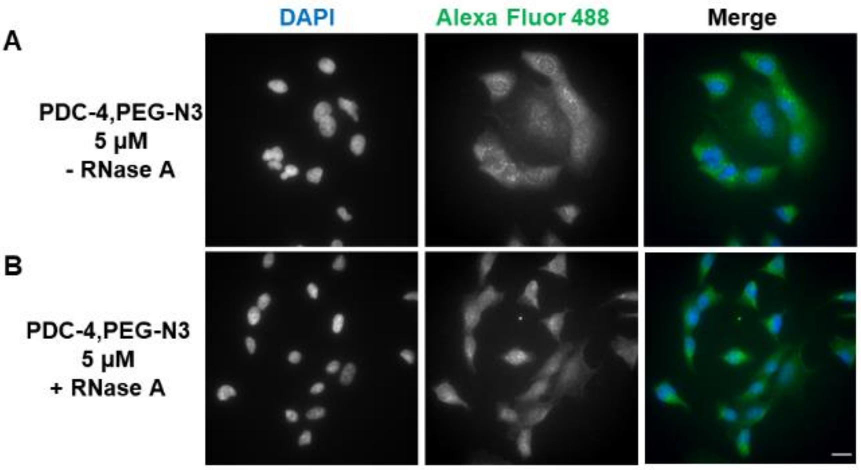

Figure S34. Comparison of untreated and RNase A treated A549 cells after incubation with 5 µM PDC-4,PEG-N3. Fluorescence wide-field microscopy images of A549 cells after 16 h incubation with 5 µM of PDC-4,PEG-N3 and CuAAC reaction: (**A**) non-RNase A treatment and (**B**) 0.1 mg/mL RNase A treatment at 37 °C for 1 h. Images were obtained after incubation with mouse anti 5-BrdU antibody and Alexa Fluor 488 conjugated goat anti mouse secondary antibody. For each image, DAPI and Alexa Fluor 488 (FITC) channels are represented separately. Merged images are shown on the right. Images are represented as a Z-projection. Scale bar: 20 µm.

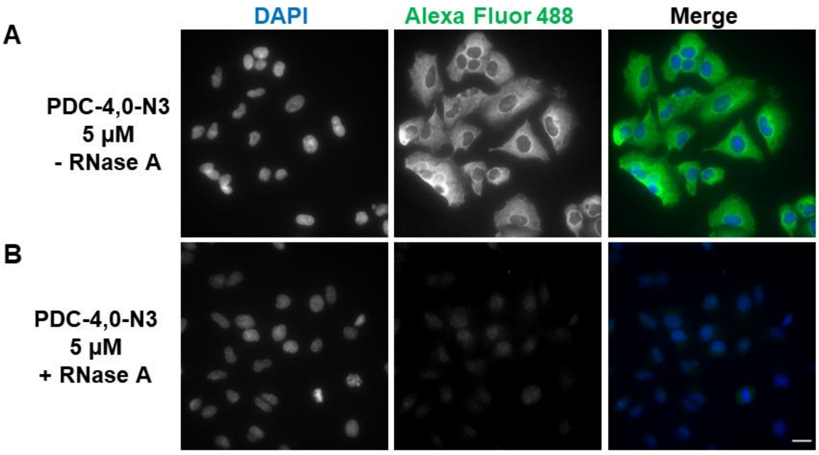

Figure S35. Comparison of untreated and RNase A treated A549 cells after incubation with 5 µM PDC-4,0-N3. Fluorescence wide-field microscopy images of A549 cells after 16 h incubation with 5 µM of PDC-4,0-N3 and CuAAC reaction: (**A**) non-RNase A treatment and (**B**) 0.1 mg/mL RNase A treatment at 37 °C for 1 h. Images were obtained after incubation with mouse anti 5-BrdU antibody and Alexa Fluor 488 conjugated goat anti mouse secondary antibody. For each image, DAPI and Alexa Fluor 488 (FITC) channels are represented separately. Merged images are shown on the right. Images are represented as a Z-projection. Scale bar: 20 µm.

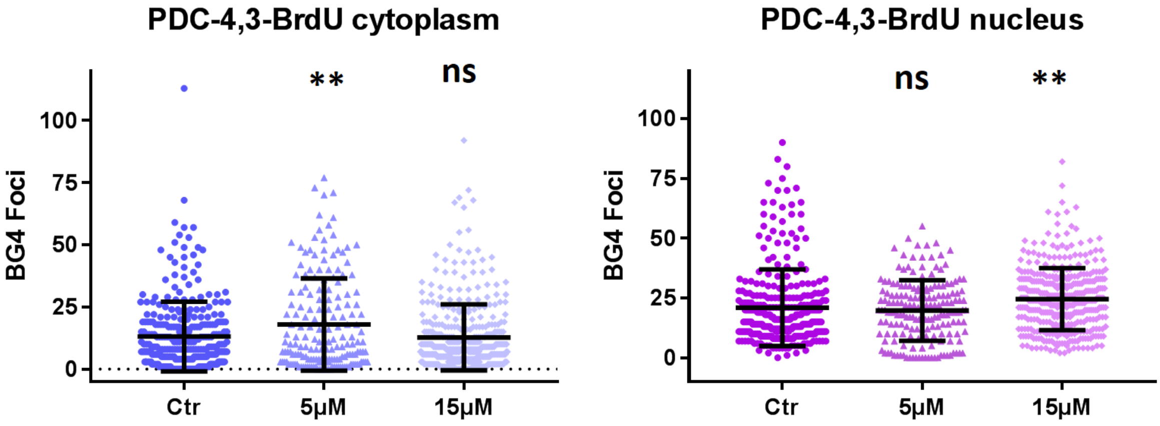

Figure S36. Column scatter plots representing the number of cytoplasmic and nuclear BG4 foci detected in control A549 cells or treated with 5 and 15 μM of PDC-4,3-BrdU for 16 hours. Mean values are indicate for each group. *P* values were calculated towards the correspondent control: ns $P > 0.05$, *$P < 0.05$, **$P < 0.01$, *t*-Student test.

References

1. Xie,X., Reznichenko,O., Chaput,L., Martin,P., Teulade-Fichou,M.P. and Granzhan,A. (2018) Topology-Selective, Fluorescent "Light-Up" Probes for G-Quadruplex DNA Based on Photoinduced Electron Transfer. *Chem. - A Eur. J.*, **24**, 12638–12651.
2. Verga,D., Hamon,F., Poyer,F., Bombard,S. and Teulade-Fichou,M.P. (2014) Photo-cross-linking probes for trapping G-quadruplex DNA. *Angew. Chemie - Int. Ed.*, **53**, 994–998.
3. Renčiuk,D., Kejnovská,I., Školáková,P., Bednářová,K., Motlová,J. and Vorlíčková,M. (2009) Arrangements of human telomere DNA quadruplex in physiologically relevant K+ solutions. *Nucleic Acids Res.*, **37**, 6625–6634.
4. Largy,E. and Mergny,J.L. (2014) Shape matters: Size-exclusion HPLC for the study of nucleic acid structural polymorphism. *Nucleic Acids Res.*, **42**, e149.
5. Amrane,S., Adrian,M., Heddi,B., Serero,A., Nicolas,A., Mergny,J.-L. and Phan,A.T. (2012) Formation of Pearl-Necklace Monomorphic G-Quadruplexes in the Human CEB25 Minisatellite. *J. Am. Chem. Soc.*, **134**, 5807–5816.
6. Piazza,A., Adrian,M., Samazan,F., Heddi,B., Hamon,F., Serero,A., Lopes,J., Teulade-Fichou,M.-P., Phan,A.T. and Nicolas,A. (2015) Short loop length and high thermal stability determine genomic instability induced by G-quadruplex-forming minisatellites. *EMBO J.*, **34**, 1718–1734.
7. Lim,K.W., Alberti,P., Guédin,A., Lacroix,L., Riou,J.F., Royle,N.J., Mergny,J.L. and Phan,A.T. (2009) Sequence variant (CTAGGG)n in the human telomere favors a G-quadruplex structure containing a G·C·G·C tetrad. *Nucleic Acids Res.*, **37**, 6239–6248.
8. Kuryavyi,V., Phan,A.T. and Patel,D.J. (2010) Solution structures of all parallel-stranded monomeric and dimeric G-quadruplex scaffolds of the human c-kit2 promoter. *Nucleic Acids Res.*, **38**, 6757–6773.
9. Collie,G.W., Haider,S.M., Neidle,S. and Parkinson,G.N. (2010) A crystallographic and modelling study of a human telomeric RNA (TERRA) quadruplex. *Nucleic Acids Res.*, **38**, 5569–5580.
10. Kumari,S., Bugaut,A., Huppert,J.L. and Balasubramanian,S. (2007) An RNA G-quadruplex in the 5′ UTR of the NRAS proto-oncogene modulates translation. *Nat. Chem. Biol.*, **3**, 218–221.
